# Intravenous ferric carboxymaltose and exercise capacity in iron-deficient kidney transplant recipients: a randomised, placebo-controlled trial in the Netherlands

**DOI:** 10.1016/j.eclinm.2026.104169

**Published:** 2026-09-01

**Authors:** Joanna Sophia J. Vinke, Michele F. Eisenga, Amarens van der Vaart, Caspar J. van Lieshout, Helma W.G.M. Dolmans, Peter van der Meer, Jozine M. ter Maaten, Jacoba M. Spikman, Aaltje L. Ziengs, Jenny E. Kootstra-Ros, Ilja M. Nolte, Carlo G.M. Gaillard, Margriet F.C. de Jong, Stefan P. Berger, Jan-Stephan F. Sanders, Stephan J.L. Bakker, Arjan D. van Zuilen, Martin H. de Borst

**Affiliations:** a Department of Nephrology, University Medical Centre Groningen, University of Groningen, Groningen, the Netherlands; b Department of Nephrology, University Medical Centre Utrecht, Utrecht, the Netherlands; c Department of Cardiology, University Medical Centre Groningen, University of Groningen, Groningen, the Netherlands; d Department of Neuropsychology, University Medical Centre Groningen, University of Groningen, Groningen, the Netherlands; e Department of Laboratory Medicine, University Medical Centre Groningen, University of Groningen, Groningen, the Netherlands; f Department of Epidemiology, University Medical Centre Groningen, University of Groningen, Groningen, the Netherlands

**Keywords:** Kidney transplantation, Iron, Exercise capacity, Anaemia

## Abstract

**Background:**

Whether correction of iron deficiency improves functional outcomes after kidney transplantation is unknown. We evaluated intravenous ferric carboxymaltose for improving exercise capacity in iron-deficient kidney transplant recipients.

**Methods:**

In this multicentre, double-blind, randomised, placebo-controlled trial, adult kidney transplant recipients with iron deficiency (ferritin <100 μg/L or 100–299 μg/L with transferrin saturation <20%) at least six months post-transplantation were recruited at two Dutch university hospitals between 7 October 2019 and 16 February 2024. Participants were assigned (1:1) to four intravenous doses of 500 mg ferric carboxymaltose or placebo at six-week intervals. The primary outcome was the between-group difference in 24-week change in 6-min walk test distance. Secondary outcomes included cardiac, kidney, muscle and cognitive function and quality of life. Efficacy was analysed by assigned treatment in 148 participants, with a per-protocol sensitivity analysis; safety analyses included 154 dosed participants. The trial was registered at ClinicalTrials.gov (NCT03769441).

**Findings:**

Of 155 participants randomised, seven were withdrawn under a prespecified COVID-19 amendment, leaving 148 participants for analysis (ferric carboxymaltose, n = 75; placebo, n = 73). Baseline 6-min walk test distance was 515 ± 91 m and 510 ± 114 m, respectively. Ferric carboxymaltose did not improve 6-min walk test distance versus placebo (adjusted mean difference +9 m [95% CI −10 to +28]). Ferric carboxymaltose corrected iron deficiency and increased haemoglobin, but did not improve cardiac function, muscle mass or strength, cognitive performance, or quality of life. Exploratory between-group differences occurred in estimated glomerular filtration rate and fibroblast growth factor 23. Infections and other clinically relevant adverse events did not differ between groups.

**Interpretation:**

Ferric carboxymaltose did not improve exercise capacity or most secondary outcomes, arguing against routine supplementation to improve functional capacity. Future studies should investigate observed differences in kidney function and fibroblast growth factor 23 concentrations and long-term clinical outcomes.

**Funding:**

10.13039/501100002997Dutch Kidney Foundation, 10.13039/100002129Dutch Heart Foundation, CSL Vifor.

**Research in context**

**Evidence before this study**

We searched PubMed, Embase, and Cochrane for randomised controlled trials, observational studies, and meta-analyses evaluating iron deficiency and iron supplementation in kidney transplant recipients, chronic kidney disease, and heart failure populations. Searches covered the period from database inception to January 1, 2026, without language restrictions. Search terms included combinations of “kidney transplantation”, “iron deficiency”, “intravenous iron”, “ferric carboxymaltose”, “exercise capacity”, “muscle strength”, “cardiac function”, “cognitive function”, and “quality of life”. Observational studies consistently showed associations between iron deficiency and reduced exercise capacity, impaired muscle strength, worse quality of life, neurocognitive dysfunction, and increased mortality in kidney transplant recipients. In contrast, randomised trials demonstrating clinical benefits of intravenous iron were largely restricted to patients with chronic heart failure or chronic kidney disease, with no inclusion of kidney transplant recipients. No randomised placebo-controlled trial had evaluated the effect of iron deficiency correction on exercise capacity and a broad range of functional outcomes after kidney transplantation.

**Added value of this study**

This multicentre, double-blind, randomised placebo-controlled trial is the first to evaluate the effects of intravenous ferric carboxymaltose on exercise capacity and multiple secondary outcomes in iron-deficient kidney transplant recipients. Despite effective correction of iron deficiency and increases in haemoglobin, ferric carboxymaltose did not improve exercise capacity, cardiac function, muscle mass, cognitive performance, fatigue, or quality of life compared with placebo. Exploratory between-group differences were observed for circulating fibroblast growth factor 23 levels and kidney function in this population, without increasing adverse events.

**Implications of all the available evidence**

Taken together, the available evidence indicates that although iron deficiency is common and associated with adverse outcomes after kidney transplantation, routine correction of iron deficiency with intravenous iron does not improve exercise capacity or most patient-centred outcomes in stable kidney transplant recipients. These findings suggest that extrapolation of benefits observed in heart failure and chronic kidney disease populations to kidney transplant recipients is not justified. Future research should focus on identifying specific subgroups who may benefit from iron supplementation, elucidating the mechanisms underlying the observed effects on kidney function and fibroblast growth factor 23, and evaluating long-term clinical outcomes.

## Introduction

Although kidney transplantation substantially improves survival and quality of life compared with dialysis, kidney transplant recipients remain a population with substantial residual disease burden. Long-term exposure to chronic kidney disease, cardiovascular disease, metabolic complications, and lifelong immunosuppression results in complex multimorbidity, persistent inflammation, and impaired physical functioning.

Consequently, reduced exercise tolerance and low physical activity levels are common among kidney transplant recipients, even years after transplantation.^1^^,^^2^ Importantly, impaired exercise capacity and reduced muscle strength in kidney transplant recipients are strongly associated with increased mortality and inferior graft outcomes, underscoring functional capacity as a clinically meaningful therapeutic target.^3^^,^^4^

Iron deficiency affects approximately 30% of kidney transplant recipients and represents a potentially modifiable contributor to impaired physical functioning in this population.^5^^,^^6^ Observational studies have consistently linked iron deficiency to adverse outcomes, including reduced exercise capacity, neurocognitive dysfunction,^7^ impaired health-related quality of life,^8^ and premature mortality.^5^ In patients with chronic heart failure and chronic kidney disease, iron deficiency has similarly been associated with impaired exercise tolerance,^9^, ^10^, ^11^, ^12^ poor skeletal muscle strength^13^ and quality of life^10^, ^11^, ^12^^,^^14^^,^^15^ and worse prognosis.^11^^,^^15^, ^16^, ^17^, ^18^

In these non-transplant populations, randomised controlled trials have demonstrated that iron deficiency correction with intravenous iron improves exercise capacity and quality of life,^19^, ^20^, ^21^, ^22^, ^23^ and may improve left ventricular function.^24^^,^^25^ These effects are thought to be mediated not only through increased haemoglobin concentrations, but also through improved cellular energy metabolism and modulation of fibroblast growth factor 23 (FGF23), a hormone implicated in cardio-renal pathology and muscle dysfunction.^26^^,^^27^ Transplant recipients differ fundamentally from patients with chronic heart failure or chronic kidney disease due to immunosuppressive therapy, transplant-specific metabolic disturbances, and competing contributors to functional impairment, making extrapolation of trial data potentially inappropriate.

Despite the high prevalence of iron deficiency and the clinical relevance of impaired exercise capacity after kidney transplantation, randomised placebo-controlled trials evaluating the effects of intravenous iron supplementation on functional outcomes in kidney transplant recipients are lacking. To provide evidence regarding this important knowledge gap, we performed the Effect of Ferric Carboxymaltose on Exercise Capacity after Kidney Transplantation (EFFECT-KTx) study, a multicentre double-blind randomised controlled trial designed to evaluate the effects of intravenous iron supplementation on exercise capacity, skeletal muscle strength, cardiac structure and function, kidney function, quality of life and cognitive performance in iron-deficient kidney transplant recipients.

## Methods

### Study design and participants

The EFFECT-KTx study was an investigator-initiated, double-blind, randomised, placebo-controlled superiority trial with two parallel treatment arms. The trial was prospectively registered at ClinicalTrials.gov (NCT03769441; first posted on 7 December 2018; https://clinicaltrials.gov/study/NCT03769441). The study was conducted at two centres in the Netherlands: University Medical Centre Groningen (UMCG) and University Medical Centre Utrecht (UMCU). Participants were screened and recruited between October 2019 and February 2024. The study design has been published previously.^28^ Adult kidney transplant recipients with iron deficiency, defined as plasma ferritin <100 μg/L or plasma ferritin 100–299 μg/L with transferrin saturation (TSAT) < 20%,^20^ who were at least six months after transplantation, were eligible for participation. Exclusion criteria included recent gastrointestinal or urogenital blood loss, severe (haemoglobin <10.5 g/dL) or progressive anaemia, a reduced mean corpuscular volume (MCV <80 fL), polycythaemia (haemoglobin >15.3 g/dL), an estimated glomerular filtration rate (eGFR) < 30 mL/min/1.73 m^2^ or active systemic infection. A full list of inclusion and exclusion criteria is provided in Supplementary Table S1. Between March and May 2020, no in-hospital study visits were allowed because of the severe acute respiratory syndrome coronavirus 2 (SARS-CoV-2) pandemic. Given the uncertainty about the duration of the pandemic, it was decided that for participants who missed two or more study treatments, of which at least one because of restrictions related to the pandemic, participation would be terminated. In that case, the participant would not be included in the completion of the aimed sample size, and a new participant would be recruited instead. Outcomes of those participants were not included in the efficacy analyses and outcomes were therefore not imputed. Participants who were excluded because of two or more missed study drug administrations during the pandemic were invited for a rescreening under a new study number, providing they were still iron deficient and still met the other inclusion criteria. This procedure was approved by the medical ethical committee of the UMCG and resulted in the termination of participation of seven participants, of whom three re-enrolled.

### Ethics

The protocol was approved by the institutional review board of the UMCG (METc 2018/482) and the study has been conducted in accordance with the principles of the Declaration of Helsinki and Good Clinical Practice Guidelines. All participants provided written informed consent.

### Randomisation and masking

Participants were randomly assigned in a 1:1 ratio using computer-generated block randomisation with block sizes of four, with two allocations to ferric carboxymaltose and two to placebo in varying order within each block. Randomisation was performed by the pharmacy of the coordinating study centre. Participants and investigators were blinded to treatment allocation. Data analysts were not blinded to treatment allocation. Medication was administered by a research nurse not involved in outcome assessment. To maintain treatment blinding, non-transparent infusion lines (Codan, Lensahn, Germany) or opaque covers (Medipak, Winchester, Virginia, USA) were used. Additionally, infusion lines and bags were covered with aluminium foil.

### Treatment procedures

Patients received either 10 mL ferric carboxymaltose, containing 500 mg of elemental iron, diluted in 250 mL sodium chloride 0.9% or placebo (250 mL sodium chloride 0.9%) at the baseline visit and at subsequent visits at weeks 6, 12 and 18. In case of a systemic infection or a plasma phosphate level ≤0.50 mmol/L on the day of the scheduled treatment, no study medication was administered. In case of a plasma ferritin level of ≥800 μg/L, or 500–799 μg/L combined with a TSAT of ≥45%, placebo was administered regardless the treatment arm.

### Data collection

Body weight and blood pressure were recorded at all study visits prior to study treatment. Blood pressure was measured three times in sedentary position, and the mean value was used. Blood samples were collected at all study visits prior to study treatment; levels of plasma iron, ferritin and transferrin were concealed to maintain blinding of treatment allocation. Participants collected a 24-h urine sample before all study visits. Ethnicity was recorded based on self-report in the study database. Sex was recorded as a categorical variable (male, female, other) based on the corresponding field in the electronic health record of the patient. Data on gender identity were not collected. Details of all other assessments are provided below and in the Supplementary Methods.

### Outcomes

The primary outcome was the change in 6-min walk test (6MWT) distance between baseline and week 24.^29^^,^^30^ Secondary outcomes were prespecified in the study protocol (version 6.2, provided as Supplementary Material) and trial registry and covered multiple physiological domains:

**Iron status and haematinic parameters** were evaluated by changes in haemoglobin, haematocrit, MCV, plasma ferritin, TSAT, and plasma iron levels.

**Mineral metabolism** was evaluated by measurement of plasma phosphate, calcium, parathyroid hormone (PTH), and total and intact plasma FGF23 levels, as well as 24-h urinary phosphate and calcium excretion.

**Skeletal muscle mass and function** were evaluated using 24-h urinary creatinine excretion as a proxy for muscle mass^31^ and functional tests including the Timed Up-and-Go test,^32^ handgrip dynamometry,^33^ and five-times sit to stand test.^34^

**Cardiac structure and function** were evaluated by transthoracic echography, including measurements of left ventricular ejection fraction, diastolic function, and cardiac strain. In addition, circulating biomarkers including plasma N-terminal pro-B-type natriuretic peptide (NT-proBNP) and high-sensitivity troponin were measured. The HFA-PEFF score^35^ was calculated as a post-hoc derived parameter of heart failure with preserved ejection fraction (HFpEF).

**Kidney function and injury** were evaluated using the 2021 CKD-EPI creatinine and creatinine-cystatin C eGFR equations, the 2012 CKD-EPI cystatin C eGFR equation^36^ and creatinine clearance, and 24-h urinary excretion of protein and albumin. Kidney injury markers included kidney injury molecule-1 (KIM-1) and neutrophil gelatinase-associated lipocalin (NGAL).

**Cognitive function** was evaluated using a neuropsychological test battery, including tests of memory (Digit Span Forward Test,^37^ 15 Word Test^38^), executive function and cognitive flexibility (Trail Making Test parts A and B^39^ and Controlled Oral Word Association Test^40^), attention and processing speed (Symbol Digit Modalities Test^41^) and working memory (Digit Span Backward Test^37^). Six participants were excluded for neuropsychological assessment because of an estimated intelligence quotient of <80 (n = 4) based on the Dutch version of the National Adult Reading Test^42^ or because of possible neurodegenerative disease based on a cognitive screening test.^43^

**Quality of life and fatigue** were evaluated using the Short Form 36 (SF36),^44^ Checklist Individual Strength (CIS),^45^ Dutch Multifactor Fatigue Scale (DMFS)^46^ and EuroQol-5D-5L^47^ questionnaires. Restless legs complaints were also evaluated.

**Safety outcomes** included the incidence of infections, hospitalisations, cardiovascular events, graft failure or rejection, and gastrointestinal symptoms assessed using the Gastrointestinal Symptom Rating Scale (GSRS).^48^

All prespecified secondary outcomes according to the study protocol and trial registry are detailed in Supplementary Table S2. Some prespecified outcomes are not reported in the present manuscript. Serum hepcidin, plasma C-reactive protein, some kidney injury markers (MCP-1, CD163, CD25, FOXP3 mRNA), immunological parameters, including peripheral blood mononuclear cell (PBMC) counts and cytokine and antibody expression, and intestinal microbiota analyses will be presented in separate manuscripts. Vitamin D levels were prespecified but ultimately not measured due to logistical constraints and are therefore not reported. Although plasma creatinine was prespecified, results are presented as eGFR as a clinically more informative derived parameter (post-hoc). SARS-CoV-2 vaccination responses were assessed in a predefined substudy and have been published previously.

The following outcomes were analysed post-hoc in the present manuscript: individually predicted 6MWT distance based on sex, age, height, and weight using a published reference equation,^49^ post-hoc derived HFA-PEFF score^35^ based on echocardiographic parameters and plasma NT-proBNP level, post-hoc derived eGFR based on creatinine and cystatin C equations, and post-hoc analyses of urinary sodium concentration, fractional sodium excretion, and 24-h urinary excretion of creatinine, protein and albumin.

### Statistical analysis

We calculated that a sample size of 144 participants (72 per arm) was required to detect a ≥10% improvement in 6MWT, based on an average baseline 6MWT distance of 495 ± 92 m as previously observed in stable kidney transplant recipients,^1^ with 90% power and a two-sided alpha of 0.05.

Normally distributed data are presented as mean ± standard deviation, data with a skewed distribution as median (interquartile range [IQR]) and categorical data as number (percentage). Data analysis for efficacy was performed primarily according to the intention-to-treat principle for the allocated treatment in the prespecified efficacy analysis population of 148 participants. Participants withdrawn under the prespecified COVID-19 protocol amendment were replaced if they remained eligible for study participation, were excluded from the efficacy analysis population, and therefore did not have their outcomes imputed.

For all continuous outcomes measured at baseline and week 24 only, including the primary outcome, differences in change from baseline between treatment groups were evaluated using baseline-adjusted linear regression models with treatment centre and baseline value as covariates. Missing outcome data were subjected to multiple imputation (Rubin's rule,^50^ five datasets), assuming they were missing at random. Because missing primary outcome data occurred only in the ferric carboxymaltose arm, imputation of the primary outcome was performed after stratification by treatment allocation, using data from the ferric carboxymaltose arm only. The imputation model included demographic and clinical baseline variables considered predictive of missingness and outcome, including age, sex, ethnicity, cardiovascular comorbidity, diabetes mellitus, smoking status, alcohol use, dialysis history, transplant-related characteristics, immunosuppressive medication use, body mass index, haemoglobin, C-reactive protein, ferritin, TSAT, and baseline eGFR. A supportive per-protocol sensitivity analysis of the primary endpoint was performed (Supplementary Table S3). In the per-protocol set, participants violating inclusion/exclusion criteria (n = 0), participants who had a 6MWT that was not considered valid by the blinded researcher (n = 18), participants who were randomised in the intervention arm but did not receive study medication on three or more study visits (n = 2) and participants who received a blood transfusion, erythropoiesis-stimulating agents or other iron supplements besides the study treatment (n = 5) were excluded. Continuous variables assessed at more than two study visits were analysed using linear mixed-effects model analyses based on all observed timepoints jointly. Time was included in the model as a categorical factor (baseline, week 6, week 12, week 18, and week 24). Fixed effects included study site, treatment arm, time, and the treatment arm-by-time interaction. An intercept per participant was included as a random effect. Treatment effects at each follow-up visit were estimated from the treatment arm-by-time interaction and are reported as estimated marginal means and between-group differences with 95% confidence intervals. Results are presented for all study visits, including the end-of-study visit at week 24. No adjustment for multiple testing was performed for secondary outcomes, which should therefore be interpreted cautiously.

Safety analyses included all participants who received at least one dose of study treatment or placebo, irrespective of protocol violations and data completeness.

The primary endpoint was also evaluated in pre-specified subgroups defined in the statistical analysis plan based on baseline anaemia status (haemoglobin < or ≥13 g/dL in men and < or ≥12 g/dL in women), ferritin level (< or ≥100 μg/L), TSAT (< or ≥20%), eGFR (< or ≥60 mL/min/1.73 m^2^) and 6MWT distance (< or ≥ median). The prespecified subgroup analysis based on left ventricular ejection fraction (<50% versus ≥ 50%) was not performed because of the very small number of participants with reduced ejection fraction. Additional subgroup analyses based on sex and NT-proBNP were conducted post-hoc.

A p value < 0.05 was considered statistically significant. IBM SPSS Statistics version 28.0 (SPSS Inc., Chicago, USA) and Prism version 8.0 (GraphPad Software Inc., San Diego, USA) were used for data analysis and visualisation.

### Patient and public involvement

During the feasibility stage, a concept version of the project plan was presented to the Dutch Kidney Patient Association (NVN) and was reviewed by an NVN employee and a kidney patient. Their recommendations were followed in the further design of the study.

### Role of the funding source

The study was funded by the Dutch Kidney Foundation (grant no. 17OKG18), the Dutch Heart Foundation (Reconnext Fellowship, grant no. 20241085) and CSL Vifor. The funders had no role in study design, data collection, data analysis, data interpretation, or writing of the report.

## Results

**Fig. 1:**
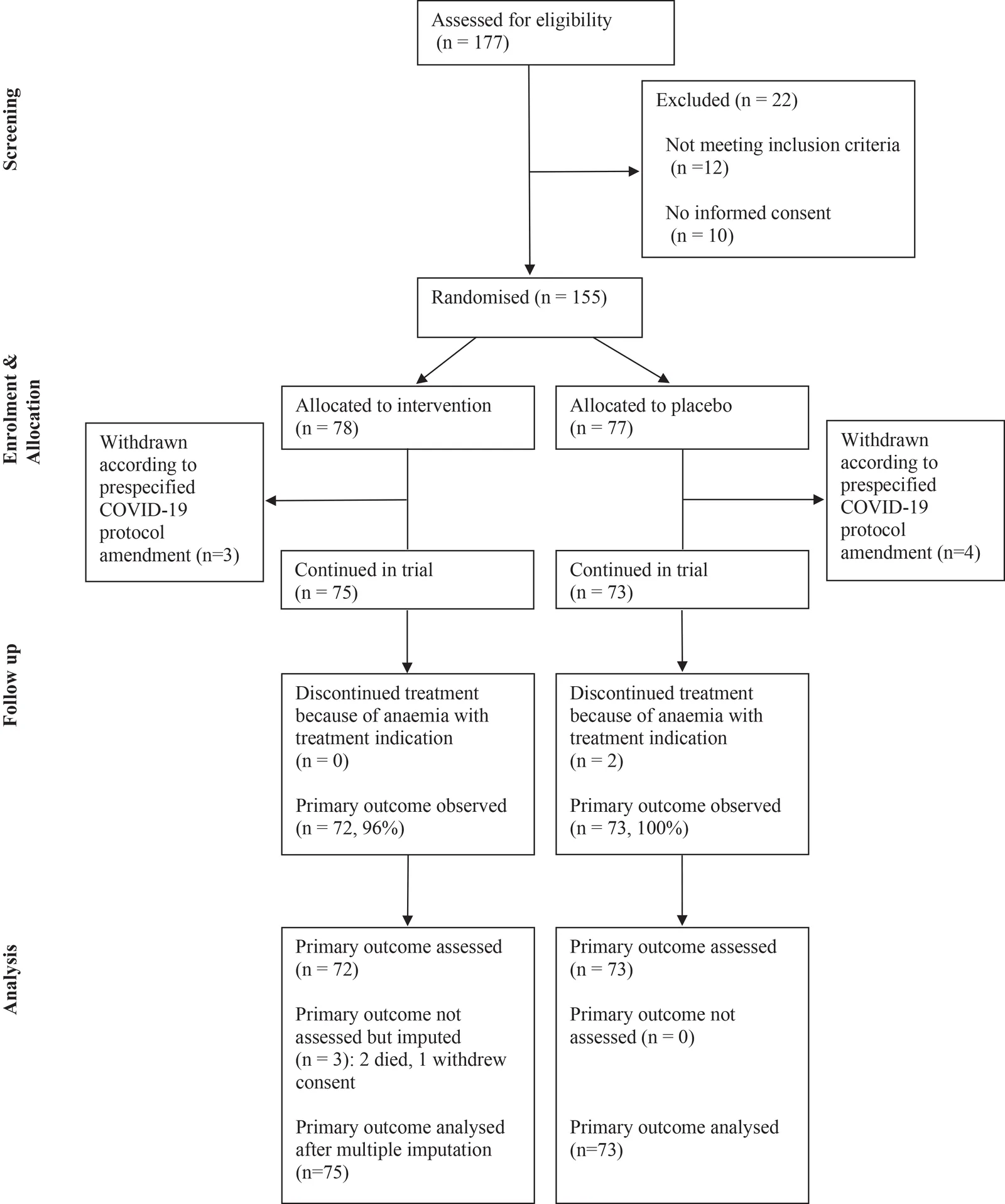
Study profile.

During the screening and recruitment phase of the study, 155 patients were randomised: 78 into the ferric carboxymaltose group and 77 into the placebo group (Fig. 1). During the study, seven participants were withdrawn according to a prespecified COVID-19 protocol amendment because repeated study visits could not be performed during the SARS-CoV-2 pandemic (Fig. 1). In accordance with the approved study protocol, these participants were replaced if they remained eligible for study participation. Consequently, 148 participants comprised the prespecified efficacy analysis population (75 assigned to ferric carboxymaltose and 73 to placebo). The safety population comprised 154 participants who received at least one dose of study treatment or placebo (77 in each group).

Baseline characteristics were similar among both study arms (Table 1). Median age was 57 years, 101 (68%) of the participants were male and 138 (93%) were White. Most participants (n = 87, 59%) had received dialysis treatment prior to transplantation, and the median time since transplantation was two years. Most kidney transplant recipients received triple immunosuppressive therapy consisting of prednisolone (n = 144, 97%), a calcineurin inhibitor (n = 133, 90%) and an antiproliferative agent (n = 122, 82%). The median plasma ferritin level was 47 μg/L, mean plasma TSAT was 20% and mean haemoglobin level was 13.2 g/dL. Almost all participants (n = 133, 90%) had a plasma ferritin level <100 μg/L, 75 (51%) had a plasma TSAT <20% and 46 (31%) had anaemia.

**Table 1:** Baseline characteristics.

Characteristics	FCM (n = 75)	Placebo (n = 73)
**Centre**		
University Medical Centre Groningen, n (%)	70 (93)	67 (92)
University Medical Centre Utrecht, n (%)	5 (7)	6 (8)
**Participant characteristics**		
Age, years	59 (50–65)	55 (44–67)
Male sex, n (%)	49 (65)	52 (71)
Female sex, n (%)	26 (35)	21 (29)
Body mass index, kg/m^2^	27 ± 4	26 ± 4
Ethnic background		
White, n (%)	69 (92)	69 (95)
South Asian, n (%)	3 (4)	0
East Asian, n (%)	0	2 (3)
Sub-Saharan African, n (%)	1 (1)	1 (1)
West Asian, n (%)	1 (1)	0
North African, n (%)	0	1 (1)
Unknown or other, n (%)	1 (1)	0
Diabetes mellitus		
Type 1, n (%)	4 (5)	4 (6)
Type 2 or steroid-associated, n (%)	24 (32)	17 (23)
Hypertension, n (%)	64 (85)	66 (90)
Coronary artery disease, n (%)	13 (17)	11 (15)
Heart failure, n (%)	3 (4)	8 (11)
History of cerebrovascular disease, n (%)	12 (16)	7 (10)
History of thrombosis, n (%)	7 (9)	5 (7)
Alcohol consumption, units per week		
None, n (%)	37 (49)	31 (43)
1–4, n (%)	22 (29)	29 (40)
5–7, n (%)	7 (9)	5 (7)
8–14, n (%)	8 (10)	4 (6)
>14, n (%)	1 (1)	4 (6)
Current tobacco use, n (%)	3 (4)	11 (15)
**Kidney disease diagnoses**		
Glomerulonephritis, n (%)	19 (25)	19 (26)
Cystic kidney disease, n (%)	15 (20)	18 (25)
Diabetes mellitus, n (%)	11 (15)	6 (8)
Hypertension, n (%)	5 (7)	10 (14)
Urological disease, n (%)	4 (6)	5 (7)
Thrombotic microangiopathy, n (%)	0	4 (6)
Other, n (%)	22 (29)	15 (21)
Unknown, n (%)	6 (8)	8 (11)
**Transplantation characteristics**		
Time since transplantation, years	2 (1–8)	3 (1–5)
Donation type		
Donation after cardiac death, n (%)	10 (13)	10 (14)
Donation after brain death, n (%)	10 (13)	9 (12)
Living related, n (%)	22 (29)	26 (36)
Living unrelated, n (%)	33 (44)	28 (38)
History of dialysis, n (%)	45 (60)	42 (58)
Remaining arteriovenous fistula, n (%)	9 (12)	9 (12)
History of graft rejection, n (%)	17 (23)	10 (14)
**Induction therapy**		
Interleukin 2 receptor antagonist, n (%)	47 (63)	40 (55)
Lymphocyte depleting agent, n (%)	2 (3)	5 (7)
**Medication use**		
Oral iron preparation, n (%)	1 (1)	3 (4)
Erythropoiesis-stimulating agent, n (%)	1 (1)	1 (1)
Prednisolone use, n (%)	74 (99)	70 (96)
Calcineurin inhibitor use, n (%)	68 (91)	65 (89)
Tacrolimus, n (%)	66 (88)	62 (85)
Ciclosporin, n (%)	2 (3)	3 (4)
Antiproliferative agent use, n (%)	61 (81)	61 (84)
Mycophenolic acid use, n (%)	58 (77)	60 (82)
Azathioprine use, n (%)	3 (4)	1 (1)
mTOR inhibitor use, n (%)	7 (9)	1 (1)
Proton pump inhibitor, n (%)	51 (68)	45 (62)
Vitamin D supplement, n (%)	8 (11)	15 (21)
Phosphate supplement, n (%)	2 (3)	3 (4)
Calcium supplement, n (%)	64 (85)	65 (89)
Calcium-regulating agent, n (%)	5 (7)	9 (12)
Angiotensin-converting-enzyme inhibitor, n (%)	16 (21)	22 (30)
Angiotensin II receptor blocker, n (%)	14 (19)	6 (8)
Antiplatelet and anticoagulant medication, n (%)	47 (63)	47 (64)
Acetylsalicylic acid, n (%)	14 (19)	11 (15)
P2Y12 inhibitor, n (%)	6 (8)	4 (6)
Low molecular weight heparin, n (%)	0	0
Vitamin K antagonist, n (%)	3 (4)	6 (8)
Direct anticoagulant agent, n (%)	7 (9)	7 (10)
**Laboratory parameters**		
Haemoglobin, g/dL	13.2 ± 1.3	13.4 ± 1.3
Anaemia, n (%)	26 (35)	20 (27)
MCV, fL	89 ± 6	90 ± 5
CRP, mg/L	1.5 (0.6–3.1)	1.3 (0.7–3.4)
eGFR, mL/min/1.73 m^2^	59 ± 16	62 ± 19
Ferritin, μg/L	48 (27–70)	47 (27–72)
TSAT, %	19 ± 9	22 ± 11
PTH, pmol/L	8.1 (6.0–10.8)	7.3 (5.3–10.3)
Phosphate, mmol/L	0.85 (0.72–0.94)	0.87 (0.72–0.99)
NT-proBNP, ng/L	153 (67–289)	157 (64–276)
**Cardiac function**		
LVEF <50%, n (%)	4 (5)	2 (2)
HFA-PEFF score <2, n (%)	22 (29)	24 (33)
HFA-PEFF score 2–4, n (%)	42 (56)	39 (53)
HFA-PEFF score 5–6, n (%)	11 (15)	10 (14)
**Exercise capacity**		
6MWT, metres	515 ± 91	510 ± 114

Baseline characteristics, assessed on the morning before the first treatment with ferric carboxymaltose (FCM) or placebo. Data are presented as mean ± standard deviation (s.d.), median with interquartile range (IQR) or number (n) with percentage (%). Participants could have more than one underlying kidney disease diagnosis; therefore, categories are not mutually exclusive and percentages may sum to more than 100%. Abbreviations: 6MWT, six-minute walk test; CRP, C-reactive protein; eGFR, estimated glomerular filtration rate (CKD-EPI 2021 without race); HFA-PEFF, Heart Failure Association Pre-test assessment, Echocardiography and natriuretic peptide, Functional testing, and Final aetiology; LVEF, left ventricular ejection fraction; MCV, mean corpuscular volume; mTOR, mammalian target of rapamycin; NT-proBNP, N-terminal pro-B-type natriuretic peptide; PTH, parathyroid hormone; TSAT, transferrin saturation.

In a post-hoc analysis, participants achieved 83 ± 14% of their individually predicted 6MWT distance at baseline (median observed distance 512 m versus predicted 618 m), indicating reduced exercise capacity despite stable graft function. Of the 75 participants in the efficacy analysis population assigned to ferric carboxymaltose, 73 (97%) received three or four doses of ferric carboxymaltose (cumulative dose ≥1500 mg): 45 (60%) received four doses, 28 (37%) received three doses, one participant (1%) received two doses, and one (1%) received only one dose. Of the 73 participants in the efficacy analysis population assigned to placebo, 50 (69%) received four doses of placebo while 22 (30%) received three doses and one participant (1%) received only one dose. Reasons for these protocol deviations are described in Supplementary Table S4.

**Fig. 2:**
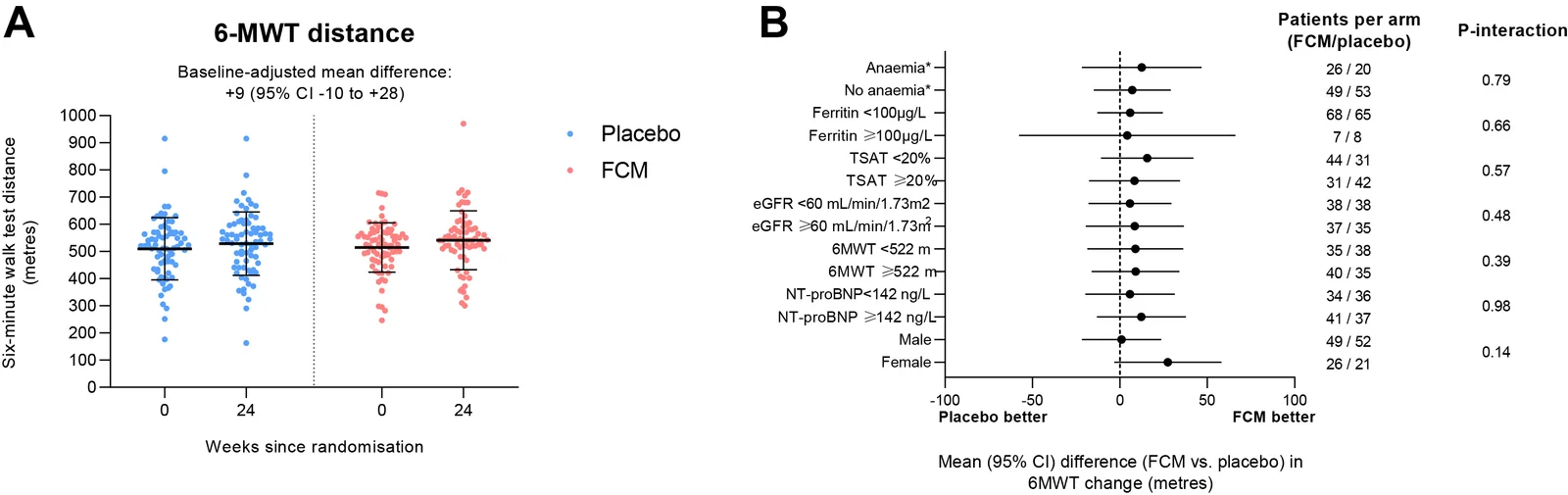
Six-minute walk test distance (full analysis set). **A.** Six-minute walk test distance in the ferric carboxymaltose (pink) and placebo (blue) groups at baseline and week 24. Depicted are jittered dot plots displaying individual observed values in both treatment groups and the lines represent the mean and standard deviation. The baseline-adjusted mean difference with 95% confidence interval was estimated using a baseline-adjusted linear regression model including treatment centre and baseline 6-min walk test distance as covariates. **B.** Change in 6-min walk test distance in prespecified and post-hoc subgroups according to assigned study treatment. Treatment effects with 95% confidence intervals are shown. p-values for interaction were derived from a two-way ANOVA model including treatment arm, subgroup, and the treatment-by-subgroup interaction term, and indicate whether the effect of FCM versus placebo on change in 6-min walk test distance differed between subgroups. Abbreviations: 6MWT, six-minute walk test; eGFR, estimated glomerular filtration rate (CKD-EPI 2021 without race); FCM, ferric carboxymaltose; NT-proBNP, N-terminal pro-B-type natriuretic peptide; TSAT, transferrin saturation. ∗Anaemia was defined as haemoglobin <13 g/dL in men and <12 g/dL in women.

At baseline, 6MWT distance was 515 ± 91 m in the ferric carboxymaltose arm and 510 ± 114 m in the placebo arm (Supplementary Table S5.1). After 24 weeks, treatment with ferric carboxymaltose did not improve 6MWT distance compared with placebo (change from baseline +28 ± 59 m versus +19 ± 58 m; baseline-adjusted mean difference +9 m [95% CI −10 to +28]; p = 0.37; Fig. 2a). Ferric carboxymaltose did not improve 6MWT distance beyond placebo in any of the prespecified subgroups (Fig. 2b). In the per *protocol* dataset, 6MWT distance increased by 24 ± 50 m in the ferric carboxymaltose group, compared to 14 ± 51 m in the placebo arm (n = 122, baseline-adjusted mean difference +10 m [95% CI −8 to +27]; p = 0.29). Thus, ferric carboxymaltose did not improve exercise capacity compared with placebo over 24 weeks.

**Fig. 3:**
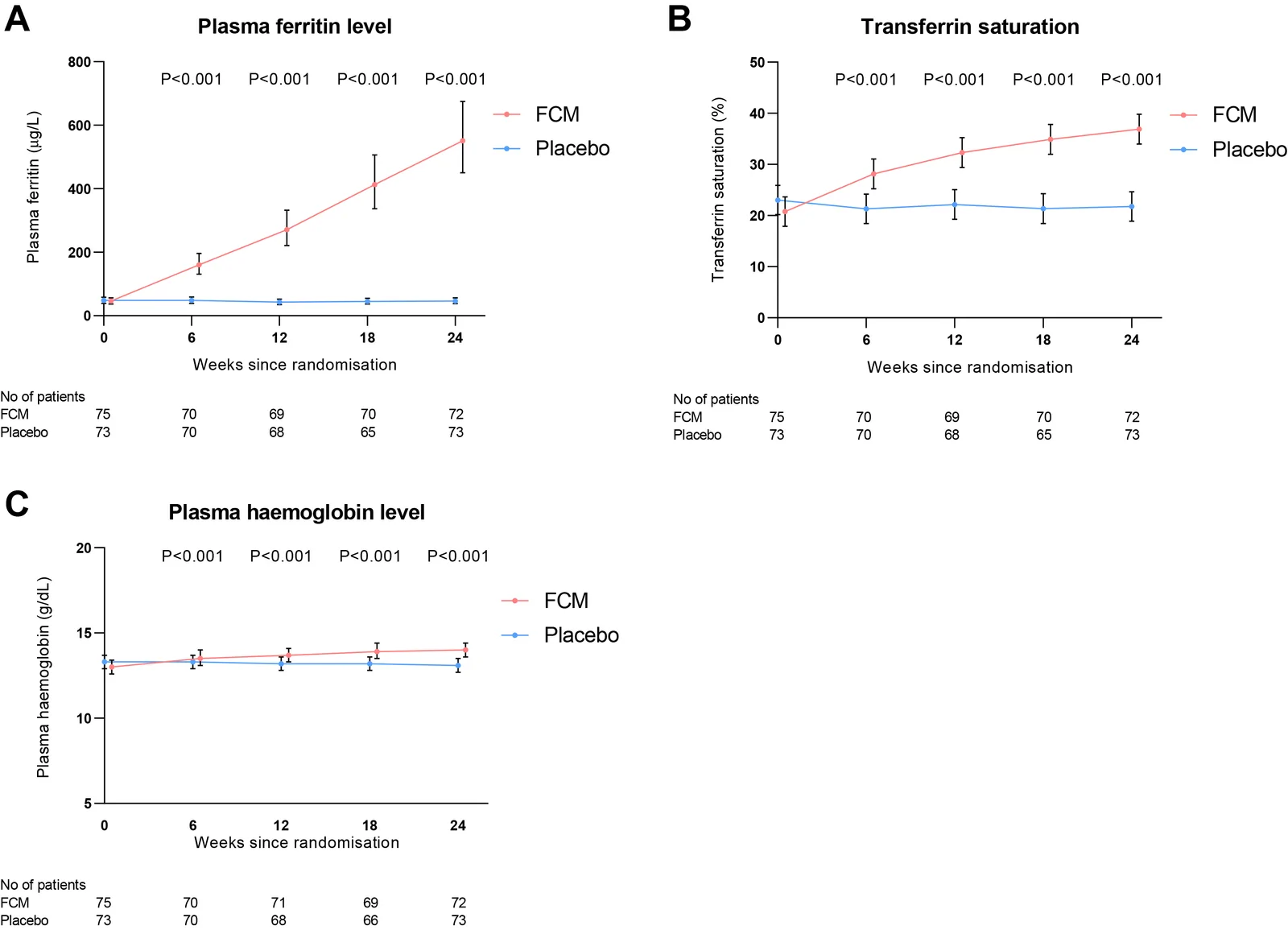
Iron status and haemoglobin (full analysis set). **A**. Plasma ferritin concentration. **B**. Transferrin saturation. **C**. Plasma haemoglobin concentration. Depicted are estimated marginal means with 95% confidence intervals at each study visit for the placebo (blue) and ferric carboxymaltose (pink) groups. Treatment effects were analysed using linear mixed-effects models including treatment group, time point, treatment-by-time interaction, and treatment centre, based jointly on all observed timepoints. p values represent between-group comparisons at each follow-up visit estimated from the treatment-by-time interaction in the linear mixed-effects model. Abbreviation: FCM, ferric carboxymaltose.

Ferric carboxymaltose markedly increased plasma ferritin concentrations compared with placebo (adjusted geometric mean ratio 12.5 [95% CI 10.5–14.8]; p < 0.001; Supplementary Table S5.2; Fig. 3a). Ferric carboxymaltose increased TSAT compared with placebo (adjusted mean difference [95% CI], +17.4% [+14.1 to +20.7], p < 0.001, Fig. 3b). Plasma iron, MCV and haematocrit showed similar between-group differences. Except for one participant, who withdrew consent for receiving study treatment, none of the participants in the ferric carboxymaltose group still met the criteria for iron deficiency after 24 weeks. Ferric carboxymaltose increased haemoglobin concentrations compared with placebo (adjusted mean difference [95% CI], +1.1 g/dL [+0.8 to +1.5], p < 0.001, Fig. 3c).

**Fig. 4:**
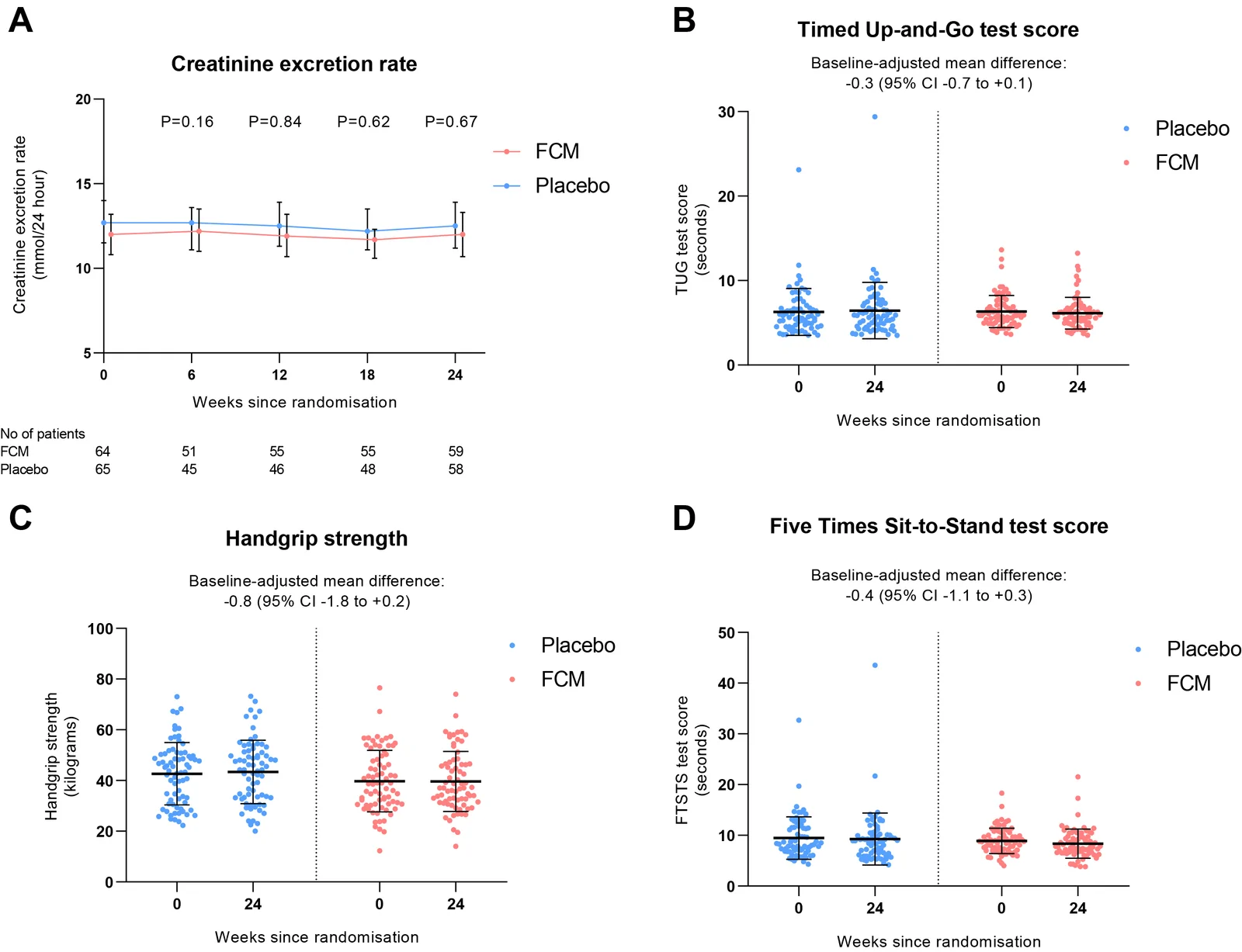
Skeletal muscle mass and function during follow-up according to assigned study treatment. **A**. Twenty-four-hour urinary creatinine excretion. Depicted are estimated marginal means with 95% confidence intervals at each study visit for the placebo (blue) and ferric carboxymaltose (pink) groups. Treatment effects were analysed using a linear mixed-effects model including treatment group, time point, treatment-by-time interaction, and treatment centre, based jointly on all observed timepoints. p values represent between-group comparisons at each follow-up visit estimated from the treatment-by-time interaction in the linear mixed-effects model. **B**. Timed Up-and-Go test performance. **C**. Handgrip strength. **D**. Five Times Sit-to-Stand test performance. For panels B–D, jittered dot plots show individual observed values at baseline and week 24; horizontal lines and error bars represent the mean and standard deviation. For each outcome, the baseline-adjusted mean difference with 95% confidence interval was estimated using a linear regression model including treatment centre and the baseline value of the respective outcome as covariates. Abbreviations: FCM, ferric carboxymaltose; FTSTS, Five Times Sit-to-Stand; TUG, Timed Up-and-Go.

Changes in the 24-h urinary creatinine excretion (reflecting muscle mass), Timed Up-and-Go test performance, handgrip strength and Five Times Sit-to-Stand test score were not different between treatment arms (Supplementary Table S5.3, Fig. 4a–d).

**Fig. 5:**
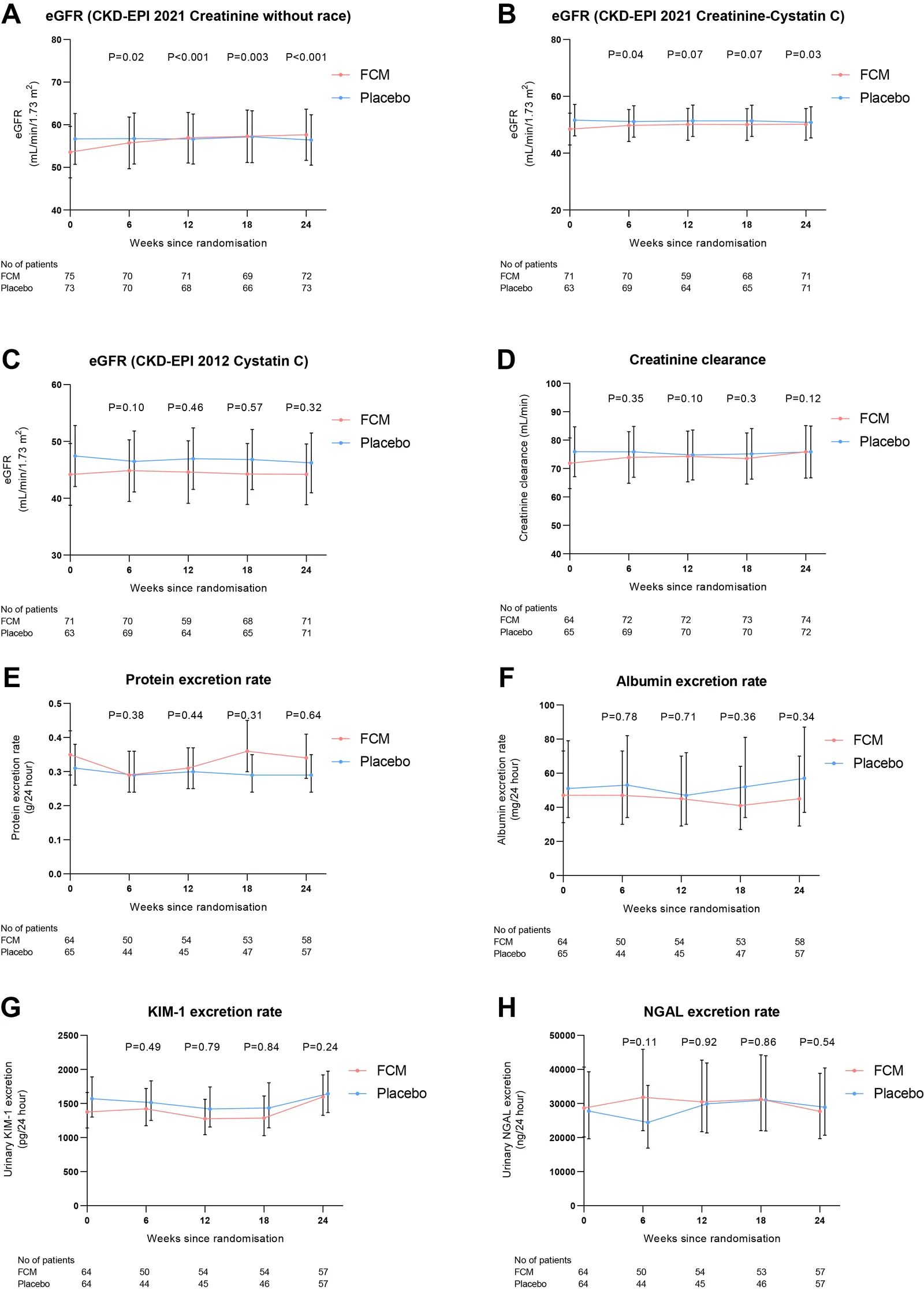
Kidney function (full analysis set). **A**. Estimated glomerular filtration rate using the CKD-EPI creatinine 2021 equation without race. **B**. Estimated glomerular filtration rate using the CKD-EPI creatinine-cystatin C 2021 equation. **C**. Estimated glomerular filtration rate using the CKD-EPI cystatin C 2012 equation. **D**. Twenty-four-hour urinary creatinine clearance. **E**. Twenty-four-hour urinary protein excretion rate. **F**. Twenty-four-hour urinary albumin excretion rate. **G**. Twenty-four-hour urinary kidney injury molecule-1 (KIM-1) excretion rate. **H**. Twenty-four-hour urinary neutrophil gelatinase-associated lipocalin excretion rate. Depicted are estimated marginal mean values with 95% confidence intervals at each study visit for the placebo (blue) and ferric carboxymaltose (pink) groups. Treatment effects were analysed using linear mixed-effects models based on all observed timepoints jointly. p values represent between-group comparisons at each follow-up visit estimated from the treatment-by-time interaction in the linear mixed-effects model. Abbreviations: eGFR, estimated glomerular filtration rate; FCM, ferric carboxymaltose; KIM-1, kidney injury molecule-1; NGAL, neutrophil gelatinase-associated lipocalin.

Using the 2021 creatinine-based CKD-EPI equation, eGFR was higher over 24 weeks in the ferric carboxymaltose group than in the placebo group (adjusted mean difference [95% CI], +4.3 [+2.1 to +6.5] mL/min/1.73 m^2^; p < 0.001, Supplementary Table S5.4, Fig. 5a). A similar between-group difference was observed when the eGFR was estimated using the CKD-EPI creatinine-cystatin C 2021 equation (adjusted mean difference [95% CI], +2.5 [+0.3 to +4.6] mL/min/1.73 m^2^; p = 0.03, Fig. 5b). When the CKD-EPI cystatin C 2012 calculation was used, eGFR remained stable in the ferric carboxymaltose group and decreased in the placebo group (adjusted mean difference [95% CI], +1.2 [−1.2 to +3.6] mL/min/1.73 m^2^; p = 0.32, Fig. 5c). The 24-h creatinine clearance was numerically higher in the ferric carboxymaltose group compared with the placebo group (adjusted mean difference [95% CI], +4.1 [−1.1 to +9.2] mL/min; p = 0.12, Fig. 5d). No between-group differences were observed in changes in proteinuria, albuminuria or urinary excretion of markers of tubular injury KIM-1 or NGAL (Fig. 5e–h).

**Fig. 6:**
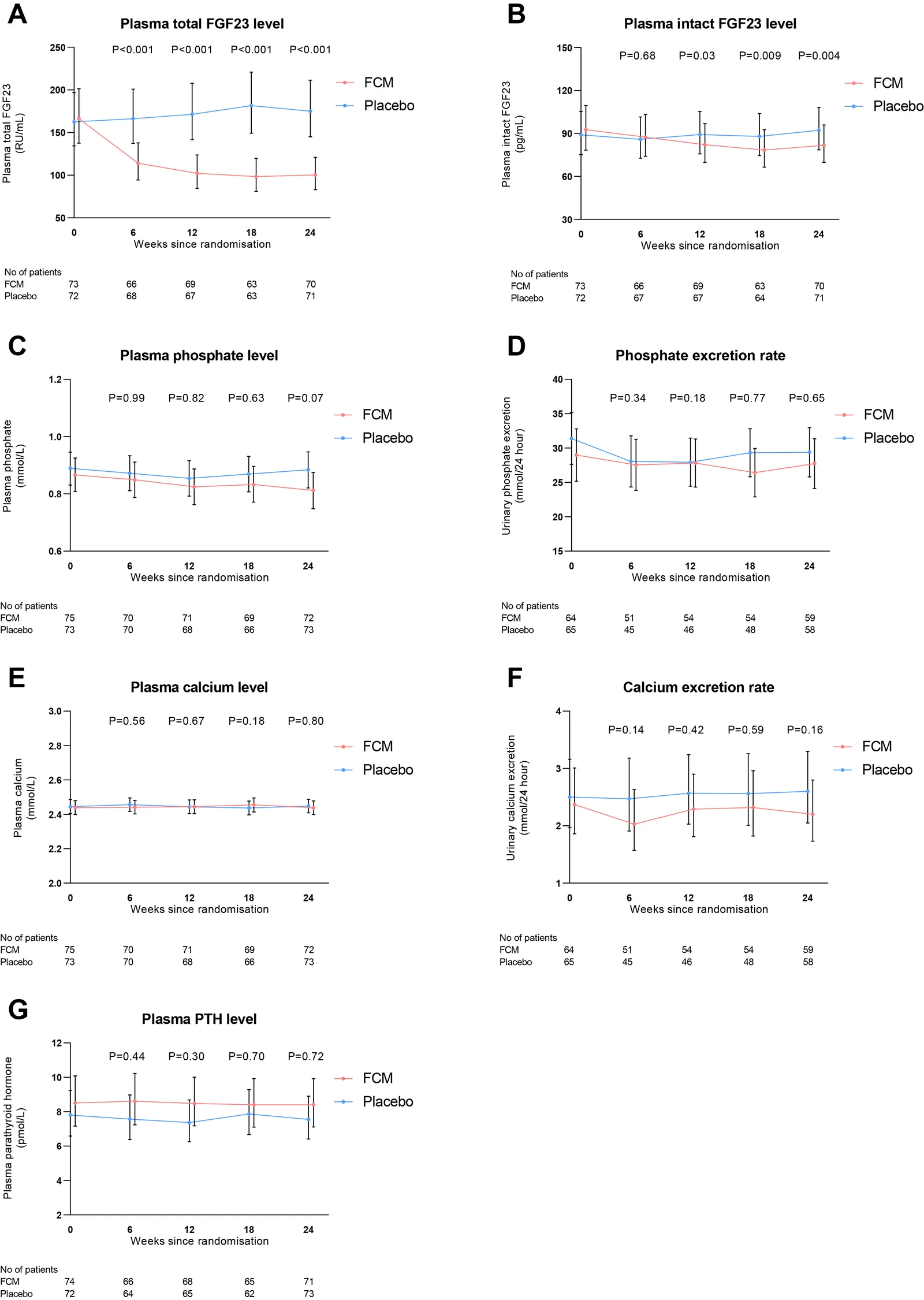
Mineral homoeostasis (full analysis set). **A**. Plasma total fibroblast growth factor 23 concentration. **B**. Plasma intact fibroblast growth factor 23 concentration. **C**. Plasma phosphate concentration. **D**. Twenty-four-hour urinary phosphate excretion rate. **E**. Plasma calcium concentration. **F**. Twenty-four-hour urinary calcium excretion rate. **G**. Plasma parathyroid hormone concentration. Depicted are estimated marginal mean values with 95% confidence intervals at each study visit for the placebo (blue) and ferric carboxymaltose (pink) groups. Treatment effects were analysed using linear mixed-effects models based on all observed timepoints jointly. p values represent between-group comparisons at each follow-up visit estimated from the treatment-by-time interaction in the linear mixed-effects model. Abbreviations: FCM, ferric carboxymaltose; FGF23, fibroblast growth factor 23; PTH, parathyroid hormone.

Over the 24-week study period, total plasma FGF23 concentrations decreased in the ferric carboxymaltose group but increased in the placebo group (adjusted geometric mean ratio [95% CI], 0.56 [0.48–0.65], p < 0.001, Supplementary Table S5.5, Fig. 6a). Intact FGF23 concentrations also differed between the groups, with lower values over time in the ferric carboxymaltose group compared with the placebo group (adjusted geometric mean ratio [95% CI], 0.85 [0.77–0.95], p = 0.004, Fig. 6b). Plasma phosphate levels were slightly and not significantly lower over time in the ferric carboxymaltose group than in the placebo group (adjusted mean difference [95% CI], −0.05 [−0.1 to +0.0] mmol/L, p = 0.07, Fig. 6c), whereas no between-group difference was observed in 24-h urinary phosphate excretion (Fig. 6d). Plasma calcium and PTH levels and urinary calcium excretion did not differ between groups during follow-up (Fig. 6e–g).

Echocardiographic parameters of ventricular wall thickness, specifically interventricular septum thickness, left ventricular end-diastolic thickness, left posterior wall thickness and relative wall thickness did not differ between the groups during follow-up (Supplementary Table S5.6). Left ventricular mass and volume indices were also similar between both groups.

No between-group differences were observed in left ventricular ejection fraction, tricuspid annular plane systolic excursion, systolic tissue velocity of the tricuspid valve annulus and early diastolic lateral and septal tissue velocities. Pressure gradients, E/A-ratio and E/e’-ratio, were also similar between the groups.

Changes in global longitudinal strain, atrial strain, plasma troponin or HFA-PEFF score did not differ between the groups. Plasma NT-proBNP significantly increased in the ferric carboxymaltose group compared to the placebo group (adjusted geometric mean ratio [95% CI], 1.24 [1.04–1.49], p = 0.02). There was a decrease in systolic blood pressure in the ferric carboxymaltose group compared to the placebo group (adjusted mean difference [95% CI], −7.1 [−12.9 to −1.2] mmHg, p = 0.02). Urinary sodium concentration or fractional sodium excretion, analysed post-hoc, did not differ between groups during follow-up (Supplementary Table S5.7).

At baseline, the median standardised percentile memory test score of the participants was 44 (35–56), the median standardised percentile score for attention and mental speed was 56 (36–69) and the median standardised percentile score for executive functioning was 43 (25–61). Three participants (2%) scored below the tenth percentile on memory, none on attention and mental speed test and eight (7%) on executive functioning. Changes in any of the individual parameters of cognitive function were not different between treatment arms (Supplementary Table S5.8).

Scores on health-related quality of life and fatigue questionnaires, including the SF36, CIS, DMFS and EuroQol-5D-5L, did not differ between groups during follow-up, and no consistent trends favouring ferric carboxymaltose were observed across patient-reported outcome domains (Supplementary Table S5.9). Restless legs symptoms did not differ between groups during follow-up.

The incidence of gastrointestinal symptoms, assessed using the GSRS, did not differ between the ferric carboxymaltose and placebo groups (Supplementary Table S6.1). Safety laboratory parameters, including liver enzymes, were comparable between groups (Supplementary Table S6.2). Adverse events occurring during or within 24 h after study treatment, or during the subsequent six weeks, are listed in Supplementary Table S7. No anaphylactic reactions occurred in any of the 276 ferric carboxymaltose administrations.

The frequency of infections was similar between groups. Two deaths (2.6%) occurred in the ferric carboxymaltose group during the study period. In one participant, metastatic oesophageal carcinoma was diagnosed and resulted in death. In another participant, acute arterial occlusion of a peripheral artery and renal artery led to lower extremity amputation and subsequent death. Neither event was considered related to study treatment.

Mild hypophosphataemia (plasma phosphate 0.35–0.50 mmol/L) was observed during follow-up study visits in twelve participants (15.6%) in the ferric carboxymaltose group and in six participants (7.8%) in the placebo group. Severe hypophosphataemia (<0.35 mmol/L) occurred in one participant (1.3%) in the ferric carboxymaltose group and two participants (2.6%) in the placebo group.

## Discussion

In this multicentre, double-blind, randomised, placebo-controlled trial, correction of iron deficiency with intravenous ferric carboxymaltose did not improve exercise capacity in iron-deficient kidney transplant recipients. This null finding was consistent across all prespecified subgroups, including participants with more pronounced iron deficiency, anaemia, or lower baseline exercise capacity. The adjusted between-group difference in 6MWT distance was small, and the 95% confidence interval excluded a treatment benefit of the magnitude anticipated in the sample size calculation. Together, these findings indicate that effective biochemical correction of iron deficiency does not translate into meaningful improvements in functional capacity in this population.

In patients with chronic diseases, such as chronic heart failure, chronic kidney disease or in kidney transplant recipients, treatment of iron deficiency (anaemia) with intravenous iron is generally preferred over oral supplementation. Intravenous iron is often more effective than oral iron^51^, ^52^, ^53^ because chronic inflammation impairs intestinal iron absorption, while oral iron is frequently associated with gastrointestinal side effects and poor adherence.^54^ However, evidence to support the management of iron deficiency with or without anaemia in kidney transplant recipients has remained limited, and recommendations have largely been extrapolated from non-transplant populations.^55^^,^^56^ The present trial helps address this evidence gap.

We hypothesised that iron deficiency correction would improve exercise capacity through different mechanisms. *First*, increased haemoglobin concentrations could enhance oxygen transport, particularly in patients with anaemia. Although haemoglobin increased following ferric carboxymaltose administration, this did not translate into improved exercise capacity, even among participants with anaemia. *Second*, iron repletion was expected to enhance skeletal muscle energy metabolism and function. Iron is crucial for myoglobin-mediated oxygen storage and mitochondrial oxidative metabolism,^57^ and experimental data suggest that iron deficiency impairs skeletal muscle growth and aerobic capacity.^58^ In the present study, no consistent effects were seen on muscle mass, Timed Up-and-Go performance, handgrip strength or five times sit-to-stand test performance, nor did these changes translate into a better overall exercise capacity. *Third*, we hypothesised that iron deficiency correction would enhance mitochondrial function in cardiomyocytes, improving myocardial contractility.^59^ Iron deficiency has been associated with impaired exercise tolerance in patients with heart failure with both reduced (HFrEF)^9^^,^^11^^,^^12^ and preserved (HFpEF)^10^ ejection fraction. In iron-deficient patients with chronic heart failure, intravenous iron improved ventricular systolic function^60^^,^^61^ as well as exercise capacity^19^, ^20^, ^21^, ^22^ and reduced heart failure hospitalisations.^62^^,^^63^ HFpEF is the dominant heart failure phenotype in kidney transplant recipients^64^ and diastolic function does usually not improve or may even deteriorate after transplantation.^65^ While 81 participants showed signs of diastolic stress (HFA-PEFF 2–4) and 21 met the diagnostic criteria for HFpEF (HFA-PEFF 5–6), ferric carboxymaltose did not improve parameters of systolic or diastolic myocardial function, nor echocardiographic parameters indicative of HFpEF.

Several factors may explain the absence of a consistent beneficial effect of iron deficiency correction on functional outcomes. Although participants demonstrated better exercise capacity than reported in a previous transplant study,^1^ baseline 6MWT distances remained substantially below predicted values^49^ and those of healthy individuals.^66^ Moreover, increases in 6MWT distance were observed in both treatment groups, possibly reflecting a Hawthorne effect, indicating that further improvement in exercise capacity was feasible. In addition, no treatment effect was observed in subgroups with lower baseline exercise capacity, higher NT-proBNP concentrations, or higher HFA-PEFF scores, suggesting that limited baseline impairment alone does not explain the null findings. Other factors inherent to the transplant population, such as corticosteroid use, chronic immune activation and multimorbidity, may attenuate potential benefits of iron repletion. Finally, the optimal definition of iron deficiency in kidney transplant recipients is unclear.^6^ However, even when analyses were restricted to participants with a baseline TSAT <20%, no evidence of benefit was observed.

Ferric carboxymaltose administration was associated with lower circulating FGF23 levels and a small reduction in plasma phosphate levels. Although these findings are biologically consistent with known interactions between iron metabolism, FGF23 regulation^67^^,^^68^ and left ventricular mass,^26^ the clinical relevance of these changes remains uncertain. No effects were observed on ventricular mass or wall thickness, and the study was not designed to assess long-term skeletal or cardiovascular outcomes. The modest reduction in phosphate concentrations and higher incidence of mild, asymptomatic hypophosphataemia were not associated with clinically relevant adverse events, but longer follow-up would be required to assess potential downstream effects.

Despite strong observational associations between iron deficiency and impaired cognitive function,^7^ fatigue, and reduced quality of life after kidney transplantation,^8^ correction of iron deficiency did not improve cognitive performance, fatigue, or health-related quality of life in this trial. Although the study was not powered to detect small treatment effects on cognitive or patient-reported outcomes, we did not observe consistent non-significant trends favouring ferric carboxymaltose across these domains. These findings suggest that iron deficiency may act as a marker of overall disease burden rather than a modifiable driver of neurocognitive or patient-reported outcomes in this population.

Ferric carboxymaltose treatment was associated with a modest increase in eGFR. This finding should be interpreted cautiously. The consistent observation among creatinine- and cystatin C-based eGFR methods and the absence of changes in muscle mass argue against artefactual explanations, but the mechanisms underlying this observation remain uncertain. Given the short follow-up and exploratory nature of this secondary outcome, no causal inferences can be drawn, and confirmation in dedicated studies is warranted.

From a clinical perspective, these findings suggest that routine use of intravenous iron to improve functional capacity in stable kidney transplant recipients without anaemia is unlikely to be beneficial. Although biochemical correction of iron deficiency was robust, this did not translate into improvements in exercise capacity or other patient-centred outcomes. These results argue against extrapolating benefits of intravenous iron observed in heart failure or chronic kidney disease populations to kidney transplant recipients and highlight the need for more targeted approaches to address impaired physical functioning after transplantation.

Limitations of this trial include its intermediate sample size and relatively short follow-up, which precluded assessment of long-term outcomes. Data analysts were not blinded to treatment allocation, which could have introduced analytical bias; however, the primary analysis followed a prespecified statistical analysis plan. Although the study population likely represented a relatively stable subset of kidney transplant recipients, reduced exercise capacity and functional impairment were common at baseline, underscoring the clinical relevance of the primary outcome. The study population was predominantly White, which limited the ability to assess differences by race or ethnicity and may limit generalisability to more diverse populations and healthcare settings. Finally, women were slightly underrepresented in the study. Pregnancy or disagreement to take adequate contraceptive precautions was an exclusion criterion, because guidelines currently recommend against prescribing ferric carboxymaltose during the first trimester of pregnancy. Although women taking adequate contraceptive precautions were allowed to participate, this exclusion criterion may have reduced the willingness of women of reproductive age to participate. Strengths include the rigorous design, clinically meaningful endpoints, and low drop-out rate.

In conclusion, intravenous ferric carboxymaltose did not improve exercise capacity or most secondary clinical outcomes in stable iron-deficient kidney transplant recipients over 24 weeks. Although exploratory differences were observed for eGFR and FGF23 concentrations, these findings require confirmation in future studies. Whether intravenous iron supplementation may benefit patients with more severe iron deficiency, advanced graft dysfunction, or overt heart failure remains to be determined.

## Contributors

MHdB, MFE, CGMG, and JSJV were involved in conceptualisation. SJLB, SPB, MHdB, MFE, IMN, JSFS, JMS, JMtM, PvdM, ADvZ, JSJV, and ALZ were involved in methodology. IMN and JSJV were involved in formal analysis. HWGMD, JEKR, AvdV, CJvL, and JSJV were involved in data collection. MHdB and JSJV wrote the original draft. All authors were involved in reviewing and editing the original draft. MHdB supervised all research activities. MHdB, MFE, and JSJV were involved in funding acquisition. SJLB, SPB, MHdB, MFCdJ, JSFS, and ADvZ were involved in the identification of participants. MHdB and JSJV accessed and verified the underlying data and were responsible for the decision to submit the manuscript.

## Data sharing statement

Deidentified participant data can be made available to individual researchers upon reasonable request to the corresponding author. The institutional review board of the UMCG (project number METc 2018/482) precludes publication of participant-level data due to privacy concerns. The statistical analysis syntax used for this manuscript is provided as Supplementary Material.

## Declaration of interests

MHdB has received grant support from CSL Vifor and declares consulting fees from Astellas, AstraZeneca, Bayer, Boehringer Ingelheim, CSL Vifor, Kyowa Kirin Pharma, Lilly and Sanofi Genzyme (all to institution). MFCdJ received grant support from the Dutch Kidney Foundation, Astellas and Chiesi. MFE has received speaker/consultancy fees from Vifor Pharma, Astellas, Cablon Medical, Medice, and Pharmacosmos; has served on the Advisory Board for Cablon Medical, GlaxoSmithKline, and Medice; and has received grant support from Cablon Medical and Astellas (all to employer in all instances). CGMG received consulting fees and speaking fees from GlaxoSmithKline, Vifor Pharma and Medice. JMtM is supported by a Veni grant from ZonMW and declares speaker fees to institution from Bayer, Boehringer Ingelheim, Moderna, Novartis, Novo Nordisk, Roche, and Johnson and Johnson, and served on the data safety monitoring board for FASTR. PvdM received research grants of Novartis, Vifor Pharma, Pharmacosmos, Ionis, AstraZeneca, Pharma Nord, Novo Nordisk, Pfizer and received research consulting fees of Novartis, Vifor Pharma, Pharmacosmos, Ionis, AstraZeneca, Pharma Nord, Novo Nordisk, Boehringer Ingelheim, Bayer, Pfizer (all to institution). All other authors declare no competing interests.

## Appendix A Supplementary data

Study Protocol

Statistical Analysis Plan

Supplementary Material

Translated abstract in Dutch
