# Supplementary material for: Intravenous ferric carboxymaltose and exercise capacity in iron-deficient kidney transplant recipients: a randomised, placebo-controlled trial in the Netherlands
Source: eClinicalMedicine. 2026 Sep 1;99:104169. doi: 10.1016/j.eclinm.2026.104169 (PMC13571158; doi:10.1016/j.eclinm.2026.104169)
Supplement: Supplementary file 2 [file mmc2.pdf]

	Scientific Research		
	**Document type**	Statistical Analysis Plan	
	**Number and title**	Statistical Analysis Plan EFFECT-KTx study	Version 01

EFFECT OF FERRIC CARBOXYMALTOSE ON EXERCISE CAPACITY AFTER KIDNEY TRANSPLANTATION: A MULTICENTER RANDOMIZED CONTROLLED TRIAL

EFFECT-KTx

UMCG Research Register Number 201800450

STATISTICAL ANALYSIS PLAN

Version: 2.0*

Date of document: September 3, 2024

**Completed by**				
Full name	Role in study	Department	Date	Signature
Joanna Sophia J Vinke	Study Coordinator	Nephrology	3-9-2024	Anna-Sophie Vinke

**Authorized by**				
Full name	Role in study	Department	Date	Signature
Professor Martin H de Borst	Principal Investigator	Nephrology	3-9-2024	

1. TABLE OF CONTENTS

1. TABLE OF CONTENTS 2
2. INTRODUCTION 3
3. PROTOCOL SUMMARY 3
3.1 Study rationale and design 3
3.2 Study flow chart 5
4. STUDY OBJECTIVE AND OUTCOME / RESPONSE MEASURES 5
4.1 Study outcome - general considerations 6
4.2 Primary outcome parameters 6
4.3 Secondary outcome parameters 6
4.4 Safety outcome parameters 6
4.5 Exploratory outcome parameters 8
5. STATISTICAL METHODS AND REPORTING 9
5.1 Sample size 9
5.2 Randomization, database lock and unblinding 9
5.3 Protocol violations 10
5.4 Study analysis sets 10
5.4.1. Full analysis set 11
5.4.2. Per protocol set 11
5.4.3. Safety analysis set 12
5.5 Study participants characteristics 12
5.6 Efficacy outcome 13
5.6.1 Primary efficacy outcome 13
5.6.2 Secondary efficacy outcomes 13
5.6.3. Exploratory efficacy outcomes 13
5.7 Safety outcome 14
5.8 Statistical methods used for efficacy and safety 14
6. PRESENTATION OF RESULTS 16
7. CHANGES FROM PROTOCOL AND REMARKS 16
8. REFERENCES 20

2. INTRODUCTION

The purpose of the statistical analysis plan (SAP) is to provide a comprehensive and detailed description of the rationale, methods and reporting of the data analysis for the EFFECT-KTx study.

3. PROTOCOL SUMMARY

3.1 Study rationale and design

Rationale and hypothesis

After kidney transplantation, exercise capacity and skeletal muscle strength improve compared with the dialysis setting, but this improvement is often incomplete. Reduced physical activity and muscle strength have been associated with an excess risk of mortality and worse graft outcomes in kidney transplant recipients (KTRs). Therefore, it is key to identify new modifiable risk factors related to impaired exercise tolerance.

Iron deficiency (ID) is common after kidney transplantation, with reported prevalences ranging between 11% and 47%, depending on the definition and time after transplantation. ID has been associated with an increased risk of mortality in KTRs, independent of coexisting anaemia. Iron is involved in oxygen transport as a component of haemoglobin. Moreover, ID has implications beyond impaired erythropoiesis as iron is crucial to myriad physiological processes. Iron is a cofactor of many enzymes involved in different cellular functions: as a constituent of aconitase and succinate dehydrogenase, both enzymes involved in the Krebs cycle, and as a constituent of different complexes involved in the mitochondrial respiratory chain, iron is pivotal to cellular energy metabolism. Additionally, iron is involved in nucleotide synthesis for replication and production of DNA and RNA. Therefore, ID may affect growth and function of many cell types, in particular those with high energy consumption or mitogenic activity such as skeletal muscle cells, cardiomyocytes, lymphocytes and neurons.

The impact of ID and its correction on muscle strength and physical function in KTRs is yet unknown. The Effect of Ferric Carboxymaltose on Exercise Capacity after Kidney Transplantation (EFFECT-KTx) trial has been designed to test the hypothesis that iron supplementation improves exercise capacity, skeletal muscle strength, cardiac function and quality of life in iron-deficient KTRs. Furthermore, the EFFECT-KTx study allows for testing of

the hypothesis that iron supplementation may improve neurocognitive function and lymphocyte growth and function. For a more detailed version of the rationale of the EFFECT-KTx study, we refer to the previously published protocol paper.[1]

Design

The EFFECT-KTx study is designed as an investigator-initiated, multicenter, randomised placebo-controlled double-blinded clinical superiority trial with two parallel treatment arms. Study medication (ferric carboxymaltose or placebo, allocation ratio 1:1) is administered at a baseline visit and at subsequent study visits 6, 12 and 18 weeks later. Study participants were screened and recruited at the University Medical Centre Groningen (UMCG) and the University Medical Centre Utrecht (UMCU), both in the Netherlands. KTRs with ID, defined as plasma ferritin <100 µg/L or plasma ferritin 100–299 µg/L with transferrin saturation (TSAT) <20%, and a haemoglobin level of ≥10.5 g/dL, who were at least 6 months after transplantation, were eligible for participation. A complete list of inclusion and exclusion criteria can be found in the protocol paper and in table 1.[1] In total, 148 participants were included.

**Inclusion criteria**	**Exclusion criteria**
Kidney transplant recipient	Known intolerance to intravenous iron products
Iron deficiency at two consecutive measurements with an interval of at least a week, defined as plasma ferritin<100 µg/L or plasma ferritin 100–299 µg/L with transferrin saturation <20%	Severe (Hb<10.5 g/dL) or progressive anaemia (Hb decline of ≥3.2 g/dL in 2 months) or a reduced mean corpuscular erythrocyte volume (<80 fL)
≥6 months after kidney transplantation	Recent history of gastrointestinal or urogenital blood loss or a positive faecal occult blood test
≥18 years of age	Erythrocyte transfusion ≤6 weeks prior to inclusion
Ability to comply with the study protocol	Polycythaemia (Hb>15.3 g/dL) or a known history of haemochromatosis
Informed consent	Estimated glomerular filtration rate of ≤30 mL/min per 1.73 m^2
	Symptomatic coronary artery disease or myocardial infarction ≤4 weeks prior to inclusion
	Disability to walk
	Severe hypophosphatemia (plasma phosphate <0.35 mmol/L) ≤4 weeks prior to inclusion
	For women at childbearing age: pregnancy or disagreement to take adequate contraceptive

	precautions
	Signs of an active systemic infection
	Participation in another intervention study

Table 1. Inclusion and exclusion criteria

3.2 Study flow chart

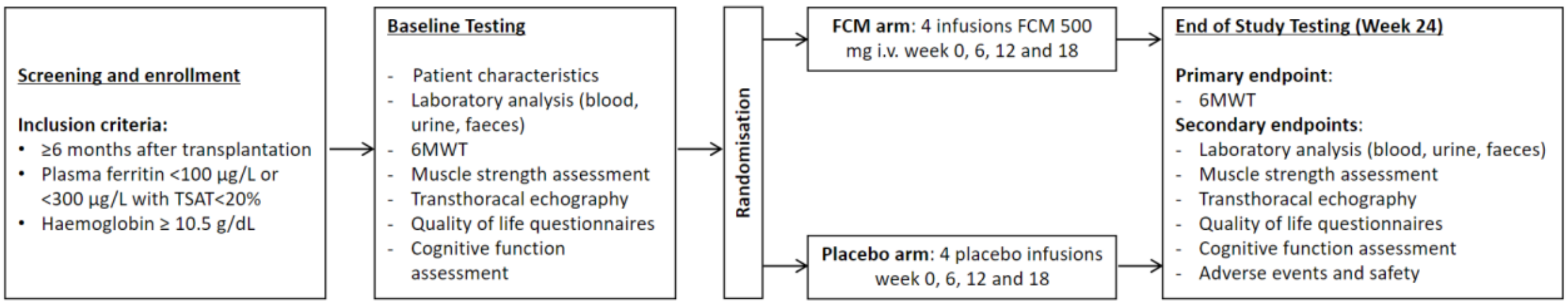

Figure 1. EFFECT-KTx study flowchart

4. STUDY OBJECTIVE AND OUTCOME / RESPONSE MEASURES

The primary outcome of the study is change in exercise capacity, which is measured using the 6 minute walk test (6MWT) from baseline to week 24. The mode of conduction of this test is described in the protocol paper.[1]

Secondary objectives include change in haematinic parameters, change in skeletal muscle strength and functional mobility (measured using the Five Times Sit-to-Stand (FTSTS) Test, the Timed Up-and-Go (TUG) Test and a handgrip dynamometry) and estimated muscle mass (using 24-hour urinary creatinine excretion rate).

Exploratory outcomes include myocardial morphology and function (measured using transthoracal echography parameters, troponin T and N-terminal pro-B-type natriuretic peptide), quality of life (using the Short Form-36 Questionnaire, the Dutch 'Checklist Individuele Spankrant', the Dutch Multifactor Fatigue Scale and the EuroQol-5D-5L Questionnaire), change in parameters of bone and mineral metabolism and a number of neurocognitive outcomes (using an extensive set of neuropsychological tests). Furthermore, changes in the incidence of restless legs related symptoms, urine markers of kidney graft rejection and injury, proliferation and function of cells of the immune system and effects of iron supplementation on the gut microbiota will be assessed as exploratory outcomes.

Safety parameters are collected and include the incidence of adverse clinical events, hypophosphataemia, hepatic injury and changes in kidney function.

4.1 Study outcome - general considerations

A concise description of the mode of collection of all relevant data for the primary and secondary outcomes can be found in the protocol paper.[1]

4.2 Primary outcome parameter

Change in number of meters walked during the 6MWT from baseline to week 24.

4.3 Secondary outcome parameters

In the table below, the secondary outcome parameters are listed.

Secondary outcome		Base-line	Week 6	Week 12	Week 18	Week 24
**Haematinic indices and iron status**						
2a	Change in plasma haemoglobin between consecutive study visits	x	x	x	x	x
2b	Change in plasma ferritin between consecutive study visits	X	x	x	x	x
2c	Change in plasma transferrin saturation between consecutive study visits	X	x	x	x	X
2d	Change in plasma soluble transferrin receptor between consecutive study visits	x		x		x
2e	Change in plasma hepcidin between consecutive study visits	x	x	x	x	x
**Muscle mass and strength and functional mobility**						
3a	Change in isometric handgrip strength between baseline and week 24	X				x
3b	Change in number of seconds to complete the Five Times Sit-to-Stand (FTSTS) Test between baseline and week 24	X				x
3c	Change in number of seconds to complete the Timed Up-and-Go (TUG) Test between baseline and week 24	X				x
3d	Change in 24-hour urinary creatinine excretion rate between consecutive study visits	x	x	x	x	x

Table 2. Secondary outcomes

4.4 Exploratory outcome parameters

In the table below, the exploratory outcome parameters are listed.

Exploratory outcome		Base-line	Week 6	Week 12	Week 18	Week 24
**Quality of life**						
4a	Change in Short-Form-36 (SF36) score between baseline and week 24	X				X
4b	Change in dutch 'Checklist Individuele Spankrant' (CIS) score	X				X

	between baseline and week 24					
4c	Change in dutch Multifactor Fatigue Scale (DMFS) score between baseline and week 24	X				X
4d	Change in EuroQol-5D-5L (EQ5D-5L) score between baseline and week 24	x				X
**Bone and mineral metabolism**						
5a	Change in plasma phosphate between consecutive study visits	X	x	x	x	X
5b	Change in 24-hour urinary phosphate excretion rate between consecutive study visits	X	x	x	x	X
5c	Change in plasma calcium between consecutive study visits	X	x	x	x	X
5d	Change in 24-hour urinary calcium excretion rate between consecutive study visits	x	x	x	x	X
5[e]	Change in plasma parathyroid hormone between consecutive study visits	x	x	x	x	x
5f	Change in plasma C-terminal fibroblast growth factor 23 (FGF23) between consecutive study visits	x	x	x	x	x
5g	Change in total plasma FGF23 between consecutive study visits	x	x	x	x	x
5h	Change in plasma 1.25-dihydroxycholecalciferol between consecutive study visits	x		x		x
5i	Change in plasma 25-hydroxycholecalciferol between consecutive study visits	x		x		x
**Cardiac structure, function and strain**						
6a	Change in interventricular septal thickness between baseline and week 24	X				X
6b	Change in left ventricular end diastolic diameter between baseline and week 24	X				X
6c	Change in left ventricular posterior wall diameter between baseline and week 24	X				X
6d	Change in left atrial volume indexed to body surface area between baseline and week 24	X				X
6e	Change in relative wall thickness between baseline and week 24	X				x
6f	Change in left ventricular mass index between baseline and week 24	X				x
6g	Change in left ventricular ejection fraction between baseline and week 24	x				X
6h	Change in mean early diastolic tissue velocity between baseline and week 24	x				X
6i	Change in E/A ratio between baseline and week 24	X				x
6j	Change in E/e' ratio between baseline and week 24	X				x
6k	Change in tricuspid annular plane systolic excursion between baseline and week 24	X				X
6l	Change in systolic tissue velocity of the tricuspid valve annulus between baseline and week 24	X				X
6m	Change in global longitudinal strain between baseline and week 24	X				X
6n	Change in atrial strain between baseline and week 24	X				X
6o	Change in plasma Troponin-T between consecutive study visits	X		x		X
6p	Change in plasma N-terminal pro-B-type natriuretic peptide between consecutive study visits	X		x		X
6q	Change in Heart Failure Association preserved ejection fraction score between baseline and week 24	x				X
**Cognitive function**						
7a	Change in combined memory score between baseline and week 24	X				X
7b	Change in combined attention and mental speed score between baseline and week 24	x				x
7c	Change in combined executive function score between baseline and week 24	x				x
7d	Change in Digit Span Forward score between baseline and	X				X

	week 24					
7[e]	Change in Word Fluency Test score between baseline and week 24	X				X
7f	Change in 15 Words Test immediate recall score between baseline and week 24	X				X
7g	Change in 15 Words Test delayed recall score between baseline and week 24	X				X
7h	Change in Symbol Digit Modalities Test score between baseline and week 24	X				X
7i	Change in Trail Making Test part A score between baseline and week 24	X				X
7j	Change in Trail Making Test part B score between baseline and week 24	X				X
7k	Change in Controlled Oral Word Association Test score between baseline and week 24	X				X
7l	Change in Digit Span Backward score between baseline and week 24	X				X
**Immune system (only in limited number of subjects)**						
8a	Change in lymphocyte iron content	X				x
8b	Change in lymphocyte ferritin content	x				x
8c	Change in CD4+ T-lymphocyte proliferation rate	X				x
8d	Change in CD8+ T-lymphocyte proliferation rate	X				x
8[e]	Change in B-lymphocyte proliferation rate	X				x
8f	Change in plasma cell formation rate	X				x
8g	Change in lymphocyte immunoglobulin G (IgG) production	X				x
8h	Change in lymphocyte interleukin-6 expression	X				x
8i	Change in lymphocyte interleukin10 expression	X				x
8j	Change in lymphocyte tumor necrosis factor expresion	x				x
**Gut microbiota**						
9	Change in variation of gut microbiota in a faeces sample between baseline and week 24	x				x
**Early graft rejection**						
10 a	Change in urine neutrophil gelatinase-associated lipocalin (NGAL) between consecutive study visits between consecutive study visits	X	x	X	x	x
10 b	Change in urine kidney injury marker 1 (KIM-1) between consecutive study visits between consecutive study visits	x	X	x	X	x
**Restless legs syndrome**						
11	Incidence of restless legs syndrome related symptoms	x	x	x	x	x

Table 3. Exploratory outcomes

Concerning the exploratory outcomes, we hold the freedom to modify the exact measurements to be performed on lymphocytes, urine and faeces when appropriate because of new scientific insights.

4.5 Safety outcome parameters

In the table below, the safety outcome parameters are listed.

Safety endpoint	Base-line	Week 6	Week 12	Week 18	Week 24
**Incidence of adverse clinical events**					
Incidence of infections		x	x	x	X
Incidence of injection site reactions		X	x	X	X

Incidence of post-infusion allergic reactions		x	X	x	X
Incidence of graft rejection		x	x	X	X
Incidence of serious adverse events		x	x	x	X
Incidence of significant hypophosphatemia (<0.50 mmol/L)		x	x	x	X
Incidence of severe hypophosphatemia (<0.35 mmol/L)		x	x	x	x
**Kidney injury**					
Change in estimated glomerular filtration rate between consecutive study visits	x	x	x	x	x
**Hepatic injury**					
Change in aspartate transaminase (AST) between consecutive study visits	X	x	X	x	x
Change in alanine transaminase (ALT) between consecutive study visits	x	X	x	X	x
**Gastro-intestinal side effects**					
Change in Gastrointestinal Symptom Rating Scale Questionnaire (GSRSQ) score between baseline and week 24	X				X

Table 4. Safety outcomes

5. STATISTICAL METHODS AND REPORTING

5.1 Sample size

Based on an average six minute walking distance of 495±92 m, as previously reported in stable KTRs, an anticipated improvement in walking distance of at least ten percent, a desired power of 0.90 with a significance level of 0.05, we calculated that a sample size of 72 participants per arm would be needed to establish a clinically meaningful effect of intravenous iron on the primary endpoint, using a two-sided independent samples t-test. Initially, to account for possible dropout of up to 10% of enrolled participants, we aimed to include 79 participants per arm (158 participants in total). Because the dropout rate was much lower (only one participant died during the study, and no participants dropped out for other reasons), and because of a stagnation in inclusion rate, 148 participants enrolled after which screening and inclusion were stopped.

5.2 Randomization, database lock and unblinding

Participants were randomized by the UMCG hospital pharmacy using a precomposed list of usable participant numbers, which are assigned to a study arm. The participant numbers on the list are grouped in blocks, with an equal distribution and a random order of treatment arm allocations within each block. To stratify for treatment center, all study numbers within a certain block are assigned to participants in the same center, in the order of the list. Other than to the pharmacist, the list is not accessible to any of the investigators or to others. In

case of a medical emergency possibly related to the study treatment, treatment allocation may be revealed by the pharmacist.

An exception was made during the COVID19 pandemic, when vaccination efficacy in a limited number of participants was compared between the study arms, as was described in version 5 of the study protocol. A researcher who was not involved in the assessments and analysis of data not directly related to the effects of the treatment on the immune system was deblinded to treatment allocation of that specific subgroup of participants. The results of this substudy were published earlier.[2]

Since the EFFECT-KTx study is considered to be low-risk, treatment allocation has not been revealed to a data and safety monitoring board for an interim analysis.

After the last study visit is completed and when all data are collected, verified and registered in the electronic data capture tool (REDCap), besides global longitudinal strain, which will be analyzed from echography material at a later time point, and results of laboratory analyses which will be performed at a later time point as well, the database will be locked. Unblinding for the final analysis will occur after the database lock. Data may be corrected afterwards but will require an explanation and will be tracked.

5.3 Protocol violations

One protocol violation occurred. During the conduction of the study, the study protocol and the participant information file (PIF) were updated three times. Potential participants received the last updated version of the PIF at their time of screening. However, the protocol and PIF version number on the informed consent forms was not updated accordingly. Therefore, officially, a large number of participants signed an informed consent form referring to an outdated version of the protocol and the PIF. In a study team meeting, which took place after the last participant had enrolled, it was decided that this protocol violation would not affect the results of the study and that participants did not have to be asked to sign an updated version of the informed consent file in retrospect.

Protocol deviations were registered in the trial master file.

5.4 Study analysis sets

Management of missed visits during the COVID19 pandemic

Between March and June 2020, recruitment was temporarily halted because of the COVID19 pandemic. Between March and May 2020, no in-hospital study visits were allowed. Given the uncertainty about the duration of the pandemic, it was decided that for participants who

missed two or more study treatments, of which at least one because of the pandemic restrictions, participation would be terminated. In that case, the participant would not be included in the completion of the aimed sample size and a new participant would be recruited instead. Participants who were excluded because of missed study drug administrations during the COVID-19 epidemic were invited for a rescreening under a new study number, providing they were still iron deficient. End-of-study visits during the pandemic were either postponed, with a maximum of two weeks, or were performed at the participant's home if possible. This procedure was decribed in version 4 of the study protocol and has been approved by the medical ethical committee of the UMCG.

Three analysis sets of subjects will be used for statistical analysis of the study.

5.4.1. Full analysis set

The full analysis set includes all randomized subjects according to the Intention-To-Treat principle (ITT), except for:

1. Participants who withdrew early because of multiple missed visits during the COVID19 pandemic, because of absence of most post-baseline data, as was decided in version 4 of the study protocol.
2. Participants who were randomized in the intervention (FCM) arm but did not receive any study treatment.
3. Participants of whom no post-baseline data at all are available for efficacy analysis.

Any exclusion from the full analysis set will be documented in the Trial Master File and shown in a flow diagram in the study paper.

5.4.2. Per protocol set

The supportive per-protocol set includes all subjects from the full analysis set, excluding:

- Subjects violating inclusion/exclusion criteria.
- Subjects deviating from the protocol guidelines during the trial, in a sufficiently serious manner to warrant data (not subject) exclusion (see section 5.4).
- Participants who were randomized in the intervention arm but did not receive study medication on three or more study visits.
- Participants who received a blood transfusion, erythropoiesis-stimulating agents or other iron supplements besides the study treatment.

- Participants who had a 6MWT that was not considered valid by the blinded researcher will be excluded from the per-protocol set.
- Subjects who withdraw from the trial resulting in missing data, in a sufficiently serious manner to warrant subject exclusion.

Any exclusion from the per-protocol set will be documented in the Trial Master File and shown in a flow diagram in the study paper.

The per protocol set will be used for a supportive analysis of the primary outcome. Potential differences in results between the analyzed data sets will be discussed.

5.4.3. Safety analysis set

Safety analysis population includes all subjects who received at least study one treatment, irrespective of protocol violations. This subsets includes the participants who withdrew early because of multiple missed visits because of the COVID19 pandemic. The safety analysis set will be used for analysis of safety outcomes.

Any exclusion from the safety population will be documented in the Trial Master File and shown in a flow diagram in the study paper.

5.5 Study participants characteristics

A description of the study population (using the primary study analysis set) will be given using the study subjects characteristics at baseline. Baseline characteristics include medical history (including type of primary renal disease, history of dialysis, presence of diabetes mellitus, hypertension, heart failure and recent infectious diseases), lifestyle characteristics, transplantation characteristics, type of immunosuppressive therapy, use of other relevant medication, body mass index, haemoglobin and iron status, estimated glomerular filtration rate and C-reactive protein.

Baseline characteristics will be presented using descriptive statistics, i.e. for quantitative parameters the mean and standard deviation or median and interquartile range with number of valid observations, depending on normality of data. Mean and median will be reported to a precision of the number of decimal places as is used in clinical practice.
For qualitative parameters (categorical or ordered), frequency counts and percentages of each category will be calculated by treatment group. Baseline characteristics will be summarized

overall, and by treatment group. Testing on differences between treatment groups will not be undertaken.

5.6 Efficacy outcome

5.6.1 Primary efficacy outcome

The primary efficacy outcome is the change in number of meters walked during the 6MWT from baseline to week 24. Change is calculated as value at week 24 – value at baseline.

5.6.2 Secondary efficacy outcome

Secondary efficacy outcomes are listed in table 2. The word ‘change’ should be interpreted as absolute change (new value – old value).

All biochemical parameters are measured systematically according to study protocol. Transferrin saturation (TSAT) is calculated as 100×plasma iron (μmol/L)÷(plasma transferrin (g/L)×25).

5.6.3 Exploratory outcomes

Regarding the echographic parameters, which are collected systematically according to study protocol, the relative wall thickness, the left atrial volume indexed to body surface area, the left ventricular mass index, the left ventricular ejection fraction, the early diastolic tissue velocity, the E/A ratio and the E/e’ ratio are calculated from the primarily observed echographic parameters.
The Heart Failure Organisation preserved ejection fraction score has functional, morphological and biomarker domains, and is described in detail elsewhere.[3]

Regarding parameters of cognitive function, assessed systematically according to study protocol, results of separate test scores as well combined scores for specific cognitive domains will be analyzed.
The combined score for memory is calculated as the mean of the norm scores of Digit Span Forward test score, 15 Word Test Immediate recall test score and 15 Word Test delayed recall test score (corrected for Word Test Immediate recall test score).

The combined score for mental speed is calculated as the mean of the norm scores of the Symbol Digit Modalities Test score and the Trail Making Test part A score.
The combined score for attention and executive function is calculated as the mean of the norm scores of the Trail Making Test part B score, the Controlled Oral Word Association Test score and the Digit Span Backward score.

5.7 Safety outcome

Safety outcomes are listed in table 3. The estimated glomerular filtration rate (eGFR) will be calculated using the Chronic Kidney Disease Epidemiology Collaboration (CKD-EPI) equation.[4]

5.8 Statistical methods used for efficacy and safety

Descriptive statistics
For quantitative parameters, descriptive statistics will be mean and standard deviation (normally distributed data) or median and interquartile range (data with a skewed distribution), and number of valid observations by treatment group. Mean or median will be reported to a precision of the number of decimals as used in clinical practice.

For qualitative parameters (categorical or ordered), frequency counts and percentages of each category will be calculated by treatment group. Where applicable, 95% confidence intervals around percentages will be calculated.

Efficacy and safety analyses
Regarding continuous variables assessed at baseline and week 24 only, differences between treatment groups will be evaluated using an analysis of covariance (ANCOVA) model with treatment center (UMCG vs UMCU), and baseline value of the assessed parameter as covariates.

Regarding continuous variables assessed at more than two study visits, mixed model analysis adjusted for study visit, treatment center and baseline value of the assessed parameter will be used.

Regarding clinical events (as part of the safety outcomes), they will be reported as number of events per treatment group. It is expected that numbers will be too low for statistical analysis.

Subgroup analysis

Several subgroup analyses will be performed to examine the consistency of the observed treatment effect and to gain insight into the effectiveness of Ferric carboxymaltose in subpopulations. Analyses of the primary endpoint will be conducted for the following subgroups:

- Participants with vs without anaemia (hemoglobin <13 g/dL in men or <12 g/dL in women)
- Participants with a ferritin level < vs ≥ 100 μg/L
- Participants with a transferrin saturation < vs ≥ 20%
- Participants with an estimated glomerular filtration rate of < vs ≥ 60 mL/min/1.73m^2
- Participants with a 6MWT score < vs ≥ the median
- Participants with a LVEF < vs ≥ 50%

Definition of statistical significance

The tests will be reported with one-sided p-values inthe direction favoring ferric carboxymaltose. A P-value less than 0.05 indicates statistical significance.

Missing data

A last observation carried forward (LOCF) imputation will be used for missing data of assessments which should have been performed at more than two study visits. Missing data of assessments which should have been performed at baseline and week 24 only will be subjected to multiple imputation assuming they are missing at random (MAR) with application of Rubin's rule combining analysis results of five imputation datasets.

Handling of multiple testing

Multiplicity testing because of multiple different outcomes will not be taken into account using a statistical correction method. Because the study is not powered to show significant changes in secondary outcomes, safety outcomes or exploratory outcomes, and sice there is no clear hierarchical order in these outcomes, correcting for multiplicity testing would increase the risk of a type II error. However, the potential risk of a type I error caused by the large number of exploratory outcomes will be mentioned in the discussion section of the study manuscript.

Statistics Software

All analyses will be performed using commercially available computer software IBM SPSS Statistics 28.

6. PRESENTATION OF RESULTS

Statistical output will be generated in accordance to this Statistical Analysis Plan and results will be presented in accordance with international standards for reporting clinical studies, as a manuscript to be submitted to a medical journal.

In addition, results of all analyses will be presented in more detail using tables of baseline data, efficacy results and safety results. In addition, results can be graphically presented.

7. CHANGES FROM PROTOCOL AND REMARKS

**Protocol version 4, June 2020 (changes from protocol version 3 - the first approved version of the protocol, June 2019)**	**Protocol version 5, Februari 2021 (changes from protocol version 4)**	**Protocol version 6, May 2022 (changes from protocol version 5)**	**Rationale and design paper[1] (Changes from protocol version 6)**	**Statistical Analysis Plan (Changes from protocol version 6 and from protocol paper)**
Management of missed visits during the COVID19 pandemic was describes: study participation of participants who missed two or more study visits because of hospital study visit restrictions was terminated. A new participant was	The effect of FCM vs placebo on SARS-CoV2 vaccination efficacy was added as an additional outcome assessed in a limited number of participants.	The UMCU was introduced as a second study site instead of the Erasmus MC. The local researcher at the Erasmus MC concluded that conduction of the study would not be feasible.	Urine markers of possible early rejection were changed based on new scientific insights.	To be able to adjust for baseline values and treatment center, changes from baseline to week 24 will not be analysed using a paired samples t-test (as was described in protocol version 6) or with mixed model analysis (as was described

recruited instead.				in the protocol paper) but with an ANCOVA model. Changes from baseline to week 6, 12, 18 and 24 will be analyzed mixed models analyses instead of a paired t-test or a Wilcoxon-signed rank test.
Two more quality of life instruments were added: the checklist individual strength and the Dutch Multifactor Fatigue Scale.				A planned evaluation of the correlation between change in haematinic parameters and other efficacy outcomes was included in protocol version 6 but was not included in the final analysis plan.
It was described that not only a nurse, but also a specifically trained doctor's assistant was allowed to take the role of unblinded study staff.				In protocol version 6 it is described that the incidence of clinical events will be analyzed using survival analysis. However, as stated in the statistical analysis plan, numbers of specific clinical events will be mentioned but not statistically

				analyzed, because of an expected power issue.
The volume of blood withdrawn per study visit was slightly adjusted to new insights in a more efficient protocol for blood distribution into aliquots.				After publication of the design paper, the aimed sample size was reduced from 158 to 148 because of lower than expected dropout and a stagnation in inclusion rate.
				In the statistical analysis plan, the description of the three different dataset was updated in relation to the design paper.
				In the design paper it was stated that missing data imputation will only be used in case of >10% missing data. However, as is stated in de SAP, imputation will also be performed in case of a lower percentage of missing data.
				Most of the outcomes that were classified as

				‘secondary’ in the protocol paper were classified as ‘exploratory’ in the SAP, because we realized the study was not powered to show a significant effect on any of these outcomes and correcting for multiplicity testing, which would be adequate when these outcomes would be considered secondary, would cause a risk of a type II error.

8. REFERENCES

1. Vinke JS, Eisenga MF, Sanders JF, Berger SP, Spikman JM, Abdulahad WH, Bakker SJ, Gaillard CAJM, van Zuilen AD, van der Meer P de BM. Effect of Intravenous Ferric Carboxymaltose on Exercise Capacity After Kidney Transplantation (EFFECT-KTx): rationale and study protocol for a double-blind, randomised, placebo-controlled trial. *BMJ Open*. 2023;13(3):e065423. doi:10.1136/bmjopen-2022-065423

2. Vinke JSJ, Altulea DHA, Eisenga MF, Jagersma RL, Niekolaas TM, van Baarle D, Heiden MV, Steenhuis M, Rispens T, Abdulahad WH, Sanders JF DBM. Ferric carboxymaltose and SARS-CoV-2 vaccination-induced immunogenicity in kidney transplant recipients with iron deficiency: The COVAC-EFFECT randomized controlled trial. *Front Immunol*. 2023;13. doi:10.3389/fimmu.2022.1017178

3. Pieske B, Tschöpe C, de Boer RA, Fraser AG, Anker SD, Donal E, Edelmann F, Fu M, Guazzi M, Lam CSP, Lancellotti P, Melenovsky V, Morris DA, Nagel E, Pieske-Kraigher E, Ponikowski P, Solomon SD, Vasan RS, Rutten FH, Voors AA, Ruschitzka

F, Paulus WJ, Sefer FG. How to diagnose heart failure with preserved ejection fraction: the HFA-PEFF diagnostic algorithm: a consensus recommendation from the Heart Failure Association (HFA) of the European Society of Cardiology (ESC). *Eur J Hear Fail*. 2020;22(3):391-412. doi:10.1002/ejhf.1741

4. Inker LA, Schmid CH, Tighiouart H, Eckfeldt JH, Feldman HI, Greene T, Kusek JW, Manzi J, Van Lente F, Zhang YL, Coresh J LAC-EI. Estimating glomerular filtration rate from serum creatinine and cystatin C. *N Engl J Med*. 2012;367(1):20-29. doi:10.1056/NEJMoa1114248
