# Supplementary material for: Intravenous ferric carboxymaltose and exercise capacity in iron-deficient kidney transplant recipients: a randomised, placebo-controlled trial in the Netherlands
Source: eClinicalMedicine. 2026 Sep 1;99:104169. doi: 10.1016/j.eclinm.2026.104169 (PMC13571158; doi:10.1016/j.eclinm.2026.104169)
Supplement: Supplementary file 3 [file mmc3.pdf]

Supplementary Materials

Index

Supplementary Methods

Laboratory assessments **p. 2-3**

Functional tests **p. 3-4**

Echocardiography **p. 4-5**

Neurocognitive assessments **p. 5-7**

Participant questionnaires **p. 7-8**

Supplementary Tables

Supplementary Table 1. Inclusion and exclusion criteria **p. 9**

Supplementary Table 2. Overview of secondary outcomes across protocol, registry, and manuscript **p. 10-15**

Supplementary Table 3. Reasons for exclusion from per-protocol sensitivity analysis **p. 16**

Supplementary Table 4. Protocol deviations concerning study treatment **p. 17**

Supplementary Table 5.1 to 5.9. Overview of efficacy results **p. 18-40**

Supplementary Table 6.1 and 6.2. Overview of safety parameters **p. 41-42**

Supplementary Table 7. Incidence of adverse events **p. 43-45**

Syntax **p. 46-210**

Study Protocol **p. 211-278**

Supplementary Methods

Laboratory assessments

Blood samples were drawn at all study visits before administration of the study medication. Participants were asked to collect 24-hour urine samples before every study visit. Levels of iron, haemoglobin, creatinine, phosphate and calcium in blood as well as concentrations of creatinine, protein, phosphate and calcium in urine were measured using enzymatic (creatinine), turbidimetric (protein) or colorimetric spectrophotometry assays.

Electrochemiluminescence immunoassays were used to measure plasma ferritin, parathyroid hormone (PTH), high-sensitivity troponin T and N-terminal pro-B-type natriuretic peptide (NT-proBNP) as well as urine sodium concentration. Levels of transferrin and cystatin C in plasma and albumin in urine were measured using an immunoturbidimetric assay. Kinetic assays were used to analyse plasma levels of aspartate aminotransferase and alanine aminotransferase. Haemoglobin levels were routinely measured on Sysmex XN automated haematology analysers (Sysmex Europe, Norderstedt, Germany). All other assays described above were performed on Roche Cobas clinical chemistry platforms (Roche, Mannheim, Germany). Enzyme-linked immunosorbent assays were used to measure urine kidney injury molecule-1 (KIM-1) and urine neutrophil gelatinase-associated lipocalin (NGAL, R&D Duoset, DY1750/DY1750B & DY1757) and intact and C-terminal fibroblast growth factor 23 (FGF23, QuidelOrtho, 60-6100 & 60-6600).

Anaemia was defined as haemoglobin <13 g/dL in men or <12 g/dL in women. Transferrin saturation (TSAT, %) was calculated as 100 x plasma iron (µmol/L) ÷ (25 × transferrin [g/L]). Excretion of creatinine, phosphate, calcium, kidney injury molecule-1, neutrophil gelatinase-associated lipocalin, protein and albumin were calculated from concentrations in 24-hour urine and total volume of 24-hour urine. Creatinine clearance (mL/min) was calculated as 24-hour urine creatinine excretion (mmol/24 hour) divided by plasma creatinine

level (mmol/L)/1440. Fractional sodium excretion was calculated as (plasma creatinine (mg/dL) * urine sodium (mmol/L) * 100) divided by (plasma sodium (mmol/L) * urine creatinine (mg/dL)). Excretion rates and creatinine clearance were not calculated from samples of incomplete urine collections. The 2021 CKD-EPI creatinine equation, the 2021 CKD-EPI creatinine-cystatin C equation and the 2012 CKD-EPI cystatin C equation (all without race) were used to estimate glomerular filtration rate (eGFR).

Functional Tests

Exercise capacity was measured using the six-minute walk test (6MWT), a validated measure of exercise capacity. Participants were asked to walk as many rounds as possible on a quiet 15-metre walking course with a flat, hard surface, marked with traffic cones at the beginning and the end, during six minutes. Participants could use a walking cane as needed. The researcher encouraged the participant to walk as fast as possible, but running was not allowed. Predicted six minute walk test distance was calculated based on sex, age, length and weight as was described previously (Delbressine JM. Reference values for six-minute walk distance and six-minute walk work in Caucasian adults. Pulmonology 2023).

The Timed Up-and-Go (TUG) Test is an instrument to assess leg strength, coordination and balance. A traffic cone was placed 3 metres before a chair and participants were instructed to stand up from the chair, again without support of their arms, walk around the cone as fast as possible and sit down on the chair again, while the time was recorded. The test was also performed three times, and the mean of the attempts was calculated.

Handgrip strength was measured with the Jamar Hydraulic Hand Dynamometer (Jamar 5030J1, Patterson Medical, Warrenville, Canada). Participants were asked to clench the device with maximum force to perform an isometric contraction, first with the right hand and then with the left hand, while sitting on a chair. Participants were not allowed to rest their

elbow on a table. Handgrip strength was tested three times with an interval of 30 seconds between each attempt, and the mean was calculated. The second handle position of the hand dynamometer was used because it has been shown to be the most accurate position.

The Five Times Sit-to-Stand (FTSTS) Test was used to measure leg strength by using the participant's body weight as resistance. Participants were instructed to stand up from a chair five times successively as fast as possible, without support of their arms. The time required to do this was recorded. The task was repeated three times, and the mean of the attempts was calculated.

Echocardiography

Cardiac structure, volumes, systolic and diastolic function and global longitudinal strain were analysed with a resting transthoracic echocardiography. A Vivid E90 cardiovascular ultrasound system (GE Healthcare, Chicago, USA) was used. Imaging and analysis were performed by a sonographer under supervision of a cardiologist, both blinded to treatment allocation.

Ventricular wall thickness, specifically interventricular septum thickness (IVSD), left ventricular end-diastolic thickness (LVEDD) and left posterior wall thickness (LVPWD), and atrial and ventricular volumes, specifically left atrial volume (LAV) and left ventricular end diastolic volume (LVEDV) and end systolic volume (LVESV) were measured using two-dimensional echocardiography. Left ventricular mass index (LVMI) was calculated using the Devereux equation: $(0{\cdot}8 \times (1{\cdot}04 \times ((((LVEDD + IVSD + LVPWD) / 10)^3) - ((LVEDD / 10)^3)) + 0{\cdot}6)$ / body surface area (BSA) (Devereux RB. Echocardiographic assessment of left ventricular hypertrophy: comparison to necropsy findings. Am J Cardiol 1986). BSA was calculated using the Dubois equation: weight $(kg)^{0.425} \times$ height $(cm)^{0.725} \times 0{\cdot}007184$ (Du Bois D. A formula to estimate the approximate surface area if height and weight be known.

Nutrition 1989). Relative wall thickness (RWT) was calculated as (2 x LVPWD) / LVEDD. Left atrial volume index (LAVI) was calculated as LAV / BSA. Left ventricular ejection fraction (LVEF) as the main parameter of cardiac function was calculated using the Simpson biplane method ((LVEDV-LVESV) x 100) / LVEDV) if image quality was sufficient, or was alternatively assessed by eyeballing. Doppler imaging was used to assess diastolic function and blood flow velocity distal to the valves, specifically tricuspid annular plane systolic excursion (TAPSE), a parameter of right ventricular function, systolic tissue velocity of the tricuspid valve annulus (s'TrV annulus), early diastolic lateral and septal tissue velocity (e'lat and e'sept) and early and late mitral valve diastolic inflow (MV-E vel and MV-A vel). Pressure gradients were calculated from these flow velocities: E/A-ratio was calculated as MV-E vel / (mean of e'lat and e'sept) and E/e'-ratio was calculated as MV-E vel/MV-A vel. Global ventricular longitudinal strain (GLS) and atrial contractile strain were evaluated as early measures of impaired myocardial contractility, using cardiac performance analysis with speckle tracking. The HFA-PEFF score was calculated using the LVMI, RWT, LAVI, LVPWD, e'lat, e'sept, s'TrV, E/e' ratio, GLS and plasma NT-proBNP level (Pieske B. How to diagnose heart failure with preserved ejection fraction: the HFA-PEFF diagnostic algorithm: a consensus recommendation from the Heart Failure Association (HFA) of the European Society of Cardiology (ESC). Eur J Heart Fail 2020). A HFA-PEFF score of two to four points implicates possible heart failure with a preserved ejection fraction, while a score of five or six points is diagnostic of heart failure with a preserved ejection fraction.

Neurocognitive assessments

Participants who met criteria for a neurodegenerative disorder based on a cognitive screening test, who had an estimated intelligence quotient of <80 based on the Dutch version of the National Adult Reading Test, or who had insufficient comprehension of the Dutch language, were excluded from further neurocognitive assessments.

Immediate memory span was measured with the Digit Span Forward subtest of the Wechsler Adult Intelligence Scale IV. Participants were asked to repeat a series of numbers after the examiner has read them out loud. The score was calculated as the total number of strings repeated correctly, with a maximum of sixteen. Norm scores adjusted for age were used.

The 15 Words Test, a Dutch version of the Rey Auditory Verbal Learning Test, was used to measure verbal memory. Fifteen unrelated words were presented in five trials. Participants were asked to recall as many as possible after each trial (immediate recall test). The score was calculated as the total number of words recalled, with a maximum of 75. After twenty minutes, participants were asked again to recall as many of the fifteen words as possible (delayed recall), with a maximum score of fifteen. Norm scores adjusted for age, sex and/or education were used.

The combined score for memory was calculated as the mean of the norm scores of the Digit Span Forward, the 15 Words Test immediate recall and the 15 Words Test delayed recall.

The Symbol Digit Modalities Test measures information processing speed. Participants were shown nine symbols which were each assigned to a number. Subsequently, they were given a list of those symbols in a random order and were asked to write as many matching numbers below the symbols as possible. The score was the total correct matches within ninety seconds with a maximum of 110. Norm scores adjusted for age and educational level were used.

The Trail Making Test part A measures visuomotor and mental speed. Participants were asked to connect a series of randomly distributed numbers from one to 25 in ascending order as fast as possible. Completion of the test was timed in seconds. A norm score adjusted for age and educational level was used.

The combined score for mental speed was calculated as the mean of the norm scores of the Symbol Digit Modalities Test and the Trail Making Test part A.

The Trail Making Test part B measures cognitive flexibility. Participants were asked to connect numbers and letters as fast as possible in an alternating ascending order. The total score was time (in seconds) to complete the test. A norm score of the ratio between the Trail Making Test part B and part A adjusted for age, sex and educational level was used.

The Controlled Oral Word Association Test measures executive control. Participants had to produce as many words as possible that started with a specific letter within one minute. Total scores from three different starting letters (D, A and T) were added up. Norm scores adjusted for age and educational level were used.

The Digit Span Backward subtest of the Wechsler Adult Intelligence Test measures working memory, which is considered an aspect of executive functioning. Participants were asked to repeat a series of numbers in the opposite order as the examiner has read them out. The score was the total number of strings repeated correctly, with a maximum of sixteen. Norm scores adjusted for age were used.

The combined score for attention and executive functioning was calculated as the mean of the norm scores of Trail Making Test part B, the Controlled Oral Word Association Test and the Digit Span Backward Test.

Participant questionnaires

Participants were requested to complete five questionnaires at baseline and after 24 weeks, to assess quality of life, level of fatigue and the occurrence of gastrointestinal side effects. The Short Form-36 (SF36) is a 36-item questionnaire which was used to measure health-related quality of life. The instrument provides separate scores for nine different domains of quality of life: physical functioning, social functioning, physical role limitations, emotional role

limitations, emotional well-being, vitality, pain, general health and health changes. Higher scores correspond to better quality of life.

Subjective fatigue was assessed using the Checklist Individual Strength (CIS) and the Dutch Multifactor Fatigue Scale (DMFS). The CIS is a Dutch 20-item questionnaire with total scores ranging from twenty to 140, higher scores indicating worse fatigue, with problematic fatigue at scores above 75. The DMFS contains 38 items and five domains: impact of fatigue, direct consequences of fatigue, mental fatigue, physical fatigue and coping with fatigue. Higher scores indicate worse fatigue.

Overall quality of life was measured with the EuroQol-5D-5L (EQ-5D-5L) Questionnaire and the EuroQol visual analogue scale. Five scores for different domains are provided by the questionnaire: mobility, self-care, usual activities, pain & discomfort, anxiety & depression. Index values were calculated with Dutch norm scores, higher scores correspond to better quality of life. The visual analogue scale records the participant's overall health, ranging from 'the worst health you can imagine' to 'the best health you can imagine'.

Occurrence of gastrointestinal side effects was examined using the Gastrointestinal Symptom Rating Scale (GSRS) Questionnaire, a 15-item instrument of which the total score was calculated.

Supplementary Tables

Supplementary Table 1. Inclusion and exclusion criteria

**Inclusion criteria**	**Exclusion criteria**
Kidney transplant recipient	Known intolerance to intravenous iron products
Iron deficiency at two consecutive measurements with an interval of at least a week, defined as plasma ferritin<100 µg/L or plasma ferritin 100–299 µg/L with transferrin saturation <20%	Severe (haemoglobin<10·5 g/dL) or progressive anaemia (haemoglobin decline of ≥3·2 g/dL in 2 months) or a reduced mean corpuscular erythrocyte volume (<80 fL)
≥6 months after kidney transplantation	Recent history of gastrointestinal or urogenital blood loss or a positive faecal occult blood test
≥18 years of age	Erythrocyte transfusion ≤6 weeks prior to inclusion
Ability to comply with the study protocol	Polycythaemia (haemoglobin>15·3 g/dL) or a known history of haemochromatosis
Informed consent	Estimated glomerular filtration rate of ≤30 mL/min per 1·73 m^2
	Symptomatic coronary artery disease or myocardial infarction ≤4 weeks prior to inclusion
	Disability to walk
	Severe hypophosphataemia (plasma phosphate <0·35 mmol/L) ≤4 weeks prior to inclusion
	For women at childbearing age: pregnancy or disagreement to take adequate contraceptive precautions
	Signs of an active systemic infection
	Participation in another intervention study

Supplementary Table 2. Overview of secondary outcomes across protocol, registry, and manuscript

Outcome	Protocol v6.2	Registry	Manuscript	Explanation for deviation between protocol/registry/manuscript
Haemoglobin	Yes	Yes	Yes	Consistent
Haematocrit, mean corpuscular erythrocyte volume	Yes	No	Yes	Consistent with protocol
Ferritin	Yes	Yes	Yes	Consistent
TSAT	Yes	Yes	Yes	Consistent
Plasma iron	Yes (states 'Serum iron')	Yes	Yes	Consistent
Serum hepcidin	Yes	No	No	Measured but not included in the present analysis; will be reported separately
Plasma C-reactive protein	Yes	No	No	Measured but not included in the present analysis; will be reported separately

Outcome	Protocol v6.2	Registry	Manuscript	Explanation for deviation between protocol/registry/manuscript
Cardiac structure & function (echocardiography, LVEF, diastolic function, strain)	Yes	Yes	Yes	Consistent
NT-proBNP, troponin	Yes	Yes	Yes	Consistent
HFA-PEFF score	No (derived)	No	Yes	Post-hoc derived outcome (exploratory)
Muscle function (TUG, FTSTS, handgrip)	Yes	Yes	Yes	Consistent
24h urinary creatinine	No	Yes (‘Muscle mass’)	Yes	Reported as proxy for muscle mass
Serum creatinine	Yes	Yes	No	Measured but not included in the present manuscript; incorporated in eGFR calculations
Kidney function (eGFR equations, creatinine clearance)	No	No	Yes	Post-hoc derived outcomes
Kidney injury markers (KIM-1, NGAL)	Yes	Yes	Yes	Consistent

Outcome	Protocol v6.2	Registry	Manuscript	Explanation for deviation between protocol/registry/manuscript
Kidney injury markers (MCP-1, CD163, CD25, FOXP3 mRNA)	Yes	No	No	Not yet analysed; analyses are ongoing and will be reported separately
Proteinuria/albuminuria	No	No	Yes	Post-hoc analysis (not prespecified in protocol or registry)
Phosphate (plasma + urine)	Yes	Partly (states 'Phosphate level')	Yes	Reported according to protocol
Calcium (plasma + urine)	Yes	Partly (states 'Calcium level')	Yes	Reported according to protocol
Vitamin D	Yes	Yes	No	Not measured in the final study dataset and therefore not reported
PTH	Yes	Yes	Yes	Consistent
FGF23	Yes	Yes	Yes	Consistent
Cognitive function (memory span, verbal memory, semantic memory, processing	Yes	Yes	Yes	Reported according to registry

Outcome	Protocol v6.2	Registry	Manuscript	Explanation for deviation between protocol/registry/manuscript
speed, visuomotor and mental speed, cognitive flexibility, executive control, working memory)				
Quality of life (SF-36, CIS, DMFS, EQ-5D-5L)	Yes	Yes	Yes	Reported according to protocol
Infections	Yes	Yes	Yes	Reported as adverse events and presented in Supplementary Table 7
Hospitalisations	Yes	Yes	Yes	Reported as adverse events and presented in Supplementary Table 7
Cardiovascular events	Yes	Yes (cardiac events)	Yes	Reported as adverse events and presented in Supplementary Table 7
Graft failure/rejection	Yes	Yes (rejection only)	Yes	Reported as adverse events and presented in Supplementary Table 7

Outcome	Protocol v6.2	Registry	Manuscript	Explanation for deviation between protocol/registry/manuscript
Death	Yes	No	Yes	Reported as adverse events and presented in Supplementary Table 7
GSRS	Yes	No	Yes	Reported as safety parameter and presented in Supplementary Table 6.1
Hepatic injury markers (Serum Alanine aminotransferase and aspartate aminotransferase)	Yes	Yes	Yes	Reported as adverse events and presented in Supplementary Table 6.2
Restless legs	No	Yes	Yes	Reported as safety event in the Results section
Immunological parameters (peripheral blood mononuclear cells, cytokines, antibodies)	Yes	Yes	No	Measured but not included in the present analysis; will be reported separately
Intestinal microbiota	Yes	Yes	No	Not yet analysed; analyses are ongoing and will be reported separately
SARS-CoV-2 vaccination responses	Yes	Yes	No	Already published (Front Immunol 2023;13:1017178)

**Outcome**	**Protocol v6.2**	**Registry**	**Manuscript**	**Explanation for deviation between protocol/registry/manuscript**
Blood pressure	Yes ('Other outcomes')	No	Yes	Reported according to protocol
Urinary sodium concentration	No	No	Yes	Post-hoc analysis (not prespecified in protocol or registry)
Fractional sodium excretion	No	No	Yes	Post-hoc analysis (not prespecified in protocol or registry)
Predicted 6MWT	No	No	Yes	Post-hoc derived outcome

Supplementary Table 3. Reasons for exclusion from per-protocol sensitivity analysis

Reason	Number
Six-minute walk test performed at home during severe acute respiratory syndrome coronavirus 2 (SARS-CoV-2) pandemic	8
Participant took a break during the six-minute-walk test	3
Participant used a walking aid during one of the six-minute-walk test trials	2
Participant was not able to finish the six-minute-walk test	3
Participant had an ankle injury on the day of one of the six-minute-walk test trials	1
Participant performed the six-minute walk test with a leg prosthesis	1
Participant randomised into the treatment arm received less than three doses of ferric carboxymaltose	2
Participant received a blood transfusion, erythropoiesis-stimulating agents or oral iron supplements during study participation	5

Some participants met more than one exclusion criterion.

Supplementary Table 4. Protocol deviations concerning study treatment

Treatment protocol deviation	Reason	Number
Placebo instead of ferric carboxymaltose	High levels of ferritin or TSAT	5
Placebo instead of ferric carboxymaltose	Administrative error	1
No treatment instead of ferric carboxymaltose	Early withdrawal or death	5
No treatment instead of ferric carboxymaltose	Missed study visit	12
No treatment instead of ferric carboxymaltose	Low phosphate level	10
No treatment instead of placebo	Missed study visit	15
No treatment instead of placebo	Low phosphate level	4
No treatment instead of placebo	Withdrawal from the study protocol because of anaemia	4
No treatment instead of placebo	Severe hypertension	1
No treatment instead of placebo	Systemic infection	1

Supplementary Table 5.1 Overview of efficacy results (Primary outcome)

	Ferric carboxymaltose			Placebo			Treatment effect, ferric carboxymaltose vs placebo	
	**n (before imputation)**	**Baseline (SD)**	**Δ (mean, SD)**	**n (before imputation)**	**Baseline (SD)**	**Δ (mean, SD)**	**Estimate (95% CI)**	**P (two-sided)**
**Primary Endpoint**								
Six-minute Walk Test, m	72	515 (91)	+28 (59)	73	510 (114)	+19 (58)	+9 (-10 to +28)*	0·37
Six-minute Walk Test, percent predicted value, %	72	84·7 (12·6)	+3·0 (8·3)	73	80·8 (14·4)	+2·4 (8·3)	+0·6 (-2·1 to +3·3)*	0·67

* Baseline-adjusted mean difference (baseline-adjusted linear regression model).

Supplementary Table 5.2 Overview of efficacy results (Iron status and haemoglobin)

	Ferric carboxymaltose		Placebo		Treatment effect, ferric carboxymaltose vs placebo	
	**N**	**Estimated marginal means (95% CI)**	**N**	**Estimated marginal means (95% CI)**	**Estimate (95% CI)**	**P (two-sided)**
**Plasma ferritin, µg/L**						
- Baseline	75	46 (37 to 56)	73	48 (39 to 58)	n.a.	n.a.
- Week 6	70	160 (131 to 196)	70	48 (39 to 59)	3·48 (3·07 to 3·95)**	**<0·001**
- Week 12	69	271 (221 to 332)	68	43 (35 to 52)	6·63 (5·66 to 7·77)**	**<0·001**
- Week 18	70	413 (337 to 506)	65	45 (37 to 55)	9·52 (8·03 to 11·28)**	**<0·001**
- Week 24	72	551 (450 to 675)	73	46 (38 to 56)	12·47 (10·46 to 14·84)**	**<0·001**
**Transferrin saturation, %**						
- Baseline	75	21 (18 to 24)	73	23 (20 to 26)	n.a.	n.a.
- Week 6	70	28 (25 to 31)	70	21 (18 to 24)	+9·1 (+6·0 to +12·1)***	**<0·001**
- Week 12	69	32 (29 to 35)	68	22 (19 to 25)	+12·4 (+9·5 to +15·3)***	**<0·001**
- Week 18	70	35 (32 to 38)	65	21 (18 to 24)	+15·8 (+12·5 to +19·1)***	**<0·001**

- Week 24	72	37 (34 to 40)	73	22 (19 to 25)	+17·4 (+14·1 to +20·7)***	**<0·001**
**Plasma iron, µmol/L**						
- Baseline	75	11·4 (10·2 to 12·9)	73	12·7 (11·3 to 14·3)	n.a.	n.a.
- Week 6	70	14·2 (12·6 to 16·0)	70	11·8 (10·5 to 13·2)	1·3 (1·2 to 1·5)**	**<0·001**
- Week 12	69	15·5 (13·8 to 17·4)	68	12·4 (11·1 to 14·0)	1·4 (1·2 to 1·6)**	**<0·001**
- Week 18	70	16·5 (14·7 to 18·6)	65	11·6 (10·3 to 13·0)	1·6 (1·4 to 1·8)**	**<0·001**
- Week 24	72	16·9 (15·1 to 19·1)	73	12·2 (10·8 to 13·6)	1·6 (1·3 to 1·8)**	**<0·001**
**Plasma haemoglobin, g/dL**						
- Baseline	75	13·0 (12·6 – 13·4)	73	13·3 (12·9 – 13·7)	n.a.	n.a.
- Week 6	70	13·5 (13·1 – 14·0)	70	13·3 (12·9 – 13·7)	+0·6 (+0·3 to +0·8)***	**<0·001**
- Week 12	71	13·7 (13·3 – 14·1)	68	13·2 (12·8 – 13·6)	+0·8 (+0·5 to +1·1)***	**<0·001**
- Week 18	69	13·9 (13·5 – 14·4)	66	13·2 (12·8 – 13·6)	+1·0 (+0·7 to +1·3)***	**<0·001**
- Week 24	72	14·0 (13·6 – 14·4)	73	13·1 (12·7 – 13·5)	+1·1 (+0·8 to +1·5)***	**<0·001**
**Plasma hematocrite, L/L**						
- Baseline	75	0·40 (0·39 to 0·42)	73	0·41 (0·40 to 0·42)	n.a.	n.a.
- Week 6	70	0·42 (0·41 to 0·43)	70	0·41 (0·40 to 0·42)	+0·01 (+0·01 to +0·02)***	**0·002**

- Week 12	70	0·42 (0·41 to 0·43)	68	0·41 (0·40 to 0·42)	+0·01 (+0·01 to +0·02)***	**<0·001**
- Week 18	69	0·42 (0·41 to 0·43)	66	0·41 (0·39 to 0·42)	+0·02 (+0·01 to +0·03)***	**<0·001**
- Week 24	72	0·42 (0·41 to 0·44)	73	0·41 (0·39 to 0·42)	+0·02 (+0·02 to +0·03)***	**<0·001**
**Plasma Mean Corpuscular Erythrocyte Volume, fl**						
- Baseline	75	89 (87 to 91)	73	90 (88 to 92)	n.a.	n.a.
- Week 6	70	90 (89 to 92)	70	90 (88 to 92)	+1·2 (0·5 to +1·8)***	**0·001**
- Week 12	70	91 (89 to 93)	68	89 (89 to 91)	+2·5 (1·6 to +3·3)***	**<0·001**
- Week 18	69	92 (89 to 94)	66	89 (87 to 91)	+3·3 (2·3 to +4·4)***	**<0·001**
- Week 24	72	93 (91 to 95)	73	90 (88 to 91)	+3·9 (2·7 to +5·0)***	**<0·001**

** Adjusted geometric mean ratio from a linear mixed-effects model including treatment group, time point, treatment-by-time interaction, and treatment centre, using observed values as the dependent variable.

*** Adjusted mean difference from a linear mixed-effects model including treatment group, time point, treatment-by-time interaction, and treatment centre, using observed values as the dependent variable.

Supplementary Table 5.3a Overview of efficacy results (Skeletal muscle mass)

	Ferric carboxymaltose		Placebo		Treatment effect, ferric carboxymaltose vs placebo	
	**N**	**Estimated marginal means (95% CI)**	**N**	**Estimated marginal means (95% CI)**	**Estimate (95% CI)**	**P (two-sided)**
**Urine creatinine excretion, mmol/24 hour**						
- Baseline	64	12·0 (10·8 to 13·2)	65	12·7 (11·5 to 14·0)	n.a.	n.a.
- Week 6	51	12·2 (11·0 to 13·5)	45	12·7 (11·1 to 13·6)	1·05 (0·98 to 1·12)**	0·16
- Week 12	55	11·9 (10·7 to 13·2)	46	12·5 (11·3 to 13·9)	1·01 (0·95 to 1·07)**	0·84
- Week 18	55	11·7 (10·6 to 12·3)	48	12·2 (11·1 to 13·5)	1·02 (0·95 to 1·09)**	0·62
- Week 24	59	12·0 (10·7 to 13·3)	58	12·5 (11·2 to 13·9)	1·02 (0·95 to 1·09)**	0·67

** Adjusted geometric mean ratio from a linear mixed-effects model including treatment group, time point, treatment-by-time interaction, and treatment centre, using observed values as the dependent variable.

Supplementary Table 5.3b Overview of efficacy results (Skeletal muscle function)

	Ferric carboxymaltose			Placebo			Treatment effect, ferric carboxymaltose vs placebo	
	n (before imputation)	Baseline (SD)	Δ (mean, SD)	n (before imputation)	Baseline (SD)	Δ (mean, SD)	Estimate (95% confidence interval)	P (two-sided)
**Timed Up-and-Go Test, sec**	72	6·3 (1·9)	-0·2 (1·1)	69	6·3 (2·8)	+0·1 (1·2)	-0·3 (-0·7 to +0·1)*	0·19
**Hand Grip Strength Test, kg**	72	39·7 (12·1)	-0·1 (3·2)	73	42·7 (12·3)	+0·8 (3·1)	-0·8 (-1·8 to +0·2)*	0·11
**Five Times Sit-to-Stand Test, sec**	72	8·9 (2·5)	-0·5 (2·1)	72	9·5 (4·2)	-0·2 (2·0)	-0·4 (-1·1 to +0·3)*	0·29

* Baseline-adjusted mean difference (baseline-adjusted linear regression model).

Supplementary Table 5.4 Overview of efficacy results (Kidney function and injury)

	Ferric carboxymaltose		Placebo		Treatment effect, ferric carboxymaltose vs placebo	
	**N**	**Estimated marginal means (95% CI)**	**N**	**Estimated marginal means (95% CI)**	**Estimate (95% CI)**	**P (two-sided)**
**Estimated glomerular filtration rate (CKD-EPI 2021 without race), mL/min/ 1.73m²**						
- Baseline	75	54 (48 to 60)	73	57 (51 to 63)	n.a.	n.a.
- Week 6	70	56 (50 to 62)	70	57 (51 to 63)	+2·1 (+0·3 to +3·8)***	**0·02**
- Week 12	71	57 (51 to 63)	68	57 (51 to 63)	+3·4 (+1·4 to +5·3)***	**<0·001**
- Week 18	69	57 (51 to 63)	66	57 (51 to 63)	+3·2 (+1·1 to +5·3)***	**0·003**
- Week 24	72	58 (52 to 64)	73	56 (51 to 62)	+4·3 (+2·1 to +6·5)***	**<0·001**
**CKD-EPI Creatinine-Cystatin C Age, Sex Equation 2021, mL/min/ 1.73m²**						
- Baseline	71	48 (43 to 54)	63	52 (46 to 57)	n.a.	n.a.
- Week 6	70	50 (44 to 55)	69	51 (46 to 57)	+1·8 (+0·1 to +3·4)***	**0·04**
- Week 12	59	50 (45 to 56)	64	51 (46 to 60)	+1·9 (-0·1 to +3·9)***	0·07

- Week 18	68	50 (44 to 56)	65	51 (46 to 57)	+1·9 (-0·2 to +3·9)***	0·07
- Week 24	71	50 (45 to 56)	71	51 (45 to 56)	+2·5 (+0·3 to +4·6)***	**0·03**
**CKD-EPI Cystatin C Age, Sex Equation 2012, mL/min/ 1.73m^2**						
- Baseline	71	44 (39 to 50)	63	47 (42 to 53)	n.a.	n.a.
- Week 6	70	45 (39 to 50)	69	46 (41 to 52)	+1·6 (-0·3 to +3·6)***	0·10
- Week 12	59	45 (39 to 50)	64	47 (42 to 52)	+0·9 (-1·5 to +3·2)***	0·46
- Week 18	68	44 (39 to 50)	65	47 (42 to 52)	+0·7 (-1·7 to +3·1)***	0·57
- Week 24	71	44 (39 to 50)	71	46 (41 to 51)	+1·2 (-1·2 to +3·6)***	0·32
**Creatinine clearance, mL/min**						
- Baseline	64	72 (63 to 81)	65	76 (67 to 85)	n.a.	n.a.
- Week 6	72	74 (65 to 83)	69	76 (67 to 85)	+2·1 (-2·2 to +6·3)***	0·35
- Week 12	72	74 (65 to 83)	70	75 (66 to 84)	+3·6 (-0·7 to +7·8)***	0·10
- Week 18	73	74 (65 to 83)	70	75 (66 to 84)	+2·4 (-2·3 to +7·1)***	0·3
- Week 24	74	77 (67 to 85)	72	76 (67 to 85)	+4·1 (-1·1 to +9·2)***	0·12
**Urinary protein excretion, g/24h**						
- Baseline	64	0·35 (0·29 to 0·42)	65	0·31 (0·26 to 0·38)	n.a.	n.a.
- Week 6	50	0·29 (0·24 to 0·36)	44	0·29 (0·24 to 0·36)	0·91 (0·73 to 1·13)**	0·38
- Week 12	54	0·31 (0·25 to 0·37)	45	0·30 (0·25 to 0·37)	0·92 (0·73 to 1·15)**	0·44

- Week 18	53	0·36 (0·30 to 0·45)	47	0·29 (0·24 to 0·35)	1·13 (0·89 to 1·45)**	0·31
- Week 24	58	0·34 (0·28 to 0·41)	57	0·29 (0·24 to 0·35)	1·06 (0·82 to 1·37)**	0·64
**Urinary albumin excretion, mg/24h**						
- Baseline	64	47 (31 to 73)	65	51 (34 to 79)	n.a.	n.a.
- Week 6	50	47 (30 to 73)	44	53 (34 to 82)	0·96 (0·72 to 1·28)**	0·78
- Week 12	54	45 (29 to 70)	45	47 (30 to 72)	1·06 (0·78 to 1·44)**	0·71
- Week 18	53	41 (27 to 64)	47	52 (34 to 81)	0·86 (0·63 to 1·18)**	0·36
- Week 24	58	45 (29 to 70)	57	57 (37 to 87)	0·87 (0·65 to 1·16)**	0·34
**Urine kidney injury molecule-1 excretion, pg/24h**						
- Baseline	64	1377 (1141 to 1662)	64	1569 (1301 to 1891)	n.a.	n.a.
- Week 6	50	1421 (1173 to 1720)	44	1513 (1250 to 1832)	1·07 (0·88 to 1·29)**	0·49
- Week 12	54	1277 (1043 to 1562)	45	1419 (1156 to 1742)	1·02 (0·85 to 1·23)**	0·79
- Week 18	54	1286 (1028 to 1610)	46	1435 (1143 to 1803)	1·02 (0·84 to 1·24)**	0·84
- Week 24	57	1596 (1327 to 1920)	57	1643 (1368 to 1974)	1·11 (0·93 to 1·31)**	0·24
**Urine neutrophil gelatinase-associated lipocalin excretion, ng/24h**						

- Baseline	64	28653 (20171 to 40701	64	27778 (19634 to 39301)	n.a.	n.a.
- Week 6	50	31793 (22004 to 45890)	44	24441 (16916 to 35313)	1·26 (0·95 to 1·67)**	0·11
- Week 12	54	30485 (21742 to 42745	45	29912 (21354 to 41898)	0·99 (0·77 to 1·27)**	0·92
- Week 18	53	31226 (22004 to 44267)	46	31039 (21917 to 44002)	0·97 (0·74 to 1·29)**	0·86
- Week 24	57	27667 (19693 to 38871)	57	28912 (20682 to 40457)	0·93 (0·73 to 1·18)**	0·54

** Adjusted geometric mean ratio from a linear mixed-effects model including treatment group, time point, treatment-by-time interaction, and treatment centre, using observed values as the dependent variable.

*** Adjusted mean difference from a linear mixed-effects model including treatment group, time point, treatment-by-time interaction, and treatment centre, using observed values as the dependent variable.

Supplementary Table 5.5 Overview of efficacy results (Mineral homeostasis)

	Ferric carboxymaltose		Placebo		Treatment effect, ferric carboxymaltose vs placebo	
	**N**	**Estimated marginal means (95% CI)**	**N**	**Estimated marginal means (95% CI)**	**Estimate (95% CI)**	**P (two-sided)**
**Plasma total Fibroblast Growth Factor 23, RU/mL**						
- Baseline	73	166 (138 to 201)	72	163 (134 to 197)	n.a.	n.a.
- Week 6	66	114 (94 to 138)	68	166 (137 to 201)	0·67 (0·59 to 0·76)**	**<0·001**
- Week 12	69	102 (85 to 124)	67	171 (142 to 208)	0·58 (0·51 to 0·67)**	**<0·001**
- Week 18	63	98 (81 to 120)	63	182 (149 to 221)	0·53 (0·46 to 0·61)**	**<0·001**
- Week 24	70	100 (83 to 121)	71	175 (145 to 211)	0·56 (0·48 to 0·65)**	**<0·001**
**Plasma intact Fibroblast Growth Factor 23, pg/mL**						
- Baseline	73	93 (78 to 110)	72	89 (75 to 105)	n.a.	n.a.
- Week 6	66	87 (74 to 103)	67	86 (73 to 102)	0·98 (0·87 to 1·09)**	0·68
- Week 12	69	82 (70 to 97)	67	89 (76 to 105)	0·88 (0·79 to 0·99)**	**0·03**
- Week 18	63	78 (66 to 93)	64	88 (75 to 104)	0·86 (0·76 to 0·96)**	**0·009**
- Week 24	70	82 (70 to 96)	71	92 (79 to 108)	0·85 (0·77 to 0·95)**	**0·004**

**Plasma phosphate, mmol/L**						
- Baseline	75	0·87 (0·81 to 0·93)	73	0·89 (0·83 to 0·95)	n.a.	n.a.
- Week 6	70	0·85 (0·79 to 0·91)	70	0·87 (0·81 to 0·93)	0·00 (-0·05 to +0·05)***	0·99
- Week 12	71	0·83 (0·76 to 0·89)	68	0·85 (0·79 to 0·92)	-0·01 (-0·06 to +0·05)***	0·82
- Week 18	69	0·83 (0·77 to 0·90)	66	0·87 (0·81 to 0·93)	-0·01 (-0·07 to +0·04)***	0·63
- Week 24	72	0·81 (0·75 to 0·88)	73	0·88 (0·82 to 0·95)	-0·05 (-0·1 to +0·00)***	0·07
**Urine phosphate excretion, mmol/24 hour**						
- Baseline	64	29·0 (25·2 to 32·8)	65	31·4 (27·6 to 35·2)	n.a.	n.a.
- Week 6	51	27·6 (23·8 to 31·3)	45	28·1 (24·3 to 31·8)	+1·9 (-2·1 to +5·9)***	0·34
- Week 12	54	27·8 (24·3 to 31·3)	46	27·9 (24·4 to 31·4)	+2·3 (-1·1 to +5·7)***	0·18
- Week 18	54	26·4 (22·9 to 29·9)	48	29·3 (25·8 to 32·8)	-0·5 (-3·9 to +2·9)***	0·77
- Week 24	59	27·7 (24·1 to 31·4)	58	29·4 (25·8 to 33·0)	+0·8 (-2·5 to +4·1)***	0·65
**Plasma calcium, mmol/L**						
- Baseline	75	2·44 (2·40 to 2·48)	73	2·45 (2·41 to 2·49)	n.a.	n.a.
- Week 6	70	2·44 (2·40 to 2·48)	70	2·46 (2·42 to 2·50)	-0·01 (-0·04 to +0·02)***	0·56

- Week 12	71	2·45 (2·41 to 2·49)	68	2·44 (2·41 to 2·48)	+0·01 (-0·02 to +0·04)***	0·67
- Week 18	69	2·46 (2·41 to 2·50)	66	2·44 (2·40 to 2·48)	+0·02 (-0·01 to +0·06)***	0·18
- Week 24	72	2·44 (2·40 to 2·48)	73	2·45 (2·41 to 2·49)	-0·00 (-0·04 to +0·03)***	0·80
**Urine calcium excretion, mmol/24h**						
- Baseline	64	2·4 (1·9 to 3·0)	65	2·5 (2·0 to 3·2)	n.a.	n.a.
- Week 6	51	2·0 (1·6 to 2·6)	45	2·5 (1·9 to 3·2)	0·87 (0·72 to +1·05)**	0·14
- Week 12	54	2·3 (1·8 to 2·9)	46	2·6 (2·0 to 3·2)	0·94 (0·81 to +1·10)**	0·42
- Week 18	54	2·3 (1·8 to 3·0)	48	2·6 (2·0 to 3·3)	0·96 0·82 to +1·12)**	0·59
- Week 24	59	2·2 (1·7 to 2·8)	58	2·6 (2·1 to 3·3)	0·89 (0·76 to +1·05)**	0·16
**Plasma parathyroid hormone, pmol/L**						
- Baseline	74	8·5 (7·2 to 10·1)	72	7·8 (6·6 to 9·2)	n.a.	n.a.
- Week 6	66	8·6 (7·2 to 10·2)	64	7·6 (6·4 to 9·0)	1·04 (0·94 to 1·16)**	0·44
- Week 12	68	8·5 (7·2 to 10·0)	65	7·4 (6·3 to 8·7)	1·06 (0·95 to 1·17)**	0·30
- Week 18	65	8·4 (7·1 to 9·9)	62	7·9 (6·7 to 9·3)	0·98 (0·88 to 1·09)**	0·70

- Week 24	71	8·4 (7·1 to 9·9)	73	7·6 (6·4 to 8·9)	1·02 (0·92 to 1·13)**	0·72

** Adjusted geometric mean ratio from a linear mixed-effects model including treatment group, time point, treatment-by-time interaction, and treatment centre, using observed values as the dependent variable.

*** Adjusted mean difference from a linear mixed-effects model including treatment group, time point, treatment-by-time interaction, and treatment centre, using observed values as the dependent variable.

Supplementary Table 5.6a Overview of efficacy results (Cardiac structure and function)

	**Ferric carboxymaltose**			**Placebo**			**Treatment effect, ferric carboxymaltose vs placebo**	
	**n (before imputation)**	**Baseline (SD/IQR)**	**Δ (mean, SD)**	**n (before imputation)**	**Baseline (SD/IQR)**	**Δ (mean, SD)**	**Estimate (95% confidence interval)**	**P (two-sided)**
Interventricular septum thickness, mm	64	11·6 (2·8)	-0·2 (2·0)	62	11·4 (2·3)	-0·5 (2·4)	+0·3 (-0·4 to +1·0)*	0·39
Left ventricular end-diastolic thickness, mm	64	47·0 (6·5)	-0·7 (5·8)	61	47·3 (6·2)	-0·9 (6·7)	+0·2 (-1·7 to +2·2)*	0·81
Left ventricular posterior wall thickness, mm	64	9·4 (1·6)	+0·6 (1·9)	61	9·4 (1·8)	+0·6 (2·0)	+0·0 (-0·6 to +0·7)*	0·97
Relative wall thickness, mm	64	0·41 (0·10)	+0·04 (0·10)	61	0·41 (0·10)	+0·03 (0·12)	+0·00 (-0·03 to +0·04)*	0·91
Left ventricular mass index	64	90·2 (26·4)	+0·7 (20·5)	61	88·8 (20·4)	-1·9 (20·0)	+2·7 (-4·3 to +9·6)*	0·46
Left atrial volume index	61	28·7 (7·5)	-0·5 (9·1)	57	30·0 (10·8)	+2·1 (10·1)	-2·6 (-5·8 to +0·6)*	0·11
Left ventricular ejection fraction, %	63	61 (7)	-1·2 (7·5)	61	60 (7)	-0·6 (9·2)	-0·6 (-3·2 to +2·0)*	0·66
Tricuspid annular plane systolic excursion, mm	63	23·5 (4·2)	-0·7 (4·0)	63	22·5 (4·7)	-0·2 (4·5)	-0·5 (-1·9 to +0·9)*	0·47

Systolic tissue velocity of tricuspid valve annulus, m/sec	14	2·3 (0·3)	-0·01 (1·04)	20	2·3 (0·4)	-0·01 (0·96)	+0·00 (-0·17 to +0·17)*	0·98
E/A ratio	62	0·91 (0·35)	-0·01 (0·36)	57	1·01 (0·39)	+0·01 (0·40)	-0·02 (-0·14 to +0·09)*	0·69
E/e' ratio	63	0·08 (0·03)	+0·00 (0·06)	59	0·09 (0·06)	+0·00 (0·06)	+0·00 (-0·02 to +0·02)*	0·91
Global longitudinal ventricular strain, %	49	-17·0 (3·0)	-0·6 (3·1)	50	-17·3 (2·7)	+0·3 (3·6)	-0·9 (-2·0 to +0·2)*	0·12
Left atrial reservoir strain, %	53	27 (7)	-0·6 (8·8)	50	28 (8)	-1·6 (8·6)	+1·0 (-1·7 to +3·8)*	0·46
Left atrial contractile strain, %	53	15 (11 – 18)	-0·3 (5·5)	50	13 (10 – 16)	-1·1 (6·3)	+0·7 (-0·9 to +2·4)*	0·39
Left atrial conduit strain, %	53	13 (6)	-0·0 (7·2)	50	15 (7)	-0·2 (7·8)	+0·1 (-1·8 to +2·1)*	0·89
HFA-PEFF score	75	2·5 (1·7)	+0·2 (1·3)	73	2·4 (1·6)	-0·1 (1·3)	+0·3 (-0·1 to +0·7)*	0·13

* Baseline-adjusted mean difference (baseline-adjusted linear regression model).

Supplementary Table 5.6b Overview of efficacy results (Cardiac structure and function)

	Ferric carboxymaltose		Placebo		Treatment effect, ferric carboxymaltose vs placebo	
	**N**	**Estimated marginal means (95% CI)**	**N**	**Estimated marginal means (95% CI)**	**Estimate (95% CI)**	**P (two-sided)**
**Plasma troponin, ng/L**						
- Baseline	75	9·1 (7·4 to 11·1)	73	8·9 (7·3 to 10·8)	n.a.	n.a.
- Week 12	69	8·9 (7·2 to 10·9)	67	8·9 (7·3 to 10·9)	0·97 (0·92 to 1·03)**	0·32
- Week 24	71	8·9 (7·3 to 11·0)	73	8·8 (7·2 to 10·7)	1·00 (0·94 to 1·06)**	0·90
**Plasma N-Terminal pro-B-type Natriuretic Peptide, ng/L**						
- Baseline	75	154 (105 to 226)	73	148 (101 to 215)	n.a.	n.a.
- Week 12	69	180 (122 to 265)	67	135 (92 to 197)	1·27 (1·08 to 1·51)**	**0·004**
- Week 24	71	201 (137 to 295)	73	155 (106 to 225)	1·24 (1·04 to 1·49)**	**0·02**

** Adjusted geometric mean ratio from a linear mixed-effects model including treatment group, time point, treatment-by-time interaction, and treatment centre, using observed values as the dependent variable.

Supplementary Table 5.7 Overview of efficacy results (BP/sodium status)

	Ferric carboxymaltose		Placebo		Treatment effect, ferric carboxymaltose vs placebo	
	**N**	**Estimated marginal means (95% CI)**	**N**	**Estimated marginal means (95% CI)**	**Estimate (95% CI)**	**P (two-sided)**
**Systolic blood pressure, mmHg**						
- Baseline	75	138 (133 to 143)	73	133 (128 to 138)	n.a.	n.a.
- Week 6	70	134 (130 to 139)	69	133 (128 to 138)	-3·3 (-8·6 to +1·9)***	0·22
- Week 12	69	136 (131 to 141)	68	133 (128 to 138)	-1·3 (-6·5 to +3·9)***	0·63
- Week 18	69	135 (130 to 140)	64	133 (128 to 138)	-3·1 (-7·7 to +1·4)***	0·17
- Week 24	72	128 (123 to 133)	73	130 (126 to 135)	-7·1 (-12·9 to -1·2)***	0·02
**Urinary sodium, mmol/L**						
- Baseline	71	53 (45 to 62)	71	59 (51 to 69)	n.a.	n.a.
- Week 6	57	54 (46 to 63)	54	59 (50 to 69)	1·01 (0·88 to 1·16)**	0·85
- Week 12	63	57 (49 to 66)	61	61 (53 to 72)	1·03 (0·90 to 1·17)**	0·65
- Week 18	58	54 (46 to 63)	59	57 (49 to 66)	1·06 (0·92 to 1·22)**	0·43
- Week 24	67	57 (49 to 67)	70	64 (55 to 74)	1·00 (0·88 to 1·14)**	1·0

**Fractional sodium excretion, %**						
- Baseline	64	0·98 (0·86 to 1·13)	65	0·89 (0·78 to 1·02)	n.a.	n.a.
- Week 6	50	0·88 (0·76 to 1·01)	44	0·89 (0·77 to 1·03)	0·89 (0·76 to 1·05)**	0·17
- Week 12	54	0·98 (0·85 to 1·13)	45	0·95 (0·82 to 1·10)	0·93 (0·80 to 1·09)**	0·37
- Week 18	52	0·94 (0·82 to 1·09)	47	0·87 (0·76 to 1·01)	0·98 (0·84 to 1·14)**	0·76
- Week 24	57	0·97 (0·85 to 1·12)	56	0·93 (0·81 to 1·07)	0·94 (0·81 to 1·10)**	0·47

** Adjusted geometric mean ratio from a linear mixed-effects model including treatment group, time point, treatment-by-time interaction, and treatment centre, using observed values as the dependent variable.

*** Adjusted mean difference from a linear mixed-effects model including treatment group, time point, treatment-by-time interaction, and treatment centre, using observed values as the dependent variable.

Supplementary Table 5.8 Overview of efficacy results (cognitive function)

	**Ferric carboxymaltose**			**Placebo**			**Treatment effect, ferric carboxymaltose vs placebo**	
	**n (before imputation)**	**Baseline (SD/IQR)**	**Δ (mean, SD)**	**n (before imputation)**	**Baseline (SD/IQR)**	**Δ (mean, SD)**	**Estimate (95% confidence interval)**	**P (two-sided)**
Digit Span Test Forward score	62	37 (16 - 75)	+0·8 (29·6)	65	37 (16 - 63)	+3·7 (27·8)	-2·9 (-11·6 to +5·8)*	0·51
Word Fluency Test score	60	48 (26)	+4·1 (26·0)	63	46 (28)	+2·5 (31·1)	+1·6 (-6·4 to +9·6)*	0·70
15 Word Test (Immediate Recall) score	59	38 (11 - 66)	+11·7 (29·4)	65	38 (18 - 62)	+8·5 (32·5)	+3·3 (-6·5 to +13·0)*	0·51
15 Word Test (Delayed Recall) score	57	44 (16 - 73)	+13·9 (32·0)	62	44 (28 - 73)	+8·1 (31·6)	+5·8 (-5·3 to +16·8)*	0·31
Memory (combined score)	59	45 (18)	+5·3 (17·1)	65	46 (15)	+3·1 (17·3)	+2·2 (-3·8 to +8·2)*	0·47
Symbol Digit Modalities Test score	61	52 (9)	+1·5 (10·8)	65	53 (11)	+0·7 (9·4)	+0·8 (-2·1 to +3·7)*	0·59
Trail Making Test A score	61	58 (27 - 84)	+8·6 (23·1)	65	58 (24 - 82)	+8·9 (27·8)	-0·3 (-8·9 to +8·3)*	0·94
Attention and mental speed (combined score)	61	54 (37 - 73)	+4·3 (15·8)	63	56 (35 - 68)	+4·6 (16·5)	-0·3 (-4·9 to +4·3)*	0·90
Trail Making Test B score	61	54 (34 - 84)	+5·6 (28·3)	65	46 (24 - 79)	+9·2 (24·9)	-3·6 (-12·2 to +5·0)*	0·42
Trail Making Test B/A ratio	61	54 (38 - 82)	+1·6 (33·1)	65	42 (24 - 74)	+4·0 (30·2)	-2·5 (-11·7 to +6·7)*	0·60

Word Fluency Letter score	60	33 (15 - 61)	+4·0 (31·4)	61	33 (11 - 67)	+1·8 (25·6)	+2·2 (-6·7 to +11·1)*	0·63
Digit Span Test Backward score	62	41 (26)	+3·7 (29·5)	65	43 (30)	+5·3 (32·9)	-1·7 (-11·2 to +7·9)*	0·73
Executive functioning (combined score)	59	45 (21)	+3·9 (22·9)	61	44 (23)	+4·3 (29·2)	-0·4 (-7·5 to +6·7)*	0·91

* Baseline-adjusted mean difference (baseline-adjusted linear regression model)

Supplementary Table 5.9 Overview of efficacy results (Quality of life)

	**Ferric carboxymaltose**			**Placebo**			**Treatment effect, ferric carboxymaltose vs placebo**	
	**n (before imputation)**	**Baseline (SD/IQR)**	**Δ (mean, SD)**	**n (before imputation)**	**Baseline (SD/IQR)**	**Δ (mean, SD)**	**Estimate (95% confidence interval)**	**P (two-sided)**
**Short Form 36**								
Physical Functioning	68	85 (64 - 95)	+2·4 (20·4)	66	90 (66 - 95)	+3·6 (22·1)	-1·2 (-7·3 to +4·9)*	0·70
Social Functioning	67	88 (63 - 100)	+1·1 (23·4)	67	75 (63 - 100)	+5·5 (23·4)	-4·3 (-11·4 to +2·8)*	0·23
Mental Health	65	79 (15)	+1·7 (13·5)	67	82 (11)	+0·8 (13·1)	+0·8 (-3·5 to +5·2)*	0·71
Vitality	67	63 (19)	+1·9 (17·9)	65	63 (16)	-0·0 (20·5)	+1·9 (-4·0 to +7·9)*	0·52
Pain	66	47 (6)	+1·1 (7·2)	68	47 (7)	-0·2 (6·8)	+1·3 (-1·0 to +3·5)*	0·27
General Health	68	56 (18)	+2·0 (16·3)	68	55 (20)	-1·3 (16·7)	+3·3 (-2·0 to +8·5)*	0·22
Change in Health	69	75 (50 - 100)	-5·8 (29·8)	68	50 (50 - 100)	-6·6 (25·5)	+0·7 (-8·1 to +9·5)*	0·87
**Checklist of Individual Strength score**	65	62·5 (25·6)	-1·2 (19·8)	66	64·9 (22·4)	-2·0 (23·2)	+0·8 (-5·6 to +7·2)*	0·81
**Dutch Multifactor Fatigue Scale**								
Impact of Fatigue	64	27·4 (10·5)	-0·1 (10·9)	65	27·4 (10·2)	-1·0 (12·1)	+0·9 (-2·5 to +4·4)*	0·60

Direct Consequences of Fatigue	65	22·1 (6·8)	-1·0 (6·0)	66	22·8 (6·3)	-1·0 (7·4)	-0·0 (-2·1 to +2·0)*	0·99
Mental Fatigue	65	18·0 (5·8)	-0·7 (5·1)	65	19·0 (5·6)	-1·1 (5·1)	+0·4 (-1·1 to +2·0)*	0·58
Physical Fatigue	66	16·3 (4·9)	+0·1 (5·3)	65	16·8 (5·0)	+0·3 (5·4)	-0·3 (-1·8 to +1·2)*	0·73
Coping with Fatigue	65	14·6 (3·4)	+0·1 (3·5)	66	14·5 (3·3)	+0·4 (3·4)	-0·3 (-1·3 to +0·8)*	0·63
**Euroqol-5D-5L index score**	67	0·9 (0·8 - 1·0)	+0·0 (0·2)	68	0·9 (0·8 - 1·0)	+0·0 (0·2)	+0·0 (-0·1 to +0·1)*	0·91
**Euroqol-5D-5L visual analogue score**	65	75·0 (15·1)	-1·2 (21·7)	61	74·3 (14·8)	+1·2 (18·3)	-2·4 (-9·4 to +4·6)*	0·50

* Baseline-adjusted mean difference (baseline-adjusted linear regression model)

Supplementary Table 6.1 Overview of safety parameters

	Ferric carboxymaltose			Placebo			Treatment effect, ferric carboxymaltose vs placebo	
	n (before imputation)	Baseline	Δ (mean, SD)	n (before imputation)	Baseline	Δ (mean, SD)	Estimate (95% confidence interval)	P (two-sided)
**Gastrointestinal Symptom Rating Scale**	69	22 (18 - 28)	+0·8 (7·0)	70	22 (18 - 30)	+0·3 (6·8)	+0·5 (-1·8 to 2·7)*	0·68

* Baseline-adjusted mean difference (baseline-adjusted linear regression model)

Supplementary Table 6.2 Overview of safety parameters

	**Ferric carboxymaltose**		**Placebo**		**Treatment effect, ferric carboxymaltose vs placebo**	
	**N**	**Estimated marginal means (95% CI)**	**N**	**Estimated marginal means (95% CI)**	**Estimate (95% CI)**	**P (two-sided)**
**Aspartate aminotransferase, U/L**						
- Baseline	77	19 (17 to 21)	77	19 (17 to 21)	n.a.	n.a.
- Week 6	71	19 (17 to 21)	71	19 (17 to 22)	+0·3 (-1·6 to +2·1)***	0·76
- Week 12	71	19 (17 to 21)	68	19 (17 to 22)	+0·4 (-2·0 to +2·8)***	0·26
- Week 18	69	20 (18 to 23)	66	20 (17 to 22)	+1·3 (-0·9 to +3·5)***	0·26
- Week 24	72	20 (17 to 23)	73	19 (16 to 22)	+1·2 (-1·9 to +4·3)***	0·45
**Alanine aminotransferase, U/L**						
- Baseline	77	22 (18 to 26)	77	22 (18 to 25)	n.a.	n.a.
- Week 6	71	23 (20 to 27)	71	22 (18 to 25)	+0·6 (-2·3 to +3·5)***	0·69
- Week 12	71	23 (19 to 27)	68	23 (18 to 27)	-0·3 (-4·3 to +3·6)***	0·87
- Week 18	69	26 (22 to 31)	66	20 (16 to 25)	+5·0 (1·1 to 9·0)***	**0·01**
- Week 24	72	26 (21 to 30)	73	22 (17 to 26)	+3·3 (-1·6 to 8·2)***	0·19

*** Adjusted mean difference from a linear mixed-effects model including treatment group, time point, treatment-by-time interaction, and treatment centre, using observed values as the dependent variable.

Supplementary Table 7. Incidence of adverse events

	**Ferric carboxymaltose**	**Placebo**
**Direct reactions reported within 24 hours after study visit**	Number of visits at which FCM was given = 276	Number of visits at which placebo or no treatment was given= 322 (279 placebo treatments, 43 visits without treatment)
Headache	7 (2·5)	3 (0·9)
Dizziness	2 (0·7)	2 (0·6)
Nausea	2 (0·7)	3 (0·9)
Abdominal pain	7 (2·5)	1 (0·3)
Diarrhea	4 (1·4)	4 (1·2)
Rash	1 (0·4)	1 (0·3)
Local reaction at injection site	1 (0·4)	3 (0·9)
Myalgia	4 (1·4)	3 (0·9)
Fever	1 (0·4)	0
Constipation	1 (0·4)	2 (0·6)
Phlebitis	1 (0·4)	0
Palpitations	1 (0·4)	1 (0·3)
Extreme fatigue	1 (0·4)	3 (0·9)
Epistaxis	1 (0·4)	1 (0·3)

Anaphylaxis	0	0
**Adverse events during six weeks after administration**	**N=77**	**N=77**
Kidney allograft rejection	1 (1·3)	0
Graft failure	0	0
Cardiovascular event	4 (5·2)	1 (1·3)
Fracture	0	3 (3·9)
Hospital admission	4 (5·2)	10 (13·0)
Epistaxis	3 (3·9)	1 (1·3)
New cancer diagnosis	1 (1·3)	3 (3·9)
Infection	48 (62·3)	61 (79·2)
- Urinary tract infection	15 (19·5)	10 (13·0)
- Upper respiratory tract infection	25 (32·5)	34 (44·2)
- Pneumonia	1 (1·3)	1 (1·3)
- Cellulitis	1 (1·3)	0
- Gastro-enteritis	4 (5·2)	7 (9·1)
- Other infection	5 (6·5)	10 (13·0)
Death	2 (2·6)	0
**Adverse laboratory results at following study visit**	**N=77**	**N=77**

Mild hypophosphataemia (0·35 – 0·50 mmol/L)	12 (15·6)	6 (7·8)
Severe hypophosphataemia (<0·35 mmol/L)	1 (1·3)	2 (2·6)

Syntax

* Long to wide transformation*

```
RECODE redcap_event_name ('followup_6_weeks_arm_1'='v6')
('prescreening_arm_1'='prescreening')
   ('baseline_arm_1'='v0') ('screening_arm_1'='vscr') ('followup_12_weeks_arm_1'='v12')
   ('followup_18_weeks_arm_1'='v18') ('end_of_study_arm_1'='v24').
EXECUTE.

SORT CASES BY study_id redcap_event_name.
CASESTOVARS
  /ID=study_id
  /INDEX=redcap_event_name
  /GROUPBY=VARIABLE.
```

** Urine markers calculation and selection based on completeness**

```
IF  (urine_complete_baseline = 1) Urine_creat_excr.baseline=urine_creatinine_baseline *
   (urine_volume_baseline / 1000).
EXECUTE.

IF  (urine_complete_t6 = 1) Urine_creat_excr.t6=urine_creatinine_t6 *
   (urine_volume_t6 / 1000).
EXECUTE.

IF  (urine_complete_t12 = 1) Urine_creat_excr.t12=urine_creatinine_t12 *
   (urine_volume_t12 / 1000).
```

```
EXECUTE.

IF  (urine_complete_t18 = 1) Urine_creat_excr.t18=urine_creatinine_t18 *
    (urine_volume_t18 / 1000).
EXECUTE.

IF  (urine_complete_eos = 1) Urine_creat_excr.eos=urine_creatinine_eos *
    (urine_volume_eos / 1000).
EXECUTE.


IF  (urine_complete_baseline = 1) Urine_phosphate_excr.baseline=urine_phosphate_baseline
*
    (urine_volume_baseline / 1000).
EXECUTE.

IF  (urine_complete_t6 = 1) Urine_phosphate_excr.t6=urine_phosphate_t6 *
    (urine_volume_t6 / 1000).
EXECUTE.

IF  (urine_complete_t12 = 1) Urine_phosphate_excr.t12=urine_phosphate_t12 *
    (urine_volume_t12 / 1000).
EXECUTE.

IF  (urine_complete_t18 = 1) Urine_phosphate_excr.t18=urine_phosphate_t18 *
    (urine_volume_t18 / 1000).
EXECUTE.

IF  (urine_complete_eos = 1) Urine_phosphate_excr.eos=urine_phosphate_eos *
```

```
    (urine_volume_eos / 1000).
EXECUTE.



IF  (urine_complete_baseline = 1) Urine_calcium_excr.baseline=urine_calcium_baseline *
    (urine_volume_baseline / 1000).
EXECUTE.

IF  (urine_complete_t6 = 1) Urine_calcium_excr.t6=urine_calcium_t6 *
    (urine_volume_t6 / 1000).
EXECUTE.

IF  (urine_complete_t12 = 1) Urine_calcium_excr.t12=urine_calcium_t12 *
    (urine_volume_t12 / 1000).
EXECUTE.

IF  (urine_complete_t18 = 1) Urine_calcium_excr.t18=urine_calcium_t18 *
    (urine_volume_t18 / 1000).
EXECUTE.

IF  (urine_complete_eos = 1) Urine_calcium_excr.eos=urine_calcium_eos *
    (urine_volume_eos / 1000).
EXECUTE.



IF  (urine_complete_baseline = 1) Urine_ngal_excr.baseline=lab_urine_ngal.v0 *
    (urine_volume_baseline / 1000).
EXECUTE.
```

```
IF  (urine_complete_t6 = 1) Urine_ngal_excr.t6=lab_urine_ngal.v6 *
    (urine_volume_t6 / 1000).
EXECUTE.

IF  (urine_complete_t12 = 1) Urine_ngal_excr.t12=lab_urine_ngal.v12 *
    (urine_volume_t12 / 1000).
EXECUTE.

IF  (urine_complete_t18 = 1) Urine_ngal_excr.t18=lab_urine_ngal.v18 *
    (urine_volume_t18 / 1000).
EXECUTE.

IF  (urine_complete_eos = 1) Urine_ngal_excr.eos=lab_urine_ngal.v24 *
    (urine_volume_eos / 1000).
EXECUTE.



IF  (urine_complete_baseline = 1) Urine_kim1_excr.baseline=lab_urine_kim1.v0 *
    (urine_volume_baseline / 1000).
EXECUTE.

IF  (urine_complete_t6 = 1) Urine_kim1_excr.t6=lab_urine_kim1.v6 *
    (urine_volume_t6 / 1000).
EXECUTE.

IF  (urine_complete_t12 = 1) Urine_kim1_excr.t12=lab_urine_kim1.v12 *
    (urine_volume_t12 / 1000).
EXECUTE.
```

```
IF  (urine_complete_t18 = 1) Urine_kim1_excr.t18=lab_urine_kim1.v18 *
    (urine_volume_t18 / 1000).
EXECUTE.

IF  (urine_complete_eos = 1) Urine_kim1_excr.eos=lab_urine_kim1.v24 *
    (urine_volume_eos / 1000).
EXECUTE.

IF  (urine_complete_baseline = 1) Urine_eiwit_excr.baseline=lab_urine_protein.v0 *
    (urine_volume_baseline / 1000).
EXECUTE.

IF  (urine_complete_t6 = 1) Urine_eiwit_excr.t6=lab_urine_protein.v6 *
    (urine_volume_t6 / 1000).
EXECUTE.

IF  (urine_complete_t12 = 1) Urine_eiwit_excr.t12=lab_urine_protein.v12 *
    (urine_volume_t12 / 1000).
EXECUTE.

IF  (urine_complete_t18 = 1) Urine_eiwit_excr.t18=lab_urine_protein.v18 *
    (urine_volume_t18 / 1000).
EXECUTE.

IF  (urine_complete_eos = 1) Urine_eiwit_excr.eos=lab_urine_protein.v24 *
    (urine_volume_eos / 1000).
EXECUTE.
```

```
IF  (urine_complete_baseline = 1) Urine_albumin_excr.baseline=lab_urine_albumin.v0 *
    (urine_volume_baseline / 1000).
EXECUTE.

IF  (urine_complete_t6 = 1) Urine_albumin_excr.t6=lab_urine_albumin.v6 *
    (urine_volume_t6 / 1000).
EXECUTE.

IF  (urine_complete_t12 = 1) Urine_albumin_excr.t12=lab_urine_albumin.v12 *
    (urine_volume_t12 / 1000).
EXECUTE.

IF  (urine_complete_t18 = 1) Urine_albumin_excr.t18=lab_urine_albumin.v18 *
    (urine_volume_t18 / 1000).
EXECUTE.

IF  (urine_complete_eos = 1) Urine_albumin_excr.eos=lab_urine_albumin.v24 *
    (urine_volume_eos / 1000).
EXECUTE.



    IF  (urine_complete_baseline = 1) Fractional_sodium_excr.baseline=((lab_creat_baseline /
88.4)  * lab_urine_sodium.v0 * 100) / (lab_natrium_baseline * (urine_creatinine_baseline /
0.0884)).
EXECUTE.

    IF  (urine_complete_t6 = 1) Fractional_sodium_excr.t6=((lab_creat_t6 / 88.4)  *
lab_urine_sodium.v6 * 100) / (lab_natrium_t6 * (urine_creatinine_t6 / 0.0884)).
EXECUTE.
```

```
IF (urine_complete_t12 = 1) Fractional_sodium_excr.t12=((lab_creat_t12 / 88.4) * lab_urine_sodium.v12 * 100) / (lab_natrium_t12 * (urine_creatinine_t12 / 0.0884)).
EXECUTE.

IF (urine_complete_t18 = 1) Fractional_sodium_excr.t18=((lab_creat_t18 / 88.4) * lab_urine_sodium.v18 * 100) / (lab_natrium_t18 * (urine_creatinine_t18 / 0.0884)).
EXECUTE.

IF (urine_complete_eos = 1) Fractional_sodium_excr.eos=((lab_creat_eos / 88.4) * lab_urine_sodium.v24 * 100) / (lab_natrium_eos * (urine_creatinine_eos / 0.0884)).
EXECUTE.

****Calculated variables****

*eGFR (CKD-EPI creatinine age sex 2021)*

COMPUTE lab_creat_mgdl_baseline=lab_creat_baseline / 88.4.
EXECUTE.

COMPUTE lab_creat_mgdl_t6=lab_creat_t6 / 88.4.
EXECUTE.

COMPUTE lab_creat_mgdl_t12=lab_creat_t12 / 88.4.
EXECUTE.

COMPUTE lab_creat_mgdl_t18=lab_creat_t18 / 88.4.
```

```
EXECUTE.

COMPUTE lab_creat_mgdl_eos=lab_creat_eos / 88.4.
EXECUTE.

IF  (gender = 2) lab_scrkratio_baseline=lab_creat_mgdl_baseline / 0.7.
EXECUTE.

IF  (gender = 1) lab_scrkratio_baseline=lab_creat_mgdl_baseline / 0.9.
EXECUTE.

IF  (gender = 2) lab_scrkratio_t6=lab_creat_mgdl_t6 / 0.7.
EXECUTE.

IF  (gender = 1) lab_scrkratio_t6=lab_creat_mgdl_t6 / 0.9.
EXECUTE.

IF  (gender = 2) lab_scrkratio_t12=lab_creat_mgdl_t12 / 0.7.
EXECUTE.

IF  (gender = 1) lab_scrkratio_t12=lab_creat_mgdl_t12 / 0.9.
EXECUTE.

IF  (gender = 2) lab_scrkratio_t18=lab_creat_mgdl_t18 / 0.7.
EXECUTE.

IF  (gender = 1) lab_scrkratio_t18=lab_creat_mgdl_t18 / 0.9.
EXECUTE.
```

```
IF  (gender = 2) lab_scrkratio_eos=lab_creat_mgdl_eos / 0.7.
EXECUTE.

IF  (gender = 1) lab_scrkratio_eos=lab_creat_mgdl_eos / 0.9.
EXECUTE.



IF  ((gender = 2) & (lab_scrkratio_baseline <= 1)) lab_egfr_ckdepi2021cras_baseline=142 *
    (lab_scrkratio_baseline**(-0.241)) * (1**(-1.2)) * (0.9938**(age_baseline)) * 1.012.
EXECUTE.

IF  ((gender = 2) & (lab_scrkratio_baseline  > 1)) lab_egfr_ckdepi2021cras_baseline=142 *
    (1**(-0.241)) * (lab_scrkratio_baseline**(-1.2)) * (0.9938**(age_baseline)) * 1.012.
EXECUTE.

IF  ((gender = 1) & (lab_scrkratio_baseline   <= 1)) lab_egfr_ckdepi2021cras_baseline=142 *
    (lab_scrkratio_baseline**(-0.302)) * (1**(-1.2)) * (0.9938**(age_baseline)).
EXECUTE.

IF  ((gender = 1) & (lab_scrkratio_baseline > 1)) lab_egfr_ckdepi2021cras_baseline=142 *
    (1**(-0.302)) * (lab_scrkratio_baseline**(-1.2)) * (0.9938**(age_baseline)).
EXECUTE.



IF  ((gender = 2) & (lab_scrkratio_t6 <= 1)) lab_egfr_ckdepi2021cras_t6=142 *
    (lab_scrkratio_t6**(-0.241)) * (1**(-1.2)) * (0.9938**(age_baseline)) * 1.012.
EXECUTE.
```

```
IF  ((gender = 2) & (lab_scrkratio_t6  > 1)) lab_egfr_ckdepi2021cras_t6=142 *
    (1**(-0.241)) * (lab_scrkratio_t6**(-1.2)) * (0.9938**(age_baseline)) * 1.012.
EXECUTE.

IF  ((gender = 1) & (lab_scrkratio_t6  <= 1)) lab_egfr_ckdepi2021cras_t6=142 *
    (lab_scrkratio_t6**(-0.302)) * (1**(-1.2)) * (0.9938**(age_baseline)).
EXECUTE.

IF  ((gender = 1) & (lab_scrkratio_t6 > 1)) lab_egfr_ckdepi2021cras_t6=142 *
    (1**(-0.302)) * (lab_scrkratio_t6**(-1.2)) * (0.9938**(age_baseline)).
EXECUTE.



IF  ((gender = 2) & (lab_scrkratio_t12 <= 1)) lab_egfr_ckdepi2021cras_t12=142 *
    (lab_scrkratio_t12**(-0.241)) * (1**(-1.2)) * (0.9938**(age_baseline)) * 1.012.
EXECUTE.

IF  ((gender = 2) & (lab_scrkratio_t12  > 1)) lab_egfr_ckdepi2021cras_t12=142 *
    (1**(-0.241)) * (lab_scrkratio_t12**(-1.2)) * (0.9938**(age_baseline)) * 1.012.
EXECUTE.

IF  ((gender = 1) & (lab_scrkratio_t12  <= 1)) lab_egfr_ckdepi2021cras_t12=142 *
    (lab_scrkratio_t12**(-0.302)) * (1**(-1.2)) * (0.9938**(age_baseline)).
EXECUTE.

IF  ((gender = 1) & (lab_scrkratio_t12 > 1)) lab_egfr_ckdepi2021cras_t12=142 *
    (1**(-0.302)) * (lab_scrkratio_t12**(-1.2)) * (0.9938**(age_baseline)).
```

```
EXECUTE.

IF  ((gender = 2) & (lab_scrkratio_t18 <= 1)) lab_egfr_ckdepi2021cras_t18=142 *
   (lab_scrkratio_t18**(-0.241)) * (1**(-1.2)) * (0.9938**(age_baseline)) * 1.012.
EXECUTE.

IF  ((gender = 2) & (lab_scrkratio_t18  > 1)) lab_egfr_ckdepi2021cras_t18=142 *
   (1**(-0.241)) * (lab_scrkratio_t18**(-1.2)) * (0.9938**(age_baseline)) * 1.012.
EXECUTE.

IF  ((gender = 1) & (lab_scrkratio_t18 <= 1)) lab_egfr_ckdepi2021cras_t18=142 *
   (lab_scrkratio_t18**(-0.302)) * (1**(-1.2)) * (0.9938**(age_baseline)).
EXECUTE.

IF  ((gender = 1) & (lab_scrkratio_t18 > 1)) lab_egfr_ckdepi2021cras_t18=142 *
   (1**(-0.302)) * (lab_scrkratio_t18**(-1.2)) * (0.9938**(age_baseline)).
EXECUTE.

IF  ((gender = 2) & (lab_scrkratio_eos <= 1)) lab_egfr_ckdepi2021cras_eos=142 *
   (lab_scrkratio_eos**(-0.241)) * (1**(-1.2)) * (0.9938**(age_baseline)) * 1.012.
EXECUTE.

IF  ((gender = 2) & (lab_scrkratio_eos  > 1)) lab_egfr_ckdepi2021cras_eos=142 *
   (1**(-0.241)) * (lab_scrkratio_eos**(-1.2)) * (0.9938**(age_baseline)) * 1.012.
```

```
EXECUTE.

IF  ((gender = 1) & (lab_scrkratio_eos <= 1)) lab_egfr_ckdepi2021cras_eos=142 *
    (lab_scrkratio_eos**(-0.302)) * (1**(-1.2)) * (0.9938**(age_baseline)).
EXECUTE.

IF  ((gender = 1) & (lab_scrkratio_eos > 1)) lab_egfr_ckdepi2021cras_eos=142 *
    (1**(-0.302)) * (lab_scrkratio_eos**(-1.2)) * (0.9938**(age_baseline)).
EXECUTE.


*The CKD-EPI Creatinine-Cystatin C Age, Sex Equation (2021)*

COMPUTE lab_cystatincratio.v0=lab_cystatinc.v0 / 0.8.
EXECUTE.

COMPUTE lab_cystatincratio.v6=lab_cystatinc.v6 / 0.8.
EXECUTE.

COMPUTE lab_cystatincratio.v12=lab_cystatinc.v12 / 0.8.
EXECUTE.

COMPUTE lab_cystatincratio.v18=lab_cystatinc.v18 / 0.8.
EXECUTE.

COMPUTE lab_cystatincratio.v24=lab_cystatinc.v24 / 0.8.
EXECUTE.
```

```
IF  ((gender = 2) & (lab_scrkratio_baseline <= 1) & (lab_cystatincratio.v0 <= 1))
lab_egfr_ckdepi2021crcysc_baseline=135 *
   (lab_scrkratio_baseline**(-0.219)) * (1**(-0.544)) *
   (lab_cystatincratio.v0**(-0.323)) * (1**(-0.778)) *
   (0.9961**(age_baseline)) * (0.963).
EXECUTE.

IF  ((gender = 2) & (lab_scrkratio_baseline <= 1) & (lab_cystatincratio.v0 > 1))
lab_egfr_ckdepi2021crcysc_baseline=135 *
   (lab_scrkratio_baseline**(-0.219)) * (1**(-0.544)) *
   (1**(-0.323)) * (lab_cystatincratio.v0**(-0.778)) *
   (0.9961**(age_baseline)) * (0.963).
EXECUTE.

IF  ((gender = 2) & (lab_scrkratio_baseline > 1) & (lab_cystatincratio.v0 <= 1))
lab_egfr_ckdepi2021crcysc_baseline=135 *
   (1**(-0.219)) * (lab_scrkratio_baseline**(-0.544)) *
   (lab_cystatincratio.v0**(-0.323)) * (1**(-0.778)) *
   (0.9961**(age_baseline)) * (0.963).
EXECUTE.

IF  ((gender = 2) & (lab_scrkratio_baseline > 1) & (lab_cystatincratio.v0 > 1))
lab_egfr_ckdepi2021crcysc_baseline=135 *
   (1**(-0.219)) * (lab_scrkratio_baseline**(-0.544)) *
   (1**(-0.323)) * (lab_cystatincratio.v0**(-0.778)) *
   (0.9961**(age_baseline)) * (0.963).
EXECUTE.

IF  ((gender = 1) & (lab_scrkratio_baseline <= 1) & (lab_cystatincratio.v0 <= 1))
lab_egfr_ckdepi2021crcysc_baseline=135 *
```

```
    (lab_scrkratio_baseline**(-0.144)) * (1**(-0.544)) *
    (lab_cystatincratio.v0**(-0.323)) * (1**(-0.778)) *
    (0.9961**(age_baseline)).
EXECUTE.

IF  ((gender = 1) & (lab_scrkratio_baseline <= 1) & (lab_cystatincratio.v0 > 1))
lab_egfr_ckdepi2021crcysc_baseline=135 *
    (lab_scrkratio_baseline**(-0.144)) * (1**(-0.544)) *
    (1**(-0.323)) * (lab_cystatincratio.v0**(-0.778)) *
    (0.9961**(age_baseline)) .
EXECUTE.

IF  ((gender = 1) & (lab_scrkratio_baseline > 1) & (lab_cystatincratio.v0 <= 1))
lab_egfr_ckdepi2021crcysc_baseline=135 *
    (1**(-0.144)) * (lab_scrkratio_baseline**(-0.544)) *
    (lab_cystatincratio.v0**(-0.323)) * (1**(-0.778)) *
    (0.9961**(age_baseline)) .
EXECUTE.

IF  ((gender = 1) & (lab_scrkratio_baseline > 1) & (lab_cystatincratio.v0 > 1))
lab_egfr_ckdepi2021crcysc_baseline=135 *
    (1**(-0.144)) * (lab_scrkratio_baseline**(-0.544)) *
    (1**(-0.323)) * (lab_cystatincratio.v0**(-0.778)) *
    (0.9961**(age_baseline)) .
EXECUTE.

**

    IF  ((gender = 2) & (lab_scrkratio_t6 <= 1) & (lab_cystatincratio.v6 <= 1))
lab_egfr_ckdepi2021crcysc_t6=135 *
```

```
    (lab_scrkratio_t6**(-0.219)) * (1**(-0.544)) *
    (lab_cystatincratio.v6**(-0.323)) * (1**(-0.778)) *
    (0.9961**(age_baseline)) * (0.963).
EXECUTE.

IF  ((gender = 2) & (lab_scrkratio_t6 <= 1) & (lab_cystatincratio.v6 > 1))
lab_egfr_ckdepi2021crcysc_t6=135 *
    (lab_scrkratio_t6**(-0.219)) * (1**(-0.544)) *
    (1**(-0.323)) * (lab_cystatincratio.v6**(-0.778)) *
    (0.9961**(age_baseline)) * (0.963).
EXECUTE.

IF  ((gender = 2) & (lab_scrkratio_t6 > 1) & (lab_cystatincratio.v6 <= 1))
lab_egfr_ckdepi2021crcysc_t6=135 *
    (1**(-0.219)) * (lab_scrkratio_t6**(-0.544)) *
    (lab_cystatincratio.v6**(-0.323)) * (1**(-0.778)) *
    (0.9961**(age_baseline)) * (0.963).
EXECUTE.

IF  ((gender = 2) & (lab_scrkratio_t6 > 1) & (lab_cystatincratio.v6 > 1))
lab_egfr_ckdepi2021crcysc_t6=135 *
    (1**(-0.219)) * (lab_scrkratio_t6**(-0.544)) *
    (1**(-0.323)) * (lab_cystatincratio.v6**(-0.778)) *
    (0.9961**(age_baseline)) * (0.963).
EXECUTE.

IF  ((gender = 1) & (lab_scrkratio_t6 <= 1) & (lab_cystatincratio.v6 <= 1))
lab_egfr_ckdepi2021crcysc_t6=135 *
    (lab_scrkratio_t6**(-0.144)) * (1**(-0.544)) *
    (lab_cystatincratio.v6**(-0.323)) * (1**(-0.778)) *
```

```
    (0.9961**(age_baseline)).
EXECUTE.


IF  ((gender = 1) & (lab_scrkratio_t6 <= 1) & (lab_cystatincratio.v6 > 1))
lab_egfr_ckdepi2021crcysc_t6=135 *
    (lab_scrkratio_t6**(-0.144)) * (1**(-0.544)) *
    (1**(-0.323)) * (lab_cystatincratio.v6**(-0.778)) *
    (0.9961**(age_baseline)) .
EXECUTE.


IF  ((gender = 1) & (lab_scrkratio_t6 > 1) & (lab_cystatincratio.v6 <= 1))
lab_egfr_ckdepi2021crcysc_t6=135 *
    (1**(-0.144)) * (lab_scrkratio_t6**(-0.544)) *
    (lab_cystatincratio.v6**(-0.323)) * (1**(-0.778)) *
    (0.9961**(age_baseline)) .
EXECUTE.


IF  ((gender = 1) & (lab_scrkratio_t6 > 1) & (lab_cystatincratio.v6 > 1))
lab_egfr_ckdepi2021crcysc_t6=135 *
    (1**(-0.144)) * (lab_scrkratio_t6**(-0.544)) *
    (1**(-0.323)) * (lab_cystatincratio.v6**(-0.778)) *
    (0.9961**(age_baseline)) .
EXECUTE.


**


IF  ((gender = 2) & (lab_scrkratio_t12 <= 1) & (lab_cystatincratio.v12 <= 1))
lab_egfr_ckdepi2021crcysc_t12=135 *
    (lab_scrkratio_t12**(-0.219)) * (1**(-0.544)) *
    (lab_cystatincratio.v12**(-0.323)) * (1**(-0.778)) *
```

```
    (0.9961**(age_baseline)) * (0.963).
EXECUTE.


IF  ((gender = 2) & (lab_scrkratio_t12 <= 1) & (lab_cystatincratio.v12 > 1))
lab_egfr_ckdepi2021crcysc_t12=135 *
    (lab_scrkratio_t12**(-0.219)) * (1**(-0.544)) *
    (1**(-0.323)) * (lab_cystatincratio.v12**(-0.778)) *
    (0.9961**(age_baseline)) * (0.963).
EXECUTE.


IF  ((gender = 2) & (lab_scrkratio_t12 > 1) & (lab_cystatincratio.v12 <= 1))
lab_egfr_ckdepi2021crcysc_t12=135 *
    (1**(-0.219)) * (lab_scrkratio_t12**(-0.544)) *
    (lab_cystatincratio.v12**(-0.323)) * (1**(-0.778)) *
    (0.9961**(age_baseline)) * (0.963).
EXECUTE.


IF  ((gender = 2) & (lab_scrkratio_t12 > 1) & (lab_cystatincratio.v12 > 1))
lab_egfr_ckdepi2021crcysc_t12=135 *
    (1**(-0.219)) * (lab_scrkratio_t12**(-0.544)) *
    (1**(-0.323)) * (lab_cystatincratio.v12**(-0.778)) *
    (0.9961**(age_baseline)) * (0.963).
EXECUTE.


IF  ((gender = 1) & (lab_scrkratio_t12 <= 1) & (lab_cystatincratio.v12 <= 1))
lab_egfr_ckdepi2021crcysc_t12=135 *
    (lab_scrkratio_t12**(-0.144)) * (1**(-0.544)) *
    (lab_cystatincratio.v12**(-0.323)) * (1**(-0.778)) *
    (0.9961**(age_baseline)).
EXECUTE.
```

```
IF  ((gender = 1) & (lab_scrkratio_t12 <= 1) & (lab_cystatincratio.v12 > 1))
lab_egfr_ckdepi2021crcysc_t12=135 *
    (lab_scrkratio_t12**(-0.144)) * (1**(-0.544)) *
    (1**(-0.323)) * (lab_cystatincratio.v12**(-0.778)) *
    (0.9961**(age_baseline)) .
EXECUTE.


IF  ((gender = 1) & (lab_scrkratio_t12 > 1) & (lab_cystatincratio.v12 <= 1))
lab_egfr_ckdepi2021crcysc_t12=135 *
    (1**(-0.144)) * (lab_scrkratio_t12**(-0.544)) *
    (lab_cystatincratio.v12**(-0.323)) * (1**(-0.778)) *
    (0.9961**(age_baseline)) .
EXECUTE.


IF  ((gender = 1) & (lab_scrkratio_t12 > 1) & (lab_cystatincratio.v12 > 1))
lab_egfr_ckdepi2021crcysc_t12=135 *
    (1**(-0.144)) * (lab_scrkratio_t12**(-0.544)) *
    (1**(-0.323)) * (lab_cystatincratio.v12**(-0.778)) *
    (0.9961**(age_baseline)) .
EXECUTE.


**


IF  ((gender = 2) & (lab_scrkratio_t18 <= 1) & (lab_cystatincratio.v18 <= 1))
lab_egfr_ckdepi2021crcysc_t18=135 *
    (lab_scrkratio_t18**(-0.219)) * (1**(-0.544)) *
    (lab_cystatincratio.v18**(-0.323)) * (1**(-0.778)) *
    (0.9961**(age_baseline)) * (0.963).
EXECUTE.
```

```
IF  ((gender = 2) & (lab_scrkratio_t18 <= 1) & (lab_cystatincratio.v18 > 1))
lab_egfr_ckdepi2021crcysc_t18=135 *
   (lab_scrkratio_t18**(-0.219)) * (1**(-0.544)) *
   (1**(-0.323)) * (lab_cystatincratio.v18**(-0.778)) *
   (0.9961**(age_baseline)) * (0.963).
EXECUTE.

IF  ((gender = 2) & (lab_scrkratio_t18 > 1) & (lab_cystatincratio.v18 <= 1))
lab_egfr_ckdepi2021crcysc_t18=135 *
   (1**(-0.219)) * (lab_scrkratio_t18**(-0.544)) *
   (lab_cystatincratio.v18**(-0.323)) * (1**(-0.778)) *
   (0.9961**(age_baseline)) * (0.963).
EXECUTE.

IF  ((gender = 2) & (lab_scrkratio_t18 > 1) & (lab_cystatincratio.v18 > 1))
lab_egfr_ckdepi2021crcysc_t18=135 *
   (1**(-0.219)) * (lab_scrkratio_t18**(-0.544)) *
   (1**(-0.323)) * (lab_cystatincratio.v18**(-0.778)) *
   (0.9961**(age_baseline)) * (0.963).
EXECUTE.

IF  ((gender = 1) & (lab_scrkratio_t18 <= 1) & (lab_cystatincratio.v18 <= 1))
lab_egfr_ckdepi2021crcysc_t18=135 *
   (lab_scrkratio_t18**(-0.144)) * (1**(-0.544)) *
   (lab_cystatincratio.v18**(-0.323)) * (1**(-0.778)) *
   (0.9961**(age_baseline)).
EXECUTE.

IF  ((gender = 1) & (lab_scrkratio_t18 <= 1) & (lab_cystatincratio.v18 > 1))
lab_egfr_ckdepi2021crcysc_t18=135 *
```

```
    (lab_scrkratio_t18**(-0.144)) * (1**(-0.544)) *
    (1**(-0.323)) * (lab_cystatincratio.v18**(-0.778)) *
    (0.9961**(age_baseline)) .
EXECUTE.

IF  ((gender = 1) & (lab_scrkratio_t18 > 1) & (lab_cystatincratio.v18 <= 1))
lab_egfr_ckdepi2021crcysc_t18=135 *
    (1**(-0.144)) * (lab_scrkratio_t18**(-0.544)) *
    (lab_cystatincratio.v18**(-0.323)) * (1**(-0.778)) *
    (0.9961**(age_baseline)) .
EXECUTE.

IF  ((gender = 1) & (lab_scrkratio_t18 > 1) & (lab_cystatincratio.v18 > 1))
lab_egfr_ckdepi2021crcysc_t18=135 *
    (1**(-0.144)) * (lab_scrkratio_t18**(-0.544)) *
    (1**(-0.323)) * (lab_cystatincratio.v18**(-0.778)) *
    (0.9961**(age_baseline)) .
EXECUTE.

**

IF  ((gender = 2) & (lab_scrkratio_eos <= 1) & (lab_cystatincratio.v24 <= 1))
lab_egfr_ckdepi2021crcysc_eos=135 *
    (lab_scrkratio_eos**(-0.219)) * (1**(-0.544)) *
    (lab_cystatincratio.v24**(-0.323)) * (1**(-0.778)) *
    (0.9961**(age_baseline)) * (0.963).
EXECUTE.

IF  ((gender = 2) & (lab_scrkratio_eos <= 1) & (lab_cystatincratio.v24 > 1))
lab_egfr_ckdepi2021crcysc_eos=135 *
```

```
    (lab_scrkratio_eos**(-0.219)) * (1**(-0.544)) *
    (1**(-0.323)) * (lab_cystatincratio.v24**(-0.778)) *
    (0.9961**(age_baseline)) * (0.963).
EXECUTE.

IF  ((gender = 2) & (lab_scrkratio_eos > 1) & (lab_cystatincratio.v24 <= 1))
lab_egfr_ckdepi2021crcysc_eos=135 *
    (1**(-0.219)) * (lab_scrkratio_eos**(-0.544)) *
    (lab_cystatincratio.v24**(-0.323)) * (1**(-0.778)) *
    (0.9961**(age_baseline)) * (0.963).
EXECUTE.

IF  ((gender = 2) & (lab_scrkratio_eos > 1) & (lab_cystatincratio.v24 > 1))
lab_egfr_ckdepi2021crcysc_eos=135 *
    (1**(-0.219)) * (lab_scrkratio_eos**(-0.544)) *
    (1**(-0.323)) * (lab_cystatincratio.v24**(-0.778)) *
    (0.9961**(age_baseline)) * (0.963).
EXECUTE.

IF  ((gender = 1) & (lab_scrkratio_eos <= 1) & (lab_cystatincratio.v24 <= 1))
lab_egfr_ckdepi2021crcysc_eos=135 *
    (lab_scrkratio_eos**(-0.144)) * (1**(-0.544)) *
    (lab_cystatincratio.v24**(-0.323)) * (1**(-0.778)) *
    (0.9961**(age_baseline)).
EXECUTE.

IF  ((gender = 1) & (lab_scrkratio_eos <= 1) & (lab_cystatincratio.v24 > 1))
lab_egfr_ckdepi2021crcysc_eos=135 *
    (lab_scrkratio_eos**(-0.144)) * (1**(-0.544)) *
    (1**(-0.323)) * (lab_cystatincratio.v24**(-0.778)) *
```

(0.9961**(age_baseline)) .

EXECUTE.

IF ((gender = 1) & (lab_scrkratio_eos > 1) & (lab_cystatincratio.v24 <= 1))
lab_egfr_ckdepi2021crcysc_eos=135 *

(1**(-0.144)) * (lab_scrkratio_eos**(-0.544)) *

(lab_cystatincratio.v24**(-0.323)) * (1**(-0.778)) *

(0.9961**(age_baseline)) .

EXECUTE.

IF ((gender = 1) & (lab_scrkratio_eos > 1) & (lab_cystatincratio.v24 > 1))
lab_egfr_ckdepi2021crcysc_eos=135 *

(1**(-0.144)) * (lab_scrkratio_eos**(-0.544)) *

(1**(-0.323)) * (lab_cystatincratio.v24**(-0.778)) *

(0.9961**(age_baseline)) .

EXECUTE.

The CKD-EPI Cystatin C Age, Sex Equation (2012)

The CKD-EPI Cystatin C Age, Sex Equation (2012), that we developed previously,[2]

can be expressed as a single equation

133 X min(Scys/0.8,1)-0.499 X max(Scys/0.8,1)-1.328 X 0.9962

age X 0.932 [if female] where Scys is serum cystatin C

IF ((gender = 2) & (lab_cystatincratio.v0 <= 1)) lab_egfr_ckdepi2012cysc_baseline=133 *

(lab_cystatincratio.v0**(-0.499)) * (1**(-1.328)) *

(0.9962**(age_baseline)) * (0.932).

EXECUTE.

```
IF  ((gender = 2) & (lab_cystatincratio.v0 > 1)) lab_egfr_ckdepi2012cysc_baseline=133 *
   (1**(-0.499)) * (lab_cystatincratio.v0**(-1.328)) *
   (0.9962**(age_baseline)) * (0.932).
EXECUTE.


IF  ((gender = 1) & (lab_cystatincratio.v0 <= 1)) lab_egfr_ckdepi2012cysc_baseline=133 *
   (lab_cystatincratio.v0**(-0.499)) * (1**(-1.328)) *
   (0.9962**(age_baseline)).
EXECUTE.

IF  ((gender = 1) & (lab_cystatincratio.v0 > 1)) lab_egfr_ckdepi2012cysc_baseline=133 *
   (1**(-0.499)) * (lab_cystatincratio.v0**(-1.328)) *
   (0.9962**(age_baseline)).
EXECUTE.

IF  ((gender = 2) & (lab_cystatincratio.v6 <= 1)) lab_egfr_ckdepi2012cysc_v6=133 *
   (lab_cystatincratio.v6**(-0.499)) * (1**(-1.328)) *
   (0.9962**(age_baseline)) * (0.932).
EXECUTE.

IF  ((gender = 2) & (lab_cystatincratio.v6 > 1)) lab_egfr_ckdepi2012cysc_v6=133 *
   (1**(-0.499)) * (lab_cystatincratio.v6**(-1.328)) *
   (0.9962**(age_baseline)) * (0.932).
EXECUTE.


IF  ((gender = 1) & (lab_cystatincratio.v6 <= 1)) lab_egfr_ckdepi2012cysc_v6=133 *
   (lab_cystatincratio.v6**(-0.499)) * (1**(-1.328)) *
```

```
   (0.9962**(age_baseline)).
EXECUTE.

IF  ((gender = 1) & (lab_cystatincratio.v6 > 1)) lab_egfr_ckdepi2012cysc_v6=133 *
   (1**(-0.499)) * (lab_cystatincratio.v6**(-1.328)) *
   (0.9962**(age_baseline)).
EXECUTE.


IF  ((gender = 2) & (lab_cystatincratio.v12 <= 1)) lab_egfr_ckdepi2012cysc_v12=133 *
   (lab_cystatincratio.v12**(-0.499)) * (1**(-1.328)) *
   (0.9962**(age_baseline)) * (0.932).
EXECUTE.

IF  ((gender = 2) & (lab_cystatincratio.v12 > 1)) lab_egfr_ckdepi2012cysc_v12=133 *
   (1**(-0.499)) * (lab_cystatincratio.v12**(-1.328)) *
   (0.9962**(age_baseline)) * (0.932).
EXECUTE.


IF  ((gender = 1) & (lab_cystatincratio.v12 <= 1)) lab_egfr_ckdepi2012cysc_v12=133 *
   (lab_cystatincratio.v12**(-0.499)) * (1**(-1.328)) *
   (0.9962**(age_baseline)).
EXECUTE.

IF  ((gender = 1) & (lab_cystatincratio.v12 > 1)) lab_egfr_ckdepi2012cysc_v12=133 *
   (1**(-0.499)) * (lab_cystatincratio.v12**(-1.328)) *
   (0.9962**(age_baseline)).
EXECUTE.
```

```
IF  ((gender = 2) & (lab_cystatincratio.v18 <= 1)) lab_egfr_ckdepi2012cysc_v18=133 *
   (lab_cystatincratio.v18**(-0.499)) * (1**(-1.328)) *
   (0.9962**(age_baseline)) * (0.932).
EXECUTE.

IF  ((gender = 2) & (lab_cystatincratio.v18 > 1)) lab_egfr_ckdepi2012cysc_v18=133 *
   (1**(-0.499)) * (lab_cystatincratio.v18**(-1.328)) *
   (0.9962**(age_baseline)) * (0.932).
EXECUTE.


IF  ((gender = 1) & (lab_cystatincratio.v18 <= 1)) lab_egfr_ckdepi2012cysc_v18=133 *
   (lab_cystatincratio.v18**(-0.499)) * (1**(-1.328)) *
   (0.9962**(age_baseline)).
EXECUTE.

IF  ((gender = 1) & (lab_cystatincratio.v18 > 1)) lab_egfr_ckdepi2012cysc_v18=133 *
   (1**(-0.499)) * (lab_cystatincratio.v18**(-1.328)) *
   (0.9962**(age_baseline)).
EXECUTE.


IF  ((gender = 2) & (lab_cystatincratio.v24 <= 1)) lab_egfr_ckdepi2012cysc_v24=133 *
   (lab_cystatincratio.v24**(-0.499)) * (1**(-1.328)) *
   (0.9962**(age_baseline)) * (0.932).
EXECUTE.
```

```
IF  ((gender = 2) & (lab_cystatincratio.v24 > 1)) lab_egfr_ckdepi2012cysc_v24=133 *
    (1**(-0.499)) * (lab_cystatincratio.v24**(-1.328)) *
    (0.9962**(age_baseline)) * (0.932).
EXECUTE.


IF  ((gender = 1) & (lab_cystatincratio.v24 <= 1)) lab_egfr_ckdepi2012cysc_v24=133 *
    (lab_cystatincratio.v24**(-0.499)) * (1**(-1.328)) *
    (0.9962**(age_baseline)).
EXECUTE.

IF  ((gender = 1) & (lab_cystatincratio.v24 > 1)) lab_egfr_ckdepi2012cysc_v24=133 *
    (1**(-0.499)) * (lab_cystatincratio.v24**(-1.328)) *
    (0.9962**(age_baseline)).
EXECUTE.


*Creatinine clearance*

IF  (urine_complete_baseline = 1) Urine_creat_clearance.baseline=
    ((1000 * urine_creatinine_baseline) / lab_creat_baseline) * (urine_volume_baseline /
1440).
EXECUTE.

IF  (urine_complete_t6 = 1) Urine_creat_clearance.t6=
    ((1000 * urine_creatinine_t6) / lab_creat_t6) * (urine_volume_t6 / 1440).
EXECUTE.
```

```
IF  (urine_complete_t12 = 1) Urine_creat_clearance.t12=
   ((1000 * urine_creatinine_t12) / lab_creat_t12) * (urine_volume_t12 / 1440).
EXECUTE.

IF  (urine_complete_t18 = 1) Urine_creat_clearance.t18=
   ((1000 * urine_creatinine_t18) / lab_creat_t18) * (urine_volume_t18 / 1440).
EXECUTE.

IF  (urine_complete_eos = 1) Urine_creat_clearance.eos=
   ((1000 * urine_creatinine_eos) / lab_creat_eos) * (urine_volume_eos / 1440).
EXECUTE.



IF  (MISSING(Urine_creat_clearance.t6))
Urine_creat_clearance.t6=Urine_creat_clearance.baseline.
EXECUTE.

IF  (MISSING(Urine_creat_clearance.t12))
Urine_creat_clearance.t12=Urine_creat_clearance.t6.
EXECUTE.

IF  (MISSING(Urine_creat_clearance.t18))
Urine_creat_clearance.t18=Urine_creat_clearance.t12.
EXECUTE.

IF  (MISSING(Urine_creat_clearance.eos))
Urine_creat_clearance.eos=Urine_creat_clearance.t18.
EXECUTE.

*Mean of muscle related outcomes performed in triplo*
```

```
COMPUTE
ftstst_mean_baseline=MEAN(ftstst_score_1.v0,ftstst_score_2.v0,ftstst_score_3.v0).
EXECUTE.

COMPUTE
ftstst_mean_eos=MEAN(ftstst_score_1.v24,ftstst_score_2.v24,ftstst_score_3.v24).
EXECUTE.

COMPUTE tug_mean_baseline=MEAN(tug_score_1.v0,tug_score_2.v0,tug_score_3.v0).
EXECUTE.

COMPUTE tug_mean_eos=MEAN(tug_score_1.v24,tug_score_2.v24,tug_score_3.v24).
EXECUTE.

COMPUTE
hgs_mean_baseline=MEAN(hgst_score_left_1.v0,hgst_score_right_1.v0,hgst_score_left_2.v
0,
   hgst_score_right_2.v0,hgst_score_left_3.v0,hgst_score_right_3.v0).
EXECUTE.

COMPUTE
hgs_mean_eos=MEAN(hgst_score_left_1.v24,hgst_score_right_1.v24,hgst_score_left_2.v24
,
   hgst_score_right_2.v24,hgst_score_left_3.v24,hgst_score_right_3.v24).
EXECUTE.

*Echographic parameter calculations*

COMPUTE bsa_dubois_baseline=(weight ** (0.425)) * ((length * 100) ** (0.725)) *
0.007184.
```

EXECUTE.

COMPUTE bsa_dubois_v6=(weight_fu.v6 ** (0.425)) * ((length * 100) ** (0.725)) * 0.007184.

EXECUTE.

COMPUTE bsa_dubois_v12=(weight_fu.v12 ** (0.425)) * ((length * 100) ** (0.725)) * 0.007184.

EXECUTE.

COMPUTE bsa_dubois_v18=(weight_fu.v18 ** (0.425)) * ((length * 100) ** (0.725)) * 0.007184.

EXECUTE.

COMPUTE bsa_dubois_v24=(weight_fu.v24 ** (0.425)) * ((length * 100) ** (0.725)) * 0.007184.

EXECUTE.

COMPUTE echo_lvef_baseline=MEAN(echo_lvef_measured.v0,echo_lvef_eb_low.v0,echo_lvef_eb_high.v0).

EXECUTE.

COMPUTE echo_lvef_v24=MEAN(echo_lvef_measured.v24,echo_lvef_eb_low.v24,echo_lvef_eb_high.v24).

EXECUTE.

COMPUTE echo_lavi_baseline=echo_lav.v0 / bsa_dubois_baseline.

EXECUTE.

COMPUTE echo_lavi_v24=echo_lav.v24 / bsa_dubois_v24.

```
EXECUTE.

COMPUTE echo_rwt_baseline=(2 * echo_lvpwd.v0) / echo_lvidd.v0.
EXECUTE.

COMPUTE echo_rwt_v24=(2 * echo_lvpwd.v24) / echo_lvidd.v24.
EXECUTE.

COMPUTE echo_lvm_devereux_baseline=0.8 * (1.04 * ((((echo_lvidd.v0 + echo_ivsd.v0 + echo_lvpwd.v0)/10) ** 3) -
   ((echo_lvidd.v0/10) ** 3))) + 0.6.
EXECUTE.

COMPUTE echo_lvm_devereux_v24=0.8 * (1.04 * ((((echo_lvidd.v24 + echo_ivsd.v24 + echo_lvpwd.v24)/10) ** 3) -
   ((echo_lvidd.v24/10) ** 3))) + 0.6.
EXECUTE.

COMPUTE echo_lvmi_devereux_baseline=echo_lvm_devereux_baseline / bsa_dubois_baseline.
EXECUTE.

COMPUTE echo_lvmi_devereux_v24=echo_lvm_devereux_v24 / bsa_dubois_v24.
EXECUTE.

COMPUTE echo_gls.v0=MEAN(echo_gls_a2c.v0,echo_gls_a4c.v0,echo_gls_aplax.v0).
EXECUTE.
```

COMPUTE
echo_gls.v24=MEAN(echo_gls_a2c.v24,echo_gls_a4c.v24,echo_gls_aplax.v24).

EXECUTE.

HFA-PEFF score

RECODE echo_e_septal.v0 (Lowest thru 6.99999=1) (7 thru Highest=0) (MISSING=0) INTO echo_hfapeff_majorfunc_a.v0.

EXECUTE.

RECODE echo_e_lateral.v0 (Lowest thru 9.99999=1) (10 thru Highest=0) (MISSING=0) INTO echo_hfapeff_majorfunc_b.v0.

EXECUTE.

RECODE echo_ratio_ee.v0 (Lowest thru 0.15=0) (0.150001 thru Highest=1) (MISSING=0) INTO echo_hfapeff_majorfunc_c.v0.

EXECUTE.

RECODE echo_strv_annulus.v0 (Lowest thru 2.8=0) (2.80001 thru Highest=1) (MISSING=0) INTO echo_hfapeff_majorfunc_d.v0.

EXECUTE.

COMPUTE echo_hfapeff_majorfunc_sum.v0=echo_hfapeff_majorfunc_a.v0 + echo_hfapeff_majorfunc_b.v0 +

echo_hfapeff_majorfunc_c.v0 + echo_hfapeff_majorfunc_d.v0.

EXECUTE.

RECODE echo_hfapeff_majorfunc_sum.v0 (MISSING=0) (0=0) (ELSE=2) INTO echo_hfapeff_majorfunc.v0.

EXECUTE.

RECODE echo_ratio_ee.v0 (0.09 thru 0.14=1) (ELSE=0) (MISSING=0) INTO echo_hfapeff_minorfunc_a.v0.

EXECUTE.

COMPUTE echo_gls_positive.v0=SQRT(echo_gls.v0 * echo_gls.v0).

EXECUTE.

RECODE echo_gls_positive.v0 (0.00 thru 15.99=1) (16.00 thru Highest=0) (MISSING=0) INTO echo_hfapeff_minorfunc_b.v0.

EXECUTE.

COMPUTE echo_hfapeff_minorfunc_sum.v0=echo_hfapeff_minorfunc_a.v0 + echo_hfapeff_minorfunc_b.v0.

EXECUTE.

RECODE echo_hfapeff_minorfunc_sum.v0 (MISSING=0) (0=0) (ELSE=1) INTO echo_hfapeff_minorfunc.v0.

EXECUTE.

COMPUTE echo_hfapeff_func_sum.v0=echo_hfapeff_majorfunc.v0 + echo_hfapeff_minorfunc.v0.

EXECUTE.

RECODE echo_hfapeff_func_sum.v0 (0=0) (1=1) (2=2) (3=2) INTO echo_hfapeff_func.v0.

```
RECODE echo_lavi_baseline (MISSING=0) (34.00001 thru Highest=1) (ELSE=0) INTO
    echo_hfapeff_majormorph_a.v0.
EXECUTE.

DO IF  (gender = 1).
RECODE echo_lvmi_devereux_baseline (MISSING=0) (149 thru Highest=1) (ELSE=0)
INTO
    echo_hfapeff_majormorph_b1.v0.
END IF.
EXECUTE.

DO IF  (gender = 2).
RECODE echo_lvmi_devereux_baseline (MISSING=0) (122 thru Highest=1) (ELSE=0)
INTO
    echo_hfapeff_majormorph_b1.v0.
END IF.
EXECUTE.

RECODE echo_rwt_baseline (MISSING=0) (0.420000001 thru Highest=1) (ELSE=0) INTO
    echo_hfapeff_majormorph_b2.v0.
EXECUTE.

COMPUTE echo_hfapeff_majormorph_bsum.v0=echo_hfapeff_majormorph_b1.v0 +
    echo_hfapeff_majormorph_b2.v0.
EXECUTE.

RECODE echo_hfapeff_majormorph_bsum.v0 (0=0) (1=1) (2=1) INTO
echo_hfapeff_majormorph_b.v0.
```

```
EXECUTE.

COMPUTE echo_hfapeff_majormorph_sum.v0=echo_hfapeff_majormorph_b.v0 +
echo_hfapeff_majormorph_a.v0.
EXECUTE.

RECODE echo_hfapeff_majormorph_sum.v0 (0=0) (1=2) (2=2) INTO
echo_hfapeff_majormorph.v0.
EXECUTE.

RECODE echo_lavi_baseline (29 thru 34=1) (ELSE=0) (MISSING=0) INTO
echo_hfapeff_minormorph_a.v0.
EXECUTE.

DO IF  (gender = 1).
RECODE echo_lvmi_devereux_baseline (MISSING=0) (115.00001 thru Highest=1)
(ELSE=0) INTO
   echo_hfapeff_minormorph_b.v0.
END IF.
EXECUTE.

DO IF  (gender = 2).
RECODE echo_lvmi_devereux_baseline (MISSING=0) (95.00001 thru Highest=1)
(ELSE=0) INTO
   echo_hfapeff_minormorph_b.v0.
END IF.
EXECUTE.

RECODE echo_rwt_baseline (MISSING=0) (0.420000001 thru Highest=1) (ELSE=0) INTO
```

```
    echo_hfapeff_minormorph_c.v0.
EXECUTE.


RECODE echo_ivsd.v0 (MISSING=0) (12 thru Highest=1) (ELSE=0) INTO
    echo_hfapeff_minormorph_d.v0.
EXECUTE.


COMPUTE echo_hfapeff_minormorph_sum.v0=echo_hfapeff_minormorph_a.v0 +
echo_hfapeff_minormorph_b.v0 + echo_hfapeff_minormorph_c.v0 +
echo_hfapeff_minormorph_d.v0.
EXECUTE.


RECODE echo_hfapeff_minormorph_sum.v0 (0=0) (1=1) (2=1) (3=1) (4=1) INTO
echo_hfapeff_minormorph.v0.
EXECUTE.




COMPUTE echo_hfapeff_morph_sum.v0=echo_hfapeff_majormorph.v0 +
echo_hfapeff_minormorph.v0.
EXECUTE.


RECODE echo_hfapeff_morph_sum.v0 (0=0) (1=1) (2=2) (3=2) INTO
echo_hfapeff_morph.v0.



DO IF  (cardio_rhytm___2 = 1).
RECODE lab_ntprobnp_baseline (MISSING=0) (Lowest thru 364.999=0) (365 thru 660=1)
(660.000001 thru
    Highest=2) INTO echo_hfapeff_biomarker.v0.
END IF.
```

```
EXECUTE.

DO IF  (cardio_rhytm___2 = 0).
RECODE lab_ntprobnp_baseline (MISSING=0) (Lowest thru 124.999=0) (125 thru 220=1) (220.000001 thru
   Highest=2) INTO echo_hfapeff_biomarker.v0.
END IF.
EXECUTE.

COMPUTE echo_hfapeff_score.v0=echo_hfapeff_func.v0 + echo_hfapeff_morph.v0 +
   echo_hfapeff_biomarker.v0.
EXECUTE.

RECODE echo_e_septal.v24 (Lowest thru 6.99999=1) (7 thru Highest=0) (MISSING=0) INTO echo_hfapeff_majorfunc_a.v24.
EXECUTE.

RECODE echo_e_lateral.v24 (Lowest thru 9.99999=1) (10 thru Highest=0) (MISSING=0) INTO echo_hfapeff_majorfunc_b.v24.
EXECUTE.

RECODE echo_ratio_ee.v24 (Lowest thru 0.15=0) (0.150001 thru Highest=1) (MISSING=0) INTO echo_hfapeff_majorfunc_c.v24.
EXECUTE.
```

```
RECODE echo_strv_annulus.v24 (Lowest thru 2.8=0) (2.80001 thru Highest=1)
(MISSING=0) INTO echo_hfapeff_majorfunc_d.v24.

EXECUTE.


COMPUTE echo_hfapeff_majorfunc_sum.v24=echo_hfapeff_majorfunc_a.v24 +
echo_hfapeff_majorfunc_b.v24 +

   echo_hfapeff_majorfunc_c.v24 + echo_hfapeff_majorfunc_d.v24.

EXECUTE.


RECODE echo_hfapeff_majorfunc_sum.v24 (MISSING=0) (0=0) (ELSE=2) INTO
echo_hfapeff_majorfunc.v24.

EXECUTE.


RECODE echo_ratio_ee.v24 (0.09 thru 0.14=1) (ELSE=0) (MISSING=0) INTO
echo_hfapeff_minorfunc_a.v24.

EXECUTE.


COMPUTE echo_gls_positive.v24=SQRT(echo_gls.v24 * echo_gls.v24).

EXECUTE.


RECODE echo_gls_positive.v24 (0.00 thru 15.99=1) (16.00 thru Highest=0) (MISSING=0)
INTO echo_hfapeff_minorfunc_b.v24.

EXECUTE.


COMPUTE echo_hfapeff_minorfunc_sum.v24=echo_hfapeff_minorfunc_a.v24 +
echo_hfapeff_minorfunc_b.v24.

EXECUTE.
```

```
RECODE echo_hfapeff_minorfunc_sum.v24 (MISSING=0) (0=0) (ELSE=1) INTO
echo_hfapeff_minorfunc.v24.
EXECUTE.



COMPUTE echo_hfapeff_func_sum.v24=echo_hfapeff_majorfunc.v24 +
echo_hfapeff_minorfunc.v24.
EXECUTE.


RECODE echo_hfapeff_func_sum.v24 (0=0) (1=1) (2=2) (3=2) INTO
echo_hfapeff_func.v24.



RECODE echo_lavi_v24 (MISSING=0) (34.00001 thru Highest=1) (ELSE=0) INTO
   echo_hfapeff_majormorph_a.v24.
EXECUTE.


DO IF  (gender = 1).
RECODE echo_lvmi_devereux_v24 (MISSING=0) (149 thru Highest=1) (ELSE=0) INTO
   echo_hfapeff_majormorph_b1.v24.
END IF.
EXECUTE.


DO IF  (gender = 2).
RECODE echo_lvmi_devereux_v24 (MISSING=0) (122 thru Highest=1) (ELSE=0) INTO
   echo_hfapeff_majormorph_b1.v24.
END IF.
EXECUTE.
```

```
RECODE echo_rwt_v24 (MISSING=0) (0.420000001 thru Highest=1) (ELSE=0) INTO
    echo_hfapeff_majormorph_b2.v24.
EXECUTE.

COMPUTE echo_hfapeff_majormorph_bsum.v24=echo_hfapeff_majormorph_b1.v24 +
    echo_hfapeff_majormorph_b2.v24.
EXECUTE.

RECODE echo_hfapeff_majormorph_bsum.v24 (0=0) (1=1) (2=1) INTO
echo_hfapeff_majormorph_b.v24.
EXECUTE.

COMPUTE echo_hfapeff_majormorph_sum.v24=echo_hfapeff_majormorph_b.v24 +
echo_hfapeff_majormorph_a.v24.
EXECUTE.

RECODE echo_hfapeff_majormorph_sum.v24 (0=0) (1=2) (2=2) INTO
echo_hfapeff_majormorph.v24.
EXECUTE.



RECODE echo_lavi_v24 (29 thru 34=1) (ELSE=0) (MISSING=0) INTO
echo_hfapeff_minormorph_a.v24.
EXECUTE.

DO IF  (gender = 1).
RECODE echo_lvmi_devereux_v24 (MISSING=0) (115.00001 thru Highest=1) (ELSE=0)
INTO
    echo_hfapeff_minormorph_b.v24.
```

```
END IF.

EXECUTE.


DO IF  (gender = 2).

RECODE echo_lvmi_devereux_v24 (MISSING=0) (95.00001 thru Highest=1) (ELSE=0)
INTO

   echo_hfapeff_minormorph_b.v24.

END IF.

EXECUTE.


RECODE echo_rwt_v24 (MISSING=0) (0.420000001 thru Highest=1) (ELSE=0) INTO

   echo_hfapeff_minormorph_c.v24.

EXECUTE.


RECODE echo_ivsd.v24 (MISSING=0) (12 thru Highest=1) (ELSE=0) INTO

   echo_hfapeff_minormorph_d.v24.

EXECUTE.


COMPUTE echo_hfapeff_minormorph_sum.v24=echo_hfapeff_minormorph_a.v24 +
echo_hfapeff_minormorph_b.v24 + echo_hfapeff_minormorph_c.v24 +
echo_hfapeff_minormorph_d.v24.

EXECUTE.


RECODE echo_hfapeff_minormorph_sum.v24 (0=0) (1=1) (2=1) (3=1) (4=1) INTO
echo_hfapeff_minormorph.v24.

EXECUTE.


COMPUTE echo_hfapeff_morph_sum.v24=echo_hfapeff_majormorph.v24 +
echo_hfapeff_minormorph.v24.

EXECUTE.
```

```
RECODE echo_hfapeff_morph_sum.v24 (0=0) (1=1) (2=2) (3=2) INTO
echo_hfapeff_morph.v24.




DO IF  (cardio_rhytm___2 = 1).
RECODE lab_ntprobnp_eos (MISSING=0) (Lowest thru 364.999=0) (365 thru 660=1)
(660.000001 thru
    Highest=2) INTO echo_hfapeff_biomarker.v24.
END IF.
EXECUTE.

DO IF  (cardio_rhytm___2 = 0).
RECODE lab_ntprobnp_eos (MISSING=0) (Lowest thru 124.999=0) (125 thru 220=1)
(220.000001 thru
    Highest=2) INTO echo_hfapeff_biomarker.v24.
END IF.
EXECUTE.




COMPUTE echo_hfapeff_score.v24=echo_hfapeff_func.v24 + echo_hfapeff_morph.v24 +
    echo_hfapeff_biomarker.v24.
EXECUTE.

*Neuropsychological tests*

COMPUTE
np_wf_mean_pscore=MEAN(np_wf_professions_pscore,np_wf_animals_pscore).
```

EXECUTE.

COMPUTE
np_wf_mean_pscore_eos=MEAN(np_wf_professions_pscore_eos,np_wf_animals_pscore_eo
s).

EXECUTE.

COMPUTE np_sdmt_mean_tscore=MEAN(np_sdmt_tscore,np_sdmt_tscore_high).

EXECUTE.

COMPUTE
np_sdmt_mean_tscore_eos=MEAN(np_sdmt_tscore_eos,np_sdmt_tscore_high_eos).

EXECUTE.

COMPUTE np_domein_geheugen_baseline=(np_dst_forward_pscore +
np_15wt_goed_pscore + np_15wt_recall_pscore) / 3.

EXECUTE.

COMPUTE np_domein_aandacht_baseline=(((np_sdmt_tscore + np_sdmt_tscore_high)/2) +
np_tmt_a_pscore) / 2.

EXECUTE.

COMPUTE np_domein_executieffunctioneren_baseline=(np_tmt_b_pscore +
np_wf_letter_pscore + np_dst_backward_pscore) / 3.

EXECUTE.

COMPUTE np_domein_geheugen_eos=(np_dst_forward_pscore_eos +
np_15wt_goed_pscore_eos + np_15wt_recall_pscore_eos) / 3.

EXECUTE.

COMPUTE np_domein_aandacht_eos=(((np_sdmt_tscore_eos +
np_sdmt_tscore_high_eos)/2) + np_tmt_a_pscore_eos) / 2.

EXECUTE.

COMPUTE np_domein_executieffunctioneren_eos=(np_tmt_b_pscore_eos + np_wf_letter_pscore_eos + np_dst_backward_pscore_eos) / 3.

EXECUTE.

Quality of life

CIS

RECODE qol_cis_1.v0 (1=7) (2=6) (3=5) (4=4) (5=3) (6=2) (7=1) INTO qol_cis_1_decod.v0.

EXECUTE.

RECODE qol_cis_3.v0 (1=7) (2=6) (3=5) (4=4) (5=3) (6=2) (7=1) INTO qol_cis_3_decod.v0.

EXECUTE.

RECODE qol_cis_4.v0 (1=7) (2=6) (3=5) (4=4) (5=3) (6=2) (7=1) INTO qol_cis_4_decod.v0.

EXECUTE.

RECODE qol_cis_9.v0 (1=7) (2=6) (3=5) (4=4) (5=3) (6=2) (7=1) INTO qol_cis_9_decod.v0.

EXECUTE.

RECODE qol_cis_10.v0 (1=7) (2=6) (3=5) (4=4) (5=3) (6=2) (7=1) INTO qol_cis_10_decod.v0.

EXECUTE.

RECODE qol_cis_13.v0 (1=7) (2=6) (3=5) (4=4) (5=3) (6=2) (7=1) INTO qol_cis_13_decod.v0.

EXECUTE.

RECODE qol_cis_14.v0 (1=7) (2=6) (3=5) (4=4) (5=3) (6=2) (7=1) INTO qol_cis_14_decod.v0.

EXECUTE.

RECODE qol_cis_16.v0 (1=7) (2=6) (3=5) (4=4) (5=3) (6=2) (7=1) INTO qol_cis_16_decod.v0.

EXECUTE.

RECODE qol_cis_17.v0 (1=7) (2=6) (3=5) (4=4) (5=3) (6=2) (7=1) INTO qol_cis_17_decod.v0.

EXECUTE.

RECODE qol_cis_18.v0 (1=7) (2=6) (3=5) (4=4) (5=3) (6=2) (7=1) INTO qol_cis_18_decod.v0.

EXECUTE.

RECODE qol_cis_19.v0 (1=7) (2=6) (3=5) (4=4) (5=3) (6=2) (7=1) INTO qol_cis_19_decod.v0.

EXECUTE.

RECODE qol_cis_1.v24 (1=7) (2=6) (3=5) (4=4) (5=3) (6=2) (7=1) INTO qol_cis_1_decod.v24.

EXECUTE.

RECODE qol_cis_3.v24 (1=7) (2=6) (3=5) (4=4) (5=3) (6=2) (7=1) INTO qol_cis_3_decod.v24.

EXECUTE.

```
RECODE qol_cis_4.v24 (1=7) (2=6) (3=5) (4=4) (5=3) (6=2) (7=1) INTO
qol_cis_4_decod.v24.

EXECUTE.


RECODE qol_cis_9.v24 (1=7) (2=6) (3=5) (4=4) (5=3) (6=2) (7=1) INTO
qol_cis_9_decod.v24.

EXECUTE.


RECODE qol_cis_10.v24 (1=7) (2=6) (3=5) (4=4) (5=3) (6=2) (7=1) INTO
qol_cis_10_decod.v24.

EXECUTE.


RECODE qol_cis_13.v24 (1=7) (2=6) (3=5) (4=4) (5=3) (6=2) (7=1) INTO
qol_cis_13_decod.v24.

EXECUTE.


RECODE qol_cis_14.v24 (1=7) (2=6) (3=5) (4=4) (5=3) (6=2) (7=1) INTO
qol_cis_14_decod.v24.

EXECUTE.


RECODE qol_cis_16.v24 (1=7) (2=6) (3=5) (4=4) (5=3) (6=2) (7=1) INTO
qol_cis_16_decod.v24.

EXECUTE.


RECODE qol_cis_17.v24 (1=7) (2=6) (3=5) (4=4) (5=3) (6=2) (7=1) INTO
qol_cis_17_decod.v24.

EXECUTE.


RECODE qol_cis_18.v24 (1=7) (2=6) (3=5) (4=4) (5=3) (6=2) (7=1) INTO
qol_cis_18_decod.v24.

EXECUTE.
```

RECODE qol_cis_19.v24 (1=7) (2=6) (3=5) (4=4) (5=3) (6=2) (7=1) INTO qol_cis_19_decod.v24.

EXECUTE.

COMPUTE qol_cis_sum.v0=qol_cis_1_decod.v0 + qol_cis_3_decod.v0 + qol_cis_4_decod.v0 +

qol_cis_9_decod.v0 + qol_cis_10_decod.v0 + qol_cis_13_decod.v0 + qol_cis_14_decod.v0 +

qol_cis_16_decod.v0 + qol_cis_17_decod.v0 + qol_cis_18_decod.v0 + qol_cis_19_decod.v0 +

qol_cis_2.v0 + qol_cis_5.v0 + qol_cis_6.v0 + qol_cis_7.v0 + qol_cis_8.v0 + qol_cis_11.v0 + qol_cis_12.v0 + qol_cis_15.v0 + qol_cis_20.v0.

EXECUTE.

COMPUTE qol_cis_sum.v24=qol_cis_1_decod.v24 + qol_cis_3_decod.v24 + qol_cis_4_decod.v24 +

qol_cis_9_decod.v24 + qol_cis_10_decod.v24 + qol_cis_13_decod.v24 + qol_cis_14_decod.v24 +

qol_cis_16_decod.v24 + qol_cis_17_decod.v24 + qol_cis_18_decod.v24 + qol_cis_19_decod.v24 +

qol_cis_2.v24 + qol_cis_5.v24 + qol_cis_6.v24 + qol_cis_7.v24 + qol_cis_8.v24 + qol_cis_11.v24 + qol_cis_12.v24 + qol_cis_15.v24 + qol_cis_20.v24.

EXECUTE.

*QOL rand36 *

RECODE qol_rand36_1.v0 (1=5) (2=4) (3=3) (4=2) (5=1) INTO qol_rand36_1_decod.v0.

EXECUTE.

```
RECODE qol_rand36_2.v0 (1=5) (2=4) (3=3) (4=2) (5=1) INTO qol_rand36_2_decod.v0.
EXECUTE.

RECODE qol_rand36_6.v0 (1=5) (2=4) (3=3) (4=2) (5=1) INTO qol_rand36_6_decod.v0.
EXECUTE.

RECODE qol_rand36_8.v0 (1=5) (2=4) (3=3) (4=2) (5=1) INTO qol_rand36_8_decod.v0.
EXECUTE.

RECODE qol_rand36_11b.v0 (1=5) (2=4) (3=3) (4=2) (5=1) INTO
qol_rand36_11b_decod.v0.
EXECUTE.

RECODE qol_rand36_11d.v0 (1=5) (2=4) (3=3) (4=2) (5=1) INTO
qol_rand36_11d_decod.v0.
EXECUTE.

RECODE qol_rand36_9a.v0 (1=5) (2=4) (3=3) (4=2) (5=1) INTO qol_rand36_9a_decod.v0.
EXECUTE.

RECODE qol_rand36_9d.v0 (1=5) (2=4) (3=3) (4=2) (5=1) INTO qol_rand36_9d_decod.v0.
EXECUTE.

RECODE qol_rand36_9e.v0 (1=5) (2=4) (3=3) (4=2) (5=1) INTO qol_rand36_9e_decod.v0.
EXECUTE.

RECODE qol_rand36_9h.v0 (1=5) (2=4) (3=3) (4=2) (5=1) INTO qol_rand36_9h_decod.v0.
EXECUTE.
```

RECODE qol_rand36_7.v0 (1=6) (2=5) (3=4) (4=3) (5=2) (6=1) INTO qol_rand36_7_decod.v0.

EXECUTE.

RECODE qol_rand36_1.v24 (1=5) (2=4) (3=3) (4=2) (5=1) INTO qol_rand36_1_decod.v24.

EXECUTE.

RECODE qol_rand36_2.v24 (1=5) (2=4) (3=3) (4=2) (5=1) INTO qol_rand36_2_decod.v24.

EXECUTE.

RECODE qol_rand36_6.v24 (1=5) (2=4) (3=3) (4=2) (5=1) INTO qol_rand36_6_decod.v24.

EXECUTE.

RECODE qol_rand36_8.v24 (1=5) (2=4) (3=3) (4=2) (5=1) INTO qol_rand36_8_decod.v24.

EXECUTE.

RECODE qol_rand36_11b.v24 (1=5) (2=4) (3=3) (4=2) (5=1) INTO qol_rand36_11b_decod.v24.

EXECUTE.

RECODE qol_rand36_11d.v24 (1=5) (2=4) (3=3) (4=2) (5=1) INTO qol_rand36_11d_decod.v24.

EXECUTE.

RECODE qol_rand36_9a.v24 (1=5) (2=4) (3=3) (4=2) (5=1) INTO qol_rand36_9a_decod.v24.

EXECUTE.

RECODE qol_rand36_9d.v24 (1=5) (2=4) (3=3) (4=2) (5=1) INTO qol_rand36_9d_decod.v24.

EXECUTE.

```
RECODE qol_rand36_9e.v24 (1=5) (2=4) (3=3) (4=2) (5=1) INTO
qol_rand36_9e_decod.v24.

EXECUTE.


RECODE qol_rand36_9h.v24 (1=5) (2=4) (3=3) (4=2) (5=1) INTO
qol_rand36_9h_decod.v24.

EXECUTE.


RECODE qol_rand36_7.v24 (1=6) (2=5) (3=4) (4=3) (5=2) (6=1) INTO
qol_rand36_7_decod.v24.

EXECUTE.


COMPUTE qol_rand36_fysiekfunctioneren_raw.v0=qol_rand36_3a.v0 + qol_rand36_3b.v0
+ qol_rand36_3c.v0

  + qol_rand36_3d.v0 + qol_rand36_3e.v0 + qol_rand36_3f.v0 + qol_rand36_3g.v0 +
qol_rand36_3h.v0 +

  qol_rand36_3i.v0 + qol_rand36_3j.v0.

EXECUTE.


COMPUTE qol_rand36_fysiekfunctioneren_raw.v24=qol_rand36_3a.v24 +
qol_rand36_3b.v24 + qol_rand36_3c.v24

  + qol_rand36_3d.v24 + qol_rand36_3e.v24 + qol_rand36_3f.v24 + qol_rand36_3g.v24 +
qol_rand36_3h.v24 +

  qol_rand36_3i.v24 + qol_rand36_3j.v24.

EXECUTE.
```

COMPUTE qol_rand36_sociaalfunctioneren_raw.v0=qol_rand36_6_decod.v0 + qol_rand36_10.v0.

EXECUTE.

COMPUTE qol_rand36_sociaalfunctioneren_raw.v24=qol_rand36_6_decod.v24 + qol_rand36_10.v24.

EXECUTE.

COMPUTE qol_rand36_fysiekerolbeperking_raw.v0=qol_rand36_4a.v0 + qol_rand36_4b.v0 + qol_rand36_4c.v0 + qol_rand36_4d.v0.

EXECUTE.

COMPUTE qol_rand36_fysiekerolbeperking_raw.v24=qol_rand36_4a.v24 + qol_rand36_4b.v24 + qol_rand36_4c.v24 + qol_rand36_4d.v24.

EXECUTE.

COMPUTE qol_rand36_emotionelerolbeperking_raw.v0=qol_rand36_5a.v0 + qol_rand36_5b.v0 + qol_rand36_5c.v0.

EXECUTE.

COMPUTE qol_rand36_emotionelerolbeperking_raw.v24=qol_rand36_5a.v24 + qol_rand36_5b.v24 + qol_rand36_5c.v24.

EXECUTE.

COMPUTE qol_rand36_mentalegezondheid_raw.v0=qol_rand36_9b.v0 + qol_rand36_9c.v0 + qol_rand36_9d_decod.v0 + qol_rand36_9f.v0 + qol_rand36_9h_decod.v0.

EXECUTE.

COMPUTE qol_rand36_mentalegezondheid_raw.v24=qol_rand36_9b.v24 + qol_rand36_9c.v24 + qol_rand36_9d_decod.v24 + qol_rand36_9f.v24 + qol_rand36_9h_decod.v24.

EXECUTE.

COMPUTE qol_rand36_vitaliteit_raw.v0=qol_rand36_9a_decod.v0 + qol_rand36_9e_decod.v0 + qol_rand36_9g.v0 + qol_rand36_9i.v0.

EXECUTE.

COMPUTE qol_rand36_vitaliteit_raw.v24=qol_rand36_9a_decod.v24 + qol_rand36_9e_decod.v24 + qol_rand36_9g.v24 + qol_rand36_9i.v24.

EXECUTE.

COMPUTE qol_rand36_pijn_raw.v0=(5 * qol_rand36_7_decod.v0) + (6 * qol_rand36_8.v0).

EXECUTE.

COMPUTE qol_rand36_pijn_raw.v24=(5 * qol_rand36_7_decod.v24) + (6 * qol_rand36_8.v24).

EXECUTE.

COMPUTE qol_rand36_algemeen_raw.v0=qol_rand36_1_decod.v0 + qol_rand36_11a.v0 + qol_rand36_11b_decod.v0 + qol_rand36_11c.v0 + qol_rand36_11d_decod.v0.

EXECUTE.

COMPUTE qol_rand36_algemeen_raw.v24=qol_rand36_1_decod.v24 + qol_rand36_11a.v24 + qol_rand36_11b_decod.v24 + qol_rand36_11c.v24 + qol_rand36_11d_decod.v24.

EXECUTE.

COMPUTE qol_rand36_verandering_raw.v0=qol_rand36_2_decod.v0.

EXECUTE.

COMPUTE qol_rand36_verandering_raw.v24=qol_rand36_2_decod.v24.

EXECUTE.

```
COMPUTE
qol_rand36_fysiekfunctioneren_schaal.v0=((qol_rand36_fysiekfunctioneren_raw.v0 - 10) /
20) * 100.

EXECUTE.


COMPUTE
qol_rand36_sociaalfunctioneren_schaal.v0=((qol_rand36_sociaalfunctioneren_raw.v0 - 2) /
8) * 100.

EXECUTE.


COMPUTE
qol_rand36_fysiekerolbeperking_schaal.v0=((qol_rand36_fysiekerolbeperking_raw.v0 - 4) /
16) * 100.

EXECUTE.


COMPUTE
qol_rand36_emotionelerolbeperking_schaal.v0=((qol_rand36_emotionelerolbeperking_raw.v
0 - 3) / 12) * 100.

EXECUTE.


COMPUTE
qol_rand36_mentalegezondheid_schaal.v0=((qol_rand36_mentalegezondheid_raw.v0 - 5) /
20) * 100.

EXECUTE.


COMPUTE qol_rand36_vitaliteit_schaal.v0=((qol_rand36_vitaliteit_raw.v0 - 4) / 16) * 100.

EXECUTE.


COMPUTE qol_rand36_pijn_schaal.v0=((qol_rand36_pijn_raw.v0 - 11) / 49) * 100.

EXECUTE.
```

COMPUTE qol_rand36_algemeen_schaal.v0=((qol_rand36_algemeen_raw.v0 - 5) / 20) * 100.

EXECUTE.

COMPUTE qol_rand36_verandering_schaal.v0=((qol_rand36_verandering_raw.v0 - 1) / 4) * 100.

EXECUTE.

COMPUTE qol_rand36_fysiekfunctioneren_schaal.v24=((qol_rand36_fysiekfunctioneren_raw.v24 - 10) / 20) * 100.

EXECUTE.

COMPUTE qol_rand36_sociaalfunctioneren_schaal.v24=((qol_rand36_sociaalfunctioneren_raw.v24 - 2) / 8) * 100.

EXECUTE.

COMPUTE qol_rand36_fysiekerolbeperking_schaal.v24=((qol_rand36_fysiekerolbeperking_raw.v24 - 4) / 16) * 100.

EXECUTE.

COMPUTE qol_rand36_emotionelerolbeperking_schaal.v24=((qol_rand36_emotionelerolbeperking_raw.v24 - 3) / 12) * 100.

EXECUTE.

COMPUTE qol_rand36_mentalegezondheid_schaal.v24=((qol_rand36_mentalegezondheid_raw.v24 - 5) / 20) * 100.

EXECUTE.

COMPUTE qol_rand36_vitaliteit_schaal.v24=((qol_rand36_vitaliteit_raw.v24 - 4) / 16) * 100.

EXECUTE.

COMPUTE qol_rand36_pijn_schaal.v24=((qol_rand36_pijn_raw.v24 - 11) / 49) * 100.

EXECUTE.

COMPUTE qol_rand36_algemeen_schaal.v24=((qol_rand36_algemeen_raw.v24 - 5) / 20) * 100.

EXECUTE.

COMPUTE qol_rand36_verandering_schaal.v24=((qol_rand36_verandering_raw.v24 - 1) / 4) * 100.

EXECUTE.

EQ5D dutch index

IF (qol_eq5d5l_1.v0=1) disut_mo.v0=0.

IF (qol_eq5d5l_1.v0=2) disut_mo.v0=0.035.

IF (qol_eq5d5l_1.v0=3) disut_mo.v0=0.057.

IF (qol_eq5d5l_1.v0=4) disut_mo.v0=0.166.

IF (qol_eq5d5l_1.v0=5) disut_mo.v0=0.203.

IF (qol_eq5d5l_2.v0=1) disut_sc.v0=0.

IF (qol_eq5d5l_2.v0=2) disut_sc.v0=0.038.

IF (qol_eq5d5l_2.v0=3) disut_sc.v0=0.061.

IF (qol_eq5d5l_2.v0=4) disut_sc.v0=0.168.

IF (qol_eq5d5l_2.v0=5) disut_sc.v0=0.168.

IF (qol_eq5d5l_3.v0=1) disut_ua.v0=0.

IF (qol_eq5d5l_3.v0=2) disut_ua.v0=0.039.

IF (qol_eq5d5l_3.v0=3) disut_ua.v0=0.087.

IF (qol_eq5d5l_3.v0=4) disut_ua.v0=0.192.

IF (qol_eq5d5l_3.v0=5) disut_ua.v0=0.192.

IF (qol_eq5d5l.v0=1) disut_p.v0=0.

IF (qol_eq5d5l.v0=2) disut_p.v0=0.066.

IF (qol_eq5d5l.v0=3) disut_p.v0=0.092.

IF (qol_eq5d5l.v0=4) disut_p.v0=0.360.

IF (qol_eq5d5l.v0=5) disut_p.v0=0.415.

IF (qol_eq5d5l_5.v0=1) disut_ad.v0=0.

IF (qol_eq5d5l_5.v0=2) disut_ad.v0=0.070.

IF (qol_eq5d5l_5.v0=3) disut_ad.v0=0.145.

IF (qol_eq5d5l_5.v0=4) disut_ad.v0=0.356.

IF (qol_eq5d5l_5.v0=5) disut_ad.v0=0.421.

Compute disut_total.v0= disut_mo.v0 +disut_sc.v0 +disut_ua.v0 +disut_p.v0 +disut_ad.v0.

Compute EQindex.v0 = 1-0.047-disut_total.v0.

IF (qol_eq5d5l_1.v0= 1 & qol_eq5d5l_2.v0= 1 & qol_eq5d5l_3.v0= 1 & qol_eq5d5l.v0= 1 & qol_eq5d5l_5.v0= 1) EQindex.v0 = 1.

execute.

IF (qol_eq5d5l_1.v24=1) disut_mo.v24=0.

IF (qol_eq5d5l_1.v24=2) disut_mo.v24=0.035.

IF (qol_eq5d5l_1.v24=3) disut_mo.v24=0.057.

IF (qol_eq5d5l_1.v24=4) disut_mo.v24=0.166.

IF (qol_eq5d5l_1.v24=5) disut_mo.v24=0.203.

IF (qol_eq5d5l_2.v24=1) disut_sc.v24=0.

IF (qol_eq5d5l_2.v24=2) disut_sc.v24=0.038.

IF (qol_eq5d5l_2.v24=3) disut_sc.v24=0.061.

IF (qol_eq5d5l_2.v24=4) disut_sc.v24=0.168.

IF (qol_eq5d5l_2.v24=5) disut_sc.v24=0.168.

IF (qol_eq5d5l_3.v24=1) disut_ua.v24=0.

IF (qol_eq5d5l_3.v24=2) disut_ua.v24=0.039.

IF (qol_eq5d5l_3.v24=3) disut_ua.v24=0.087.

IF (qol_eq5d5l_3.v24=4) disut_ua.v24=0.192.

IF (qol_eq5d5l_3.v24=5) disut_ua.v24=0.192.

IF (qol_eq5d5l.v24=1) disut_p.v24=0.

IF (qol_eq5d5l.v24=2) disut_p.v24=0.066.

IF (qol_eq5d5l.v24=3) disut_p.v24=0.092.

IF (qol_eq5d5l.v24=4) disut_p.v24=0.360.

IF (qol_eq5d5l.v24=5) disut_p.v24=0.415.

IF (qol_eq5d5l_5.v24=1) disut_ad.v24=0.

IF (qol_eq5d5l_5.v24=2) disut_ad.v24=0.070.

IF (qol_eq5d5l_5.v24=3) disut_ad.v24=0.145.

IF (qol_eq5d5l_5.v24=4) disut_ad.v24=0.356.

IF (qol_eq5d5l_5.v24=5) disut_ad.v24=0.421.

Compute disut_total.v24= disut_mo.v24 +disut_sc.v24 +disut_ua.v24 +disut_p.v24 +disut_ad.v24.

Compute EQindex.v24 = 1-0.047-disut_total.v24.

IF (qol_eq5d5l_1.v24= 1 & qol_eq5d5l_2.v24= 1 & qol_eq5d5l_3.v24= 1 & qol_eq5d5l.v24= 1 & qol_eq5d5l_5.v24= 1) EQindex.v24 = 1.

```
execute.

*DMFS*

RECODE qol_dmfs22.v0 (1=5) (2=4) (3=3) (4=2) (5=1) INTO qol_dmfs22_decod.v0.
EXECUTE.

RECODE qol_dmfs22.v24 (1=5) (2=4) (3=3) (4=2) (5=1) INTO qol_dmfs22_decod.v24.
EXECUTE.

RECODE qol_dmfs24.v0 (1=5) (2=4) (3=3) (4=2) (5=1) INTO qol_dmfs24_decod.v0.
EXECUTE.

RECODE qol_dmfs24.v24 (1=5) (2=4) (3=3) (4=2) (5=1) INTO qol_dmfs24_decod.v24.
EXECUTE.

RECODE qol_dmfs11.v0 (1=5) (2=4) (3=3) (4=2) (5=1) INTO qol_dmfs11_decod.v0.
EXECUTE.

RECODE qol_dmfs11.v24 (1=5) (2=4) (3=3) (4=2) (5=1) INTO qol_dmfs11_decod.v24.
EXECUTE.

RECODE qol_dmfs3.v0 (1=5) (2=4) (3=3) (4=2) (5=1) INTO qol_dmfs3_decod.v0.
EXECUTE.

RECODE qol_dmfs3.v24 (1=5) (2=4) (3=3) (4=2) (5=1) INTO qol_dmfs3_decod.v24.
EXECUTE.

RECODE qol_dmfs5.v0 (1=5) (2=4) (3=3) (4=2) (5=1) INTO qol_dmfs5_decod.v0.
```

EXECUTE.

RECODE qol_dmfs5.v24 (1=5) (2=4) (3=3) (4=2) (5=1) INTO qol_dmfs5_decod.v24.
EXECUTE.

RECODE qol_dmfs9.v0 (1=5) (2=4) (3=3) (4=2) (5=1) INTO qol_dmfs9_decod.v0.
EXECUTE.

RECODE qol_dmfs9.v24 (1=5) (2=4) (3=3) (4=2) (5=1) INTO qol_dmfs9_decod.v24.
EXECUTE.

RECODE qol_dmfs14.v0 (1=5) (2=4) (3=3) (4=2) (5=1) INTO qol_dmfs14_decod.v0.
EXECUTE.

RECODE qol_dmfs14.v24 (1=5) (2=4) (3=3) (4=2) (5=1) INTO qol_dmfs14_decod.v24.
EXECUTE.

RECODE qol_dmfs2.v0 (1=5) (2=4) (3=3) (4=2) (5=1) INTO qol_dmfs2_decod.v0.
EXECUTE.

RECODE qol_dmfs2.v24 (1=5) (2=4) (3=3) (4=2) (5=1) INTO qol_dmfs2_decod.v24.
EXECUTE.

RECODE qol_dmfs16.v0 (1=5) (2=4) (3=3) (4=2) (5=1) INTO qol_dmfs16_decod.v0.
EXECUTE.

RECODE qol_dmfs16.v24 (1=5) (2=4) (3=3) (4=2) (5=1) INTO qol_dmfs16_decod.v24.
EXECUTE.

COMPUTE qol_dmfs_impactvanvermoeidheid.v0=qol_dmfs1.v0 + qol_dmfs6.v0 + qol_dmfs13.v0 +

qol_dmfs20.v0 + qol_dmfs22_decod.v0 + qol_dmfs24_decod.v0 + qol_dmfs26.v0 + qol_dmfs29.v0 + qol_dmfs33.v0 + qol_dmfs35.v0 + qol_dmfs38.v0.

EXECUTE.

COMPUTE qol_dmfs_directegevolgenvanvermoeidheid.v0=qol_dmfs4.v0 + qol_dmfs7.v0 + qol_dmfs11_decod.v0 + qol_dmfs15.v0 + qol_dmfs19.v0 + qol_dmfs21.v0 + qol_dmfs23.v0 + qol_dmfs28.v0 + qol_dmfs31.v0.

EXECUTE.

COMPUTE qol_dmfs_mentalevermoeidheid.v0=qol_dmfs3_decod.v0 + qol_dmfs10.v0 + qol_dmfs17.v0 + qol_dmfs27.v0 + qol_dmfs32.v0 + qol_dmfs34.v0 + qol_dmfs37.v0.

EXECUTE.

COMPUTE qol_dmfs_fysiekevermoeidheid.v0=qol_dmfs5_decod.v0 + qol_dmfs9_decod.v0 + qol_dmfs14_decod.v0 + qol_dmfs18.v0 + qol_dmfs25.v0 + qol_dmfs30.v0.

EXECUTE.

COMPUTE qol_dmfs_omgaanmetvermoeidheid.v0=qol_dmfs2_decod.v0 + qol_dmfs8.v0 + qol_dmfs12.v0 + qol_dmfs16_decod.v0 + qol_dmfs36.v0.

EXECUTE.

COMPUTE qol_dmfs_impactvanvermoeidheid.v24=qol_dmfs1.v24 + qol_dmfs6.v24 + qol_dmfs13.v24 +

qol_dmfs20.v24 + qol_dmfs22_decod.v24 + qol_dmfs24_decod.v24 + qol_dmfs26.v24 + qol_dmfs29.v24 + qol_dmfs33.v24 + qol_dmfs35.v24 + qol_dmfs38.v24.

EXECUTE.

COMPUTE qol_dmfs_directegevolgenvanvermoeidheid.v24=qol_dmfs4.v24 + qol_dmfs7.v24 + qol_dmfs11_decod.v24 + qol_dmfs15.v24 + qol_dmfs19.v24 + qol_dmfs21.v24 + qol_dmfs23.v24 + qol_dmfs28.v24 + qol_dmfs31.v24.

EXECUTE.

COMPUTE qol_dmfs_mentalevermoeidheid.v24=qol_dmfs3_decod.v24 + qol_dmfs10.v24 + qol_dmfs17.v24 + qol_dmfs27.v24 + qol_dmfs32.v24 + qol_dmfs34.v24 + qol_dmfs37.v24.

EXECUTE.

COMPUTE qol_dmfs_fysiekevermoeidheid.v24=qol_dmfs5_decod.v24 + qol_dmfs9_decod.v24 + qol_dmfs14_decod.v24 + qol_dmfs18.v24 + qol_dmfs25.v24 + qol_dmfs30.v24.

EXECUTE.

COMPUTE qol_dmfs_omgaanmetvermoeidheid.v24=qol_dmfs2_decod.v24 + qol_dmfs8.v24 + qol_dmfs12.v24 + qol_dmfs16_decod.v24 + qol_dmfs36.v24.

EXECUTE.

GSRS

COMPUTE GSRS_total_V0=gsrs_1_abdominalpain.v0 + gsrs_2_heartburn.v0 + gsrs_3_reflux.v0 +

gsrs_4_hungerpain.v0 + gsrs_5_nausea.v0 + gsrs_6_rumbling.v0 + gsrs_7_bloating.v0 +

gsrs_8_burping.v0 + gsrs_9_flatus.v0 + gsrs_10_constipation.v0 + gsrs_11_diarrhea.v0 +

gsrs_12_loosestools.v0 + gsrs_13_hardstools.v0 + gsrs_14_urgency.v0 + gsrs_15_incomplete.v0.

EXECUTE.

COMPUTE GSRS_total_V24=gsrs_1_abdominalpain.v24 + gsrs_2_heartburn.v24 + gsrs_3_reflux.v24 +

gsrs_4_hungerpain.v24 + gsrs_5_nausea.v24 + gsrs_6_rumbling.v24 + gsrs_7_bloating.v24 +

gsrs_8_burping.v24 + gsrs_9_flatus.v24 + gsrs_10_constipation.v24 + gsrs_11_diarrhea.v24 +

gsrs_12_loosestools.v24 + gsrs_13_hardstools.v24 + gsrs_14_urgency.v24 + gsrs_15_incomplete.v24.

```
EXECUTE.

*Predicted 6mwt*

IF  (gender = 1) SixMWT_pred_baseline=489.22 - (4.33 * age_baseline) + (3.19 * length * 100) -
    (2.18 * weight).
EXECUTE.

IF  (gender = 2) SixMWT_pred_baseline=498.06 - (4.80 * age_baseline) + (2.64 * length * 100) -
    (1.24 * weight).
EXECUTE.

COMPUTE SixMWT_percpred_baseline=(sixmwt_distance.v0 / SixMWT_pred_baseline) * 100.
EXECUTE.

COMPUTE SixMWT_metersbelowpred_baseline=(SixMWT_pred_baseline - sixmwt_distance.v0).
EXECUTE.

IF  (gender = 1) SixMWT_pred_eos=489.22 - (4.33 * age_baseline) + (3.19 * length * 100) -
    (2.18 * weight_fu.v24).
EXECUTE.
```

```
IF  (gender = 2) SixMWT_pred_eos=498.06 - (4.80 * age_baseline) + (2.64 * length * 100) -
    (1.24 * weight_fu.v24).
EXECUTE.

COMPUTE SixMWT_percpred_eos=(sixmwt_distance.v24 / SixMWT_pred_eos) * 100.
EXECUTE.

COMPUTE SixMWT_metersbelowpred_eos=(SixMWT_pred_eos - sixmwt_distance.v24).
EXECUTE.


IF  ((treatment_arm = 1) & (lab_ferritine_blinded.v24 < 300))
IDpostfcm=lab_tsat_blinded.v24.
EXECUTE.


*******Generation of three datasets*******
 *-> save as 'raw dataset*

**prepatation of safety analysis dataset**

FILTER OFF.
USE ALL.
SELECT IF ((administr_type.v0 = 2) | (administr_type.v0 = 3) | (administr_type.v12 = 2) |
    (administr_type.v12 = 3) | (administr_type.v18 = 2) | (administr_type.v18 = 3) |
(administr_type.v6
    = 2) | (administr_type.v6 = 3)).
```

```
EXECUTE.

*-> save as safety analysis dataset*

**prepatation of full analysis dataset**

*1. exclusion of 7 participants because of withdrawal during sarscov2 pandemic*

RECODE study_id (‘XXX’=1) ('XXX'=1) ('XXX'=1) ('XXX'=1) ('XXX'=1) ('XXX'=1)
    ('XXX'=1) (ELSE=0) INTO covid_exclusie.
EXECUTE.

FILTER OFF.
USE ALL.
SELECT IF (covid_exclusie = 0).
EXECUTE.

*2. exclusion of participants who were not randomised or who were randomised to the FCM
arm but never received FCM*

FILTER OFF.
USE ALL.
SELECT IF ((treatment_arm = 0) | ((treatment_arm = 1) & ((administr_type.v0 = 2) |
    (administr_type.v12 = 2) | (administr_type.v18 = 2) | (administr_type.v6 = 2)))).
EXECUTE.

*-> save as full analysis dataset*

**prepatation of per protocol analysis dataset**
```

1. exclusion of 7 participants because of withdrawal during sarscov2 pandemic

```
RECODE study_id (‘XXX’=1) (‘XXX’=1) (‘XXX’=1) (‘XXX’=1) (‘XXX’=1) (‘XXX’=1)
    (‘XXX’=1) (ELSE=0) INTO covid_exclusie.
EXECUTE.

FILTER OFF.
USE ALL.
SELECT IF (covid_exclusie = 0).
EXECUTE.
```

2. exclusion of participants who were not randomised or who were randomised to the FCM arm but never received FCM

```
FILTER OFF.
USE ALL.
SELECT IF ((treatment_arm = 0) | ((treatment_arm = 1) & ((administr_type.v0 = 2) |
    (administr_type.v12 = 2) | (administr_type.v18 = 2) | (administr_type.v6 = 2)))).
EXECUTE.
```

3. exclusion of participants violating 6mwt protocol

```
RECODE study_id (‘XXX’=1) (‘XXX’=1) (‘XXX’=1) (‘XXX’=1) (‘XXX’=1) (‘XXX’=1)
    (‘XXX’=1) (‘XXX’=1) (‘XXX’=1) (‘XXX’=1) (‘XXX’=1) (‘XXX’=1) (‘XXX’=1)
(‘XXX’=1) (‘XXX’=1) (‘XXX’=1) (‘XXX’=1) (‘XXX’=1) (ELSE=0) INTO
sixmwt_exclusie.
EXECUTE.
```

```
FILTER OFF.
USE ALL.
SELECT IF (sixmwt_exclusie = 0).
EXECUTE.

*4. exclusion of participants in the FCM arm who received less than 3 FCM doses*

RECODE administr_type.v0 (2=1) (ELSE=0) INTO administration_iron.v0.
EXECUTE.

RECODE administr_type.v6 (2=1) (ELSE=0) INTO administration_iron.v6.
EXECUTE.

RECODE administr_type.v12 (2=1) (ELSE=0) INTO administration_iron.v12.
EXECUTE.

RECODE administr_type.v18 (2=1) (ELSE=0) INTO administration_iron.v18.
EXECUTE.

COMPUTE adminstration_fcm_nr=administration_iron.v0 + administration_iron.v6 +
    administration_iron.v12 + administration_iron.v18.
EXECUTE.

FILTER OFF.
USE ALL.
SELECT IF ((adminstration_fcm_nr = 0) | (adminstration_fcm_nr = 3 |
(adminstration_fcm_nr = 4))).
EXECUTE.
```

5. Exclusion of participants who received blood products, iron (outside study protocol) or EPO stimulating agents

FILTER OFF.

USE ALL.

SELECT IF ((med_oral_iron = 0) | MISSING(med_oral_iron)).

EXECUTE.

FILTER OFF.

USE ALL.

SELECT IF (med_esa = 6).

EXECUTE.

RECODE study_id (‘XXX’=1) (‘XXX’=1) (ELSE=0) INTO iron_exclusie.

EXECUTE.

FILTER OFF.

USE ALL.

SELECT IF (iron_exclusie = 0).

EXECUTE.

-> save as per protocol analysis dataset

****** Imputation deel 2 according to rules of Rubin**********

-> test whether variables are MCAR -> confirmed

-> use the following syntax for the safety analysis database, the full analysis database and the per protocol database separately. change name of imputation database accordingly

SORT CASES BY treatment_arm.

```
SPLIT FILE SEPARATE BY treatment_arm.

DATASET DECLARE effect_safetyanalysis_imputed.
MULTIPLE IMPUTATION
gender ethnicity age_baseline hypertension cardio_hf cardio_cor diabetes_mellitus smoking alcohol
kidney_dialysis transplant_cmv_donor transplant_cmv_participant transplant_induction_il2ra transplant_rejection med_pred
 med_ci
 med_apa
 bmi
 lab_hb_baseline
 lab_crp_baseline
 lab_ferritine_baseline
 lab_tsat_baseline
 lab_egfr_ckdepi2021cras_baseline
  sixmwt_distance.v0
 sixmwt_distance.v24
 ftstst_mean_baseline
 ftstst_mean_eos
 tug_mean_baseline
 tug_mean_eos
 hgs_mean_baseline
 hgs_mean_eos
 qol_cis_sum.v0
 qol_cis_sum.v24
 EQindex.v0
 EQindex.v24
 qol_eq5d5l_6.v0 qol_eq5d5l_6.v24
 qol_dmfs_impactvanvermoeidheid.v0
```

qol_dmfs_directegevolgenvanvermoeidheid.v0

qol_dmfs_mentalevermoeidheid.v0

qol_dmfs_fysiekevermoeidheid.v0

qol_dmfs_omgaanmetvermoeidheid.v0

qol_dmfs_impactvanvermoeidheid.v24

qol_dmfs_directegevolgenvanvermoeidheid.v24

qol_dmfs_mentalevermoeidheid.v24

qol_dmfs_fysiekevermoeidheid.v24

qol_dmfs_omgaanmetvermoeidheid.v24

qol_rand36_fysiekfunctioneren_schaal.v0

qol_rand36_sociaalfunctioneren_schaal.v0

qol_rand36_mentalegezondheid_schaal.v0

qol_rand36_vitaliteit_schaal.v0

qol_rand36_pijn_schaal.v0

qol_rand36_algemeen_schaal.v0

qol_rand36_verandering_schaal.v0

qol_rand36_fysiekfunctioneren_schaal.v24

qol_rand36_sociaalfunctioneren_schaal.v24

qol_rand36_mentalegezondheid_schaal.v24

qol_rand36_vitaliteit_schaal.v24

qol_rand36_pijn_schaal.v24

qol_rand36_algemeen_schaal.v24

qol_rand36_verandering_schaal.v24

echo_ivsd.v0

echo_ivsd.v24

echo_lvidd.v0

echo_lvidd.v24

echo_lvpwd.v0

echo_lvpwd.v24

echo_lavi_baseline

echo_lavi_v24

echo_rwt_baseline

echo_rwt_v24

echo_lvmi_devereux_baseline

echo_lvmi_devereux_v24

echo_lvef_baseline

echo_lvef_v24

echo_e_lateral_septal.v0

echo_e_lateral_septal.v24

echo_tapse.v0

echo_tapse.v24

echo_srv.v0

echo_srv.v24

echo_strv_annulus.v0

echo_strv_annulus.v24

echo_ratio_ea.v0

echo_ratio_ea.v24

echo_ratio_ee.v0

echo_ratio_ee.v24

echo_gls_positive.v0

echo_gls_positive.v24

echo_lasr.v0

echo_lasr.v24

echo_lasct.v0

echo_lasct.v24

echo_lascd.v0

echo_lascd.v24

echo_hfapeff_score.v0

echo_hfapeff_score.v24

np_domein_geheugen_baseline

np_domein_aandacht_baseline

np_domein_executieffunctioneren_baseline

np_domein_geheugen_eos

np_domein_aandacht_eos

np_domein_executieffunctioneren_eos

np_dst_forward_pscore

np_dst_forward_pscore_eos

np_wf_mean_pscore np_wf_mean_pscore_eos

np_15wt_goed_pscore np_15wt_recall_pscore_age_sex_edu np_15wt_recall_pscore np_15wt_goed_pscore_eos

np_15wt_recall_pscore_age_sex_edu_eos np_15wt_recall_pscore_eos np_sdmt_mean_tscore np_sdmt_mean_tscore_eos np_tmt_a_pscore np_tmt_b_pscore np_tmt_b_a_pscore np_tmt_a_pscore_eos

np_tmt_b_pscore_eos np_tmt_b_a_pscore_eos np_wf_letter_pscore np_wf_letter_pscore_eos np_dst_backward_pscore np_dst_backward_pscore_eos GSRS_total_V0 GSRS_total_V24

/IMPUTE METHOD=AUTO NIMPUTATIONS=5 MAXPCTMISSING=NONE MAXCASEDRAWS=50 MAXPARAMDRAWS=2 MAXMODELPARAM=2000

/CONSTRAINTS gender(ROLE=IND)

/CONSTRAINTS ethnicity(ROLE=IND)

/CONSTRAINTS age_baseline(ROLE=IND)

/CONSTRAINTS hypertension(ROLE=IND)

/CONSTRAINTS cardio_hf(ROLE=IND)

/CONSTRAINTS cardio_cor(ROLE=IND)

/CONSTRAINTS diabetes_mellitus(ROLE=IND)

/CONSTRAINTS smoking(ROLE=IND)

/CONSTRAINTS alcohol(ROLE=IND)

/CONSTRAINTS kidney_dialysis(ROLE=IND)

/CONSTRAINTS transplant_cmv_donor(ROLE=IND)

```
/CONSTRAINTS transplant_cmv_participant( ROLE=IND)
/CONSTRAINTS transplant_induction_il2ra( ROLE=IND)
/CONSTRAINTS transplant_rejection( ROLE=IND)
/CONSTRAINTS med_pred( ROLE=IND)
/CONSTRAINTS med_ci( ROLE=IND)
/CONSTRAINTS med_apa( ROLE=IND)
/CONSTRAINTS bmi( ROLE=IND)
/CONSTRAINTS lab_hb_baseline( ROLE=IND)
/CONSTRAINTS lab_crp_baseline( ROLE=IND)
/CONSTRAINTS lab_ferritine_baseline( ROLE=IND)
/CONSTRAINTS lab_tsat_baseline( ROLE=IND)
/CONSTRAINTS lab_egfr_ckdepi2021cras_baseline( ROLE=IND)
  /CONSTRAINTS sixmwt_distance.v0( ROLE=DEP)
/CONSTRAINTS sixmwt_distance.v24( ROLE=DEP)
/CONSTRAINTS ftstst_mean_baseline( ROLE=DEP)
/CONSTRAINTS ftstst_mean_eos( ROLE=DEP)
/CONSTRAINTS tug_mean_baseline( ROLE=DEP)
/CONSTRAINTS tug_mean_eos( ROLE=DEP)
/CONSTRAINTS hgs_mean_baseline( ROLE=DEP)
/CONSTRAINTS hgs_mean_eos( ROLE=DEP)
/CONSTRAINTS qol_cis_sum.v0( ROLE=DEP)
/CONSTRAINTS qol_cis_sum.v24( ROLE=DEP)
/CONSTRAINTS EQindex.v0( ROLE=DEP)
/CONSTRAINTS EQindex.v24( ROLE=DEP)
  /CONSTRAINTS qol_eq5d5l_6.v0( ROLE=DEP)
/CONSTRAINTS qol_eq5d5l_6.v24( ROLE=DEP)
/CONSTRAINTS qol_dmfs_impactvanvermoeidheid.v0( ROLE=DEP)
/CONSTRAINTS qol_dmfs_directegevolgenvanvermoeidheid.v0( ROLE=DEP)
/CONSTRAINTS qol_dmfs_mentalevermoeidheid.v0( ROLE=DEP)
```

/CONSTRAINTS qol_dmfs_fysiekevermoeidheid.v0(ROLE=DEP)

/CONSTRAINTS qol_dmfs_omgaanmetvermoeidheid.v0(ROLE=DEP)

/CONSTRAINTS qol_dmfs_impactvanvermoeidheid.v24(ROLE=DEP)

/CONSTRAINTS qol_dmfs_directegevolgenvanvermoeidheid.v24(ROLE=DEP)

/CONSTRAINTS qol_dmfs_mentalevermoeidheid.v24(ROLE=DEP)

/CONSTRAINTS qol_dmfs_fysiekevermoeidheid.v24(ROLE=DEP)

/CONSTRAINTS qol_dmfs_omgaanmetvermoeidheid.v24(ROLE=DEP)

/CONSTRAINTS qol_rand36_fysiekfunctioneren_schaal.v0(ROLE=DEP)

/CONSTRAINTS qol_rand36_sociaalfunctioneren_schaal.v0(ROLE=DEP)

/CONSTRAINTS qol_rand36_mentalegezondheid_schaal.v0(ROLE=DEP)

/CONSTRAINTS qol_rand36_vitaliteit_schaal.v0(ROLE=DEP)

/CONSTRAINTS qol_rand36_pijn_schaal.v0(ROLE=DEP)

/CONSTRAINTS qol_rand36_algemeen_schaal.v0(ROLE=DEP)

/CONSTRAINTS qol_rand36_verandering_schaal.v0(ROLE=DEP)

/CONSTRAINTS qol_rand36_fysiekfunctioneren_schaal.v24(ROLE=DEP)

/CONSTRAINTS qol_rand36_sociaalfunctioneren_schaal.v24(ROLE=DEP)

/CONSTRAINTS qol_rand36_mentalegezondheid_schaal.v24(ROLE=DEP)

/CONSTRAINTS qol_rand36_vitaliteit_schaal.v24(ROLE=DEP)

/CONSTRAINTS qol_rand36_pijn_schaal.v24(ROLE=DEP)

/CONSTRAINTS qol_rand36_algemeen_schaal.v24(ROLE=DEP)

/CONSTRAINTS qol_rand36_verandering_schaal.v24(ROLE=DEP)

/CONSTRAINTS echo_ivsd.v0(ROLE=DEP)

/CONSTRAINTS echo_ivsd.v24(ROLE=DEP)

/CONSTRAINTS echo_lvidd.v0(ROLE=DEP)

/CONSTRAINTS echo_lvidd.v24(ROLE=DEP)

/CONSTRAINTS echo_lvpwd.v0(ROLE=DEP)

/CONSTRAINTS echo_lvpwd.v24(ROLE=DEP)

/CONSTRAINTS echo_lavi_baseline(ROLE=DEP)

/CONSTRAINTS echo_lavi_v24(ROLE=DEP)

/CONSTRAINTS echo_rwt_baseline(ROLE=DEP)

/CONSTRAINTS echo_rwt_v24(ROLE=DEP)

/CONSTRAINTS echo_lvmi_devereux_baseline(ROLE=DEP)

/CONSTRAINTS echo_lvmi_devereux_v24(ROLE=DEP)

/CONSTRAINTS echo_lvef_baseline(ROLE=DEP)

/CONSTRAINTS echo_lvef_v24(ROLE=DEP)

/CONSTRAINTS echo_e_lateral_septal.v0(ROLE=DEP)

/CONSTRAINTS echo_e_lateral_septal.v24(ROLE=DEP)

/CONSTRAINTS echo_tapse.v0(ROLE=DEP)

/CONSTRAINTS echo_tapse.v24(ROLE=DEP)

/CONSTRAINTS echo_srv.v0(ROLE=DEP)

/CONSTRAINTS echo_srv.v24(ROLE=DEP)

/CONSTRAINTS echo_strv_annulus.v0(ROLE=DEP)

/CONSTRAINTS echo_strv_annulus.v24(ROLE=DEP)

/CONSTRAINTS echo_ratio_ea.v0(ROLE=DEP)

/CONSTRAINTS echo_ratio_ea.v24(ROLE=DEP)

/CONSTRAINTS echo_ratio_ee.v0(ROLE=DEP)

/CONSTRAINTS echo_ratio_ee.v24(ROLE=DEP)

/CONSTRAINTS echo_gls_positive.v0(ROLE=DEP)

/CONSTRAINTS echo_gls_positive.v24(ROLE=DEP)

/CONSTRAINTS echo_lasr.v0(ROLE=DEP)

/CONSTRAINTS echo_lasr.v24(ROLE=DEP)

/CONSTRAINTS echo_lasct.v0(ROLE=DEP)

/CONSTRAINTS echo_lasct.v24(ROLE=DEP)

/CONSTRAINTS echo_lascd.v0(ROLE=DEP)

/CONSTRAINTS echo_lascd.v24(ROLE=DEP)

/CONSTRAINTS echo_hfapeff_score.v0(ROLE=DEP)

/CONSTRAINTS echo_hfapeff_score.v24(ROLE=DEP)

/CONSTRAINTS np_domein_geheugen_baseline(ROLE=DEP)

```
/CONSTRAINTS np_domein_aandacht_baseline( ROLE=DEP)
/CONSTRAINTS np_domein_executieffunctioneren_baseline( ROLE=DEP)
/CONSTRAINTS np_domein_geheugen_eos( ROLE=DEP)
/CONSTRAINTS np_domein_aandacht_eos( ROLE=DEP)
/CONSTRAINTS np_domein_executieffunctioneren_eos( ROLE=DEP)
/CONSTRAINTS np_dst_forward_pscore( ROLE=DEP)
 /CONSTRAINTS np_dst_forward_pscore_eos( ROLE=DEP)
/CONSTRAINTS np_wf_mean_pscore( ROLE=DEP)
/CONSTRAINTS np_wf_mean_pscore_eos( ROLE=DEP)
/CONSTRAINTS np_15wt_goed_pscore( ROLE=DEP)
/CONSTRAINTS np_15wt_recall_pscore_age_sex_edu( ROLE=DEP)
/CONSTRAINTS np_15wt_recall_pscore( ROLE=DEP)
/CONSTRAINTS np_15wt_goed_pscore_eos( ROLE=DEP)
/CONSTRAINTS np_15wt_recall_pscore_age_sex_edu_eos( ROLE=DEP)
/CONSTRAINTS np_15wt_recall_pscore_eos( ROLE=DEP)
/CONSTRAINTS np_sdmt_mean_tscore( ROLE=DEP)
/CONSTRAINTS np_sdmt_mean_tscore_eos( ROLE=DEP)
/CONSTRAINTS np_tmt_a_pscore( ROLE=DEP)
/CONSTRAINTS np_tmt_b_pscore( ROLE=DEP)
/CONSTRAINTS np_tmt_b_a_pscore( ROLE=DEP)
/CONSTRAINTS np_tmt_a_pscore_eos( ROLE=DEP)
/CONSTRAINTS np_tmt_b_pscore_eos( ROLE=DEP)
/CONSTRAINTS np_tmt_b_a_pscore_eos( ROLE=DEP)
 /CONSTRAINTS np_wf_letter_pscore( ROLE=DEP)
/CONSTRAINTS np_wf_letter_pscore_eos( ROLE=DEP)
/CONSTRAINTS np_dst_backward_pscore( ROLE=DEP)
/CONSTRAINTS np_dst_backward_pscore_eos( ROLE=DEP)
/CONSTRAINTS GSRS_total_V0( ROLE=DEP)
/CONSTRAINTS GSRS_total_V24( ROLE=DEP)
```

```
  /MISSINGSUMMARIES NONE
  /IMPUTATIONSUMMARIES MODELS
  /OUTFILE IMPUTATIONS=effect_safetyanalysis_imputed .


SORT CASES  BY Imputation_.
SPLIT FILE BY Imputation_.


****Back to non-imputed data for baseline characteristics and safety events****


DO IF  (gender = 2).
RECODE lab_hb_baseline (Lowest thru 7.44=1) (7.45 thru Highest=0) INTO anemia.
END IF.
EXECUTE.

DO IF  (gender = 1).
RECODE lab_hb_baseline (Lowest thru 8.07=1) (8.08 thru Highest=0) INTO anemia.
END IF.
EXECUTE.

* Date and Time Wizard: time_since_Tx.
COMPUTE  time_since_Tx=DATEDIF(date_baseline, transplant_date, "years").
VARIABLE LABELS  time_since_Tx.
VARIABLE LEVEL  time_since_Tx (SCALE).
FORMATS  time_since_Tx (F5.0).
VARIABLE WIDTH  time_since_Tx(5).
```

```
EXECUTE.

***********Baseline characteristics -> from full analysis database********************

EXAMINE VARIABLES=
   age_baseline
   bmi
   time_since_Tx
   lab_hb_baseline
   lab_mcv_baseline
   lab_ht_baseline
   lab_ferritine_baseline
   lab_tsat_baseline
   lab_ijzer_baseline
   lab_crp_baseline
   lab_egfr_ckdepi2021cras_baseline
   Urine_creat_clearance.baseline
   lab_egfr_ckdepi2021crcysc_baseline
   lab_egfr_ckdepi2012cysc_baseline
   Urine_eiwit_excr.baseline
   Urine_albumin_excr.baseline
   Urine_ngal_excr.baseline
   Urine_kim1_excr.baseline
  lab_pth_baseline
  lab_fosfaat_baseline
  Urine_phosphate_excr.baseline
  lab_calcium_baseline
  Urine_calcium_excr.baseline
```

lab_cfgf23.v0 lab_ifgf23.v0

lab_ntprobnp_baseline

lab_tropo_baseline

sixmwt_distance.v0

SixMWT_pred_baseline

Urine_creat_excr.baseline

ftstst_mean_baseline

tug_mean_baseline

hgs_mean_baseline

qol_cis_sum.v0

qol_eq5d5l_6.v0

qol_dmfs_impactvanvermoeidheid.v0

qol_dmfs_directegevolgenvanvermoeidheid.v0

qol_dmfs_mentalevermoeidheid.v0

qol_dmfs_fysiekevermoeidheid.v0

qol_dmfs_omgaanmetvermoeidheid.v0

qol_rand36_mentalegezondheid_schaal.v0

qol_rand36_vitaliteit_schaal.v0

qol_rand36_pijn_schaal.v0

qol_rand36_algemeen_schaal.v0

echo_ivsd.v0

echo_lvidd.v0

echo_lvpwd.v0

echo_lavi_baseline

echo_rwt_baseline

echo_lvmi_devereux_baseline

echo_lvef_baseline

echo_e_lateral_septal.v0

echo_tapse.v0

echo_srv.v0

echo_strv_annulus.v0

echo_ratio_ea.v0

echo_ratio_ee.v0

echo_gls_positive.v0

echo_lasr.v0

echo_lascd.v0

echo_hfapeff_score.v0

np_domein_geheugen_baseline

np_domein_executieffunctioneren_baseline

np_wf_mean_pscore

np_sdmt_mean_tscore

np_dst_backward_pscore

EQindex.v0

qol_rand36_fysiekfunctioneren_schaal.v0

qol_rand36_sociaalfunctioneren_schaal.v0

qol_rand36_verandering_schaal.v0

echo_lasct.v0

np_domein_aandacht_baseline

np_dst_forward_pscore

np_15wt_goed_pscore

np_15wt_recall_pscore_age_sex_edu

np_tmt_a_pscore

np_tmt_b_pscore

np_tmt_b_a_pscore

np_wf_letter_pscore

syst_bp

lab_urine_sodium.v0

Fractional_sodium_excr.baseline

```
  /PLOT BOXPLOT HISTOGRAM
  /STATISTICS NONE
  /CINTERVAL 95
  /MISSING LISTWISE
  /NOTOTAL.
```

Categories

```
FREQUENCIES VARIABLES= center gender ethnicity
   diabetes_mellitus hypertension anemia
   cardio_cor cardio_cor_type___1 cardio_cor_type___2 cardio_cor_type___3
cardio_cor_type___4 cardio_cor_type___5
   cardio_hf
   neuro___1 neuro___2 neuro___3 neuro___4 neuro___5 neuro___6 neuro___7 neuro___8
neuro___9 neuro___10 neuro___11 neuro___12 cva_type___1 cva_type___2 cva_type___3
cva_type___4
   haemat_benign___1 haemat_benign___2 haemat_benign___3 haemat_benign___4
haemat_benign___5 haemat_benign___6 haemat_benign___7 haemat_benign___8
haemat_benign___9 haemat_benign_other
   alcohol smoking
   kidney_gn___1 kidney_gn___2 kidney_gn___3 kidney_gn___4 kidney_gn___5
kidney_gn___6 kidney_gn___7 kidney_gn_type kidney_ns___1 kidney_ns___2
kidney_ns___3 kidney_ns___4 kidney_ns_type kidney_cyst
   kidney_other kidney_disease___1 kidney_disease___2 kidney_disease___3
kidney_disease___4 kidney_disease___5 kidney_disease___6
   kidney_disease___7 kidney_disease___8 kidney_disease___9 kidney_disease___10
kidney_renovasc
   transplant_type kidney_dialysis kidney_dialysis_type kidney_vasc_access
   transplant_rejection transplant_rejection_2 transplant_rejection_therapy___1
transplant_rejection_therapy___2 transplant_rejection_therapy___3
transplant_rejection_therapy___4 transplant_rejection_therapy___5
   transplant_rejection_therapy___6 transplant_rejection_therapy_2
```

```
 transplant_induction___1 transplant_induction___2 transplant_induction___3
transplant_induction___4 transplant_induction_il2ra transplant_induction_lda
transplant_induction_other

   med_oral_iron med_oral_iron2 med_esa med_esa2

  med_pred med_pred_dose

  med_ci med_ci_ciclo med_ci_tacro

  med_apa med_apa_mpf med_apa_aza med_apa_mpa

  med_mtori med_mtori_si med_mtori_ever

  med_ppi

   med_vitd___1 med_vitd___2 med_vitd___3 med_vitd___4 med_vitd___5 med_vitd___6
med_vitd2

  med_phos med_phosp2 med_calc___1 med_calc___2 med_calc___3 med_calc2
med_calcreg___1 med_calcreg___2 med_calcreg___3 med_calcreg___4 med_calcreg___5
med_calcreg___6 med_calcreg___7 med_calcreg___8 med_calcreg___9

    med_antihyp___1  med_antihyp___2 med_antihyp___3 med_antihyp___4
med_antihyp___5 med_antihyp___6 med_antihyp___7 med_antihyp___8 med_antihyp___9
med_antihyp_acei med_antihyp_at2a med_antihyp_cca

  med_antihyp_bb med_antihyp_loopdiu med_antihyp_psd med_antihyp_td
med_antihyp_other

  med_acd___1 med_acd___2 med_acd___3 med_acd___4 med_acd___5 med_acd___6

  echo_hfapeff_score.v0

 /ORDER=ANALYSIS.


*normal distribution*


DESCRIPTIVES VARIABLES=  lab_tropo_baseline

 lab_fosfaat_baseline

 Urine_phosphate_excr.baseline

 lab_calcium_baseline

 Urine_creat_excr.baseline

lab_egfr_ckdepi2021cras_baseline

  Urine_creat_clearance.baseline
```

lab_egfr_ckdepi2021crcysc_baseline

lab_egfr_ckdepi2012cysc_baseline

sixmwt_distance.v0

SixMWT_pred_baseline

ftstst_mean_baseline

tug_mean_baseline

hgs_mean_baseline

qol_cis_sum.v0

qol_eq5d5l_6.v0

qol_dmfs_impactvanvermoeidheid.v0

qol_dmfs_directegevolgenvanvermoeidheid.v0

qol_dmfs_mentalevermoeidheid.v0

qol_dmfs_fysiekevermoeidheid.v0

qol_dmfs_omgaanmetvermoeidheid.v0

qol_rand36_mentalegezondheid_schaal.v0

qol_rand36_vitaliteit_schaal.v0

qol_rand36_pijn_schaal.v0

qol_rand36_algemeen_schaal.v0

echo_ivsd.v0

echo_lvidd.v0

echo_lvpwd.v0

echo_lavi_baseline

echo_rwt_baseline

echo_lvmi_devereux_baseline

echo_lvef_baseline

echo_e_lateral_septal.v0

echo_tapse.v0

echo_srv.v0

echo_strv_annulus.v0

```
echo_ratio_ea.v0
echo_ratio_ee.v0
echo_gls_positive.v0
echo_lasr.v0
echo_lascd.v0
echo_hfapeff_score.v0
np_domein_geheugen_baseline
np_domein_executieffunctioneren_baseline
np_wf_mean_pscore
np_sdmt_mean_tscore
np_dst_backward_pscore
syst_bp
  lab_hb_baseline
      lab_mcv_baseline
    lab_ht_baseline
     lab_tsat_baseline
         bmi
  /STATISTICS=MEAN STDDEV MIN MAX.

*no normal distribution*

FREQUENCIES VARIABLES=EQindex.v0
qol_rand36_fysiekfunctioneren_schaal.v0
qol_rand36_sociaalfunctioneren_schaal.v0
qol_rand36_verandering_schaal.v0
 echo_lasct.v0
  np_domein_aandacht_baseline
  np_dst_forward_pscore
 np_15wt_goed_pscore
```

```
 np_15wt_recall_pscore_age_sex_edu
np_tmt_a_pscore
 np_tmt_b_pscore
 np_tmt_b_a_pscore
 np_wf_letter_pscore
   lab_ferritine_baseline
    lab_ijzer_baseline
    lab_crp_baseline
    Urine_eiwit_excr.baseline
    Urine_albumin_excr.baseline
    Urine_ngal_excr.baseline
    Urine_kim1_excr.baseline
  lab_pth_baseline
  Urine_calcium_excr.baseline
  lab_cfgf23.v0 lab_ifgf23.v0
  lab_ntprobnp_baseline
    age_baseline
    time_since_Tx
lab_urine_sodium.v0
Fractional_sodium_excr.baseline
  /FORMAT=NOTABLE
  /NTILES=4
  /STATISTICS=MEDIAN
  /ORDER=ANALYSIS.



SORT CASES  BY treatment_arm.
```

SPLIT FILE SEPARATE BY treatment_arm.

Categories

FREQUENCIES VARIABLES= center gender ethnicity

diabetes_mellitus hypertension anemia

cardio_cor cardio_cor_type___1 cardio_cor_type___2 cardio_cor_type___3 cardio_cor_type___4 cardio_cor_type___5

cardio_hf

neuro___1 neuro___2 neuro___3 neuro___4 neuro___5 neuro___6 neuro___7 neuro___8 neuro___9 neuro___10 neuro___11 neuro___12 cva_type___1 cva_type___2 cva_type___3 cva_type___4

haemat_benign___1 haemat_benign___2 haemat_benign___3 haemat_benign___4 haemat_benign___5 haemat_benign___6 haemat_benign___7 haemat_benign___8 haemat_benign___9 haemat_benign_other

alcohol smoking

kidney_gn___1 kidney_gn___2 kidney_gn___3 kidney_gn___4 kidney_gn___5 kidney_gn___6 kidney_gn___7 kidney_gn_type kidney_ns___1 kidney_ns___2 kidney_ns___3 kidney_ns___4 kidney_ns_type kidney_cyst

kidney_other kidney_disease___1 kidney_disease___2 kidney_disease___3 kidney_disease___4 kidney_disease___5 kidney_disease___6

kidney_disease___7 kidney_disease___8 kidney_disease___9 kidney_disease___10 kidney_renovasc

transplant_type kidney_dialysis kidney_dialysis_type kidney_vasc_access

transplant_rejection transplant_rejection_2 transplant_rejection_therapy___1 transplant_rejection_therapy___2 transplant_rejection_therapy___3 transplant_rejection_therapy___4 transplant_rejection_therapy___5

transplant_rejection_therapy___6 transplant_rejection_therapy_2

transplant_induction___1 transplant_induction___2 transplant_induction___3 transplant_induction___4 transplant_induction_il2ra transplant_induction_lda transplant_induction_other

med_oral_iron med_oral_iron2 med_esa med_esa2

```
  med_pred med_pred_dose
  med_ci med_ci_ciclo med_ci_tacro
  med_apa med_apa_mpf med_apa_aza med_apa_mpa
  med_mtori med_mtori_si med_mtori_ever
  med_ppi
   med_vitd___1 med_vitd___2 med_vitd___3 med_vitd___4 med_vitd___5 med_vitd___6
med_vitd2
  med_phos med_phosp2 med_calc___1 med_calc___2 med_calc___3 med_calc2
med_calcreg___1 med_calcreg___2 med_calcreg___3 med_calcreg___4 med_calcreg___5
med_calcreg___6 med_calcreg___7 med_calcreg___8 med_calcreg___9
    med_antihyp___1  med_antihyp___2 med_antihyp___3 med_antihyp___4
med_antihyp___5 med_antihyp___6 med_antihyp___7 med_antihyp___8 med_antihyp___9
med_antihyp_acei med_antihyp_at2a med_antihyp_cca
  med_antihyp_bb med_antihyp_loopdiu med_antihyp_psd med_antihyp_td
med_antihyp_other
  med_acd___1 med_acd___2 med_acd___3 med_acd___4 med_acd___5 med_acd___6
  echo_hfapeff_score.v0
 /ORDER=ANALYSIS.


*normal distribution*


DESCRIPTIVES VARIABLES=  lab_tropo_baseline
 lab_fosfaat_baseline
 Urine_phosphate_excr.baseline
 lab_calcium_baseline
 Urine_creat_excr.baseline
lab_egfr_ckdepi2021cras_baseline
   Urine_creat_clearance.baseline
   lab_egfr_ckdepi2021crcysc_baseline
   lab_egfr_ckdepi2012cysc_baseline
sixmwt_distance.v0
```

SixMWT_pred_baseline

ftstst_mean_baseline

tug_mean_baseline

hgs_mean_baseline

qol_cis_sum.v0

qol_eq5d5l_6.v0

qol_dmfs_impactvanvermoeidheid.v0

qol_dmfs_directegevolgenvanvermoeidheid.v0

qol_dmfs_mentalevermoeidheid.v0

qol_dmfs_fysiekevermoeidheid.v0

qol_dmfs_omgaanmetvermoeidheid.v0

qol_rand36_mentalegezondheid_schaal.v0

qol_rand36_vitaliteit_schaal.v0

qol_rand36_pijn_schaal.v0

qol_rand36_algemeen_schaal.v0

echo_ivsd.v0

echo_lvidd.v0

echo_lvpwd.v0

echo_lavi_baseline

echo_rwt_baseline

echo_lvmi_devereux_baseline

echo_lvef_baseline

echo_e_lateral_septal.v0

echo_tapse.v0

echo_srv.v0

echo_strv_annulus.v0

echo_ratio_ea.v0

echo_ratio_ee.v0

echo_gls_positive.v0

```
echo_lasr.v0
echo_lascd.v0
echo_hfapeff_score.v0
np_domein_geheugen_baseline
np_domein_executieffunctioneren_baseline
np_wf_mean_pscore
np_sdmt_mean_tscore
np_dst_backward_pscore
syst_bp
  lab_hb_baseline
      lab_mcv_baseline
    lab_ht_baseline
     lab_tsat_baseline
         bmi
  /STATISTICS=MEAN STDDEV MIN MAX.

*no normal distribution*

FREQUENCIES VARIABLES=EQindex.v0
qol_rand36_fysiekfunctioneren_schaal.v0
qol_rand36_sociaalfunctioneren_schaal.v0
qol_rand36_verandering_schaal.v0
 echo_lasct.v0
  np_domein_aandacht_baseline
  np_dst_forward_pscore
 np_15wt_goed_pscore
 np_15wt_recall_pscore_age_sex_edu
np_tmt_a_pscore
 np_tmt_b_pscore
```

```
 np_tmt_b_a_pscore
 np_wf_letter_pscore
   lab_ferritine_baseline
    lab_ijzer_baseline
    lab_crp_baseline
    Urine_eiwit_excr.baseline
    Urine_albumin_excr.baseline
    Urine_ngal_excr.baseline
    Urine_kim1_excr.baseline
  lab_pth_baseline
  Urine_calcium_excr.baseline
  lab_cfgf23.v0 lab_ifgf23.v0
  lab_ntprobnp_baseline
    age_baseline
    time_since_Tx
lab_urine_sodium.v0
Fractional_sodium_excr.baseline
  /FORMAT=NOTABLE
  /NTILES=4
  /STATISTICS=MEDIAN
  /ORDER=ANALYSIS.


SPLIT FILE OFF.


RECODE administr_type.v0 (2=1) (ELSE=0) INTO administration_iron.v0.
EXECUTE.
```

```
RECODE administr_type.v6 (2=1) (ELSE=0) INTO administration_iron.v6.
EXECUTE.

RECODE administr_type.v12 (2=1) (ELSE=0) INTO administration_iron.v12.
EXECUTE.

RECODE administr_type.v18 (2=1) (ELSE=0) INTO administration_iron.v18.
EXECUTE.



RECODE administr_type.v0 (1=1) (ELSE=0) INTO administration_none.v0.
EXECUTE.

RECODE administr_type.v6 (1=1) (ELSE=0) INTO administration_none.v6.
EXECUTE.

RECODE administr_type.v12 (1=1) (ELSE=0) INTO administration_none.v12.
EXECUTE.

RECODE administr_type.v18 (1=1) (ELSE=0) INTO administration_none.v18.
EXECUTE.



RECODE administr_type.v0 (3=1) (ELSE=0) INTO administration_placebo.v0.
EXECUTE.

RECODE administr_type.v6 (3=1) (ELSE=0) INTO administration_placebo.v6.
```

```
EXECUTE.

RECODE administr_type.v12 (3=1) (ELSE=0) INTO administration_placebo.v12.
EXECUTE.

RECODE administr_type.v18 (3=1) (ELSE=0) INTO administration_placebo.v18.
EXECUTE.

COMPUTE adminstration_iron_nr=administration_iron.v0 + administration_iron.v6 +
    administration_iron.v12 + administration_iron.v18.
EXECUTE.

COMPUTE adminstration_none_nr=administration_none.v0 + administration_none.v6 +
    administration_none.v12 + administration_none.v18.
EXECUTE.

COMPUTE adminstration_placebo_nr=administration_placebo.v0 +
administration_placebo.v6 +
    administration_placebo.v12 + administration_placebo.v18.
EXECUTE.

SORT CASES  BY treatment_arm.
SPLIT FILE SEPARATE BY treatment_arm.

FREQUENCIES VARIABLES=adminstration_iron_nr adminstration_none_nr
adminstration_placebo_nr
  /ORDER=ANALYSIS.


SPLIT FILE OFF.
```

```
SORT CASES  BY treatment_arm.

SPLIT FILE SEPARATE BY treatment_arm.


DATASET ACTIVATE DataSet1.

FREQUENCIES VARIABLES=administr_type.v0 administr_type.v12 administr_type.v18
administr_type.v6

    administr_none___1.v0 administr_none___1.v12 administr_none___1.v18
administr_none___1.v6

    administr_none___2.v0 administr_none___2.v12 administr_none___2.v18
administr_none___2.v6

    administr_none___3 administr_none___4 administr_none___5.v0
administr_none___5.v12

    administr_none___5.v18 administr_none___5.v6 administr_none___6.v0
administr_none___6.v12

    administr_none___6.v18 administr_none___6.v6 administr_none2
administr_placebo_why___1.v0

    administr_placebo_why___1.v12 administr_placebo_why___1.v18
administr_placebo_why___1.v6

    administr_placebo_why___2 administr_placebo_why2 administr_placebo_why___3.v0

    administr_placebo_why___3.v12 administr_placebo_why___3.v18
administr_placebo_why___3.v6

  /ORDER=ANALYSIS.


SPLIT FILE OFF.



***********Safety analysis baseline characteristics and incidence of events -> from safety
analysis database******************
```

```
*Categories*

FREQUENCIES VARIABLES=
    restless_legs
  health_event___4 health_event_cv___1 health_event_cv___3 health_event_cv___5
health_event_cv___6 health_event_cv___7  health_event_cv___8 health_event_cv___9
health_event_cv___10 health_event_cv___11 health_event_cv2
  health_event_ren health_event_surg health_event_frac health_event_haem2
 /ORDER=ANALYSIS.

*normal distribution*

DESCRIPTIVES VARIABLES=
 lab_asat_baseline
lab_alat_baseline
 /STATISTICS=MEAN STDDEV MIN MAX.

*no normal destribution*

FREQUENCIES VARIABLES= GSRS_total_V0
 /FORMAT=NOTABLE
 /NTILES=4
 /STATISTICS=MEDIAN
 /ORDER=ANALYSIS.

SORT CASES  BY treatment_arm.
SPLIT FILE SEPARATE BY treatment_arm.

*Categories*
```

```
FREQUENCIES VARIABLES=
    restless_legs
  health_event___4 health_event_cv___1 health_event_cv___3 health_event_cv___5
health_event_cv___6 health_event_cv___7  health_event_cv___8 health_event_cv___9
health_event_cv___10 health_event_cv___11 health_event_cv2
  health_event_ren health_event_surg health_event_frac health_event_haem2
  /ORDER=ANALYSIS.

*normal distribution*

DESCRIPTIVES VARIABLES=
  lab_asat_baseline
lab_alat_baseline
  /STATISTICS=MEAN STDDEV MIN MAX.

*no normal distribution*

FREQUENCIES VARIABLES= GSRS_total_V0
  /FORMAT=NOTABLE
  /NTILES=4
  /STATISTICS=MEDIAN
  /ORDER=ANALYSIS.

SPLIT FILE OFF.

RECODE lab_fosfaat_t6 (Lowest thru 0.49999=1) (0.5 thru Highest=0) INTO
event_fosfaat_0.5_v6.
EXECUTE.
```

```
RECODE lab_fosfaat_t12 (Lowest thru 0.49999=1) (0.5 thru Highest=0) INTO
event_fosfaat_0.5_v12.

EXECUTE.


RECODE lab_fosfaat_t18 (Lowest thru 0.49999=1) (0.5 thru Highest=0) INTO
event_fosfaat_0.5_v18.

EXECUTE.


RECODE lab_fosfaat_eos (Lowest thru 0.49999=1) (0.5 thru Highest=0) INTO
event_fosfaat_0.5_v24.

EXECUTE.


RECODE lab_fosfaat_t6 (Lowest thru 0.349999=1) (0.35 thru Highest=0) INTO
event_fosfaat_0.35_v6.

EXECUTE.


RECODE lab_fosfaat_t12 (Lowest thru 0.349999=1) (0.35 thru Highest=0) INTO
event_fosfaat_0.35_v12.

EXECUTE.


RECODE lab_fosfaat_t18 (Lowest thru 0.349999=1) (0.35 thru Highest=0) INTO
event_fosfaat_0.35_v18.

EXECUTE.


RECODE lab_fosfaat_eos (Lowest thru 0.349999=1) (0.35 thru Highest=0) INTO
event_fosfaat_0.35_v24.

EXECUTE.


SORT CASES  BY administr_type.v0.

SPLIT FILE LAYERED BY administr_type.v0.
```

```
FREQUENCIES VARIABLES=
    previous_visit_se___1.v6
    previous_visit_se___2.v6
    previous_visit_se___3.v6
    previous_visit_se___4.v6
    previous_visit_se___5.v6
    previous_visit_se___6.v6
    previous_visit_se___7.v6
    previous_visit_se___8.v6
    previous_visit_se___9.v6
    previous_visit_se___10.v6
    previous_visit_se___11.v6
    previous_visit_se_free.v6
    administr_se___4
    administr_se___5
    administr_se___6
    administr_se___7
    administr_se___8
    administr_se___1.v0
    administr_se___2.v0
    administr_se___3.v0
    administr_se___9.v0
    administr_se___10.v0
    administr_se2.v0
  /ORDER=ANALYSIS.


SORT CASES  BY administr_type.v6.
SPLIT FILE LAYERED BY administr_type.v6.
```

```
FREQUENCIES VARIABLES=
   previous_visit_se___1.v12
   previous_visit_se___2.v12
   previous_visit_se___3.v12
   previous_visit_se___4.v12
   previous_visit_se___5.v12
   previous_visit_se___6.v12
   previous_visit_se___7.v12
   previous_visit_se___8.v12
   previous_visit_se___9.v12
   previous_visit_se___10.v12
   previous_visit_se___11.v12
   previous_visit_se_free.v12
   administr_se___4
   administr_se___5
   administr_se___6
   administr_se___7
   administr_se___8
   administr_se___1.v6
   administr_se___2.v6
   administr_se___3.v6
   administr_se___9.v6
   administr_se___10.v6
   administr_se2.v6
  /ORDER=ANALYSIS.
```

```
SORT CASES  BY administr_type.v12.
SPLIT FILE LAYERED BY administr_type.v12.


FREQUENCIES VARIABLES=
  previous_visit_se___1.v18
  previous_visit_se___2.v18
  previous_visit_se___3.v18
  previous_visit_se___4.v18
  previous_visit_se___5.v18
  previous_visit_se___6.v18
  previous_visit_se___7.v18
  previous_visit_se___8.v18
  previous_visit_se___9.v18
  previous_visit_se___10.v18
  previous_visit_se___11.v18
  previous_visit_se_free.v18
  administr_se___4
  administr_se___5
  administr_se___6
  administr_se___7
  administr_se___8
  administr_se___1.v12
  administr_se___2.v12
  administr_se___3.v12
  administr_se___9.v12
  administr_se___10.v12
  administr_se2.v12
 /ORDER=ANALYSIS.
```

```
SORT CASES  BY administr_type.v18.
SPLIT FILE LAYERED BY administr_type.v18.

FREQUENCIES VARIABLES=
  previous_visit_se___1.v24
  previous_visit_se___2.v24
  previous_visit_se___3.v24
  previous_visit_se___4.v24
  previous_visit_se___5.v24
  previous_visit_se___6.v24
  previous_visit_se___7.v24
  previous_visit_se___8.v24
  previous_visit_se___9.v24
  previous_visit_se___10.v24
  previous_visit_se___11.v24
  previous_visit_se_free.v24
  administr_se___4
  administr_se___5
  administr_se___6
  administr_se___7
  administr_se___8
  administr_se___1.v18
  administr_se___2.v18
  administr_se___3.v18
  administr_se___9.v18
  administr_se___10.v18
  administr_se2.v18
```

```
 /ORDER=ANALYSIS.



SORT CASES  BY treatment_arm.
SPLIT FILE LAYERED BY treatment_arm.

FREQUENCIES VARIABLES=
health_event___4
health_event_cv___1
health_event_cv___3
health_event_cv___5
health_event_cv___6
health_event_cv___7
health_event_cv___8
health_event_cv___9
health_event_cv___10
health_event_cv___11
health_event_cv2
health_event_ren
health_event_surg
health_event_frac
health_event_haem2
health_event___1.v6
health_event___2.v6
health_event___3.v6
health_event___5.v6
health_event___6.v6
health_event___7.v6
```

health_event___8.v6

health_event___9.v6

health_event_ha.v6

health_event_inf___1.v6

health_event_inf___2.v6

health_event_inf___3.v6

health_event_inf___4.v6

health_event_inf___5.v6

health_event_inf___6.v6

health_event_inf2.v6

health_event_inf_cult.v6

health_event_inf_cult2.v6

health_event_cv___2.v6

health_event_cv___4.v6

health_event_haem___1.v6

health_event_haem___2.v6

health_event_haem___3.v6

health_event_haem___4.v6

health_event_free.v6

ae_serious___2

ae_serious___5

ae_serious___6

ae_serious___7

ae_nonserious___4

ae_nonserious___7

ae_serious___1.v6

ae_serious___3.v6

ae_serious___4.v6

ae_serious___8.v6

ae_serious_free.v6

ae_serious_date.v6

ae_nonserious___1.v6

ae_nonserious___2.v6

ae_nonserious___3.v6

ae_nonserious___5.v6

ae_nonserious___6.v6

ae_nonserious___8.v6

ae_nonserious_free.v6

ae_nonserious_date.v6

event_fosfaat_0.5_v6

event_fosfaat_0.35_v6

/ORDER=ANALYSIS.

FREQUENCIES VARIABLES=

health_event___1.v12

health_event___2.v12

health_event___3.v12

health_event___5.v12

health_event___6.v12

health_event___7.v12

health_event___8.v12

health_event___9.v12

health_event_ha.v12

health_event_inf___1.v12

health_event_inf___2.v12

health_event_inf___3.v12

health_event_inf___4.v12

health_event_inf___5.v12

```
health_event_inf___6.v12
health_event_inf2.v12
health_event_inf_cult.v12
health_event_inf_cult2.v12
health_event_cv___2.v12
health_event_cv___4.v12
health_event_haem___1.v12
health_event_haem___2.v12
health_event_haem___3.v12
health_event_haem___4.v12
health_event_free.v12
ae_serious___1.v12
ae_serious___3.v12
ae_serious___4.v12
ae_serious___8.v12
ae_serious_free.v12
ae_serious_date.v12
ae_nonserious___1.v12
ae_nonserious___2.v12
ae_nonserious___3.v12
ae_nonserious___5.v12
ae_nonserious___6.v12
ae_nonserious___8.v12
ae_nonserious_free.v12
ae_nonserious_date.v12
event_fosfaat_0.5_v12
event_fosfaat_0.35_v12
  /ORDER=ANALYSIS.
```

FREQUENCIES VARIABLES=

health_event___1.v18

health_event___2.v18

health_event___3.v18

health_event___5.v18

health_event___6.v18

health_event___7.v18

health_event___8.v18

health_event___9.v18

health_event_ha.v18

health_event_inf___1.v18

health_event_inf___2.v18

health_event_inf___3.v18

health_event_inf___4.v18

health_event_inf___5.v18

health_event_inf___6.v18

health_event_inf2.v18

health_event_inf_cult.v18

health_event_inf_cult2.v18

health_event_cv___2.v18

health_event_cv___4.v18

health_event_haem___1.v18

health_event_haem___2.v18

health_event_haem___3.v18

health_event_haem___4.v18

health_event_free.v18

ae_serious___1.v18

ae_serious___3.v18

ae_serious___4.v18

```
ae_serious___8.v18
ae_serious_free.v18
ae_serious_date.v18
ae_nonserious___1.v18
ae_nonserious___2.v18
ae_nonserious___3.v18
ae_nonserious___5.v18
ae_nonserious___6.v18
ae_nonserious___8.v18
ae_nonserious_free.v18
ae_nonserious_date.v18
event_fosfaat_0.5_v18
event_fosfaat_0.35_v18
  /ORDER=ANALYSIS.

FREQUENCIES VARIABLES=
health_event___1.v24
health_event___2.v24
health_event___3.v24
health_event___5.v24
health_event___6.v24
health_event___7.v24
health_event___8.v24
health_event___9.v24
health_event_ha.v24
health_event_inf___1.v24
health_event_inf___2.v24
health_event_inf___3.v24
health_event_inf___4.v24
```

```
health_event_inf___5.v24
health_event_inf___6.v24
health_event_inf2.v24
health_event_inf_cult.v24
health_event_inf_cult2.v24
health_event_cv___2.v24
health_event_cv___4.v24
health_event_haem___1.v24
health_event_haem___2.v24
health_event_haem___3.v24
health_event_haem___4.v24
health_event_free.v24
ae_serious___1.v24
ae_serious___3.v24
ae_serious___4.v24
ae_serious___8.v24
ae_serious_free.v24
ae_serious_date.v24
ae_nonserious___1.v24
ae_nonserious___2.v24
ae_nonserious___3.v24
ae_nonserious___5.v24
ae_nonserious___6.v24
ae_nonserious___8.v24
ae_nonserious_free.v24
ae_nonserious_date.v24
event_fosfaat_0.5_v24
event_fosfaat_0.35_v24
  /ORDER=ANALYSIS.
```

```
SPLIT FILE OFF.

*****Mixed Models analyses*****

COMPUTE hb_baseline=lab_hb_baseline.
EXECUTE.

COMPUTE ferritine_baseline=lab_ferritine_baseline.
EXECUTE.

COMPUTE lab_ferritine_t6=lab_ferritine_blinded.v6.
EXECUTE.

COMPUTE lab_ferritine_t12=lab_ferritine_blinded.v12.
EXECUTE.

COMPUTE lab_ferritine_t18=lab_ferritine_blinded.v18.
EXECUTE.

COMPUTE lab_ferritine_eos=lab_ferritine_blinded.v24.
EXECUTE.

   COMPUTE tsat_baseline=lab_tsat_baseline.
EXECUTE.

COMPUTE lab_tsat_t6=lab_tsat_blinded.v6.
EXECUTE.
```

```
COMPUTE lab_tsat_t12=lab_tsat_blinded.v12.
EXECUTE.

COMPUTE lab_tsat_t18=lab_tsat_blinded.v18.
EXECUTE.

COMPUTE lab_tsat_eos=lab_tsat_blinded.v24.
EXECUTE.

COMPUTE ijzer_baseline=lab_ijzer_baseline.
EXECUTE.

COMPUTE lab_ijzer_t6=lab_ijzer_blinded.v6.
EXECUTE.

COMPUTE lab_ijzer_t12=lab_ijzer_blinded.v12.
EXECUTE.

COMPUTE lab_ijzer_t18=lab_ijzer_blinded.v18.
EXECUTE.

COMPUTE lab_ijzer_eos=lab_ijzer_blinded.v24.
EXECUTE.

COMPUTE ht_baseline=lab_ht_baseline.
EXECUTE.

COMPUTE mcv_baseline=lab_mcv_baseline.
```

```
EXECUTE.

COMPUTE createxcr_baseline=Urine_creat_excr.baseline.
EXECUTE.

COMPUTE egfr_baseline=lab_egfr_ckdepi2021cras_baseline.
EXECUTE.

COMPUTE egfr_crcysc_baseline=lab_egfr_ckdepi2021crcysc_baseline.
EXECUTE.

COMPUTE egfr_cysc_baseline=lab_egfr_ckdepi2012cysc_baseline.
EXECUTE.


COMPUTE Creatclearance_baseline=Urine_creat_clearance.baseline.
EXECUTE.

COMPUTE eiwit_excr.baseline=Urine_eiwit_excr.baseline.
EXECUTE.

COMPUTE albumin_excr.baseline=Urine_albumin_excr.baseline.
EXECUTE.

COMPUTE ngal_excr_baseline=Urine_ngal_excr.baseline.
EXECUTE.

COMPUTE kim1_excr_baseline=Urine_kim1_excr.baseline.
EXECUTE.
```

```
COMPUTE lab_tropo_t6=1.
EXECUTE.

RECODE lab_tropo_t6 (1=SYSMIS).
EXECUTE.

COMPUTE lab_tropo_t18=1.
EXECUTE.

RECODE lab_tropo_t18 (1=SYSMIS).
EXECUTE.

COMPUTE lab_ntprobnp_t6=1.
EXECUTE.

RECODE lab_ntprobnp_t6 (1=SYSMIS).
EXECUTE.

COMPUTE lab_ntprobnp_t18=1.
EXECUTE.

RECODE lab_ntprobnp_t18 (1=SYSMIS).
EXECUTE.

COMPUTE tropo_baseline=lab_tropo_baseline.
EXECUTE.

COMPUTE ntprobnp_baseline=lab_ntprobnp_baseline.
```

```
EXECUTE.

COMPUTE Phosphateexcr_baseline=Urine_phosphate_excr.baseline.
EXECUTE.

COMPUTE Calciumexcr_baseline=Urine_calcium_excr.baseline.
EXECUTE.

COMPUTE pth_baseline=lab_pth_baseline.
EXECUTE.

COMPUTE fosfaat_baseline=lab_fosfaat_baseline.
EXECUTE.

COMPUTE calcium_baseline=lab_calcium_baseline.
EXECUTE.

COMPUTE asat_baseline=lab_asat_baseline.
EXECUTE.

COMPUTE alat_baseline=lab_alat_baseline.
EXECUTE.

COMPUTE totalfgf23_baseline=lab_cfgf23.v0.
EXECUTE.

COMPUTE ifgf23_baseline=lab_ifgf23.v0.
EXECUTE.
```

```
COMPUTE tacro_baseline=lab_tacro_baseline.
EXECUTE.

COMPUTE urinenatrium_baseline=lab_urine_sodium.v0.
EXECUTE.

COMPUTE urinekalium_baseline=lab_urine_potassium.v0.
EXECUTE.

COMPUTE urinemagnesium_baseline=lab_urine_magnesium.v0.
EXECUTE.

COMPUTE fena_baseline=Fractional_sodium_excr.baseline.
EXECUTE.

COMPUTE syst_bp_baseline=syst_bp.
EXECUTE.

COMPUTE diast_bp_baseline=diast_bp.
EXECUTE.

VARSTOCASES
  /ID=id
    /MAKE ferritine FROM lab_ferritine_baseline lab_ferritine_t6 lab_ferritine_t12
lab_ferritine_t18 lab_ferritine_eos
  /MAKE tsat FROM lab_tsat_baseline lab_tsat_t6 lab_tsat_t12 lab_tsat_t18 lab_tsat_eos
    /MAKE ijzer FROM lab_ijzer_baseline lab_ijzer_t6 lab_ijzer_t12 lab_ijzer_t18
lab_ijzer_eos
  /MAKE hb FROM lab_hb_baseline lab_hb_t6 lab_hb_t12 lab_hb_t18 lab_hb_eos
    /MAKE ht FROM lab_ht_baseline lab_ht_t6 lab_ht_t12 lab_ht_t18 lab_ht_eos
```

/MAKE mcv FROM lab_mcv_baseline lab_mcv_t6 lab_mcv_t12 lab_mcv_t18 lab_mcv_eos

/MAKE urinecreatexcr FROM Urine_creat_excr.baseline Urine_creat_excr.t6 Urine_creat_excr.t12 Urine_creat_excr.t18 Urine_creat_excr.eos

/MAKE egfr FROM lab_egfr_ckdepi2021cras_baseline lab_egfr_ckdepi2021cras_t6 lab_egfr_ckdepi2021cras_t12 lab_egfr_ckdepi2021cras_t18 lab_egfr_ckdepi2021cras_eos

/MAKE egfr_crcysc FROM lab_egfr_ckdepi2021crcysc_baseline lab_egfr_ckdepi2021crcysc_t6 lab_egfr_ckdepi2021crcysc_t12 lab_egfr_ckdepi2021crcysc_t18 lab_egfr_ckdepi2021crcysc_eos

/MAKE egfr_cysc FROM lab_egfr_ckdepi2012cysc_baseline lab_egfr_ckdepi2012cysc_v6 lab_egfr_ckdepi2012cysc_v12 lab_egfr_ckdepi2012cysc_v18 lab_egfr_ckdepi2012cysc_v24

/MAKE Urine_creat_clearance FROM Urine_creat_clearance.baseline Urine_creat_clearance.t6 Urine_creat_clearance.t12 Urine_creat_clearance.t18 Urine_creat_clearance.eos

/MAKE eiwit_excr FROM Urine_eiwit_excr.baseline Urine_eiwit_excr.t6 Urine_eiwit_excr.t12 Urine_eiwit_excr.t18 Urine_eiwit_excr.eos

/MAKE albumin_excr FROM Urine_albumin_excr.baseline Urine_albumin_excr.t6 Urine_albumin_excr.t12 Urine_albumin_excr.t18 Urine_albumin_excr.eos

/MAKE urinengalexcr FROM Urine_ngal_excr.baseline Urine_ngal_excr.t6 Urine_ngal_excr.t12 Urine_ngal_excr.t18 Urine_ngal_excr.eos

/MAKE urinekim1excr FROM Urine_kim1_excr.baseline Urine_kim1_excr.t6 Urine_kim1_excr.t12 Urine_kim1_excr.t18 Urine_kim1_excr.eos

/MAKE totalfgf23 FROM lab_cfgf23.v0 lab_cfgf23.v6 lab_cfgf23.v12 lab_cfgf23.v18 lab_cfgf23.v24

/MAKE ifgf23 FROM lab_ifgf23.v0 lab_ifgf23.v6 lab_ifgf23.v12 lab_ifgf23.v18 lab_ifgf23.v24

/MAKE fosfaat FROM lab_fosfaat_baseline lab_fosfaat_t6 lab_fosfaat_t12 lab_fosfaat_t18 lab_fosfaat_eos

/MAKE urinefosfaatexcr FROM Urine_phosphate_excr.baseline Urine_phosphate_excr.t6 Urine_phosphate_excr.t12 Urine_phosphate_excr.t18 Urine_phosphate_excr.eos

/MAKE calcium FROM lab_calcium_baseline lab_calcium_t6 lab_calcium_t12 lab_calcium_t18 lab_calcium_eos

/MAKE urinecalciumexcr FROM Urine_calcium_excr.baseline Urine_calcium_excr.t6 Urine_calcium_excr.t12 Urine_calcium_excr.t18 Urine_calcium_excr.eos

/MAKE pth FROM lab_pth_baseline lab_pth_t6 lab_pth_t12 lab_pth_t18 lab_pth_eos

```
  /MAKE troponine FROM lab_tropo_baseline lab_tropo_t6 lab_tropo_t12 lab_tropo_t18
lab_tropo_eos
  /MAKE ntprobnp FROM lab_ntprobnp_baseline lab_ntprobnp_t6 lab_ntprobnp_t12
lab_ntprobnp_t18 lab_ntprobnp_eos
  /MAKE syst_bp FROM syst_bp syst_bp_fu.v6 syst_bp_fu.v12 syst_bp_fu.v18
syst_bp_fu.v24
  /MAKE urinenatrium FROM lab_urine_sodium.v0 lab_urine_sodium.v6
lab_urine_sodium.v12 lab_urine_sodium.v18 lab_urine_sodium.v24
  /MAKE fena FROM Fractional_sodium_excr.baseline Fractional_sodium_excr.t6
Fractional_sodium_excr.t12 Fractional_sodium_excr.t18 Fractional_sodium_excr.eos
  /MAKE asat FROM lab_asat_baseline lab_asat_t6 lab_asat_t12 lab_asat_t18 lab_asat_eos
  /MAKE alat FROM lab_alat_baseline lab_alat_t6 lab_alat_t12 lab_alat_t18 lab_alat_eos
  /INDEX=Visite(5)
  /KEEP=study_id
ferritine_baseline
tsat_baseline
ijzer_baseline
hb_baseline
ht_baseline
mcv_baseline
createxcr_baseline
egfr_baseline
egfr_crcysc_baseline
egfr_cysc_baseline
Createclearance_baseline
eiwit_excr.baseline
albumin_excr.baseline
kim1_excr_baseline
ngal_excr_baseline
totalfgf23_baseline
ifgf23_baseline
```

```
fosfaat_baseline
calcium_baseline
Phosphateexcr_baseline
Calciumexcr_baseline
pth_baseline
ntprobnp_baseline
tropo_baseline
syst_bp_baseline
urinenatrium_baseline
fena_baseline
asat_baseline
alat_baseline
gender
center
ethnicity
age_baseline
treatment_arm
  /NULL=KEEP.


RECODE Visite (1=0) (2=6) (3=12) (4=18) (5=24) INTO Tijd.
EXECUTE.

COMPUTE Tijd_tot_eos=24 - Tijd.
EXECUTE.

**Save as new database**

COMPUTE ln_ferritine=LN(ferritine).
```

```
EXECUTE.

COMPUTE ln_ijzer=LN(ijzer).
EXECUTE.

COMPUTE ln_urinecreatexcr=LN(urinecreatexcr).
EXECUTE.

COMPUTE ln_eiwit_excr=LN(eiwit_excr).
EXECUTE.

COMPUTE ln_albumin_excr=LN(albumin_excr).
EXECUTE.

COMPUTE ln_urinekim1excr=LN(urinekim1excr).
EXECUTE.

COMPUTE urinengalexcr_newunit=urinengalexcr / 1000.
EXECUTE.

COMPUTE ln_urinengalexcr=LN(urinengalexcr_newunit).
EXECUTE.


COMPUTE ln_troponine=LN(troponine).
EXECUTE.

COMPUTE ln_ntprobnp=LN(ntprobnp).
EXECUTE.
```

```
COMPUTE ln_totalfgf23=LN(totalfgf23).
EXECUTE.

COMPUTE ln_ifgf23=LN(ifgf23).
EXECUTE.

COMPUTE ln_urinecalciumexcr=LN(urinecalciumexcr).
EXECUTE.

COMPUTE ln_pth=LN(pth).
EXECUTE.

COMPUTE ln_urinenatrium=LN(urinenatrium).
EXECUTE.

COMPUTE ln_fena=LN(fena).
EXECUTE.

********************

MIXED ln_ferritine BY center treatment_arm Tijd_tot_eos
  /CRITERIA=DFMETHOD(SATTERTHWAITE) CIN(95) MXITER(100) MXSTEP(10)
SCORING(1)
    SINGULAR(0.000000000001) HCONVERGE(0.00000001, RELATIVE)
LCONVERGE(0, ABSOLUTE) PCONVERGE(0,
    ABSOLUTE)
  /FIXED=center treatment_arm Tijd_tot_eos treatment_arm*Tijd_tot_eos | SSTYPE(3)
  /METHOD=REML
  /PRINT=SOLUTION
```

```
  /RANDOM=INTERCEPT | SUBJECT(id) COVTYPE(VC)
  /REPEATED=Tijd_tot_eos | SUBJECT(id) COVTYPE(TP)
  /EMMEANS=TABLES(treatment_arm*Tijd_tot_eos) .

MIXED tsat BY center treatment_arm Tijd_tot_eos
  /CRITERIA=DFMETHOD(SATTERTHWAITE) CIN(95) MXITER(100) MXSTEP(10)
SCORING(1)
    SINGULAR(0.000000000001) HCONVERGE(0.00000001, RELATIVE)
LCONVERGE(0, ABSOLUTE) PCONVERGE(0,
    ABSOLUTE)
  /FIXED=center treatment_arm Tijd_tot_eos treatment_arm*Tijd_tot_eos | SSTYPE(3)
  /METHOD=REML
  /PRINT=SOLUTION
  /RANDOM=INTERCEPT | SUBJECT(id) COVTYPE(VC)
  /REPEATED=Tijd_tot_eos | SUBJECT(id) COVTYPE(TP)
  /EMMEANS=TABLES(treatment_arm*Tijd_tot_eos) .

MIXED ln_ijzer BY center treatment_arm Tijd_tot_eos
  /CRITERIA=DFMETHOD(SATTERTHWAITE) CIN(95) MXITER(100) MXSTEP(10)
SCORING(1)
    SINGULAR(0.000000000001) HCONVERGE(0.00000001, RELATIVE)
LCONVERGE(0, ABSOLUTE) PCONVERGE(0,
    ABSOLUTE)
  /FIXED=center treatment_arm Tijd_tot_eos treatment_arm*Tijd_tot_eos | SSTYPE(3)
  /METHOD=REML
  /PRINT=SOLUTION
  /RANDOM=INTERCEPT | SUBJECT(id) COVTYPE(VC)
  /REPEATED=Tijd_tot_eos | SUBJECT(id) COVTYPE(TP)
  /EMMEANS=TABLES(treatment_arm*Tijd_tot_eos) .
```

```
MIXED hb BY center treatment_arm Tijd_tot_eos
  /CRITERIA=DFMETHOD(SATTERTHWAITE) CIN(95) MXITER(100) MXSTEP(10)
SCORING(1)
    SINGULAR(0.000000000001) HCONVERGE(0.00000001, RELATIVE)
LCONVERGE(0, ABSOLUTE) PCONVERGE(0,
    ABSOLUTE)
  /FIXED=center treatment_arm Tijd_tot_eos treatment_arm*Tijd_tot_eos | SSTYPE(3)
  /METHOD=REML
  /PRINT=SOLUTION
  /RANDOM=INTERCEPT | SUBJECT(id) COVTYPE(VC)
  /REPEATED=Tijd_tot_eos | SUBJECT(id) COVTYPE(UN)
  /EMMEANS=TABLES(treatment_arm*Tijd_tot_eos) .

MIXED ht BY center treatment_arm Tijd_tot_eos
  /CRITERIA=DFMETHOD(SATTERTHWAITE) CIN(95) MXITER(100) MXSTEP(10)
SCORING(1)
    SINGULAR(0.000000000001) HCONVERGE(0.00000001, RELATIVE)
LCONVERGE(0, ABSOLUTE) PCONVERGE(0,
    ABSOLUTE)
  /FIXED=center treatment_arm Tijd_tot_eos treatment_arm*Tijd_tot_eos | SSTYPE(3)
  /METHOD=REML
  /PRINT=SOLUTION
  /RANDOM=INTERCEPT | SUBJECT(id) COVTYPE(VC)
  /REPEATED=Tijd_tot_eos | SUBJECT(id) COVTYPE(TPH)
  /EMMEANS=TABLES(treatment_arm*Tijd_tot_eos) .

MIXED mcv BY center treatment_arm Tijd_tot_eos
  /CRITERIA=DFMETHOD(SATTERTHWAITE) CIN(95) MXITER(100) MXSTEP(10)
SCORING(1)
```

```
    SINGULAR(0.000000000001) HCONVERGE(0.00000001, RELATIVE)
LCONVERGE(0, ABSOLUTE) PCONVERGE(0,
    ABSOLUTE)
  /FIXED=center treatment_arm Tijd_tot_eos treatment_arm*Tijd_tot_eos | SSTYPE(3)
  /METHOD=REML
  /PRINT=SOLUTION
  /RANDOM=INTERCEPT | SUBJECT(id) COVTYPE(VC)
  /REPEATED=Tijd_tot_eos | SUBJECT(id) COVTYPE(TP)
  /EMMEANS=TABLES(treatment_arm*Tijd_tot_eos) .

MIXED ln_urinecreatexcr BY center treatment_arm Tijd_tot_eos
  /CRITERIA=DFMETHOD(SATTERTHWAITE) CIN(95) MXITER(100) MXSTEP(10)
SCORING(1)
    SINGULAR(0.000000000001) HCONVERGE(0.00000001, RELATIVE)
LCONVERGE(0, ABSOLUTE) PCONVERGE(0,
    ABSOLUTE)
  /FIXED=center treatment_arm Tijd_tot_eos treatment_arm*Tijd_tot_eos | SSTYPE(3)
  /METHOD=REML
  /PRINT=SOLUTION
  /RANDOM=INTERCEPT | SUBJECT(id) COVTYPE(VC)
  /REPEATED=Tijd_tot_eos | SUBJECT(id) COVTYPE(UN)
  /EMMEANS=TABLES(treatment_arm*Tijd_tot_eos) .

**

MIXED egfr BY center treatment_arm Tijd_tot_eos
  /CRITERIA=DFMETHOD(SATTERTHWAITE) CIN(95) MXITER(100) MXSTEP(10)
SCORING(1)
    SINGULAR(0.000000000001) HCONVERGE(0.00000001, RELATIVE)
LCONVERGE(0, ABSOLUTE) PCONVERGE(0,
    ABSOLUTE)
```

```
  /FIXED=center treatment_arm Tijd_tot_eos treatment_arm*Tijd_tot_eos | SSTYPE(3)
  /METHOD=REML
  /PRINT=SOLUTION
  /RANDOM=INTERCEPT | SUBJECT(id) COVTYPE(VC)
  /REPEATED=Tijd_tot_eos | SUBJECT(id) COVTYPE(UN)
  /EMMEANS=TABLES(treatment_arm*Tijd_tot_eos) .

MIXED egfr_crcysc BY center treatment_arm Tijd_tot_eos
  /CRITERIA=DFMETHOD(SATTERTHWAITE) CIN(95) MXITER(100) MXSTEP(10)
SCORING(1)
    SINGULAR(0.000000000001) HCONVERGE(0.00000001, RELATIVE)
LCONVERGE(0, ABSOLUTE) PCONVERGE(0,
    ABSOLUTE)
  /FIXED=center treatment_arm Tijd_tot_eos treatment_arm*Tijd_tot_eos | SSTYPE(3)
  /METHOD=REML
  /PRINT=SOLUTION
  /RANDOM=INTERCEPT | SUBJECT(id) COVTYPE(VC)
  /REPEATED=Tijd_tot_eos | SUBJECT(id) COVTYPE(UN)
  /EMMEANS=TABLES(treatment_arm*Tijd_tot_eos) .

MIXED egfr_cysc BY center treatment_arm Tijd_tot_eos
  /CRITERIA=DFMETHOD(SATTERTHWAITE) CIN(95) MXITER(100) MXSTEP(10)
SCORING(1)
    SINGULAR(0.000000000001) HCONVERGE(0.00000001, RELATIVE)
LCONVERGE(0, ABSOLUTE) PCONVERGE(0,
    ABSOLUTE)
  /FIXED=center treatment_arm Tijd_tot_eos treatment_arm*Tijd_tot_eos | SSTYPE(3)
  /METHOD=REML
  /PRINT=SOLUTION
  /RANDOM=INTERCEPT | SUBJECT(id) COVTYPE(VC)
```

```
  /REPEATED=Tijd_tot_eos | SUBJECT(id) COVTYPE(UN)
  /EMMEANS=TABLES(treatment_arm*Tijd_tot_eos) .

MIXED Urine_creat_clearance BY center treatment_arm Tijd_tot_eos
  /CRITERIA=DFMETHOD(SATTERTHWAITE) CIN(95) MXITER(100) MXSTEP(10)
SCORING(1)
    SINGULAR(0.000000000001) HCONVERGE(0.00000001, RELATIVE)
LCONVERGE(0, ABSOLUTE) PCONVERGE(0,
    ABSOLUTE)
  /FIXED=center treatment_arm Tijd_tot_eos treatment_arm*Tijd_tot_eos | SSTYPE(3)
  /METHOD=REML
  /PRINT=SOLUTION
  /RANDOM=INTERCEPT | SUBJECT(id) COVTYPE(VC)
  /REPEATED=Tijd_tot_eos | SUBJECT(id) COVTYPE(TPH)
  /EMMEANS=TABLES(treatment_arm*Tijd_tot_eos) .

MIXED ln_eiwit_excr BY center treatment_arm Tijd_tot_eos
  /CRITERIA=DFMETHOD(SATTERTHWAITE) CIN(95) MXITER(100) MXSTEP(10)
SCORING(1)
    SINGULAR(0.000000000001) HCONVERGE(0.00000001, RELATIVE)
LCONVERGE(0, ABSOLUTE) PCONVERGE(0,
    ABSOLUTE)
  /FIXED=center treatment_arm Tijd_tot_eos treatment_arm*Tijd_tot_eos | SSTYPE(3)
  /METHOD=REML
  /PRINT=SOLUTION
  /RANDOM=INTERCEPT | SUBJECT(id) COVTYPE(VC)
  /REPEATED=Tijd_tot_eos | SUBJECT(id) COVTYPE(TP)
  /EMMEANS=TABLES(treatment_arm*Tijd_tot_eos) .

MIXED ln_albumin_excr BY center treatment_arm Tijd_tot_eos
```

```
  /CRITERIA=DFMETHOD(SATTERTHWAITE) CIN(95) MXITER(100) MXSTEP(10)
SCORING(1)
    SINGULAR(0.000000000001) HCONVERGE(0.00000001, RELATIVE)
LCONVERGE(0, ABSOLUTE) PCONVERGE(0,
    ABSOLUTE)
  /FIXED=center treatment_arm Tijd_tot_eos treatment_arm*Tijd_tot_eos | SSTYPE(3)
  /METHOD=REML
  /PRINT=SOLUTION
  /RANDOM=INTERCEPT | SUBJECT(id) COVTYPE(VC)
  /REPEATED=Tijd_tot_eos | SUBJECT(id) COVTYPE(TP)
  /EMMEANS=TABLES(treatment_arm*Tijd_tot_eos) .

MIXED ln_urinekim1excr BY center treatment_arm Tijd_tot_eos
  /CRITERIA=DFMETHOD(SATTERTHWAITE) CIN(95) MXITER(100) MXSTEP(10)
SCORING(1)
    SINGULAR(0.000000000001) HCONVERGE(0.00000001, RELATIVE)
LCONVERGE(0, ABSOLUTE) PCONVERGE(0,
    ABSOLUTE)
  /FIXED=center treatment_arm Tijd_tot_eos treatment_arm*Tijd_tot_eos | SSTYPE(3)
  /METHOD=REML
  /PRINT=SOLUTION
  /RANDOM=INTERCEPT | SUBJECT(id) COVTYPE(VC)
  /REPEATED=Tijd_tot_eos | SUBJECT(id) COVTYPE(UN)
  /EMMEANS=TABLES(treatment_arm*Tijd_tot_eos) .

MIXED ln_urinengalexcr BY center treatment_arm Tijd_tot_eos
  /CRITERIA=DFMETHOD(SATTERTHWAITE) CIN(95) MXITER(100) MXSTEP(10)
SCORING(1)
    SINGULAR(0.000000000001) HCONVERGE(0.00000001, RELATIVE)
LCONVERGE(0, ABSOLUTE) PCONVERGE(0,
    ABSOLUTE)
```

```
  /FIXED=center treatment_arm Tijd_tot_eos treatment_arm*Tijd_tot_eos | SSTYPE(3)
  /METHOD=REML
  /PRINT=SOLUTION
  /RANDOM=INTERCEPT | SUBJECT(id) COVTYPE(VC)
  /REPEATED=Tijd_tot_eos | SUBJECT(id) COVTYPE(TPH)
  /EMMEANS=TABLES(treatment_arm*Tijd_tot_eos) .

MIXED ln_totalfgf23 BY center treatment_arm Tijd_tot_eos
  /CRITERIA=DFMETHOD(SATTERTHWAITE) CIN(95) MXITER(100) MXSTEP(10)
SCORING(1)
    SINGULAR(0.000000000001) HCONVERGE(0.00000001, RELATIVE)
LCONVERGE(0, ABSOLUTE) PCONVERGE(0,
    ABSOLUTE)
  /FIXED=center treatment_arm Tijd_tot_eos treatment_arm*Tijd_tot_eos | SSTYPE(3)
  /METHOD=REML
  /PRINT=SOLUTION
  /RANDOM=INTERCEPT | SUBJECT(id) COVTYPE(VC)
  /REPEATED=Tijd_tot_eos | SUBJECT(id) COVTYPE(UN)
  /EMMEANS=TABLES(treatment_arm*Tijd_tot_eos) .

MIXED ln_ifgf23 BY center treatment_arm Tijd_tot_eos
  /CRITERIA=DFMETHOD(SATTERTHWAITE) CIN(95) MXITER(100) MXSTEP(10)
SCORING(1)
    SINGULAR(0.000000000001) HCONVERGE(0.00000001, RELATIVE)
LCONVERGE(0, ABSOLUTE) PCONVERGE(0,
    ABSOLUTE)
  /FIXED=center treatment_arm Tijd_tot_eos treatment_arm*Tijd_tot_eos | SSTYPE(3)
  /METHOD=REML
  /PRINT=SOLUTION
  /RANDOM=INTERCEPT | SUBJECT(id) COVTYPE(VC)
```

```
  /REPEATED=Tijd_tot_eos | SUBJECT(id) COVTYPE(TPH)
  /EMMEANS=TABLES(treatment_arm*Tijd_tot_eos) .

MIXED fosfaat BY center treatment_arm Tijd_tot_eos
  /CRITERIA=DFMETHOD(SATTERTHWAITE) CIN(95) MXITER(100) MXSTEP(10)
SCORING(1)
    SINGULAR(0.000000000001) HCONVERGE(0.00000001, RELATIVE)
LCONVERGE(0, ABSOLUTE) PCONVERGE(0,
    ABSOLUTE)
  /FIXED=center treatment_arm Tijd_tot_eos treatment_arm*Tijd_tot_eos | SSTYPE(3)
  /METHOD=REML
  /PRINT=SOLUTION
  /RANDOM=INTERCEPT | SUBJECT(id) COVTYPE(VC)
  /REPEATED=Tijd_tot_eos | SUBJECT(id) COVTYPE(UN)
  /EMMEANS=TABLES(treatment_arm*Tijd_tot_eos) .

MIXED urinefosfaatexcr BY center treatment_arm Tijd_tot_eos
  /CRITERIA=DFMETHOD(SATTERTHWAITE) CIN(95) MXITER(100) MXSTEP(10)
SCORING(1)
    SINGULAR(0.000000000001) HCONVERGE(0.00000001, RELATIVE)
LCONVERGE(0, ABSOLUTE) PCONVERGE(0,
    ABSOLUTE)
  /FIXED=center treatment_arm Tijd_tot_eos treatment_arm*Tijd_tot_eos | SSTYPE(3)
  /METHOD=REML
  /PRINT=SOLUTION
  /RANDOM=INTERCEPT | SUBJECT(id) COVTYPE(VC)
  /REPEATED=Tijd_tot_eos | SUBJECT(id) COVTYPE(TPH)
  /EMMEANS=TABLES(treatment_arm*Tijd_tot_eos) .

MIXED calcium BY center treatment_arm Tijd_tot_eos
```

```
  /CRITERIA=DFMETHOD(SATTERTHWAITE) CIN(95) MXITER(100) MXSTEP(10) SCORING(1)
    SINGULAR(0.000000000001) HCONVERGE(0.00000001, RELATIVE) LCONVERGE(0, ABSOLUTE) PCONVERGE(0,
    ABSOLUTE)
  /FIXED=center treatment_arm Tijd_tot_eos treatment_arm*Tijd_tot_eos | SSTYPE(3)
  /METHOD=REML
  /PRINT=SOLUTION
  /RANDOM=INTERCEPT | SUBJECT(id) COVTYPE(VC)
  /REPEATED=Tijd_tot_eos | SUBJECT(id) COVTYPE(TPH)
  /EMMEANS=TABLES(treatment_arm*Tijd_tot_eos) .

MIXED ln_urinecalciumexcr BY center treatment_arm Tijd_tot_eos
  /CRITERIA=DFMETHOD(SATTERTHWAITE) CIN(95) MXITER(100) MXSTEP(10) SCORING(1)
    SINGULAR(0.000000000001) HCONVERGE(0.00000001, RELATIVE) LCONVERGE(0, ABSOLUTE) PCONVERGE(0,
    ABSOLUTE)
  /FIXED=center treatment_arm Tijd_tot_eos treatment_arm*Tijd_tot_eos | SSTYPE(3)
  /METHOD=REML
  /PRINT=SOLUTION
  /RANDOM=INTERCEPT | SUBJECT(id) COVTYPE(VC)
  /REPEATED=Tijd_tot_eos | SUBJECT(id) COVTYPE(TPH)
  /EMMEANS=TABLES(treatment_arm*Tijd_tot_eos) .

MIXED ln_pth BY center treatment_arm Tijd_tot_eos
  /CRITERIA=DFMETHOD(SATTERTHWAITE) CIN(95) MXITER(100) MXSTEP(10) SCORING(1)
    SINGULAR(0.000000000001) HCONVERGE(0.00000001, RELATIVE) LCONVERGE(0, ABSOLUTE) PCONVERGE(0,
    ABSOLUTE)
```

```
  /FIXED=center treatment_arm Tijd_tot_eos treatment_arm*Tijd_tot_eos | SSTYPE(3)
  /METHOD=REML
  /PRINT=SOLUTION
  /RANDOM=INTERCEPT | SUBJECT(id) COVTYPE(VC)
  /REPEATED=Tijd_tot_eos | SUBJECT(id) COVTYPE(TPH)
  /EMMEANS=TABLES(treatment_arm*Tijd_tot_eos) .

MIXED ln_troponine BY center treatment_arm Tijd_tot_eos
  /CRITERIA=DFMETHOD(SATTERTHWAITE) CIN(95) MXITER(100) MXSTEP(10)
SCORING(1)
    SINGULAR(0.000000000001) HCONVERGE(0.00000001, RELATIVE)
LCONVERGE(0, ABSOLUTE) PCONVERGE(0,
    ABSOLUTE)
  /FIXED=center treatment_arm Tijd_tot_eos treatment_arm*Tijd_tot_eos | SSTYPE(3)
  /METHOD=REML
  /PRINT=SOLUTION
  /RANDOM=INTERCEPT | SUBJECT(id) COVTYPE(VC)
  /REPEATED=Tijd_tot_eos | SUBJECT(id) COVTYPE(TP)
  /EMMEANS=TABLES(treatment_arm*Tijd_tot_eos) .

MIXED ln_ntprobnp BY center treatment_arm Tijd_tot_eos
  /CRITERIA=DFMETHOD(SATTERTHWAITE) CIN(95) MXITER(100) MXSTEP(10)
SCORING(1)
    SINGULAR(0.000000000001) HCONVERGE(0.00000001, RELATIVE)
LCONVERGE(0, ABSOLUTE) PCONVERGE(0,
    ABSOLUTE)
  /FIXED=center treatment_arm Tijd_tot_eos treatment_arm*Tijd_tot_eos | SSTYPE(3)
  /METHOD=REML
  /PRINT=SOLUTION
  /RANDOM=INTERCEPT | SUBJECT(id) COVTYPE(VC)
```

```
  /REPEATED=Tijd_tot_eos | SUBJECT(id) COVTYPE(UN)
  /EMMEANS=TABLES(treatment_arm*Tijd_tot_eos) .

MIXED syst_bp BY center treatment_arm Tijd_tot_eos
  /CRITERIA=DFMETHOD(SATTERTHWAITE) CIN(95) MXITER(100) MXSTEP(10)
SCORING(1)
    SINGULAR(0.000000000001) HCONVERGE(0.00000001, RELATIVE)
LCONVERGE(0, ABSOLUTE) PCONVERGE(0,
    ABSOLUTE)
  /FIXED=center treatment_arm Tijd_tot_eos treatment_arm*Tijd_tot_eos | SSTYPE(3)
  /METHOD=REML
  /PRINT=SOLUTION
  /RANDOM=INTERCEPT | SUBJECT(id) COVTYPE(VC)
  /REPEATED=Tijd_tot_eos | SUBJECT(id) COVTYPE(UN)
  /EMMEANS=TABLES(treatment_arm*Tijd_tot_eos) .

MIXED ln_urinenatrium BY center treatment_arm Tijd_tot_eos
  /CRITERIA=DFMETHOD(SATTERTHWAITE) CIN(95) MXITER(100) MXSTEP(10)
SCORING(1)
    SINGULAR(0.000000000001) HCONVERGE(0.00000001, RELATIVE)
LCONVERGE(0, ABSOLUTE) PCONVERGE(0,
    ABSOLUTE)
  /FIXED=center treatment_arm Tijd_tot_eos treatment_arm*Tijd_tot_eos | SSTYPE(3)
  /METHOD=REML
  /PRINT=SOLUTION
  /RANDOM=INTERCEPT | SUBJECT(id) COVTYPE(VC)
  /REPEATED=Tijd_tot_eos | SUBJECT(id) COVTYPE(TP)
  /EMMEANS=TABLES(treatment_arm*Tijd_tot_eos) .

MIXED ln_fena BY center treatment_arm Tijd_tot_eos
```

```
  /CRITERIA=DFMETHOD(SATTERTHWAITE) CIN(95) MXITER(100) MXSTEP(10)
SCORING(1)
    SINGULAR(0.000000000001) HCONVERGE(0.00000001, RELATIVE)
LCONVERGE(0, ABSOLUTE) PCONVERGE(0,
    ABSOLUTE)
  /FIXED=center treatment_arm Tijd_tot_eos treatment_arm*Tijd_tot_eos | SSTYPE(3)
  /METHOD=REML
  /PRINT=SOLUTION
  /RANDOM=INTERCEPT | SUBJECT(id) COVTYPE(VC)
  /REPEATED=Tijd_tot_eos | SUBJECT(id) COVTYPE(TP)
  /EMMEANS=TABLES(treatment_arm*Tijd_tot_eos) .

MIXED asat BY center treatment_arm Tijd_tot_eos
  /CRITERIA=DFMETHOD(SATTERTHWAITE) CIN(95) MXITER(100) MXSTEP(10)
SCORING(1)
    SINGULAR(0.000000000001) HCONVERGE(0.00000001, RELATIVE)
LCONVERGE(0, ABSOLUTE) PCONVERGE(0,
    ABSOLUTE)
  /FIXED=center treatment_arm Tijd_tot_eos treatment_arm*Tijd_tot_eos | SSTYPE(3)
  /METHOD=REML
  /PRINT=SOLUTION
  /RANDOM=INTERCEPT | SUBJECT(id) COVTYPE(VC)
  /REPEATED=Tijd_tot_eos | SUBJECT(id) COVTYPE(UN)
  /EMMEANS=TABLES(treatment_arm*Tijd_tot_eos) .

MIXED alat BY center treatment_arm Tijd_tot_eos
  /CRITERIA=DFMETHOD(SATTERTHWAITE) CIN(95) MXITER(100) MXSTEP(10)
SCORING(1)
    SINGULAR(0.000000000001) HCONVERGE(0.00000001, RELATIVE)
LCONVERGE(0, ABSOLUTE) PCONVERGE(0,
    ABSOLUTE)
```

```
 /FIXED=center treatment_arm Tijd_tot_eos treatment_arm*Tijd_tot_eos | SSTYPE(3)
 /METHOD=REML
 /PRINT=SOLUTION
 /RANDOM=INTERCEPT | SUBJECT(id) COVTYPE(VC)
 /REPEATED=Tijd_tot_eos | SUBJECT(id) COVTYPE(UN)
 /EMMEANS=TABLES(treatment_arm*Tijd_tot_eos) .
```

Conversion in another database

```
COMPUTE Estimate_convertedln=(Estimate_ln) * ( - 1).
EXECUTE.

COMPUTE LB_Estimate_convertedln=(LB_estimate_ln) * ( - 1).
EXECUTE.

COMPUTE UB_Estimate_convertedln=(UB_estimate_ln) * ( - 1).
EXECUTE.

COMPUTE Estimate=EXP(Estimate_convertedln).
EXECUTE.

COMPUTE LB_estimate=EXP(LB_Estimate_convertedln).
EXECUTE.

COMPUTE UB_estimate=EXP(UB_Estimate_convertedln).
EXECUTE.
```

** Syntax for ANCOVA**

Use full analysis imputation dataset

```
SORT CASES  BY Imputation_.
SPLIT FILE BY Imputation_.



COMPUTE Delta_6mwt_distance=sixmwt_distance.v24 - sixmwt_distance.v0.
EXECUTE.

COMPUTE Delta_6mwt_percpred=SixMWT_percpred_eos - SixMWT_percpred_baseline.
EXECUTE.

COMPUTE Delta_ftstst_mean=ftstst_mean_eos - ftstst_mean_baseline.
EXECUTE.

COMPUTE Delta_tug_mean=tug_mean_eos - tug_mean_baseline.
EXECUTE.

COMPUTE Delta_hgs_mean=hgs_mean_eos - hgs_mean_baseline.
 EXECUTE.

COMPUTE Delta_qol_cis_sum=qol_cis_sum.v24 - qol_cis_sum.v0.
 EXECUTE.

COMPUTE Delta_EQindex=EQindex.v24 - EQindex.v0.
   EXECUTE.
```

```
COMPUTE Delta_EQvas=qol_eq5d5l_6.v24 - qol_eq5d5l_6.v0.
  EXECUTE.

COMPUTE
Delta_qol_dmfs_impactvanvermoeidheid=qol_dmfs_impactvanvermoeidheid.v24 -
qol_dmfs_impactvanvermoeidheid.v0.
  EXECUTE.

COMPUTE
Delta_qol_dmfs_directegevolgenvanvermoeidheid=qol_dmfs_directegevolgenvanvermoeidh
eid.v24 - qol_dmfs_directegevolgenvanvermoeidheid.v0.
EXECUTE.

COMPUTE Delta_qol_dmfs_mentalevermoeidheid=qol_dmfs_mentalevermoeidheid.v24 -
qol_dmfs_mentalevermoeidheid.v0.
EXECUTE.

COMPUTE Delta_qol_dmfs_fysiekevermoeidheid=qol_dmfs_fysiekevermoeidheid.v24 -
qol_dmfs_fysiekevermoeidheid.v0.
EXECUTE.

COMPUTE
Delta_qol_dmfs_omgaanmetvermoeidheid=qol_dmfs_omgaanmetvermoeidheid.v24 -
qol_dmfs_omgaanmetvermoeidheid.v0.
EXECUTE.

COMPUTE
Delta_qol_rand36_fysiekfunctioneren_schaal=qol_rand36_fysiekfunctioneren_schaal.v24 -
qol_rand36_fysiekfunctioneren_schaal.v0.
EXECUTE.
```

COMPUTE
Delta_qol_rand36_sociaalfunctioneren_schaal=qol_rand36_sociaalfunctioneren_schaal.v24 - qol_rand36_sociaalfunctioneren_schaal.v0.

EXECUTE.

COMPUTE
Delta_qol_rand36_mentalegezondheid_schaal=qol_rand36_mentalegezondheid_schaal.v24 - qol_rand36_mentalegezondheid_schaal.v0.

EXECUTE.

COMPUTE Delta_qol_rand36_vitaliteit_schaal=qol_rand36_vitaliteit_schaal.v24 - qol_rand36_vitaliteit_schaal.v0.

EXECUTE.

COMPUTE Delta_qol_rand36_pijn_schaal=qol_rand36_pijn_schaal.v24 - qol_rand36_pijn_schaal.v0.

EXECUTE.

COMPUTE Delta_qol_rand36_algemeen_schaal=qol_rand36_algemeen_schaal.v24 - qol_rand36_algemeen_schaal.v0.

EXECUTE.

COMPUTE Delta_qol_rand36_verandering_schaal=qol_rand36_verandering_schaal.v24 - qol_rand36_verandering_schaal.v0.

EXECUTE.

COMPUTE Delta_echo_ivsd=echo_ivsd.v24 - echo_ivsd.v0.

EXECUTE.

COMPUTE Delta_echo_lvidd=echo_lvidd.v24 - echo_lvidd.v0.

EXECUTE.

```
COMPUTE Delta_echo_lvpwd=echo_lvpwd.v24 - echo_lvpwd.v0.
EXECUTE.

COMPUTE Delta_echo_lavi=echo_lavi_v24 - echo_lavi_baseline.
EXECUTE.

COMPUTE Delta_echo_rwt=echo_rwt_v24 - echo_rwt_baseline.
EXECUTE.

COMPUTE Delta_echo_lvmi_devereux=echo_lvmi_devereux_v24 -
echo_lvmi_devereux_baseline.
EXECUTE.

COMPUTE Delta_echo_lvef=echo_lvef_v24 - echo_lvef_baseline.
EXECUTE.

COMPUTE Delta_echo_e_lateral_septal=echo_e_lateral_septal.v24 -
echo_e_lateral_septal.v0.
EXECUTE.

COMPUTE Delta_echo_tapse=echo_tapse.v24 - echo_tapse.v0.
EXECUTE.

COMPUTE Delta_echo_srv=echo_srv.v24 - echo_srv.v0.
EXECUTE.

COMPUTE Delta_echo_strv_annulus=echo_strv_annulus.v24 - echo_strv_annulus.v0.
EXECUTE.

COMPUTE Delta_echo_ratio_ea=echo_ratio_ea.v24 - echo_ratio_ea.v0.
```

```
EXECUTE.

COMPUTE Delta_echo_ratio_ee=echo_ratio_ee.v24 - echo_ratio_ee.v0.
EXECUTE.

COMPUTE Delta_echo_gls_positive=echo_gls_positive.v24 - echo_gls_positive.v0.
EXECUTE.

COMPUTE Delta_echo_lasr=echo_lasr.v24 - echo_lasr.v0.
EXECUTE.

COMPUTE Delta_echo_lasct=echo_lasct.v24 - echo_lasct.v0.
EXECUTE.

COMPUTE Delta_echo_lascd=echo_lascd.v24 - echo_lascd.v0.
EXECUTE.

COMPUTE Delta_echo_hfapeff_score=echo_hfapeff_score.v24 - echo_hfapeff_score.v0.
EXECUTE.

IF  (((np_cst_cognitive_decline = 0) OR MISSING(np_cst_cognitive_decline)) &
((np_nlv_iqscore >=
   80) OR (MISSING(np_nlv_iqscore))))
Delta_np_domein_geheugen=np_domein_geheugen_eos - np_domein_geheugen_baseline.
  EXECUTE.

IF  (((np_cst_cognitive_decline = 0) OR MISSING(np_cst_cognitive_decline)) &
((np_nlv_iqscore >=
```

```
   80) OR (MISSING(np_nlv_iqscore))))
Delta_np_domein_aandacht=np_domein_aandacht_eos - np_domein_aandacht_baseline.
  EXECUTE.


IF  (((np_cst_cognitive_decline = 0) OR MISSING(np_cst_cognitive_decline)) &
((np_nlv_iqscore >=
   80) OR (MISSING(np_nlv_iqscore))))
Delta_np_domein_executieffunctioneren=np_domein_executieffunctioneren_eos -
np_domein_executieffunctioneren_baseline.
EXECUTE.


IF  (((np_cst_cognitive_decline = 0) OR MISSING(np_cst_cognitive_decline)) &
((np_nlv_iqscore >=
   80) OR (MISSING(np_nlv_iqscore))))
Delta_np_dst_forward_pscore=np_dst_forward_pscore_eos - np_dst_forward_pscore.
EXECUTE.


IF  (((np_cst_cognitive_decline = 0) OR MISSING(np_cst_cognitive_decline)) &
((np_nlv_iqscore >=
   80) OR (MISSING(np_nlv_iqscore))))
Delta_np_wf_mean_pscore=np_wf_mean_pscore_eos - np_wf_mean_pscore.
EXECUTE.


IF  (((np_cst_cognitive_decline = 0) OR MISSING(np_cst_cognitive_decline)) &
((np_nlv_iqscore >=
   80) OR (MISSING(np_nlv_iqscore))))
Delta_np_15wt_goed_pscore=np_15wt_goed_pscore_eos - np_15wt_goed_pscore.
  EXECUTE.


IF  (((np_cst_cognitive_decline = 0) OR MISSING(np_cst_cognitive_decline)) &
((np_nlv_iqscore >=
```

80) OR (MISSING(np_nlv_iqscore))))
Delta_np_15wt_recall_pscore_age_sex_edu=np_15wt_recall_pscore_age_sex_edu_eos - np_15wt_recall_pscore_age_sex_edu.

EXECUTE.

IF (((np_cst_cognitive_decline = 0) OR MISSING(np_cst_cognitive_decline)) & ((np_nlv_iqscore >=

80) OR (MISSING(np_nlv_iqscore))))
Delta_np_sdmt_mean_tscore=np_sdmt_mean_tscore_eos - np_sdmt_mean_tscore.

EXECUTE.

IF (((np_cst_cognitive_decline = 0) OR MISSING(np_cst_cognitive_decline)) & ((np_nlv_iqscore >=

80) OR (MISSING(np_nlv_iqscore)))) Delta_np_tmt_a_pscore=np_tmt_a_pscore_eos - np_tmt_a_pscore.

EXECUTE.

IF (((np_cst_cognitive_decline = 0) OR MISSING(np_cst_cognitive_decline)) & ((np_nlv_iqscore >=

80) OR (MISSING(np_nlv_iqscore)))) Delta_np_tmt_b_pscore=np_tmt_b_pscore_eos - np_tmt_b_pscore.

EXECUTE.

IF (((np_cst_cognitive_decline = 0) OR MISSING(np_cst_cognitive_decline)) & ((np_nlv_iqscore >=

80) OR (MISSING(np_nlv_iqscore)))) Delta_np_tmt_b_a_pscore=np_tmt_b_a_pscore_eos - np_tmt_b_a_pscore.

EXECUTE.

IF (((np_cst_cognitive_decline = 0) OR MISSING(np_cst_cognitive_decline)) & ((np_nlv_iqscore >=

80) OR (MISSING(np_nlv_iqscore))))
Delta_np_wf_letter_pscore=np_wf_letter_pscore_eos - np_wf_letter_pscore.

```
EXECUTE.


IF  (((np_cst_cognitive_decline = 0) OR MISSING(np_cst_cognitive_decline)) &
((np_nlv_iqscore >=
    80) OR (MISSING(np_nlv_iqscore))))
Delta_np_dst_backward_pscore=np_dst_backward_pscore_eos - np_dst_backward_pscore.
EXECUTE.


COMPUTE Delta_GSRS_total=GSRS_total_V24 - GSRS_total_V0.
EXECUTE.


UNIANOVA Delta_6mwt_distance BY treatment_arm WITH center sixmwt_distance.v0
  /METHOD=SSTYPE(3)
  /INTERCEPT=INCLUDE
  /EMMEANS=TABLES(treatment_arm) WITH(sixmwt_distance.v0=MEAN
center=MEAN) COMPARE ADJ(BONFERRONI)
  /CRITERIA=ALPHA(.05)
  /DESIGN=center sixmwt_distance.v0 treatment_arm.


UNIANOVA Delta_6mwt_percpred BY treatment_arm WITH center
SixMWT_percpred_baseline
  /METHOD=SSTYPE(3)
  /INTERCEPT=INCLUDE
  /EMMEANS=TABLES(treatment_arm) WITH(SixMWT_percpred_baseline =MEAN
center=MEAN) COMPARE ADJ(BONFERRONI)
  /CRITERIA=ALPHA(.05)
  /DESIGN=center SixMWT_percpred_baseline treatment_arm.
```

```
UNIANOVA Delta_hgs_mean BY treatment_arm WITH center hgs_mean_baseline
  /METHOD=SSTYPE(3)
  /INTERCEPT=INCLUDE
  /EMMEANS=TABLES(treatment_arm) WITH(hgs_mean_baseline  =MEAN
center=MEAN) COMPARE ADJ(BONFERRONI)
  /CRITERIA=ALPHA(.05)
  /DESIGN=center hgs_mean_baseline treatment_arm.

UNIANOVA Delta_ftstst_mean BY treatment_arm WITH center ftstst_mean_baseline
  /METHOD=SSTYPE(3)
  /INTERCEPT=INCLUDE
  /EMMEANS=TABLES(treatment_arm) WITH(ftstst_mean_baseline =MEAN
center=MEAN) COMPARE ADJ(BONFERRONI)
  /CRITERIA=ALPHA(.05)
  /DESIGN=center ftstst_mean_baseline treatment_arm.

UNIANOVA Delta_tug_mean BY treatment_arm WITH center tug_mean_baseline
  /METHOD=SSTYPE(3)
  /INTERCEPT=INCLUDE
  /EMMEANS=TABLES(treatment_arm) WITH(tug_mean_baseline =MEAN
center=MEAN) COMPARE ADJ(BONFERRONI)
  /CRITERIA=ALPHA(.05)
  /DESIGN=center tug_mean_baseline treatment_arm.


UNIANOVA Delta_qol_cis_sum BY treatment_arm WITH center qol_cis_sum.v0
  /METHOD=SSTYPE(3)
  /INTERCEPT=INCLUDE
```

```
  /EMMEANS=TABLES(treatment_arm) WITH(qol_cis_sum.v0 =MEAN center=MEAN) COMPARE ADJ(BONFERRONI)
  /CRITERIA=ALPHA(.05)
  /DESIGN=center qol_cis_sum.v0  treatment_arm.
```

```
UNIANOVA Delta_EQindex BY treatment_arm WITH center EQindex.v0
  /METHOD=SSTYPE(3)
  /INTERCEPT=INCLUDE
  /EMMEANS=TABLES(treatment_arm) WITH(EQindex.v0 =MEAN center=MEAN) COMPARE ADJ(BONFERRONI)
  /CRITERIA=ALPHA(.05)
  /DESIGN=center EQindex.v0  treatment_arm.
```

```
UNIANOVA Delta_EQvas BY treatment_arm WITH center qol_eq5d5l_6.v0
  /METHOD=SSTYPE(3)
  /INTERCEPT=INCLUDE
  /EMMEANS=TABLES(treatment_arm) WITH(qol_eq5d5l_6.v0 =MEAN center=MEAN) COMPARE ADJ(BONFERRONI)
  /CRITERIA=ALPHA(.05)
  /DESIGN=center qol_eq5d5l_6.v0  treatment_arm.
```

```
UNIANOVA Delta_qol_dmfs_impactvanvermoeidheid BY treatment_arm WITH center qol_dmfs_impactvanvermoeidheid.v0
  /METHOD=SSTYPE(3)
```

```
  /INTERCEPT=INCLUDE
  /EMMEANS=TABLES(treatment_arm) WITH(qol_dmfs_impactvanvermoeidheid.v0
=MEAN center=MEAN) COMPARE ADJ(BONFERRONI)
  /CRITERIA=ALPHA(.05)
  /DESIGN=center qol_dmfs_impactvanvermoeidheid.v0  treatment_arm.


UNIANOVA Delta_qol_dmfs_directegevolgenvanvermoeidheid BY treatment_arm WITH
center qol_dmfs_directegevolgenvanvermoeidheid.v0
  /METHOD=SSTYPE(3)
  /INTERCEPT=INCLUDE
  /EMMEANS=TABLES(treatment_arm)
WITH(qol_dmfs_directegevolgenvanvermoeidheid.v0 =MEAN center=MEAN) COMPARE
ADJ(BONFERRONI)
  /CRITERIA=ALPHA(.05)
  /DESIGN=center qol_dmfs_directegevolgenvanvermoeidheid.v0  treatment_arm.


UNIANOVA Delta_qol_dmfs_mentalevermoeidheid BY treatment_arm WITH center
qol_dmfs_mentalevermoeidheid.v0
  /METHOD=SSTYPE(3)
  /INTERCEPT=INCLUDE
  /EMMEANS=TABLES(treatment_arm) WITH(qol_dmfs_mentalevermoeidheid.v0=MEAN
center=MEAN) COMPARE ADJ(BONFERRONI)
  /CRITERIA=ALPHA(.05)
  /DESIGN=center qol_dmfs_mentalevermoeidheid.v0  treatment_arm.


UNIANOVA Delta_qol_dmfs_fysiekevermoeidheid BY treatment_arm WITH center
qol_dmfs_fysiekevermoeidheid.v0
  /METHOD=SSTYPE(3)
  /INTERCEPT=INCLUDE
  /EMMEANS=TABLES(treatment_arm) WITH(qol_dmfs_fysiekevermoeidheid.v0 =MEAN
center=MEAN) COMPARE ADJ(BONFERRONI)
  /CRITERIA=ALPHA(.05)
```

```
  /DESIGN=center qol_dmfs_fysiekevermoeidheid.v0  treatment_arm.


UNIANOVA Delta_qol_dmfs_omgaanmetvermoeidheid BY treatment_arm WITH center
qol_dmfs_omgaanmetvermoeidheid.v0

  /METHOD=SSTYPE(3)

  /INTERCEPT=INCLUDE

  /EMMEANS=TABLES(treatment_arm) WITH(qol_dmfs_omgaanmetvermoeidheid.v0
=MEAN center=MEAN) COMPARE ADJ(BONFERRONI)

  /CRITERIA=ALPHA(.05)

  /DESIGN=center qol_dmfs_omgaanmetvermoeidheid.v0  treatment_arm.



UNIANOVA Delta_qol_rand36_fysiekfunctioneren_schaal BY treatment_arm WITH center
qol_rand36_fysiekfunctioneren_schaal.v0

  /METHOD=SSTYPE(3)

  /INTERCEPT=INCLUDE

  /EMMEANS=TABLES(treatment_arm)
WITH(qol_rand36_fysiekfunctioneren_schaal.v0=MEAN center=MEAN) COMPARE
ADJ(BONFERRONI)

  /CRITERIA=ALPHA(.05)

  /DESIGN=center qol_rand36_fysiekfunctioneren_schaal.v0  treatment_arm.


UNIANOVA Delta_qol_rand36_sociaalfunctioneren_schaal BY treatment_arm WITH center
qol_rand36_sociaalfunctioneren_schaal.v0

  /METHOD=SSTYPE(3)

  /INTERCEPT=INCLUDE

  /EMMEANS=TABLES(treatment_arm) WITH(qol_rand36_sociaalfunctioneren_schaal.v0
=MEAN center=MEAN) COMPARE ADJ(BONFERRONI)

  /CRITERIA=ALPHA(.05)

  /DESIGN=center qol_rand36_sociaalfunctioneren_schaal.v0  treatment_arm.
```

```
UNIANOVA Delta_qol_rand36_mentalegezondheid_schaal BY treatment_arm WITH center qol_rand36_mentalegezondheid_schaal.v0
  /METHOD=SSTYPE(3)
  /INTERCEPT=INCLUDE
  /EMMEANS=TABLES(treatment_arm) WITH(qol_rand36_mentalegezondheid_schaal.v0 =MEAN center=MEAN) COMPARE ADJ(BONFERRONI)
  /CRITERIA=ALPHA(.05)
  /DESIGN=center qol_rand36_mentalegezondheid_schaal.v0  treatment_arm.

UNIANOVA Delta_qol_rand36_vitaliteit_schaal BY treatment_arm WITH center qol_rand36_vitaliteit_schaal.v0
  /METHOD=SSTYPE(3)
  /INTERCEPT=INCLUDE
  /EMMEANS=TABLES(treatment_arm) WITH(qol_rand36_vitaliteit_schaal.v0 =MEAN center=MEAN) COMPARE ADJ(BONFERRONI)
  /CRITERIA=ALPHA(.05)
  /DESIGN=center qol_rand36_vitaliteit_schaal.v0  treatment_arm.

UNIANOVA Delta_qol_rand36_pijn_schaal BY treatment_arm WITH center qol_rand36_pijn_schaal.v0
  /METHOD=SSTYPE(3)
  /INTERCEPT=INCLUDE
  /EMMEANS=TABLES(treatment_arm) WITH(qol_rand36_pijn_schaal.v0 =MEAN center=MEAN) COMPARE ADJ(BONFERRONI)
  /CRITERIA=ALPHA(.05)
  /DESIGN=center qol_rand36_pijn_schaal.v0  treatment_arm.

UNIANOVA Delta_qol_rand36_algemeen_schaal BY treatment_arm WITH center qol_rand36_algemeen_schaal.v0
  /METHOD=SSTYPE(3)
  /INTERCEPT=INCLUDE
```

```
  /EMMEANS=TABLES(treatment_arm) WITH(qol_rand36_algemeen_schaal.v0 =MEAN
center=MEAN) COMPARE ADJ(BONFERRONI)
  /CRITERIA=ALPHA(.05)
  /DESIGN=center qol_rand36_algemeen_schaal.v0  treatment_arm.

UNIANOVA Delta_qol_rand36_verandering_schaal BY treatment_arm WITH center
qol_rand36_verandering_schaal.v0
  /METHOD=SSTYPE(3)
  /INTERCEPT=INCLUDE
  /EMMEANS=TABLES(treatment_arm) WITH(qol_rand36_verandering_schaal.v0
=MEAN center=MEAN) COMPARE ADJ(BONFERRONI)
  /CRITERIA=ALPHA(.05)
  /DESIGN=center qol_rand36_verandering_schaal.v0  treatment_arm.

UNIANOVA Delta_echo_ivsd BY treatment_arm WITH echo_ivsd.v0
  /METHOD=SSTYPE(3)
  /INTERCEPT=INCLUDE
  /EMMEANS=TABLES(treatment_arm) WITH(echo_ivsd.v0 =MEAN) COMPARE
ADJ(BONFERRONI)
  /CRITERIA=ALPHA(.05)
  /DESIGN=treatment_arm echo_ivsd.v0.

UNIANOVA Delta_echo_lvidd BY treatment_arm WITH echo_lvidd.v0
  /METHOD=SSTYPE(3)
  /INTERCEPT=INCLUDE
  /EMMEANS=TABLES(treatment_arm) WITH(echo_lvidd.v0 =MEAN) COMPARE
ADJ(BONFERRONI)
  /CRITERIA=ALPHA(.05)
  /DESIGN=treatment_arm echo_lvidd.v0.
```

```
UNIANOVA Delta_echo_lvpwd BY treatment_arm WITH echo_lvpwd.v0
  /METHOD=SSTYPE(3)
  /INTERCEPT=INCLUDE
  /EMMEANS=TABLES(treatment_arm) WITH(echo_lvpwd.v0 =MEAN) COMPARE ADJ(BONFERRONI)
  /CRITERIA=ALPHA(.05)
  /DESIGN=treatment_arm echo_lvpwd.v0.

UNIANOVA Delta_echo_lavi BY treatment_arm WITH echo_lavi_baseline
  /METHOD=SSTYPE(3)
  /INTERCEPT=INCLUDE
  /EMMEANS=TABLES(treatment_arm) WITH(echo_lavi_baseline=MEAN) COMPARE ADJ(BONFERRONI)
  /CRITERIA=ALPHA(.05)
  /DESIGN=treatment_arm echo_lavi_baseline.

UNIANOVA Delta_echo_rwt BY treatment_arm WITH echo_rwt_baseline
  /METHOD=SSTYPE(3)
  /INTERCEPT=INCLUDE
  /EMMEANS=TABLES(treatment_arm) WITH(echo_rwt_baseline =MEAN) COMPARE ADJ(BONFERRONI)
  /CRITERIA=ALPHA(.05)
  /DESIGN=treatment_arm echo_rwt_baseline.

UNIANOVA Delta_echo_lvmi_devereux BY treatment_arm WITH echo_lvmi_devereux_baseline
  /METHOD=SSTYPE(3)
  /INTERCEPT=INCLUDE
  /EMMEANS=TABLES(treatment_arm) WITH(echo_lvmi_devereux_baseline =MEAN) COMPARE ADJ(BONFERRONI)
```

```
  /CRITERIA=ALPHA(.05)
  /DESIGN=treatment_arm echo_lvmi_devereux_baseline.

UNIANOVA Delta_echo_lvef BY treatment_arm WITH echo_lvef_baseline
  /METHOD=SSTYPE(3)
  /INTERCEPT=INCLUDE
  /EMMEANS=TABLES(treatment_arm) WITH(echo_lvef_baseline=MEAN) COMPARE
ADJ(BONFERRONI)
  /CRITERIA=ALPHA(.05)
  /DESIGN=treatment_arm echo_lvef_baseline.



UNIANOVA Delta_echo_e_lateral_septal BY treatment_arm WITH
echo_e_lateral_septal.v0
  /METHOD=SSTYPE(3)
  /INTERCEPT=INCLUDE
  /EMMEANS=TABLES(treatment_arm) WITH(echo_e_lateral_septal.v0=MEAN)
COMPARE ADJ(BONFERRONI)
  /CRITERIA=ALPHA(.05)
  /DESIGN=treatment_arm echo_e_lateral_septal.v0.

UNIANOVA Delta_echo_tapse BY treatment_arm WITH echo_tapse.v0
  /METHOD=SSTYPE(3)
  /INTERCEPT=INCLUDE
  /EMMEANS=TABLES(treatment_arm) WITH(echo_tapse.v0=MEAN) COMPARE
ADJ(BONFERRONI)
  /CRITERIA=ALPHA(.05)
  /DESIGN=treatment_arm echo_tapse.v0.

UNIANOVA Delta_echo_srv BY treatment_arm WITH echo_srv.v0
```

```
  /METHOD=SSTYPE(3)
  /INTERCEPT=INCLUDE
  /EMMEANS=TABLES(treatment_arm) WITH(echo_srv.v0=MEAN) COMPARE ADJ(BONFERRONI)
  /CRITERIA=ALPHA(.05)
  /DESIGN=treatment_arm echo_srv.v0.

UNIANOVA Delta_echo_strv_annulus BY treatment_arm WITH echo_strv_annulus.v0
  /METHOD=SSTYPE(3)
  /INTERCEPT=INCLUDE
  /EMMEANS=TABLES(treatment_arm) WITH(echo_strv_annulus.v0=MEAN) COMPARE ADJ(BONFERRONI)
  /CRITERIA=ALPHA(.05)
  /DESIGN=treatment_arm echo_strv_annulus.v0.

UNIANOVA Delta_echo_ratio_ea BY treatment_arm WITH echo_ratio_ea.v0
  /METHOD=SSTYPE(3)
  /INTERCEPT=INCLUDE
  /EMMEANS=TABLES(treatment_arm) WITH(echo_ratio_ea.v0=MEAN) COMPARE ADJ(BONFERRONI)
  /CRITERIA=ALPHA(.05)
  /DESIGN=treatment_arm echo_ratio_ea.v0.

UNIANOVA Delta_echo_ratio_ee BY treatment_arm WITH echo_ratio_ee.v0
  /METHOD=SSTYPE(3)
  /INTERCEPT=INCLUDE
  /EMMEANS=TABLES(treatment_arm) WITH(echo_ratio_ee.v0=MEAN) COMPARE ADJ(BONFERRONI)
  /CRITERIA=ALPHA(.05)
  /DESIGN=treatment_arm echo_ratio_ee.v0.
```

```
UNIANOVA Delta_echo_gls_positive BY treatment_arm WITH echo_gls_positive.v0
  /METHOD=SSTYPE(3)
  /INTERCEPT=INCLUDE
  /EMMEANS=TABLES(treatment_arm) WITH(echo_gls_positive.v0=MEAN) COMPARE
ADJ(BONFERRONI)
  /CRITERIA=ALPHA(.05)
  /DESIGN=treatment_arm echo_gls_positive.v0.

UNIANOVA Delta_echo_lasr BY treatment_arm WITH echo_lasr.v0
  /METHOD=SSTYPE(3)
  /INTERCEPT=INCLUDE
  /EMMEANS=TABLES(treatment_arm) WITH(echo_lasr.v0=MEAN) COMPARE
ADJ(BONFERRONI)
  /CRITERIA=ALPHA(.05)
  /DESIGN=treatment_arm echo_lasr.v0.

UNIANOVA Delta_echo_lasct BY treatment_arm WITH echo_lasct.v0
  /METHOD=SSTYPE(3)
  /INTERCEPT=INCLUDE
  /EMMEANS=TABLES(treatment_arm) WITH(echo_lasct.v0=MEAN) COMPARE
ADJ(BONFERRONI)
  /CRITERIA=ALPHA(.05)
  /DESIGN=treatment_arm echo_lasct.v0.

UNIANOVA Delta_echo_lascd BY treatment_arm WITH echo_lascd.v0
  /METHOD=SSTYPE(3)
  /INTERCEPT=INCLUDE
  /EMMEANS=TABLES(treatment_arm) WITH(echo_lascd.v0=MEAN) COMPARE
ADJ(BONFERRONI)
```

```
  /CRITERIA=ALPHA(.05)
  /DESIGN=treatment_arm echo_lascd.v0.

UNIANOVA Delta_echo_hfapeff_score BY treatment_arm WITH echo_hfapeff_score.v0
  /METHOD=SSTYPE(3)
  /INTERCEPT=INCLUDE
  /EMMEANS=TABLES(treatment_arm) WITH(echo_hfapeff_score.v0=MEAN)
COMPARE ADJ(BONFERRONI)
  /CRITERIA=ALPHA(.05)
  /DESIGN=treatment_arm echo_hfapeff_score.v0.

UNIANOVA Delta_np_domein_geheugen BY treatment_arm WITH
np_domein_geheugen_baseline
  /METHOD=SSTYPE(3)
  /INTERCEPT=INCLUDE
  /EMMEANS=TABLES(treatment_arm) WITH(np_domein_geheugen_baseline=MEAN)
COMPARE ADJ(BONFERRONI)
  /CRITERIA=ALPHA(.05)
  /DESIGN=treatment_arm np_domein_geheugen_baseline.

UNIANOVA Delta_np_domein_aandacht BY treatment_arm WITH
np_domein_aandacht_baseline
  /METHOD=SSTYPE(3)
  /INTERCEPT=INCLUDE
  /EMMEANS=TABLES(treatment_arm) WITH(np_domein_aandacht_baseline=MEAN)
COMPARE ADJ(BONFERRONI)
  /CRITERIA=ALPHA(.05)
  /DESIGN=treatment_arm np_domein_aandacht_baseline.
```

```
UNIANOVA Delta_np_domein_executieffunctioneren BY treatment_arm WITH
np_domein_executieffunctioneren_baseline

  /METHOD=SSTYPE(3)

  /INTERCEPT=INCLUDE

  /EMMEANS=TABLES(treatment_arm)
WITH(np_domein_executieffunctioneren_baseline=MEAN) COMPARE
ADJ(BONFERRONI)

  /CRITERIA=ALPHA(.05)

  /DESIGN=treatment_arm np_domein_executieffunctioneren_baseline.


UNIANOVA Delta_np_dst_forward_pscore BY treatment_arm WITH
np_dst_forward_pscore

  /METHOD=SSTYPE(3)

  /INTERCEPT=INCLUDE

  /EMMEANS=TABLES(treatment_arm) WITH(np_dst_forward_pscore=MEAN)
COMPARE ADJ(BONFERRONI)

  /CRITERIA=ALPHA(.05)

  /DESIGN=treatment_arm np_dst_forward_pscore.


UNIANOVA Delta_np_wf_mean_pscore BY treatment_arm WITH np_wf_mean_pscore

  /METHOD=SSTYPE(3)

  /INTERCEPT=INCLUDE

  /EMMEANS=TABLES(treatment_arm) WITH(np_wf_mean_pscore=MEAN) COMPARE
ADJ(BONFERRONI)

  /CRITERIA=ALPHA(.05)

  /DESIGN=treatment_arm np_wf_mean_pscore.


UNIANOVA Delta_np_15wt_goed_pscore BY treatment_arm WITH np_15wt_goed_pscore

  /METHOD=SSTYPE(3)

  /INTERCEPT=INCLUDE
```

```
  /EMMEANS=TABLES(treatment_arm) WITH(np_15wt_goed_pscore=MEAN)
COMPARE ADJ(BONFERRONI)
  /CRITERIA=ALPHA(.05)
  /DESIGN=treatment_arm np_15wt_goed_pscore.

UNIANOVA Delta_np_15wt_recall_pscore_age_sex_edu BY treatment_arm WITH
np_15wt_recall_pscore_age_sex_edu
  /METHOD=SSTYPE(3)
  /INTERCEPT=INCLUDE
  /EMMEANS=TABLES(treatment_arm)
WITH(np_15wt_recall_pscore_age_sex_edu=MEAN) COMPARE ADJ(BONFERRONI)
  /CRITERIA=ALPHA(.05)
  /DESIGN=treatment_arm np_15wt_recall_pscore_age_sex_edu.

UNIANOVA Delta_np_sdmt_mean_tscore BY treatment_arm WITH np_sdmt_mean_tscore
  /METHOD=SSTYPE(3)
  /INTERCEPT=INCLUDE
   /EMMEANS=TABLES(treatment_arm) WITH(np_sdmt_mean_tscore=MEAN)
COMPARE ADJ(BONFERRONI)
  /CRITERIA=ALPHA(.05)
  /DESIGN=treatment_arm np_sdmt_mean_tscore.

UNIANOVA Delta_np_tmt_a_pscore BY treatment_arm WITH np_tmt_a_pscore
  /METHOD=SSTYPE(3)
  /INTERCEPT=INCLUDE
  /EMMEANS=TABLES(treatment_arm) WITH(np_tmt_a_pscore=MEAN) COMPARE
ADJ(BONFERRONI)
  /CRITERIA=ALPHA(.05)
  /DESIGN=treatment_arm np_tmt_a_pscore.

UNIANOVA Delta_np_tmt_b_pscore BY treatment_arm WITH np_tmt_b_pscore
```

```
  /METHOD=SSTYPE(3)
  /INTERCEPT=INCLUDE
  /EMMEANS=TABLES(treatment_arm) WITH(np_tmt_b_pscore=MEAN) COMPARE
ADJ(BONFERRONI)
  /CRITERIA=ALPHA(.05)
  /DESIGN=treatment_arm np_tmt_b_pscore.

UNIANOVA Delta_np_tmt_b_a_pscore BY treatment_arm WITH np_tmt_b_a_pscore
  /METHOD=SSTYPE(3)
  /INTERCEPT=INCLUDE
  /EMMEANS=TABLES(treatment_arm) WITH(np_tmt_b_a_pscore=MEAN) COMPARE
ADJ(BONFERRONI)
  /CRITERIA=ALPHA(.05)
  /DESIGN=treatment_arm np_tmt_b_a_pscore.

UNIANOVA Delta_np_wf_letter_pscore BY treatment_arm WITH np_wf_letter_pscore
  /METHOD=SSTYPE(3)
  /INTERCEPT=INCLUDE
  /EMMEANS=TABLES(treatment_arm) WITH(np_wf_letter_pscore=MEAN) COMPARE
ADJ(BONFERRONI)
  /CRITERIA=ALPHA(.05)
  /DESIGN=treatment_arm np_wf_letter_pscore.

UNIANOVA Delta_np_dst_backward_pscore BY treatment_arm WITH
np_dst_backward_pscore
  /METHOD=SSTYPE(3)
  /INTERCEPT=INCLUDE
  /EMMEANS=TABLES(treatment_arm) WITH(np_dst_backward_pscore=MEAN)
COMPARE ADJ(BONFERRONI)
  /CRITERIA=ALPHA(.05)
  /DESIGN=treatment_arm np_dst_backward_pscore.
```

```
UNIANOVA Delta_GSRS_total BY treatment_arm WITH center GSRS_total_V0
  /METHOD=SSTYPE(3)
  /INTERCEPT=INCLUDE
  /EMMEANS=TABLES(treatment_arm) WITH(GSRS_total_V0 =MEAN  center=MEAN) COMPARE ADJ(BONFERRONI)
  /CRITERIA=ALPHA(.05)
  /DESIGN=center GSRS_total_V0  treatment_arm.



******* subanalyses********



DO IF  (gender = 2).
RECODE lab_hb_baseline (Lowest thru 7.44=1) (7.45 thru Highest=0) INTO anemia.
END IF.
EXECUTE.

DO IF  (gender = 1).
RECODE lab_hb_baseline (Lowest thru 8.07=1) (8.08 thru Highest=0) INTO anemia.
END IF.
EXECUTE.



USE ALL.
```

```
COMPUTE filter_$=(anemia = 0).
VARIABLE LABELS filter_$ 'anemia = 0 (FILTER)'.
VALUE LABELS filter_$ 0 'Not Selected' 1 'Selected'.
FORMATS filter_$ (f1.0).
FILTER BY filter_$.
EXECUTE.

UNIANOVA Delta_6mwt_distance BY treatment_arm WITH center sixmwt_distance.v0
  /METHOD=SSTYPE(3)
  /INTERCEPT=INCLUDE
  /EMMEANS=TABLES(treatment_arm) WITH(sixmwt_distance.v0=MEAN
center=MEAN) COMPARE ADJ(BONFERRONI)
  /CRITERIA=ALPHA(.05)
  /DESIGN=center sixmwt_distance.v0 treatment_arm.

USE ALL.
COMPUTE filter_$=(anemia = 1).
VARIABLE LABELS filter_$ 'anemia = 1 (FILTER)'.
VALUE LABELS filter_$ 0 'Not Selected' 1 'Selected'.
FORMATS filter_$ (f1.0).
FILTER BY filter_$.
EXECUTE.



UNIANOVA Delta_6mwt_distance BY treatment_arm WITH center sixmwt_distance.v0
  /METHOD=SSTYPE(3)
  /INTERCEPT=INCLUDE
  /EMMEANS=TABLES(treatment_arm) WITH(sixmwt_distance.v0=MEAN
center=MEAN) COMPARE ADJ(BONFERRONI)
```

```
  /CRITERIA=ALPHA(.05)
  /DESIGN=center sixmwt_distance.v0 treatment_arm.

USE ALL.
COMPUTE filter_$=(lab_ferritine_baseline < 100).
VARIABLE LABELS filter_$ 'lab_ferritine_baseline < 100 (FILTER)'.
VALUE LABELS filter_$ 0 'Not Selected' 1 'Selected'.
FORMATS filter_$ (f1.0).
FILTER BY filter_$.
EXECUTE.


UNIANOVA Delta_6mwt_distance BY treatment_arm WITH center sixmwt_distance.v0
  /METHOD=SSTYPE(3)
  /INTERCEPT=INCLUDE
  /EMMEANS=TABLES(treatment_arm) WITH(sixmwt_distance.v0=MEAN
center=MEAN) COMPARE ADJ(BONFERRONI)
  /CRITERIA=ALPHA(.05)
  /DESIGN=center sixmwt_distance.v0 treatment_arm.

USE ALL.
COMPUTE filter_$=(lab_ferritine_baseline  >=  100).
VARIABLE LABELS filter_$ 'lab_ferritine_baseline  >=  100 (FILTER)'.
VALUE LABELS filter_$ 0 'Not Selected' 1 'Selected'.
FORMATS filter_$ (f1.0).
FILTER BY filter_$.
EXECUTE.
```

```
UNIANOVA Delta_6mwt_distance BY treatment_arm WITH center sixmwt_distance.v0
  /METHOD=SSTYPE(3)
  /INTERCEPT=INCLUDE
  /EMMEANS=TABLES(treatment_arm) WITH(sixmwt_distance.v0=MEAN
center=MEAN) COMPARE ADJ(BONFERRONI)
  /CRITERIA=ALPHA(.05)
  /DESIGN=center sixmwt_distance.v0 treatment_arm.

USE ALL.
COMPUTE filter_$=(lab_tsat_baseline < 20).
VARIABLE LABELS filter_$ 'lab_tsat_baseline < 20 (FILTER)'.
VALUE LABELS filter_$ 0 'Not Selected' 1 'Selected'.
FORMATS filter_$ (f1.0).
FILTER BY filter_$.
EXECUTE.

UNIANOVA Delta_6mwt_distance BY treatment_arm WITH center sixmwt_distance.v0
  /METHOD=SSTYPE(3)
  /INTERCEPT=INCLUDE
  /EMMEANS=TABLES(treatment_arm) WITH(sixmwt_distance.v0=MEAN
center=MEAN) COMPARE ADJ(BONFERRONI)
  /CRITERIA=ALPHA(.05)
  /DESIGN=center sixmwt_distance.v0 treatment_arm.

USE ALL.
COMPUTE filter_$=(lab_tsat_baseline  >=  20).
VARIABLE LABELS filter_$ 'lab_tsat_baseline  >=  20 (FILTER)'.
VALUE LABELS filter_$ 0 'Not Selected' 1 'Selected'.
FORMATS filter_$ (f1.0).
FILTER BY filter_$.
```

```
EXECUTE.

UNIANOVA Delta_6mwt_distance BY treatment_arm WITH center sixmwt_distance.v0
  /METHOD=SSTYPE(3)
  /INTERCEPT=INCLUDE
  /EMMEANS=TABLES(treatment_arm) WITH(sixmwt_distance.v0=MEAN
center=MEAN) COMPARE ADJ(BONFERRONI)
  /CRITERIA=ALPHA(.05)
  /DESIGN=center sixmwt_distance.v0 treatment_arm.

USE ALL.
COMPUTE filter_$=(lab_egfr_ckdepi2021cras_baseline < 60).
VARIABLE LABELS filter_$ 'lab_egfr_ckdepi2021cras_baseline < 60 (FILTER)'.
VALUE LABELS filter_$ 0 'Not Selected' 1 'Selected'.
FORMATS filter_$ (f1.0).
FILTER BY filter_$.
EXECUTE.

UNIANOVA Delta_6mwt_distance BY treatment_arm WITH center sixmwt_distance.v0
  /METHOD=SSTYPE(3)
  /INTERCEPT=INCLUDE
  /EMMEANS=TABLES(treatment_arm) WITH(sixmwt_distance.v0=MEAN
center=MEAN) COMPARE ADJ(BONFERRONI)
  /CRITERIA=ALPHA(.05)
  /DESIGN=center sixmwt_distance.v0 treatment_arm.

USE ALL.
COMPUTE filter_$=(lab_egfr_ckdepi2021cras_baseline  >=  60).
```

```
VARIABLE LABELS filter_$ 'lab_egfr_ckdepi2021cras_baseline  >=  60 (FILTER)'.
VALUE LABELS filter_$ 0 'Not Selected' 1 'Selected'.
FORMATS filter_$ (f1.0).
FILTER BY filter_$.
EXECUTE.


UNIANOVA Delta_6mwt_distance BY treatment_arm WITH center sixmwt_distance.v0
  /METHOD=SSTYPE(3)
  /INTERCEPT=INCLUDE
  /EMMEANS=TABLES(treatment_arm) WITH(sixmwt_distance.v0=MEAN
center=MEAN) COMPARE ADJ(BONFERRONI)
  /CRITERIA=ALPHA(.05)
  /DESIGN=center sixmwt_distance.v0 treatment_arm.

USE ALL.
COMPUTE filter_$=(sixmwt_distance.v0 < 522).
VARIABLE LABELS filter_$ 'sixmwt_distance.v0 < 522 (FILTER)'.
VALUE LABELS filter_$ 0 'Not Selected' 1 'Selected'.
FORMATS filter_$ (f1.0).
FILTER BY filter_$.
EXECUTE.

UNIANOVA Delta_6mwt_distance BY treatment_arm WITH center sixmwt_distance.v0
  /METHOD=SSTYPE(3)
  /INTERCEPT=INCLUDE
  /EMMEANS=TABLES(treatment_arm) WITH(sixmwt_distance.v0=MEAN
center=MEAN) COMPARE ADJ(BONFERRONI)
  /CRITERIA=ALPHA(.05)
  /DESIGN=center sixmwt_distance.v0 treatment_arm.
```

```
USE ALL.
COMPUTE filter_$=(sixmwt_distance.v0  >=  522).
VARIABLE LABELS filter_$ 'sixmwt_distance.v0  >=  522 (FILTER)'.
VALUE LABELS filter_$ 0 'Not Selected' 1 'Selected'.
FORMATS filter_$ (f1.0).
FILTER BY filter_$.
EXECUTE.


UNIANOVA Delta_6mwt_distance BY treatment_arm WITH center sixmwt_distance.v0
  /METHOD=SSTYPE(3)
  /INTERCEPT=INCLUDE
  /EMMEANS=TABLES(treatment_arm) WITH(sixmwt_distance.v0=MEAN
center=MEAN) COMPARE ADJ(BONFERRONI)
  /CRITERIA=ALPHA(.05)
  /DESIGN=center sixmwt_distance.v0 treatment_arm.

USE ALL.
COMPUTE filter_$=(lab_ntprobnp_baseline >= 142).
VARIABLE LABELS filter_$ 'lab_ntprobnp_baseline >= 142 (FILTER)'.
VALUE LABELS filter_$ 0 'Not Selected' 1 'Selected'.
FORMATS filter_$ (f1.0).
FILTER BY filter_$.
EXECUTE.

UNIANOVA Delta_6mwt_distance BY treatment_arm WITH center sixmwt_distance.v0
  /METHOD=SSTYPE(3)
  /INTERCEPT=INCLUDE
```

```
  /EMMEANS=TABLES(treatment_arm) WITH(sixmwt_distance.v0=MEAN
center=MEAN) COMPARE ADJ(BONFERRONI)
  /CRITERIA=ALPHA(.05)
  /DESIGN=center sixmwt_distance.v0 treatment_arm.

USE ALL.
COMPUTE filter_$=(lab_ntprobnp_baseline  <  142).
VARIABLE LABELS filter_$ 'lab_ntprobnp_baseline  <  142 (FILTER)'.
VALUE LABELS filter_$ 0 'Not Selected' 1 'Selected'.
FORMATS filter_$ (f1.0).
FILTER BY filter_$.
EXECUTE.

UNIANOVA Delta_6mwt_distance BY treatment_arm WITH center sixmwt_distance.v0
  /METHOD=SSTYPE(3)
  /INTERCEPT=INCLUDE
  /EMMEANS=TABLES(treatment_arm) WITH(sixmwt_distance.v0=MEAN
center=MEAN) COMPARE ADJ(BONFERRONI)
  /CRITERIA=ALPHA(.05)
  /DESIGN=center sixmwt_distance.v0 treatment_arm.

USE ALL.
COMPUTE filter_$=(gender = 1).
VARIABLE LABELS filter_$ 'gender = 1 (FILTER)'.
VALUE LABELS filter_$ 0 'Not Selected' 1 'Selected'.
FORMATS filter_$ (f1.0).
FILTER BY filter_$.
EXECUTE.

UNIANOVA Delta_6mwt_distance BY treatment_arm WITH center sixmwt_distance.v0
```

```
  /METHOD=SSTYPE(3)
  /INTERCEPT=INCLUDE
  /EMMEANS=TABLES(treatment_arm) WITH(sixmwt_distance.v0=MEAN
center=MEAN) COMPARE ADJ(BONFERRONI)
  /CRITERIA=ALPHA(.05)
  /DESIGN=center sixmwt_distance.v0 treatment_arm.

USE ALL.
COMPUTE filter_$=(gender = 2).
VARIABLE LABELS filter_$ 'gender = 2 (FILTER)'.
VALUE LABELS filter_$ 0 'Not Selected' 1 'Selected'.
FORMATS filter_$ (f1.0).
FILTER BY filter_$.
EXECUTE.

UNIANOVA Delta_6mwt_distance BY treatment_arm WITH center sixmwt_distance.v0
  /METHOD=SSTYPE(3)
  /INTERCEPT=INCLUDE
  /EMMEANS=TABLES(treatment_arm) WITH(sixmwt_distance.v0=MEAN
center=MEAN) COMPARE ADJ(BONFERRONI)
  /CRITERIA=ALPHA(.05)
  /DESIGN=center sixmwt_distance.v0 treatment_arm.


FILTER OFF.
USE ALL.
EXECUTE.
```

```
RECODE lab_ferritine_baseline (Lowest thru 99.999=1) (100 thru Highest=2) INTO
    lab_ferritine_baseline_100.
EXECUTE.

RECODE lab_tsat_baseline (Lowest thru 19.999=1) (20 thru Highest=2) INTO
    lab_tsat_baseline_20.
EXECUTE.

RECODE lab_egfr_ckdepi2021cras_baseline (Lowest thru 59.999=1) (60 thru Highest=2)
INTO
    lab_egfr_ckdepi2021cras_baseline_60.
EXECUTE.

RECODE sixmwt_distance.v0 (Lowest thru 521.999=1) (522 thru Highest=2) INTO
    sixmwt_distance.v0_522.
EXECUTE.

RECODE lab_ntprobnp_baseline (Lowest thru 141.999=1) (142 thru Highest=2) INTO
    lab_ntprobnp_baseline_142.
EXECUTE.

DO IF  (gender = 2).
RECODE lab_hb_baseline (Lowest thru 7.44=1) (7.45 thru Highest=0) INTO anemia.
END IF.
EXECUTE.

DO IF  (gender = 1).
RECODE lab_hb_baseline (Lowest thru 8.07=1) (8.08 thru Highest=0) INTO anemia.
END IF.
```

EXECUTE.

P-interaction

```
UNIANOVA Delta_6mwt_distance BY treatment_arm anemia
  /METHOD=SSTYPE(3)
  /INTERCEPT=INCLUDE
  /PLOT=PROFILE(treatment_arm*anemia) TYPE=LINE ERRORBAR=CI
MEANREFERENCE=NO
    YAXIS=AUTO
  /PRINT ETASQ DESCRIPTIVE HOMOGENEITY
  /CRITERIA=ALPHA(.05)
  /DESIGN=treatment_arm anemia treatment_arm*anemia.
```

```
UNIANOVA Delta_6mwt_distance BY treatment_arm lab_ferritine_baseline_100
  /METHOD=SSTYPE(3)
  /INTERCEPT=INCLUDE
  /PLOT=PROFILE(treatment_arm*lab_ferritine_baseline_100) TYPE=LINE
ERRORBAR=CI MEANREFERENCE=NO
    YAXIS=AUTO
  /PRINT ETASQ DESCRIPTIVE HOMOGENEITY
  /CRITERIA=ALPHA(.05)
  /DESIGN=treatment_arm lab_ferritine_baseline_100
treatment_arm*lab_ferritine_baseline_100.
```

```
UNIANOVA Delta_6mwt_distance BY treatment_arm lab_tsat_baseline_20
  /METHOD=SSTYPE(3)
  /INTERCEPT=INCLUDE
  /PLOT=PROFILE(treatment_arm*lab_tsat_baseline_20) TYPE=LINE ERRORBAR=CI
MEANREFERENCE=NO
    YAXIS=AUTO
  /PRINT ETASQ DESCRIPTIVE HOMOGENEITY
  /CRITERIA=ALPHA(.05)
  /DESIGN=treatment_arm lab_tsat_baseline_20 treatment_arm*lab_tsat_baseline_20.
```

```
UNIANOVA Delta_6mwt_distance BY treatment_arm
lab_egfr_ckdepi2021cras_baseline_60
  /METHOD=SSTYPE(3)
  /INTERCEPT=INCLUDE
  /PLOT=PROFILE(treatment_arm*lab_egfr_ckdepi2021cras_baseline_60) TYPE=LINE
ERRORBAR=CI MEANREFERENCE=NO
    YAXIS=AUTO
  /PRINT ETASQ DESCRIPTIVE HOMOGENEITY
  /CRITERIA=ALPHA(.05)
  /DESIGN=treatment_arm lab_egfr_ckdepi2021cras_baseline_60
treatment_arm*lab_egfr_ckdepi2021cras_baseline_60.
```

```
UNIANOVA Delta_6mwt_distance BY treatment_arm sixmwt_distance.v0_522
  /METHOD=SSTYPE(3)
  /INTERCEPT=INCLUDE
  /PLOT=PROFILE(treatment_arm*sixmwt_distance.v0_522) TYPE=LINE
ERRORBAR=CI MEANREFERENCE=NO
    YAXIS=AUTO
```

```
  /PRINT ETASQ DESCRIPTIVE HOMOGENEITY
  /CRITERIA=ALPHA(.05)
  /DESIGN=treatment_arm sixmwt_distance.v0_522
treatment_arm*sixmwt_distance.v0_522.
```

```
UNIANOVA Delta_6mwt_distance BY treatment_arm lab_ntprobnp_baseline_142
  /METHOD=SSTYPE(3)
  /INTERCEPT=INCLUDE
  /PLOT=PROFILE(treatment_arm*lab_ntprobnp_baseline_142) TYPE=LINE
ERRORBAR=CI MEANREFERENCE=NO
    YAXIS=AUTO
  /PRINT ETASQ DESCRIPTIVE HOMOGENEITY
  /CRITERIA=ALPHA(.05)
  /DESIGN=treatment_arm lab_ntprobnp_baseline_142
treatment_arm*lab_ntprobnp_baseline_142.
```

```
UNIANOVA Delta_6mwt_distance BY treatment_arm gender
  /METHOD=SSTYPE(3)
  /INTERCEPT=INCLUDE
  /PLOT=PROFILE(treatment_arm*gender) TYPE=LINE ERRORBAR=CI
MEANREFERENCE=NO
    YAXIS=AUTO
  /PRINT ETASQ DESCRIPTIVE HOMOGENEITY
  /CRITERIA=ALPHA(.05)
  /DESIGN=treatment_arm gender treatment_arm*gender.
```

In a separate database

```
COMPUTE SD_placebo_pooled=SE_placebo_pooled * (SQRT(N_placebo_original)).
EXECUTE.

COMPUTE SD_fcm_pooled=SE_FCM_pooled * (SQRT(N_FCM_original)).
EXECUTE.

COMPUTE CI_LB_effect_pooled=Mean_difference_pooled - 1.96 * SE_difference_pooled.
EXECUTE.

COMPUTE CI_UB_effect_pooled=Mean_difference_pooled + 1.96 * SE_difference_pooled.
EXECUTE.

COMPUTE z_value = ABS(Mean_difference_pooled / SE_difference_pooled).
EXECUTE.

COMPUTE p_value = 2 * (1 - CDF.NORMAL(z_value,0,1)).
EXECUTE.
```

Study protocol

RESEARCH PROTOCOL

(May 2022)

PROTOCOL TITLE

EFFECT OF FERRIC CARBOXYMALTOSE ON EXERCISE CAPACITY AFTER KIDNEY TRANSPLANTATION: A MULTICENTER RANDOMIZED CONTROLLED TRIAL

**Protocol ID**	**NL67065.042.18**
**Short title**	**EFFECT-KTx**
**EudraCT number**	**2018-002571-18**
**Version**	**6.2**
**Date**	**10-5-2022**
**Coordinating investigator/project leader**	**Drs. J.S.J. Vinke**<br>**Email: j.s.j.vinke@umcg.nl**
**Principal investigator(s) (in Dutch: hoofdonderzoeker/ uitvoerder)**	**UMCG (coordinating center):**<br>**Dr. M.H. de Borst, internist-nephrologist**<br>**Email: m.h.de.borst@umcg.nl**<br><br>**UMC Utrecht:**<br>**Dr. A.D. van Zuilen, internist-nephrologist**<br>**Email: a.vanzuilen@umcutrecht.nl**
**Sponsor (in Dutch: verrichter/opdrachtgever)**	**Universitair Medisch Centrum Groningen (UMCG)**<br>**Hanzeplein 1**<br>**Postbus 30001**<br>**9700 RB Groningen**
**Subsidizing party**	**Dutch Kidney Foundation, Vifor Pharma**
**Independent expert**	**Dr. A. Ozyilmaz, internist-nephrologist, Dialysis Center Groningen (DCG)**<br>**Email: a.ozyilmaz@dcg.nl**
**Laboratory sites**	**Dr. J. van den Born**<br>**Universitair Medisch Centrum Groningen (UMCG)**<br>**Hanzeplein 1**<br>**Postbus 30001**<br>**9700 RB Groningen**<br>**Email: j.van.den.born@umcg.nl**
**Pharmacy**	**Drs. T.Y.J. Appeldoorn**<br>**Email: trials@apoth.umcg.nl**

PROTOCOL SIGNATURE SHEET

**Name**	**Signature**	**Date**
**Head of Department:**<br>***Prof. Dr. R.O.B. Gans, professor in internal medicine***	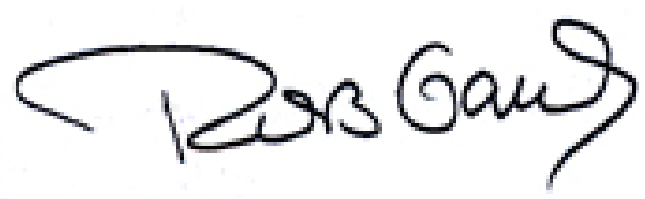	**10-5-2020**
**Principal Investigator:**<br>***Dr. M.H. de Borst, internist-nephrologist***	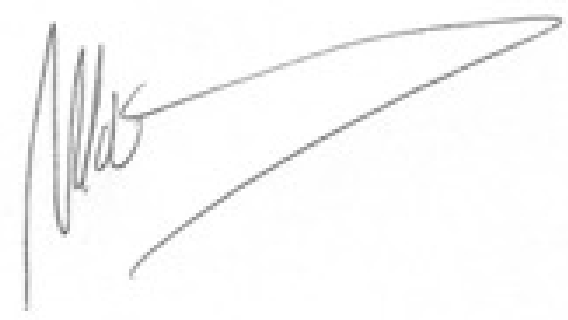	**10-5-2020**

TABLE OF CONTENTS

1. INTRODUCTION AND RATIONALE 10
2. OBJECTIVES AND HYPOTHESES 17
2.1 Primary Research Question 16
2.2 Secondary Research Questions 16
2.3 Main Hypothesis 18
2.4 Secondary Hypotheses 18
3. STUDY DESIGN 20
4. STUDY POPULATION 24
4.1 Population 24
4.2 Inclusion criteria 25
5. TREATMENT OF SUBJECTS 27
5.1 Investigational product/treatment 27
5.2 Escape medication 27
6. INVESTIGATIONAL PRODUCT 28
6.1 Name and description of investigational product(s) 28
6.2 Summary of findings from non-clinical studies 29
6.3 Summary of findings from clinical studies 29
6.4 Summary of known and potential risks and benefits 29
6.5 Description and justification of route of administration and dosage 31
6.6 Dosages, dosage modifications and method of administration 31
6.7 Preparation and labelling of Investigational Medicinal Product 32
6.8 Drug accountability 32
7 NON-INVESTIGATIONAL PRODUCT 33
7.1 Name and description of non-investigational product(s) 33
7.2 Summary of findings from non-clinical studies 33
7.3 Summary of findings from clinical studies 33
7.4 Summary of known and potential risks and benefits 33
7.5 Description and justification of route of administration and dosage 33
7.6 Dosages, dosage modifications and method of administration 33
7.7 Preparation and labelling of Non Investigational Medicinal Product 33
7.8 Drug accountability 33
8 Methods 34
8.1 Study Parameters/end points 34
ii. Secondary study parameters/endpoints 34
8.2 Randomisation, blinding and treatment allocation 35
8.3 Study Procedures 36
8.4 Withdrawal of individual subjects 45
8.5 Replacement of individual subjects after withdrawal 45
8.6 Follow-up of subjects withdrawn from treatment 45
8.7 Premature termination of the study 45
9 SAFETY REPORTING 45

9.1. Temporary halt for reasons of subject safety 45
9.2. AEs, SAEs and SUSARs 46
9.2.1. Adverse events (AEs) 46
9.2.2. Serious adverse events (SAEs) 46
9.2.3. Suspected unexpected serious adverse reactions (SUSARs) 46
9.3. Annual safety report 47
9.4. Follow-up of adverse events 48
9.5. Data Safety Monitoring Board (DSMB) 48
10. STATISTICAL ANALYSIS 49
10.1. Primary study parameter 49
10.2. Secondary study parameter(s) 50
10.3. Other study parameters 51
10.4. Interim analysis 51
11. ETHICAL CONSIDERATIONS 52
11.1. Regulation statement 52
11.2. Recruitment and consent 52
11.3. Objection by minors or incapacitated subjects 53
11.4. Benefits and risks assessment, group relatedness 53
11.5. Compensation for injury 54
11.6. Incentives (if applicable) 54
12. ADMINISTRATIVE ASPECTS, MONITORING AND PUBLICATION 55
12.1. Handling and storage of data and documents 55
12.2. Monitoring and Quality Assurance 55
12.3. Amendments 55
12.4. Annual progress report 56
12.5. Temporary halt and (prematurely) end of study report 56
12.6. Public disclosure and publication policy 56
13. STRUCTURED RISK ANALYSIS 57
13.1. Potential issues of concern 57
13.2. Synthesis 59
14. REFERENCES 60

LIST OF ABBREVIATIONS AND RELEVANT DEFINITIONS

6MWT	Six-minute walking test
ABR	ABR form, General Assessment and Registration form, the application form that is required for submission to the accredited Ethics Committee (In Dutch, ABR = Algemene Beoordeling en Registratie)
ACE	Angiotensin-converting-enzyme
AE	Adverse Event
ALAT	Alanine Aminotransferase
ASAT	Asparate Aminotransferase
BADS	Behavioral Assessment of the Dysexecutive Syndrome
BMP6	Bone Morphogenetic Protein 6
CCMO	Central Committee on Research Involving Human Subjects; in Dutch: Centrale Commissie Mensgebonden Onderzoek
CDT	Clock Drawing Test
CIS	Checklist Individual Strength
CKD	Chronic Kidney Disease
COVAC-EFFECT	Coronavirus vaccination substudy of the EFFECT-KTx study
COVID19	Coronavirus Disease 2019
COWAT	Controlled Oral Word Association Test
CST	Cognitive Screening Test
CV	Curriculum Vitae
DMFS	Dutch Multifactor Fatigue Scale
DSMB	Data Safety Monitoring Board
EDTA	Ethylenediaminetetraacetic acid
eGFR	Estimated glomerular filtration rate
ELISA	Enzyme-Linked Immuno Sorbent Assay
EudraCT	European drug regulatory affairs Clinical Trials
EQ5D-5L	Five-dimensional EuroQol
FACS	Fluorescence-activated cell-sorter
FCM	Ferric(III)Carboxymaltose
FGF23	Fibroblast Growth Factor 23
FGFR	Fibroblast Growth Factor Receptor
FTSTS	Five Times Sit To Stand
GAF	Global assessment of functioning
GIT-2	Groninger Intelligentie Test 2
Hb	Haemoglobin
hrQOL	Health related quality of life
IB	Investigator’s Brochure
IBD	Inflammatory Bowel Disease

IC	Informed Consent
IL-6	Interleukin-6
IV	Intravenous
ISC	Iron Sucrose
KDIGO	Kidney Disease: improving global outcomes
KIM-1	Kidney Injury Marker 1
LPS	Lipopolysaccharide toxin
LVEF	Left Ventricular Ejection Fraction
METC	Medical research ethics committee (MREC); in Dutch: medisch ethische toetsing commissie (METC)
MHC	Major Histocompatibility Complex
NaCl	Sodium Chloride
NLV	Dutch Adult Learning Test (in Dutch: Nederlandse Leertest voor Volwassenen)
ns	Non-significant
NYHA	New York Heart Association
PBMC	Peripheral Blood Mononuclear Cell
PTH	Parathyroid Hormone
QOL	Quality of life
RAVLT	Rey Auditory Verbal Learning Test
(S)AE	(Serious) Adverse Event
SAP	Statistical Analysis Plan
SARS-CoV2	Severe acute respiratory syndrome coronavirus 2
SD	Standard Deviation
SDMT	Symbol Digit Modalities Test
SF36	Short Form 36
SPC	Summary of Product Characteristics (Dutch: officiële productinfomatie IB1-tekst)
Sponsor	The sponsor is the party that commissions the organisation or performance of the research, for example a pharmaceutical company, academic hospital, scientific organisation or investigator. A party that provides funding for a study but does not commission it is not regarded as the sponsor, but referred to as a subsidising party.
SUSAR	Suspected Unexpected Serious Adverse Reaction
TMT	Trail Making Test
TSAT	Transferrin Saturation
TNF a	Tumor Necrosis Factor alpha
TUG	Timed Up and Go
UMCG	University Medical Center Groningen
UMCU	University Medical Center Utrecht
VO2	Oxygen Consumption rate
WAIS-IV	Wechsler Adult Intelligence Scale IV

WGBO	Medical Treatment Act (in Dutch: Wet op de Geneeskundige Behandelovereenkomst)
WMO	Medical Research Involving Human Subjects Act (in Dutch: Wet Medisch-wetenschappelijk Onderzoek met Mensen)

SUMMARY

Rationale: *Iron deficiency is common in kidney transplant recipients. The presence of iron deficiency is associated with impaired exercise tolerance and an unfavourable prognosis in these patients. In patients with heart failure and iron deficiency, treatment with intravenous iron improved exercise capacity and quality of life. Whether such beneficial effects may also occur in kidney transplant recipients is unknown.*

Objective: *Our main objective is to address whether correction of iron deficiency with ferric*(III)*carboxymaltose improves exercise tolerance and quality of life in iron-deficient kidney-transplant recipients.*

Study design: *We will perform a multicentre double-blind, placebo-controlled randomized controlled clinical trial to compare the effects of ferric*(III)*carboxymaltose with placebo.*

Study population: *158 iron-deficient kidney transplant recipients.*

I*ntervention (if applicable)*: *The intervention arm will receive 10 mL of ferric(III)carboxymaltose (50 mg* Fe^{3+}*/mL, intravenously) every six weeks, with a total of four dosages. The control arm receives an intravenous placebo solution (saline).*

Main study parameters/endpoints: *The primary endpoint is the distance walked in six minutes, as quantified by the six-minute-walking-test at the end of follow-up.*

Nature and extent of the burden and risks associated with participation, benefit and group relatedness: *Ferric*(III)*carboxymaltose has been thoroughly studied and has been approved for use in clinical practice in The Netherlands and elsewhere in various populations. It has proved to be a safe and highly effective medicine to treat iron deficiency. A potential side effect of ferric*(III)*carboxymaltose (incidence 1-10%) is hypophosphatemia: serum phosphate will be monitored regularly during the study and corrected if necessary. We expect that iron-deficient kidney transplant recipients will benefit from ferric*(III)*carboxymaltose treatment as a result of an improvement in exercise tolerance and general wellbeing.*

1. INTRODUCTION AND RATIONALE

Kidney transplantation is the treatment of choice for patients with end-stage renal disease. The prevalence of patients with a functioning kidney allograft is increasing in the Netherlands as in most western countries (Figure 1). With the advancement of surgical techniques, anesthesia and immunosuppressive regimens, graft and patient outcomes have substantially improved over the past decades. The next step in the field is to further increase quality of life for patients after kidney transplantation.

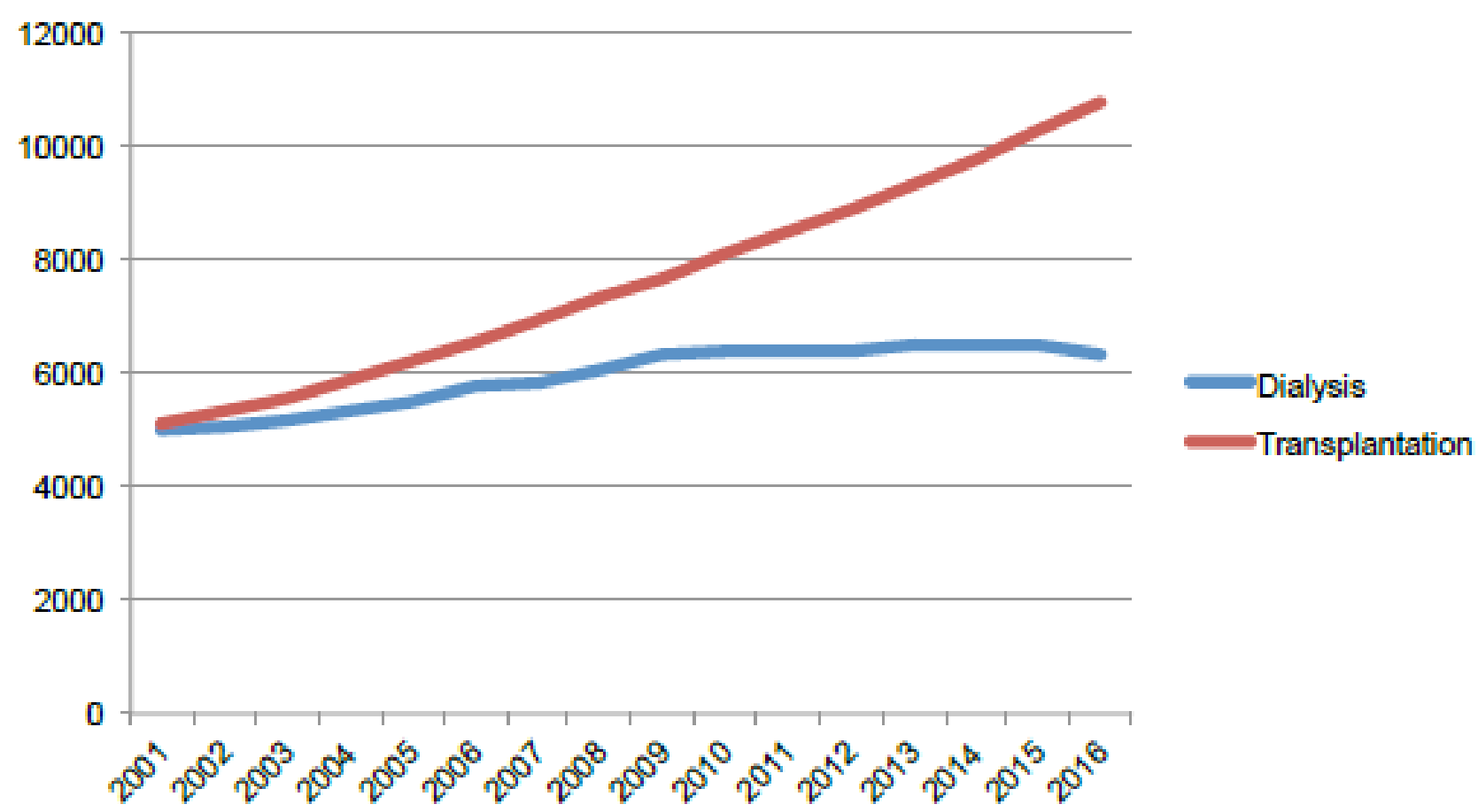

***Figure 1**. Trends in prevalence of dialysis and transplantation in the Netherlands. Source: Dutch national end-stage renal disease registry*[1]

Despite improved renal function, the post-transplantation setting is characterized by impaired exercise capacity (± 70% of the norm, Figure 2), restricting normal daily activities.[2] Currently, actionable targets to improve exercise capacity are lacking.

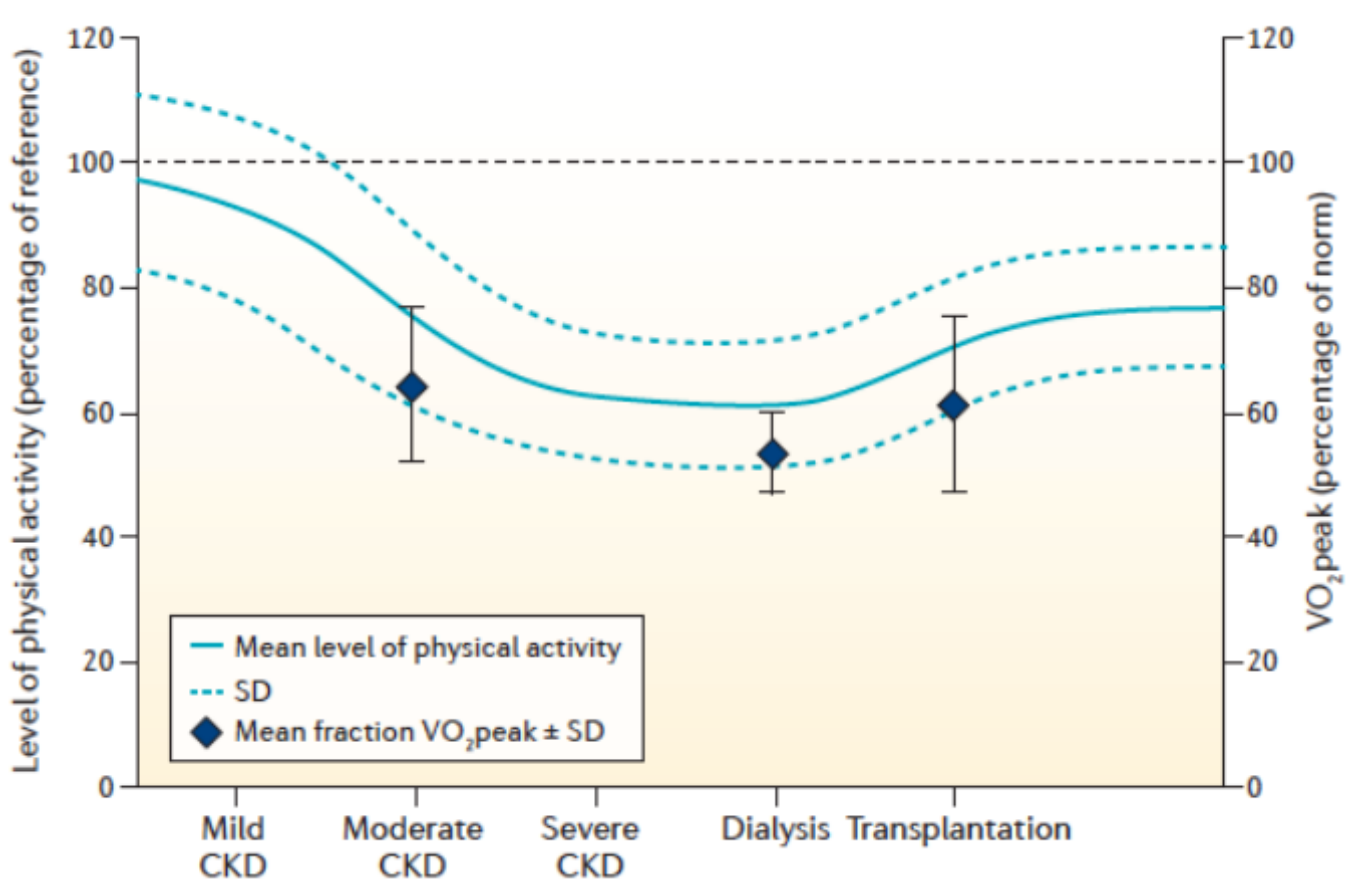

***Figure 2**. Semi-quantitative graph illustrating the average physical activity level according to the severity of CKD and transplantation status, as a percentage of age-matched healthy controls (reference, horizontal line). VO2peak values (mean and SD), providing a measure of cardiorespiratory fitness, are also shown as percentage of VO2max in healthy individuals.*

Iron deficiency is common in chronic kidney disease (CKD) patients and in kidney transplant recipients.[3] Unpublished data from our institution indicate that iron deficiency is affecting 39% of kidney transplant recipients at six months after transplantation.[4] Of these iron-deficient patients, only 39% are anaemic. In other studies, percentages of iron deficiency up to 62% have been observed in kidney transplant recipients.[5]

Iron is an important constituent of haemoglobin and myoglobin.[6] It plays a central role in oxygen transport and in energy metabolism, especially in cardiac and skeletal muscle cells. Iron is absorbed in the duodenum, a process that can be downregulated by hepcidin, an acute phase-protein. Hepcidin also decreases the bioavailability of iron by augmenting its storage in macrophages. Iron is transported by transferrin and is stored mainly as a component of ferritin. Iron status of the body can be determined by measuring serum ferritin, another acute phase protein and thus not reliable in the context of inflammation. In this case, transferrin saturation is a more accurate measurement and is normally around 33%. Iron deficiency in the context of inflammation can be defined as a serum ferritin of <100 ng/mL or a serum ferritin of 100-300 ng/mL with a transferrin saturation (TSAT) of <20%.[6]

The mechanisms behind iron deficiency in kidney transplant recipients are not yet fully understood, but several causes can be identified. In our previous studies we found a normal median hepcidin level of 7.2 ng/mL in kidney transplant recipients, but it is unknown if iron-deficient patients have a higher hepcidin.[7] Chronic inflammation in the native kidney or elsewhere in the body related to infections or an immune response to the graft could elevate hepcidin and consequently cause iron deficiency. Particularly interleukin-6 (IL-6) has been demonstrated to connect chronic diseases with iron deficiency.[8] Bone morphogenic protein 6 (BMP6), a member of the transforming growth factor beta superfamily and a modulator of the renal response to injury, is another factor inducing upregulation of hepcidin.[7]
A second theoretical explanation of iron deficiency in kidney transplant recipients is the frequent usage of proton pump inhibitors and anticoagulant therapy, which have been linked to an increased risk of iron deficiency.[9,10]
Collection of blood samples and blood loss during the transplantation procedure contribute to iron deficiency. Zheng et al observed an average blood loss of 180 mL during surgery and 600 mL because of peri-operative blood sampling.[11]
Finally, the increase of serum erythropoietin levels and the return of the menstrual cycle in women after transplantation could cause a relative shortage of iron.

Iron deficiency, with or without anaemia, has been linked to muscular weakness, restless legs, fatigue and impaired cognitive performance.[6,12–14] Multiple studies reported a strong association between iron deficiency and declined exercise tolerance in patients with heart failure.[15–17] In vitro, iron deficiency impairs mitochondrial respiration and cardiomyocyte contractility, which may be a causal factor of fatigue.[18]
In our previous studies, iron deficiency in kidney transplant recipients correlated with increased mortality, even after correction for haemoglobin level (Figure 3).[3]

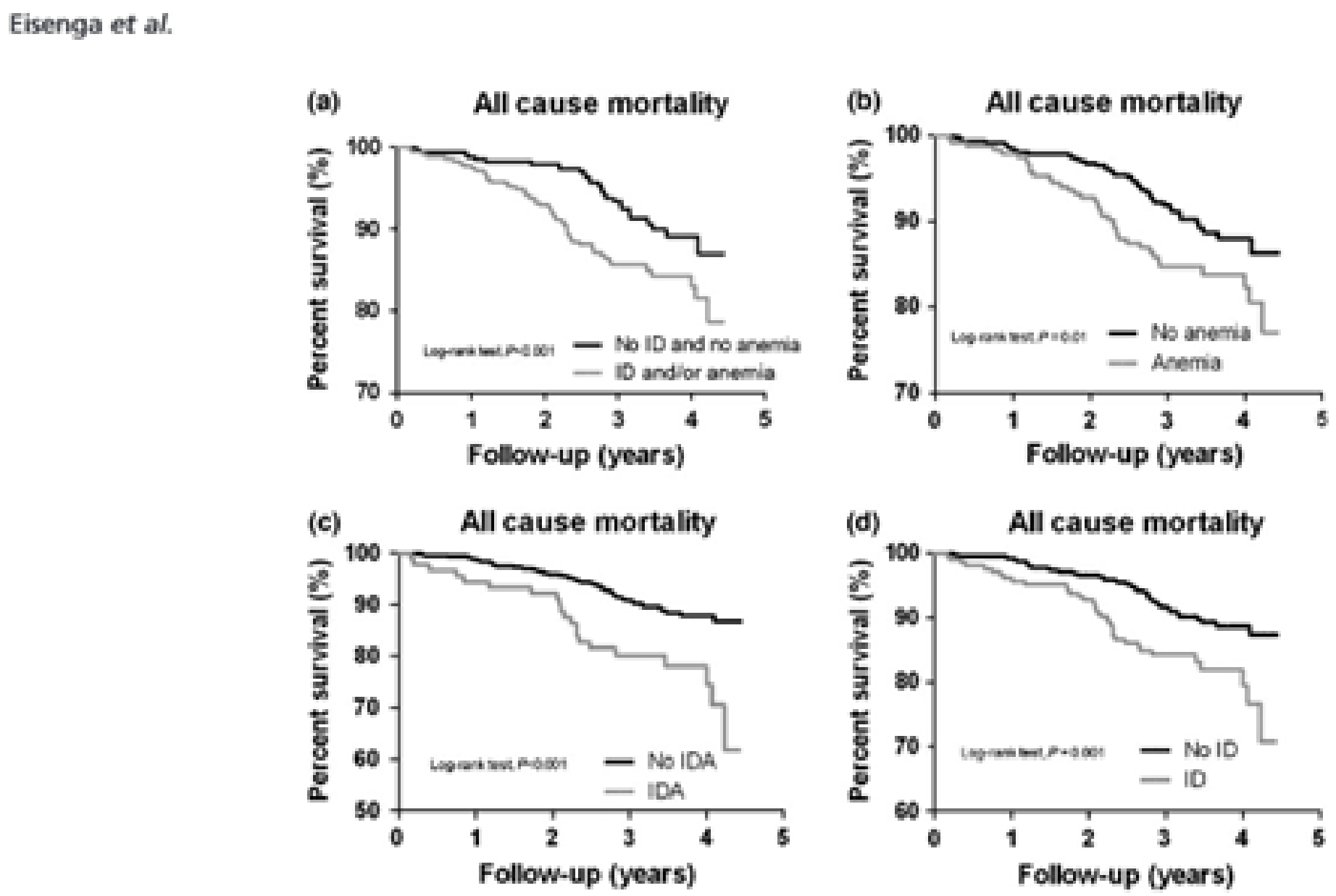

Figure 3. *Kaplan-Meier curves for the difference in patient survival in renal transplant recipients with or without iron deficiency and/or anaemia. ID, iron deficiency.*[3]

Favrat et al observed a reduction in fatigue and an improvement in cognitive function after a single dose of ferric carboxymaltose compared to placebo, in young iron-deficient women suffering from fatigue.[19]
The majority of prospective data originate from studies in the heart failure population, where recent randomized controlled trials demonstrated improved exercise capacity and quality of life after intravenous iron supplementation as summarized in table 1.[20–29]

Because of the impaired iron absorption from the bowel in the context of inflammation and chronic disease, intravenous iron preparations have a superior effect over oral preparations in chronic kidney disease.[6,30–33] In the FIND-CKD trial, a multicenter study conducted in 626 CKD-patients suffering from iron deficiency anaemia, participants who received high-dose ferric(III)carboxymaltose (500-1000mg iron every four weeks) had a significantly larger increase in haemoglobin and ferritin compared to participants who received an oral iron preparation.[31]

In the current KDIGO guidelines concerning kidney transplantation, recommendations on how to manage iron deficiency in non-anaemic patients are lacking.[34] However, given the central role of iron in energy metabolism combined with the data in the heart failure population, it seems plausible that iron deficiency is a causal factor of fatigue and impaired exercise tolerance in kidney transplant patients and that correction of iron status would increase exercise capacity and quality of life. Therefore, the main purpose of the proposed study is to investigate whether treatment with an intravenous iron-solution in the form of ferric(III)carboxymaltose will improve exercise capacity and quality of life in iron-deficient kidney transplant recipients. Furthermore, we would like to investigate the effect of this

intervention on relevant secondary endpoints including heart function, skeletal muscle function, haematologic parameters, phosphate metabolism, immune function and microbiota.

Trial	First author and reference	Year	Mean eGFR (mL/min/1.73m2)	Interventions and number per arm	Follow-up (weeks)	Outcome
	Toblli[21,22]	2007, 2015	39	I.V. ISC (n=20) I.V. saline (n=20)	24	NTproBNP↓, LVEF↑, hrQOL↑, 6MWT↑
FERRIC-HF	Okonko[23]	2008	Scr 104-109 µmol/L	I.V. ISC (n=24) I.V. saline (n=11)	18	pVO2↑, NYHA↓, GAF↑, hrQOL↑, LVEF ns
FAIR-HF	Anker[24–26]	2009, 2015	64	I.V. FCM (n=304) I.V. saline (n=155)	26	GAF↑, NYHA↓, 6MWT↑, hrQOL↑, GFR↑ (minimal effect)
CONFIRM-HF	Ponikowski[27]	2015	65	I.V. FCM (n=150) I.V. saline (n=151)	52	6MWT↑, NYHA↓, GAF↑, hrQOL↑
EFFECT-HF	Van Veldhuisen[28]	2017	52	I.V. FCM (n=86) I.V. saline (n=86)	24	pVO2 ↑, GAF↑, NYHA↓
IRON-HF	Beck da Silva[29]	2013	Scr 97 µmol/L	I.V. ISC (n=10), saline (n=6), ferrous sulphate p.o. (n=7)	12	pVO2 ns

Table 1. *Patient characteristics, interventions and primary results of RCT's addressing the effect of i.v. iron on clinically relevant outcomes in patients with chronic heart failure and iron deficiency. Scr, serum creatinine; I.V., intravenous; ISC, iron sucrose; FCM, Ferric*(III)*carboxymaltose; pVO2, peak VO2, LVEF, left ventricular ejection fraction; hrQOL, health related quality of life; NYHA, New York Heart Association Class, NTproBNP, N-terminal prohormone of brain natriuretic peptide; 6MWT, six-minute walk test; GAF, Global Assessment of Functioning; NS, non-significant.*

More specifically, we aim to address the effect of iron supplementation on plasma fibroblast growth factor 23 (FGF23). This hormone was first described in the early 2000's as the factor causing excessive phosphaturia in autosomal dominant hypophosphatemic rickets, and has since become subject of extensive research.[35–38]

FGF23 is an endocrine member of the fibroblast growth factor family and is mainly produced by osteocytes. The exact triggers are not completely understood. Oral phosphate intake causes immediate upregulation of FGF23 production, although an intravenous bolus does not have this effect. Other factors associated with increased FGF23 production are hypercalcaemia, iron deficiency and high levels of active vitamin D (1,25-dihydroxycholecalciferol). Besides promoting phosphaturia, FGF23 reduces the conversion of 25 hydroxycholecalciferol to 1,25 dihydroxycholecalciferol and inhibits the secretion of parathyroid hormone.
FGF23 has several adverse effects on the cardiovascular system. It causes left ventricular hypertrophy.[39,40] Furthermore, high levels of FGF23 are associated with heart failure, endothelial dysfunction and, in CKD patients, with a higher incidence of myocardial infarction.[38] Finally, the effect of ACE-inhibitors and salt restriction on the activity of the renin-angiotensin-aldosterone system seems to be reduced during high circulating FGF23 levels.[41,42]

In early CKD, FGF23 levels are increased and they further rise with the progression of kidney failure, up to levels of more than 1000 times the normal value in end-stage renal disease, slowing down the development of hyperphosphatemia and causing vitamin D deficiency and, probably, cardiovascular disease. Although phosphate intake seems to be a factor promoting FGF23 production in osteocytes, FGF23 is increased long before hyperphosphatemia develops. We suspect that iron deficiency might be an important factor contributing to high FGF23 levels in renal disease.

During the first weeks after renal transplantation, FGF23 levels rapidly decline but stay higher-than normal until months to years after transplantation.[43–45] In combination with hyperparathyroidism, erythropoiesis and increased cellular phosphate consumption, persistently increased levels of FGF23 in the context of a normal kidney function contribute to post-transplantation hypophosphatemia.[46] This phenomenon occurs frequently and is rarely symptomatic, but it might expose patients to a risk of decreased bone density which could be further aggravated by the use of corticosteroids. Clinicians are reserved in prescribing phosphate supplementation, because this might lead to deposition of calcium and phosphate, promoting atherosclerosis and nephrocalcinosis. Six months after the transplantation, phosphate has usually normalized and is rarely lower than 0.7 mmol/L.[46] Besides causing phosphate wasting and vitamin D deficiency, FGF23 is independently associated with all-cause mortality, cardiovascular mortality and kidney allograft loss in renal transplant recipients.[47,48]

Previous studies suggest a strong interaction between iron and FGF23. Iron deficiency is associated with a rise in total FGF23 and a stable intact FGF23 in women with menorrhagia and kidney transplant recipients.[8,49,50] Most likely, FGF23 production or secretion in osteocytes is increased in iron deficiency, but an intact enzymatic degradation function regulates intact FGF23 levels. Infusion of various iron products decreased total FGF23 by 80% on average. However, after the infusion of ferric(III)carboxymaltose or ferric oxide, but

not with other iron preparations, intact FGF23 increased in the two weeks after treatment, although other studies report an opposite effect.[49,51–53] Treatment with ferric(III)carboxymaltose may cause, or in the case of kidney transplant recipients, aggravate, hypophosphatemia by increasing FGF23.[31,54–56]
It would be interesting to gain a better insight in the effect of iron deficiency and ferric(III)carboxymaltose infusion on FGF23. Therefore, we will assess both total and intact FGF23 at baseline and at the end of the trial.

Recent studies focused on the effect of iron on inflammation and the alloimmune response after transplantation. We will analyze the effects of ferric(III)carboxymaltose on different types of leucocytes and the expression of cytokines, to get a better understanding of the results of iron supplementation on the immune system in kidney transplant recipients. Ambiguous effects of iron on lymphocytes have been reported. Iron is an essential factor in all three pathways of T-cell activation in the context of an alloimmune response: allo-antigen presentation by dendritic cells and macrophages in the context of MHC class II molecules; expression of CD28, which is involved in costimulation, and IL-2 and IL-15 mediated T-cell activation. Also, iron is involved in multiple intracellular T-cell processes such as the calcineurin pathway, the RAS-mitogen-activated pathway and the NF-κβ pathway.[57] Besides the crucial role of iron in the T-cell mediated immune response, it is also important for the humoral immune system since B-cells require iron for their proliferation. Human B-cells with immunomodulatory properties, also called Bregs, express the anti-inflammatory cytokine IL-10. However, they may also express the pro-inflammatory TNF-a. We are interested in the effect of iron on the function of B-cells and on their IL-10/TNF-a expression rate, because a low rate may predispose to graft rejection.[58] Moreover, recent studies suggest that iron deficiency reduces the immune response to vaccination.[59,60] This may be highly relevant during the current Coronavirus Disease 2019 (COVID-19) epidemic, since vaccination is the cornerstone of COVID-19 prevention. Particularly since kidney transplant recipients are known to be less responsive to other vaccinations (e.g. hepatitis B, influenza and Streptococcus Pneumoniae), it is important to identify the potential role of iron deficiency and supplementation in this context.[61] In contrast, the macrophages of the innate immune system, protecting the body from peri-operative stressors, tend to store iron during immune reactions under the influence of hepcidin and this reduces their immune effector function.[57]
Contrary to this, Vaugier et al observed a protective effect of iron abundance in kidney transplant recipients, resulting in a better graft function and a lower rate of graft failure compared to iron deficient patients, although this was only significant in a subgroup of older patients.[62] In mice, intravenous iron protected against a macrophage response to the endotoxin LPS.[62]
In conclusion, both a deficiency as well as an overload of iron might be unfavorable for graft outcome, and the immune system is involved in this phenomenon. We are interested to investigate the effect of iron deficiency correction on T- and B-granulocytes, macrophage abundance and function.

Finally, we will investigate the result of iron deficiency and intravenous iron supplementation on the gut microbiota after kidney transplantation. Human microbiota have a major interaction with the immune system and recent studies suggest an important effect of the host microbiota profile on graft survival, incidence of infections and metabolism of immunosuppressive medication.[63–65] Simultaneously, immunosuppression and ischemia-reperfusion injury have disturbing consequences for the microbiota. The impact of iron on the microbiota is still new as a topic of research in the field of transplantation. Kortman et al describe a change of the colonic climate in favor of pathogenic bacteria as a consequence of oral iron intake or supplementation.[66] In inflammatory bowel disease (IBD) patients, who are often iron-deficient, both oral as well as intravenous iron preparations influence the microbiota.[67] The EFFECT-KTx Trial will involve pioneer research of the effect of intravenous iron on the microbiota in the kidney transplant population.

This will be the first placebo-controlled using ferric(III)carboxymaltose in kidney transplant recipients. It provides us with a unique opportunity to investigate the effect of the treatment on various important clinical and biochemical outcome parameters and will be a useful source of information for future clinical studies and may influence future protocols and guidelines for management of these patients.

2. OBJECTIVES AND HYPOTHESES

Considering the background information given in the previous chapter, the following research questions and hypothesis have been formulated:

2.1 Primary Research Question

What is the effect of four six-weekly intravenous doses of 10 mL ferric(III)carboxymaltose (FCM) (50 mg Fe^{3+}/mL), versus placebo (normal saline 0.9%), on exercise capacity, quantified by the 6-minute walk test (6MWT) in iron-deficient kidney transplant recipients?

2.2 Secondary Research Questions

1. What is the effect of ferric(III)carboxymaltose versus placebo on haematologic parameters including haemoglobin, ferritin and TSAT in iron-deficient kidney transplant recipients?

2. What is the effect of ferric(III)carboxymaltose versus placebo on health-related quality of life, quantified by the Short-Form-36 (SF-36) questionnaire, the 5-Dimensial EuroQol (EQ-5D-5L) instrument, the Checklist Individual Strenght (CIS) and the Dutch Multifactor Fatigue Scale (DMFS) in iron-deficient kidney transplant recipients?

3. What is the effect of ferric(III)carboxymaltose versus placebo on systolic and diastolic cardiac function, measured by transthoracic echography, NTproBNP and troponin-T, in iron-deficient kidney transplant recipients?

4. What is the effect of ferric(III)carboxymaltose versus placebo on skeletal muscle function, measured by a variety of muscle function tests in iron-deficient kidney transplant recipients?

5. What is the effect of ferric(III)carboxymaltose versus placebo on mineral and bone parameters, including levels of serum and urinary phosphate, calcium, PTH, vitamin D and total and intact FGF23, in iron-deficient kidney transplant recipients?

6. What is the effect of ferric(III)carboxymaltose versus placebo on the intestinal microbiota in iron-deficient kidney transplant recipients?

7. What is the effect of ferric(III)carboxymaltose versus placebo on the incidence of infections, hospitalizations, cardiovascular events, acute rejection and mortality in iron-deficient kidney transplant recipients?

8. What is the effect of ferric(III)carboxymaltose versus placebo on B- and T-cell function?

9. What is the effect of ferric(III)carboxymaltose versus placebo on cognitive function, quantified by neuropsychological testing, in iron-deficient kidney transplant recipients?

10. What is the effect of ferric(III)carboxymaltose versus placebo on severe acute respiratory syndrome coronavirus 2 (SARS-CoV2) specific antibody titre and T-lymphocyte response after vaccination in iron-deficient kidney transplant recipients?

2.3 Main Hypothesis

Compared to placebo, ferric(III)carboxymaltose infusion will improve exercise tolerance in iron-deficient kidney transplant recipients.

2.4 Secondary Hypotheses

1. Compared to placebo, ferric(III)carboxymaltose infusion will increase ferritin levels and TSAT in iron-deficient kidney transplant recipients.

2. Compared to placebo, ferric(III)carboxymaltose infusion will improve quality of life in iron-deficient kidney transplant recipients.

3. Compared to placebo, ferric(III)carboxymaltose infusion will improve systolic and diastolic cardiac function in iron-deficient kidney transplant recipients with a decreased cardiac function at baseline.

4. Compared to placebo, ferric(III)carboxymaltose infusion will improve skeletal muscle strength and endurance in iron-deficient kidney transplant recipients.

5. Compared to placebo, ferric(III)carboxymaltose infusion will decrease total FGF23 levels after an initial increase in iron-deficient kidney transplant recipients. We expect an initial aggravation of phosphaturia followed by stabilization of phosphate levels.

6. Compared to placebo, ferric(III)carboxymaltose infusion will change gut microbiota in iron-deficient kidney transplant recipients.

7. Compared to placebo, ferric(III)carboxymaltose infusion will have no effect on the incidence of infections and acute rejection and will protect against cardiovascular events and mortality in iron-deficient kidney transplant recipients.

8. Compared to placebo, ferric(III)carboxymaltose infusion will improve T-cell and B-cell function in iron-deficient kidney transplant recipients, but we don't expect a significant increase in kidney allograft rejection.

9. Compared to placebo, ferric(III)carboxymaltose infusion will improve the ability to concentrate on tasks in iron-deficient kidney transplant recipients.

10. Compared to placebo, ferric(III)carboxymaltose infusion will improve S-protein-specific antibody response and T-lymphocyte response after vaccination against SARS-CoV2 in iron-deficient kidney transplant recipients, leading to enhanced vaccine efficacy.

3. STUDY DESIGN

The study is designed as a 24 week, multicenter, randomized placebo-controlled clinical trial with two parallel arms to investigate the effect of ferric(III)carboxymaltose on exercise tolerance, heart and skeletal muscle function, quality of life, the gut microbiota and on the immune system, to be performed at the University Medical Center Groningen (UMCG) and the , University Medical Center Utrecht (UMCU).

3.1 Screening and Inclusion

Patients are recruited at the UMCG (local coordinator: Dr. M.H. de Borst) and UMCU (local coordinator: Dr. A.D. van Zuilen), The Netherlands. All kidney transplant recipients visiting the outpatient clinic of one of both centres are being screened. Those patients who meet the inclusion criteria according to their nephrologist will be asked for permission to be called by the researcher. During this telephone call, the researcher informs the patient about the details of the study. In case the patient is interested to participate, he or she is invited for a pre-baseline screening visit with laboratory investigation. During this screening visit, the researcher will evaluate the inclusion and exclusion criteria again and will ask the patient to sign the informed consent form.
Subsequently, patients will be randomized to receive either intravenous ferric(III)carboxymaltose (FCM) or placebo treatment (saline solution) during the next visit, which is considered the baseline visit.The treatment phase of the EFFECT-KTx Trial will be 24 weeks. Our aim is to include 158 participants.

During the first year following kidney transplantation, patients are usually seen by a nephrologist in an academic hospital, respectively the UMCG and UMCU, before they are referred back to a local hospital in their community and come back to the academic center only once per year. An exception are patients who were already in follow-up at the academic hospital before transplantation: these patients remain in more frequent follow-up (three to four times per year) at the academic hospital.
At the UMCG, around 70% patients are included in a large cohort study, called Transplant Lines. Participation in Transplant Lines comprehends extensive testing at six and twelve months after transplantation. In the UMCU, transplant recipients are not included in this cohort study.

Most participants in the EFFECT-KTx trial are recruited between around six months after transplantation. In this case, study visits coincide with regular six-weekly visits to the outpatient clinic. Moreover, participants who are also recruited for Transplant Lines benefit from a large overlap of tests at six and twelve months. Other patients will are recruited at visits later after transplantation during their visits to the academic hospital. For those participants, not all study visits overlap with regular care visits and extra visits to the hospital are inevitable. We make an effort to limit this inconvenience.

Recruitment or inclusion in the first four months following transplantation is not allowed, because post-surgical iron deficiency is expected to be a self-limiting phenomenon in most cases and is not the topic of this study.[11]

3.2 Expected Inclusion Time

Based on the number of kidney transplantations in the UMCG and the UMCU together and an incidence of iron deficiency of 30-40%, we expected 120 newly transplanted patients per year to meet the inclusion criteria. Assuming that 50% of the patients will agree to sign informed consent, we expected to enroll around 60 recently transplanted patients per year. Because we include an additional number of patients who have undergone a transplantation more than a year ago, we originally expected the inclusion process to take no longer than two years. However, due to the COVID-19 epidemic, which has limited the possibilities to invite patients for study visits, there is a delay in inclusion rate of about one year.

3.3 Tests

The baseline visit (t=0) will consist of an extensive medical history and physical examination. In addition to blood sampling, the patient will be asked to bring a container of a 24-hour urine collection and a pot of faeces, which will be analysed and stored in a biobank.

A cardiac ultrasound will be performed to evaluate systolic and diastolic function. Flow and pressure in the atria and ventricles will be assessed, wall thickness and the left ventricular ejection fraction will be measured on the spot and images will be analysed at a later stage for strain.

To measure endurance and muscle strength, participants will be asked to do a six-minute walk test (6MWT), the ‘Five-Times-Sit-to-Stand-test’ (FTSTS) and the Timed-up-and-Go test (TUG) and to undergo a handgrip dynamometry.

Participants will be interviewed using four questionnaires concerning quality of life and fatigue. Finally, the patients who also participate in the Transplant Lines Cohort Study undergo multiple neuropsychological tests. We will use these results for our analyses as well and extend the testing to the other patients who visit the UMCG. Participants will also be asked to fill in a food diary during three days. The results will be used for interpretation of the data concerning microbiota. Neuropsychological tests are performed at the UMCG study site by neuropsychology students and take 45 to 60 minutes for each patient. The specific tests and questionnaires are described in detail in chapter 8. Participants at the UMCU study site will not undergo neuropsychological assessment, because of logistic limitations.
At 24 weeks after baseline, all examinations will be repeated. During interim visits at the outpatient clinic at six, twelve and 18 weeks after baseline, limited laboratory testing of blood and urine will be done. Iron status of later visits will only be exposed to unblinded personnel.

3.4 Treatment and Withdrawal

An overview of the study design is provided in Figure 4. Administration of the study medication (ferric(III)carboxymaltose or placebo) will take place at the beginning of the study (t=0) and after six (t=6), twelve (t=12) and 18 (t=18) weeks.
Ferric(III)carboxymaltose will be replaced by placebo in case of iron overload, defined as a serum ferritin level of 800 ug/L or more, or a ferritin level of 500-799 ug/L in combination with a TSAT of at least 45%.[68,69] However, treatment allocation will be maintained and the participant will remain in the intervention arm and analysed as such according to the intention to treat principle. In the case of intermediate or severe hypophosphatemia, defined by a phosphate level of 0.50 mmol/L or lower, no ferric(III)carboxymaltose or placebo will be administered until the phosphate level is again above this level.
In the event of serious adverse effects, for example an allergic reaction, participants will be unblinded and withdrawn from further treatment, but they will be analysed according to the intention to treat principle.
Also, in case of a systemic infection, known pregnancy or a blood pressure of more than 160/100 mmHg, participants will not receive study medication. All patients who have been withdrawn or who dropped out from the study prematurely but who received at least one dose of medication will be asked to participate in a check-out-visit, including all examinations that otherwise would have been performed at 24 weeks.

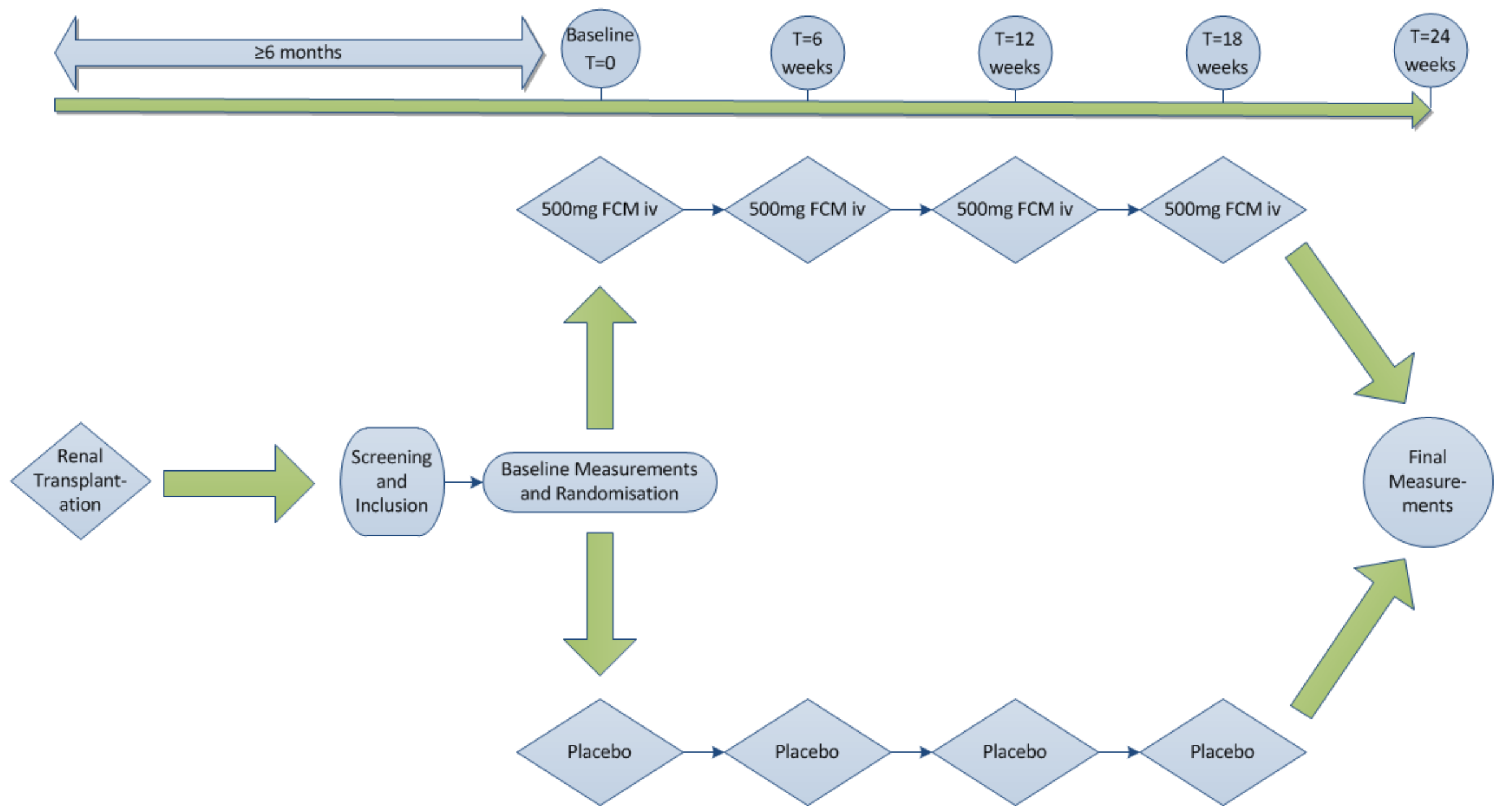

Figure 4. *Study Design. FCM = Ferric(III)Carboxymaltose.*

3.5. Management of missed study visits and drug administrations due to the COVID19 epidemic

Our initial plan was to allow participants who miss study administrations to continue their participation and to analyse their data according to the intention-to-treat principle. However, due to the unforeseen circumstances resulting from the COVID19 epidemic, all study drug administrations between March 25th, 2020 and May 22th, 2020 had to be cancelled, resulting

in an unacceptably high number of missed study drug administrations. When a participant misses two or more study drug administrations, with at least one administration missed because of the COVID19 epidemic, his or her study participation will therefore be terminated. A new participant will be recruited instead.
Participants who are excluded because of missed study drug administrations during the COVID19 epidemic may be re-screened.
Participants who miss two or more study drug administrations because of other reasons will not be withdrawn from the study and will be analysed according to the intention-to-treat principle, as described elsewhere in this protocol.
End of Study Visits which cannot be accomplished at the study site because of the COVID19 epidemic restrictions may be performed at the patient’s home.

3.6 The COVID19 vaccination substudy of the EFFECT-KTx trial (COVAC-EFFECT)

In the first half of 2021, KTRs will be invited to be vaccinated against SARS-CoV2 as part of the national vaccination campaign. This substudy, which is performed at the UMCG study site only, will address the immune response to SARS-CoV2 vaccination that EFFECT-KTx participants will receive in the context of the national vaccination campaign. Three vaccines are currently approved for use in the European Union, all comprising two injections which should be administered with an interval of at least three or four weeks. Immunosuppressive medication as well as iron deficiency might impair efficacy of vaccination against SARS-CoV2. We aim to assess the effect of prior intravenous iron treatment on SARS-CoV2 specific antibody and T-lymphocyte response after vaccination in iron-deficient KTRs. The effect of repeated intravenous ferric(III)carboxymaltose administrations is likely to result in a sustained correction of iron deficiency (up to one year). Therefore, former and current participants of the EFFECT-KTx study at the UMCG study site are eligible for participation in COVAC-EFFECT when they (1) received at least one FCM or placebo treatment in the year before the first SARS-CoV2 vaccination, (2) do not have a previous or current proven diagnosis of COVID-19 and (3) have given informed consent to be asked for participation in supplementary studies. Eligible patients will receive a supplementary patient information letter including a supplementary informed consent form and will be contacted by the study coordinator within 5 days. Because of the low impact of participation in this observational substudy and for logistic reasons, patients will be allowed to fill in the informed consent form at home. In these cases, the study doctor will sign the form at a later moment and send a copy to the patient. We expect 40 to 50 EFFECT-KTx study participants to meet the inclusion criteria and give consent for participation in the COVAC-EFFECT substudy.

At four time points, SARS-CoV2-specific antibodies will be measured using capillary blood quantitative antibody measurements available through Sanquin, The Netherlands. This assay allows patients to perform the measurement at home, and send in capillary blood samples by regular mail for analysis. Patients will be asked to contact the EFFECT-KTx study coordinator as soon as they receive an invitation for vaccination. A capillary blood collection kit will be sent to their home and they will be asked to collect a blood sample on the day before the first and second vaccine injections as well as six and twelve months after the

second injection. Moreover, at 28 days after the second injection of the vaccination, participants will be asked to come to the UMCG for one venous blood collection. SARS-CoV2-specific antibodies will be measured using the same assay provided by Sanquin on venous blood. At this visit, additional blood samples will be collected for isolation of PBMCs which will be used for analysis of SARS-CoV2 specific T-lymphocyte response. In total, two 10 mL blood tubes and one 5 mL blood tube will be taken from the patient during this visit (see Table 4, page 43).

As part of the national vaccination campaign, patients will receive a SARS-CoV2 vaccination registration card containing information about the type and batch of the vaccine and about the date of the vaccination. Participants will be asked to send a copy of this card to the researcher, so these data can be registered. Participants will also be asked to inform the researcher about any (severe) adverse events around the vaccination, even in case the participant has already completed the EFFECT KTx study. In case of an adverse event that might be related to the vaccination, the National Adverse Event Evaluation Registration (dutch acronym: Lareb) will be notified. In case of severe adverse events, the medical ethical committee will also be notified.

Because of the urgency to improve SARS-CoV2 vaccine efficacy in KTRs, results of this substudy will be analysed before completion of the main EFFECT-KTx study. A researcher who is not otherwise involved in the trial will be unblinded by the unblinded study nurse to analyse the results. The blinded study personnel will not be unblinded before completion of the main EFFECT-KTx trial.

4. STUDY POPULATION

4.1 Population

Kidney transplant recipients with iron deficiency will be invited to enter the EFFECT-KTx trial starting from 6 months after the transplantation.

In the UMCG, 184 adult patients underwent a kidney transplantation in 2017. 56% of them were male. Their age varied from 18 to 79 years and was on average 55 years. The mean BMI was 26.5 kg/m2 and the average creatinine clearance at 6 months follow-up was 48 ml/min. 25% of the kidney transplant recipients were diabetic and required oral glucose-lowering drugs or insulin. 18% of patients were active smokers.[4] We anticipate that the patient population recruited for the EFFECT-KTx trial will be highly similar.

At one year after transplantation, 30-39% of kidney transplant recipients had an iron deficiency.[3] Based on previous studies, the prevalence of iron deficiency may be even higher at later stages after kidney transplantation.[5]

A small pilot analysis of the patients who had undergone a kidney transplantation in the UMCG in the first six months of 2018 was performed. 63% of 56 patients was iron-deficient after three months. 86% of the iron-deficient patients had a hemoglobin level of at least 6.5

mmol/L. Six months after the transplantation, 54% of 33 patients had iron deficiency. Only one of those patients had a hemoglobin level below 6.5 mmol/L.

4.2 Inclusion criteria

In order to be eligible to participate in this study, an individual must meet all of the following criteria (at the time of screening and at the time of inclusion during the pre-baseline visit):

1. Kidney transplant recipient
2. Iron deficiency, defined by a ferritin level of ≤100 ug/L, or 100-299 ug/L combined with a transferrin saturation of ≤20%
3. At least four months after transplantation at the time of inclusion (and six months after transplantation at baseline)
4. Age ≥18 years
5. Ability to comply with the study protocol
6. Informed consent

4.3 Exclusion Criteria

A potential subject who meets any of the following criteria at the time of screening and at the time of inclusion during the pre-baseline visit will be excluded from participation in the EFFECT-KTx Trial:

1. Intolerance of any intravenous iron solution
2. Severe anemia (Hb <10.4 g/dL, <6.5 mmol/L), microcytic anemia or progressive anemia (>1 mmol/L per month decline for two months or more)
3. A positive feces occult blood test or otherwise demonstrated gastrointestinal or urogenital, blood loss
4. Blood transfusion in the past six weeks
5. Polycythemia (Hb >15.3 g/dL, 9.5 mmol/L)
6. History of haemochromatosis
7. An estimated glomerular filtration rate (eGFR) of ≤ 30 ml/min per 1.73 m^2 at screening.
8. Resting heart rate of more than 120 per minute
9. Unstable angina or myocardial infarction during the previous month
10. Disability to walk
11. Severe hypophosphatemia in the month before baseline (serum phosphate <0.35 mmol/L)
12. Pregnancy or inability to take adequate contraceptive measures when at childbearing age
13. Severe hyponatremia (Na <130 mmol/L) or fluid overload
14. Participation in another interventional study

4.4 Sample Size Calculation

Based on an average six-minute walking distance of 495 meters with a standard deviation of 92 meters, as was observed in a previous study in kidney transplant recipients, a preferred increase in walking distance of at least ten percent, a desired power of 0.90 and a significance level of 0.05, we calculate that a sample size of 72 participants per arm is required.[70,71] To take account for a 10% drop-out percentage, we will include 79 participants per group and 158 participants in total. We decided to calculate the sample size using a desired power of 0.90 to prevent a type II error from occurring.

40 participants are expected to meet the inclusion criteria for participation in the COVAC-EFFECT substudy. This will provide 80% power to detect a 50% difference in antibody titres between the FCM and placebo groups with an alpha of 0.05.

5. TREATMENT OF SUBJECTS

5.1 Investigational product/treatment

The investigational product will be an intravenous iron preparation, called ferric(III)carboxymaltose or ‘Ferinject©’, of which we will investigate biochemical and clinical effects compared to a placebo. The manufacturer will be Vifor Pharma Ltd. The intervention group will be treated with four dosages of 500 mg iron in the form of 10 mL ferric(III)carboxymaltose dissolved in 240 mL of NaCl 0.9%, with interval periods of six weeks. The placebo-controlled group will receive equal volumes of NaCl 0.9% solution.

5.2 Use of co-intervention

To be able to make a valid comparison between the use of an intravenous iron solution and placebo, the patient’s transplant physician will be asked not to treat the iron deficiency with oral iron preparations. In the case of severe iron deficiency anaemia, patients will be excluded from the study and further examination of the cause is advised. Participating women will have to prevent getting pregnant during the study.

5.3 Escape medication

Not applicable to the EFFECT-KTx study

5.4 Safety Precautions

Since ferric(III)carboxymaltose is being used routinely in clinical practise, there is extensive experience with this product in our department. During and after the treatment with the investigational product or the placebo, a medical doctor trained in treating the possible adverse reactions will be available. Facilities for cardiopulmonary resuscitation and treatment of acute anaphylactic reactions, including 1:1000 adrenalin solution, will be immediately available.
In case of paravenous leakage, an allergic reaction or other signs of intolerance, the administration of the product will be stopped and the symptoms will be treated according to protocol.

After the administration of the investigational product or the placebo, the participant will have to wait for at least 30 minutes before departure. A nurse or a specially trained doctor’s assistant will perform checks of the vital signs before and after administration and before the patient leaves the treatment room (after 30 minutes). In case of any symptoms, the nurse or doctor’s assistant will also perform a check of the vital signs and discuss with a doctor (either one of the investigators or the attending doctor of the ward).

6. INVESTIGATIONAL PRODUCT

6.1 Name and description of investigational product(s)

The active compound of the investigated product is ferric(III)carboxymaltose, with a concentration of 50 mg Fe^{3+}/mL.[72,73]

Additives are:

- Sodiumhydroxide (E524)
- Nitrogen (E941)
- Hydrochloric acid (E507)
- Water

Ferric(III)carboxymaltose is produced by Vifor Pharma Ltd in Switzerland. It will be administered in doses of 10mL (containing 500 mg of iron), dispersed in 240 mL of 0.9% sodiumchloride solution, during at least 15 minutes. Ferric(III)carboxymaltose is a dark-brown non-transparent solution.

The product that will be used as a placebo is sodiumchloride solution (NaCl 0.9%). It is a colourless, transparent solution of which 250 mL will be administered. It is supplied by Baxter B.V. in the Netherlands.

The placebo product comprises:

- Sodiumchloride (9 grams per liter, 2.3 grams per dose)
- Water

Because of the difference in colour between ferric(III)carboxymaltose and the placebo solution, black infusion lines produced by BBraun or white non-transparent infusion lines produced by Codan will be used, or the transparent infusion lines will be covered with aluminium foil, to prevent unblinding of the participants (Figure 5).[74] Infusion bags will also be covered with aluminium foil.

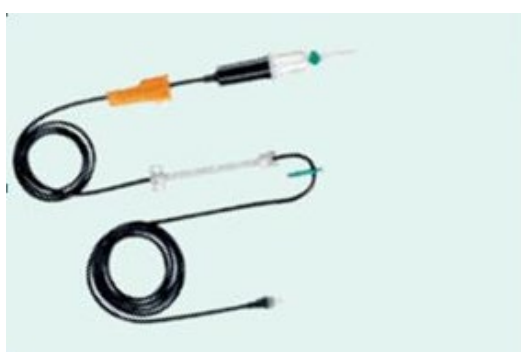

Beschreibung	Länge (cm)	Länge ab Pumpen-segment
Typ Opaque PVC (DEHP-frei): Schwarze Leitung für lichtempfindliche Infusionslösungen (100% Lichtschutz)	250	145 cm

Figure 5. *Black syringes and infusion lines as produced by BBraun.*

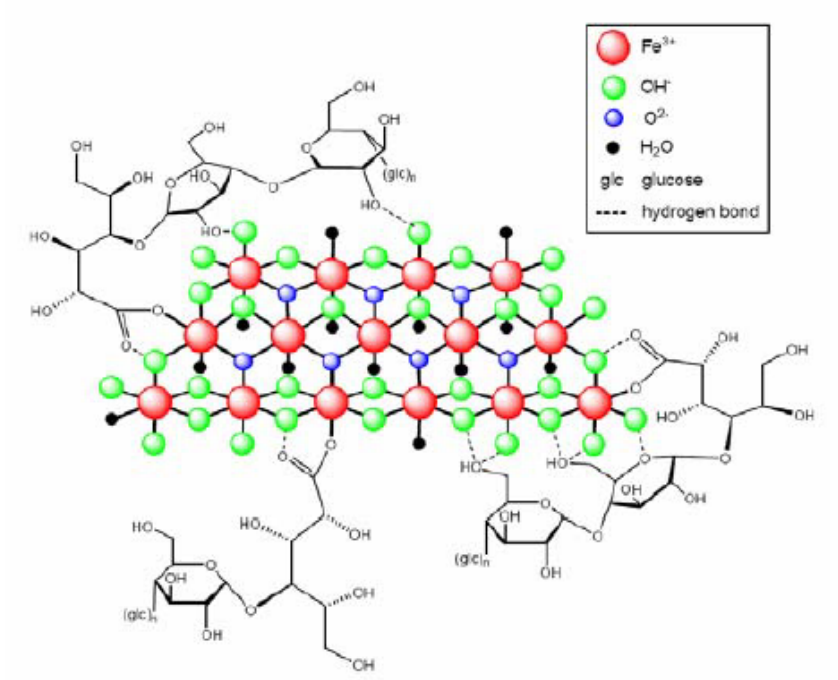

Figure 6. *Molecular Structure of Ferric(III)Carboxymaltose*

6.2 Summary of findings from non-clinical studies

As described in the manufacturers' brochure of ferric(III)carboxymaltose (Module 2 Paragraph 5.3 and Module 5 Paragraph 3, page 8 and page 19 to 30), pre-clinical safety studies reveal no serious hazard for humans, in terms of toxicity and gonadotoxicity.[72] In animal studies, iron does cross the placenta and is excreted in milk. The use of ferric(III)carboxymaltose results in minor skeletal abnormalities of the foetus and is therefore not safe in the first trimester of pregnancy. No long-term preclinical data about carcinogenic potential of ferric(III)carboxymaltose are available.

In the case of the placebo preparation, saline, no preclinical studies have been performed. All components of the product are physiological components of human plasma. No toxic effects of the placebo product can be expected.

6.3 Summary of findings from clinical studies

Clinical studies concerning ferric(III)carboxymaltose are described in the manufacturers' brochure.[72] In summary, ferric(III)carboxymaltose is an effective treatment option for patients with iron deficiency with a favourable risk profile. Safety concerns are hypersensitivity reactions to the carbohydrate group or to free iron in case of an overload. Also, there is a potential for haemosiderosis. In clinical studies, side effects were limited. The only frequently occurring adverse events were headache and mild hypotension. In contrast to other intravenous iron solutions, especially iron dextran, few hypersensitivity reactions or cases or iron overload were reported with the use of ferric(III)carboxymaltose

As explained in the previous paragraph, no clinical safety studies concerning the placebo preparation, saline, have been performed, because all its compound are physiological body compounds. However, in quantities much larger than our study's dose, hyponatraemia could theoretically occur in patients with heart failure, especially in combination with the use of corticosteroids.

6.4 Summary of known and potential risks and benefits

Ferric(III)carboxymaltose is frequently used as an intravenous iron preparation and is well tolerated and safe, although some theoretical concerns can be identified.[30,33,75] Reported side-effects include headache, dizziness, nausea, abdominal pain, constipation, diarrhea, rash and injection-side reactions. In the FIND-CKD trial, there was no significantly higher incidence of adverse events observed in ferric(III)carboxymaltose treated patients compared with patients treated with an oral iron preparation, although the oral preparation was associated with a significantly higher incidence of gastrointestinal symptoms (41% versus 23% in the ferric(III)carboxymaltose group).[31] Two out of 304 patients who received ferric(III)carboxymaltose experienced a drug hypersensitivity reaction, one of which was mild and the other one was moderate in severity. No other serious adverse events were considered to be related to the treatment.

Moore at al performed a meta-analysis of 14 studies with 2348 patients exposed to ferric(III)carboxymaltose, 832 patients exposed to oral iron preparations and 762 participants

exposed to placebo.[32] Incidence of serious adverse events and mortality rates were similar in ferric(III)carboxymaltose and comparators, while constipation, nausea and vomiting occurred more often with oral iron.
Intravenous administration of iron could theoretically result in high concentrations of free iron with toxic reactions due to oxidative stress. This does occur with other intravenous iron solutions. In ferric(III)carboxymaltose however, free iron concentrations after administration have been proved to be relatively low because it is rapidly cleared from the circulation and distributed to the bone marrow and other tissues.[33] There is no evidence of the occurrence of toxic iron reactions with ferric(III)carboxymaltose.[30]

Another potential concern with the administration of ferric(III)carboxymaltose is the development of hypophosphatemia. Transient hypophosphatemia is frequently observed in the first months after kidney transplantation and is usually asymptomatic. Treatment with ferric(III)carboxymaltose and other intravenous iron solutions is also associated with a decline in serum phosphate. Most likely, the underlying mechanism is the increase of erythropoiesis after iron supplementation and a ferric(III)carboxymaltose -induced rise of FGF23.[54] In the FIND-CKD trial, the mean serum phosphate level of patients with CKD decreased by 0.18 mmol/L from a baseline value of 1.22 (0.2) mmol/L in the first four weeks after ferric(III)carboxymaltose infusion, and returned to baseline in the following months. Recently, Wolf at al published the results of a randomized controlled trial to compare the effects on phosphate levels of ferric(III)carboxymaltose versus ferumoxytol in 1997 adults with iron deficiency anemia.[76] FCM was significantly associated with a higher incidence of hypophosphatemia (<0.65 mmol/L, 50.8% vs 0.9%; <0.42 mmol/L 10.0% vs 0.0%), with a nadir at week 2. However, much higher dosages of FCM were used in this trial (1500mg in 2 dosages of 750mg in a week) compared to our proposed trial (500mg per six weeks).
Cases of deep symptomatic hypophosphatemia and even osteomalacia after iron infusion have been reported, mostly upon administration of high-doses of intravenous iron or with relatively short intervals between doses.[55] In a small cohort of 23 renal transplant recipients who had received 100mg (n=1), 500mg (n=3) or 1000mg (n=19) Ferric(III)carboxymaltose, mean serum phosphate levels decreased by 0.27 mmol/L on average.[56] 34.8% of patients developed a hypophosphatemia (<0.5 mmol/L) with a median time to hypophosphatemia of 15 days.[56] Only one patient needed short-term phosphate supplementation.[56]
It has been suggested that long-term administration of iron infusion may, after many years, contribute to the development of osteomalacia.[54,77–79] In our study, the administration of four relatively low doses of ferric(III)carboxymaltose over 24 weeks in unlikely to elicit bone abnormalities.
In order to address the risk of hypophosphatemia, it will be necessary to closely monitor the phosphate levels and exclude patients with severe hypophosphatemia at baseline. Phospate levels will be assessed at every study visit. Severe hypophosphatemia will be treated with oral phosphate solution. Furthermore, we will prescribe relatively low dosages of 500 mg iron in the form of ferric(III)carboxymaltose with a six week interval, to prevent the occurrence of hypophosphatemia. Study medication will only be administered if the serum phosphate level is above 0.5 mmol/L.

Previously, the question has been raised whether iron administration could cause an increased susceptibility to infections, since pathogens need iron to proliferate. This would be a specific concern in kidney transplant recipients, whose immune system is suppressed.[6] There is little evidence of an increased risk of infection after treatment with ferric(III)carboxymaltose. In the FIND-CKD trial, 33.6% patients who received ferric(III)carboxymaltose suffered from a systemic infection during follow-up, compared to 30.4% in the orally treated group. This difference was not significant.[31]

Finally, in the presence of hepcidin and underexpression of ferroportin on macrophages, iron supplementation could in theory cause excessive iron storage in the reticulo-endothelial system, promoting the formation of foam cells and atherosclerosis.[80] This is however a theoretical concern for which there is no clinical evidence.
Possible concerns with the use of large quantities of sodium-chloride solution, the placebo product, are fluid overload, hyponatremia, hypokalemia or local skin reactions. However, no serious adverse reactions can be expected from the administration of 250 mL saline solution.

6.5 Description and justification of route of administration and dosage

Active treatment will consist of ferric(III)carboxymaltose administered intravenously in individual dosages of 500 mg iron (=10 mL), dispersed in 240 mL of 0.9% saline solution.

Control treatment (placebo) will be sodium-chloride solution (saline, 0.9%), administrated intravenously as well, in individual dosages of 250 mL.

6.6 Dosages, dosage modifications and method of administration

Patients will receive four infusions of the study medication (active treatment or placebo) at six week intervals. There will be no individual modifications in dosage. Patients may receive placebo while in the active treatment group in case of imminent iron overload as described above.

6.7 Preparation and labelling of Investigational Medicinal Product

The investigational product will be produced by Vifor Pharma.

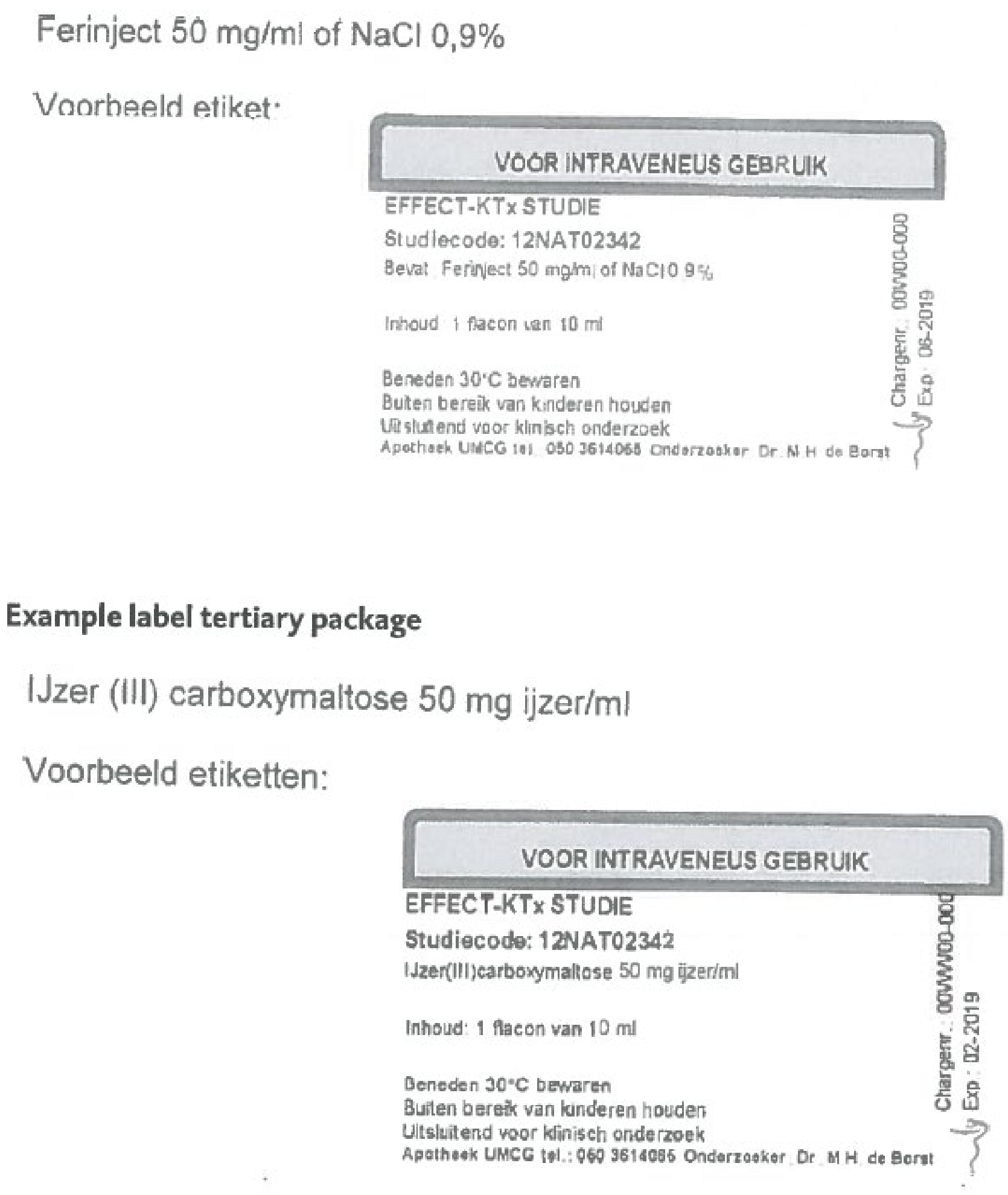

Figure 7. *Example of labels for study medication.*

6.8 Drug accountability

The investigational product, ferric(III)carboxymaltose, will be supplied by the manufacturer (Vifor Pharma) and shipped to the pharmacy at the UMCG, where it will be labelled. From there, medication will be sent to the UMCU. From the pharmacies, the product will be disposed just before administration to the patient.

The placebo solution will be supplied by the hospital pharmacies. Both ferric(III)carboxymaltose and the placebo will be stored in storages rooms at room temperature (below 25 degrees). Study medication that is not used will be destroyed by each pharmacy.

7. NON-INVESTIGATIONAL PRODUCT

7.1 Name and description of non-investigational product(s)
Not applicable to the EFFECT-KTx study

7.2 Summary of findings from non-clinical studies
Not applicable to the EFFECT-KTx study

7.3 Summary of findings from clinical studies
Not applicable to the EFFECT-KTx study

7.4 Summary of known and potential risks and benefits
Not applicable to the EFFECT-KTx study

7.5 Description and justification of route of administration and dosage
Not applicable to the EFFECT-KTx study

7.6 Dosages, dosage modifications and method of administration
Not applicable to the EFFECT-KTx study

7.7 Preparation and labelling of Non Investigational Medicinal Product
Not applicable to the EFFECT-KTx study

7.8 Drug accountability
Not applicable to the EFFECT-KTx study

8 Methods

8.1 Study Parameters/end points

i. Main study parameter/endpoint

The main study endpoint of the EFFECT-KTx study is exercise tolerance, quantified by the six-minute walk test (6MWT), after four dosages of ferric(III)carboxymaltose compared to placebo.

ii. Secondary study parameters/endpoints

Secondary study endpoints of the EFFECT-KTx study are:

- Haematologic and iron parameters: haemoglobin, haematocrit, mean corpuscular volume (MCV), free serum iron, serum ferritin, serum transferrin, serum hepcidin and C-reactive protein
- Quality of life, assessed by the Short-Form-36 (SF-36) questionnaire, the 5-Dimensial EuroQol (EQ-5D-5L) instrument, the Checklist Individual Strength (CIS) and the Dutch Multifactor Fatigue Scale (DMFS)
- Systolic and diastolic cardiac function analysed with a transthoracic echocardiography measuring left ventricular ejection fraction (LVEF), diastolic function and strain; serum NTproBNP and high-sensitive troponin-T
- Muscle strength, measured by the ‘Five-Times-Sit-to-Stand-test (FTSTS), the Timed-up-and-Go test (TUG) and a handgrip dynamometry
- Parameters of phosphate and bone metabolism:
    - Serum: phosphate, calcium, vitamin D, vitamin D3, parathyroid hormone, total FGF23 and intact FGF23
    - 24-hour urine: phosphate, calcium
- Intestinal microbiota
- Occurrence of clinical events: infections, hospitalizations, cardiovascular events, graft rejection and mortality
- Characteristics of the immune system: peripheral blood mononuclear cell (PBMC) count and cytokine and antibody expression.
- SARS-CoV2 vaccine efficacy
- Cognitive performance, measured by: the Dutch version of the National Adult Reading Test, the Digit Span which is a component of the Wechsler Adult Intelligence Scale, a Dutch version of the Rey Auditory Verbal Learning Test, the Cognitive Screening Test, the Trail Making Test, the Clock Drawing Test, the Word Fluency subtest of the Groninger Intelligentietest, the Controlled Oral Word Association Test and the Symbol Digit Modalities Test

- Other outcomes:
    - Parameters of kidney function and injury:
        - Blood: serum creatinine
        - Urine: MCD1, CD163, CD25, FOXP3 mRNA, kidney injury marker 1 (KIM-1)
    - Blood Pressure
    - Hepatic parameters: serum aspartate aminotransferase and alanine aminotransferase

iii. Baseline information

In addition to the above-mentioned parameters, we will collect some patient data at baseline, including:

- Medical history
- Diet (three day food diary)
- Demographic data
- Current medication
- Previous use of iron
- Previous blood donations
- Allergies
- Alcohol intake, smoking habits and drug use
- Blood pressure
- Body mass index, length and weight
- Signs of fluid retention

8.2 Randomisation, blinding and treatment allocation

The EFFECT-KTx trial will be a double blinded randomized controlled trial, for which the pharmacy of the UMCG will perform randomisation.
The pharmacy will compose a list of usable participant numbers grouped in blocks for the purpose of treatment center stratification. Per block, a simple randomisation will be executed, to ensure that the participants in a certain block will equally be divided between the two treatment arms in a random order.

The participant numbers will be distributed per group to one of both pharmacies (of the UMCG and UMCU). Once a new participant is included, either the pharmacy of the UMCG or the pharmacy of the UMCU will assign the first number on the list to this participant. Consequently, the employees of the pharmacy are not blinded to treatment allocation.
The transplantation nephrologist treating the patient, the patient and all investigators except one nurse or specially trained doctor's assistant will be blinded for treatment allocation and for iron parameters during the study. Iron-related blood sample results collected after baseline will not be shown in the regular file but in a locked document.
In both centres, the research nurse or doctor's assistant in charge of administration of the study medication will not be blinded. In case of acute adverse reactions requiring immediate medical treatment, this nurse or doctor's assistant or the pharmacy can unblind the patient and the doctor. This nurse or doctor's assistant will have access to lab data (iron status, phosphate levels) of each patient and will inform the pharmacy in case of imminent iron overload or severe hypophosphatemia, so a placebo can be administered instead (or, in the case of hypophosphatemia: no medication at all). The unblinded research nurse or doctor's

assistant will not inform the patient or other investigators about the treatment arm of a specific participant. Also, he or she will not be involved in collection of patient data or testing. Study medication will be packaged in a sealed cardboard box in order to make the package of the investigational product indistinguishable from the placebo. Before preparation, the flask inside the bottle containing the product will not be blinded. The unblinded nurse or doctor's assistant will unpack the medication in a closed room right before administration and add it to a bag of 240 mL 0.9% sodiumchloride solution. As described in paragraph 6.1, the bag will be covered with aluminium foil and black or white infusion lines will be used to prevent unblinding of the participants.

After the study, when the last participants have finished their treatment phase, all persons involved including the participant will be unblinded.

In case these tasks are performed by a doctor's assistant, he/she will be thoroughly trained by a qualified nurse and his/her competence will be examined.

8.3 Study Procedures

Type of test or treatment	Pre-baseline screening visit	T=0 (baseline)	T=6 weeks	T=12 weeks	T=18 weeks	T=24 weeks (or: check-out visit)
Medical history	15 minutes	15-30 minutes	10 minutes	10 minutes	10 minutes	15-30 minutes
Physical examination		15 minutes	10 minutes	10 minutes	10 minutes	15 minutes
6-minute walk test and muscle strength assessment	-	20-30 minutes	-	-	-	20-30 minutes
Blood sampling (including samples for clinical care, see Table 3)	20 minutes, 8 samples, 57.5 mL	20 minutes 7 samples, 40.5 mL	20 minutes 7 samples, 35 mL	20 minutes 8 samples, 45 mL	20 minutes 7 samples, 35 mL	20 minutes 13 samples, 90 mL
24-hour urine	-	At home	At home	At home	At home	At home
Faeces collection	-	At home	-	-	-	At home
Transthoracic Echocardiography	-	30 minutes	-	-	-	30 minutes
Quality of life Questionnaires	-	20 minutes	-	-	-	20 minutes
Food diary		At home (10-20 minutes)				At home (10-20 minutes)

Neuropsychological assessment (UMCG only)	-	45-60 minutes	-	-	-	45-60 minutes
FCM or placebo infusion	-	60 minutes Burden: Intravenous needle placement and possible side effects	60 minutes Burden: Intravenous needle placement and possible side effects	60 minutes Burden: Intravenous needle placement and possible side effects	60 minutes Burden: Intravenous needle placement and possible side effects	-
Total Burden	0.5 hours	4-5 hours	1.5-2 hours	1.5-2 hours	1.5-2 hours	3-4 hours

Table 2. *Tests and treatment per study visit, including an estimation of duration and burden. Participants will visit the outpatient clinic six times during the study: for a pre-baseline screening visit, an extensive baseline visit including the first dosage of medication, three visits at six, twelve and 18 weeks after baseline and for a checkout-visit at 24 weeks after baseline. In table 2, the procedures for each visit are summarized.*

Pre-baseline screening visit

During the pre-baseline screening visit, comprising blood collection and a meeting with the researcher, he or she will re-evaluate whether the patient meets all criteria and the patient will be asked for consent.

Medical history

During all visits, participants will be seen by a research physician or nurse. At the baseline visit, an extensive medical history will be taken. Participants will be interviewed about possible side effects of the study medication and about recent clinical events at the subsequent visits.

Physical examination and assessment of muscle strength and endurance

Participants will undergo physical examination at all study visits, including blood pressure measurements. Blood pressure is measured three times in succession with a Dinamap blood pressure meter.

Anthropometric measurements, including body weight and length, will be assessed at baseline and during the check-out visit while the patients wears light weighing clothes and no shoes.

At both the baseline visit and the check-out-visit, exercise capacity will be measured by a six-minute walk test (6MWT)and participants will also undergo the ‘Five-Times-Sit-to-Stand-test (FTSTS), the Timed-up-and-Go test (TUG) and a handgrip dynamometry. As all of these tests are also part of the Transplant Lines Cohort Study Protocol, patients who are also participant in this study will not have to perform them again for our trial.

The six-minute walk test is a well-known clinical exercise test. It requires a 30-meter, quiet walking course with marks every three meters and a flat, hard surface, but no other equipment. The 6MWT measures the distance a patient can walk as quickly as possible in six minutes of time, by letting the participant walk back and forth on the walking course. By using this test, cardiopulmonary fitness and muscle metabolism can be assessed. Patients do not reach their maximal exercise capacity during this test because they are allowed to choose their own intensity and are allowed to rest during the test. Also, they are permitted to use their usual walking aids such as a cane or a walker. Therefore, it accurately reflects functional exercise level for daily activities.[81]
Participants will be asked to wear comfortable shoes and clothes and not to exercise vigorously within two hours of beginning the test. Before the test, they should sit in a chair near the starting position for at least 10 minutes.

Handgrip strength will be assessed with the Jamar hydraulic hand dynamometer (Patterson Medical JAMAR 5030J1, Warrenville, Canada, Figure 8). The maximal handgrip strength reflects the muscle function and the total body muscle mass.[81,82] The test should be performed in the following way: the participant will be asked to sit in a chair and rest his dominant arm with the elbow on a table with the shoulder in adduction and should squeeze the device with force to perform a maximal isometric contraction. Handgrip strength will be tested three times with an interval of 30 seconds rest for recovery between each attempt. The second handle position of the hand dynamometer will be used which has been shown to be the most accurate position.[83]

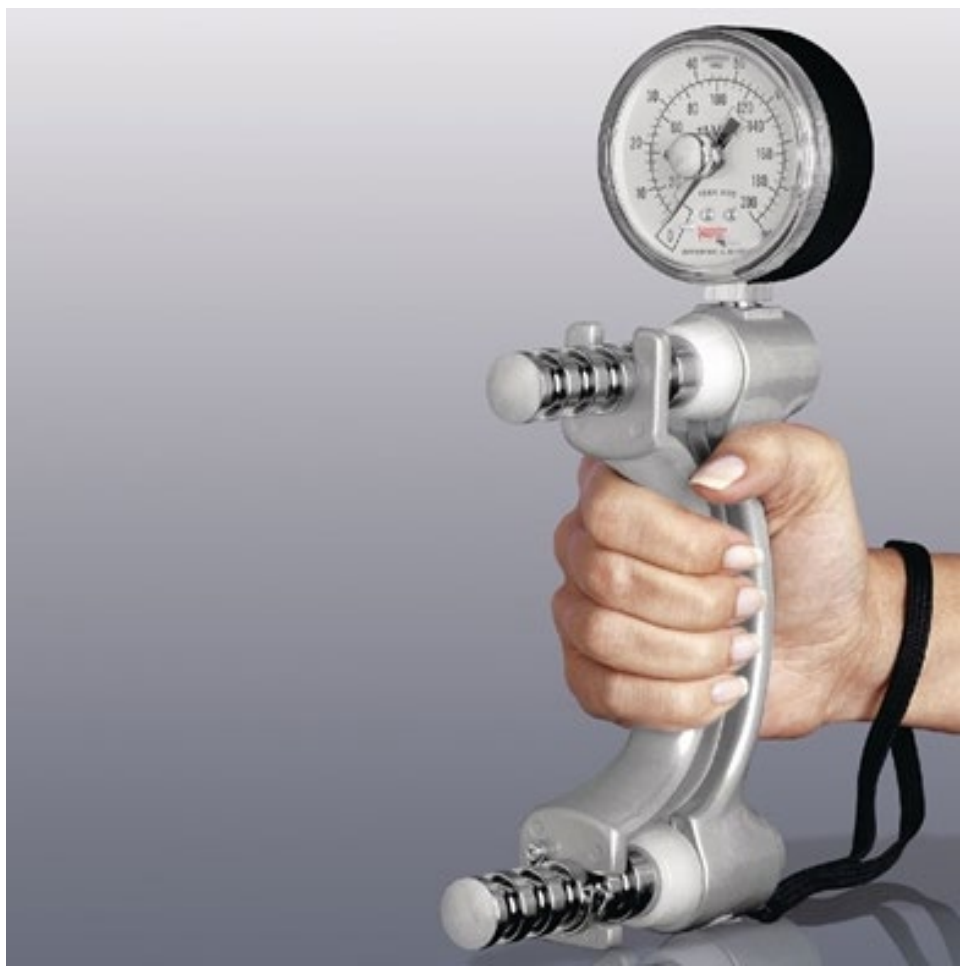

Figure 8. *Patterson Medical JAMAR. Source: www.devinemedical.com*[84]

Functional mobility will be tested using the FTSTS and the TUG.[81,82] The five times stand to sit (FTSTS) is a test measuring leg strength or the force-generating capacity of muscle by using the participants' body weight as resistance. Participants will be asked to stand up from their chair five times as fast as possible. The time required to do this is recorded in seconds. The task will be repeated five times.

The Timed up and go test (TUG) is a basic test based on strength, coordination and balance.[81,82,85,86] A pylon and a chair will be put three meters apart and participants will be instructed to stand up from the chair without support of their arms, then walk with their usual gait speed around the pylon and go back to the chair and sit down again. The test will be performed four times, with the first round being a trial. The test may be performed with the participants' usual walking aids. The TUG is measured in seconds.

Questionnaires

To quantify the quality of life, three measuring instruments will be done at baseline and check-out visits.

The Short-Form-36 (SF36) questionnaire is a frequently used questionnaire to assess health-related quality of life.[82,87] It has multiple domains: physical functioning, social functioning, physical and emotional limitations, mental health, energy, pain and overall perception of health. The questionnaire comprises 36 items and can be filled out by the patient in the waiting room.

The Dutch 'Checklist Individuele Spankrant' (CIS) questionnaire will be used to assess fatigue. It contains four domains (subjective fatigue, concentration, motivation and physical activity) and a total of 20 items.

The Dutch Multifactor Fatigue Scale (DMFS) is another questionnaire to assess fatigue and it comprises 38 items.

The EuroQol-5D-5L (EQ5D-5L) is another tool and consists of a questionnaire and a visual analogue scale.[82,88,89] It contains six domains: mobility, self-care, usual activities, pain/discomfort, anxiety/depression and cognition.

At baseline and at check-out visits, participants will also be asked to fill in a food diary during three subsequent days.

Blood sampling

At the pre-baseline screening visit and all five study visits, blood samples will be taken, requiring a venipuncture. For the participants who are in their first year after transplantation and who will have their baseline study visit at six months after transplantation, all blood collections will coincide with regular blood sampling. However, in all cases some extra tubes of blood will be taken during the study visit. In total, 303 mL of blood will have to be taken during the study, including the 101 mL of blood samples that have to be collected for clinical care (Table 3).

Other samples will be stored in a biobank at temperatures of -80C for 15 years, so additional analyses can be performed in the future.

At each visit, phosphate, ferritin and TSAT levels will be analyzed with priority.

Most laboratory tests in the study are part of a daily routine of participating study centers. Peripheral blood mononuclear cells (PBMCs) of participants in the UMCG will be isolated in the laboratories of transplant immunology . In the UMCU, due to logistic reasons, PBMCs will not be isolated and studied. Lymphocyte growth and function will be analyzed as well. For detection of cytokines, ELISA techniques will be used, in combination with fluorescence-activated cell sorting (FACS) of PBMC's.
Furthermore, total (Intact and cleavaged C-terminal) and intact FGF23 will be quantified using ELISA (Quidel, formerly Immutopics) in our research laboratory.

Collection of urine and faeces
At all study visits, participants will be asked to bring a 24-hour urine sample, which we will use to quantify phosphate and calcium excretion and store in a biobank. In a later study phase, we aim to analyze the urine for markers of kidney injury. Participants will receive special urine containers. They will be instructed to discard their first morning urine on the day before the visit and collect all subsequent urine throughout the next 24 hours including the first morning urine of the day of the visit.

Participants will be asked to collect and bring a faeces sample to the baseline and checkout visits. A FecesCatcher (TAG Hemi VOF, Zeijden, the Netherlands) will be given to them. After collection the participant should transfer the faeces to specific tubes and store them in the freezer. In the hospital, faeces samples will be stored in a biobank at -80C. The samples will be used for microbiota analyses. Since faeces samples are also collected for the Transplant Lines Cohort Study, for most patients this will not be an extra burden.

Blood, urine and faeces samples per study visit.

Same-day analyses (In black: for clinical and research purposes. In blue: for research only)

	Screening	Baseline	T=6	T=12	T=18	T=24
**EDTA/sysmex 4mL**						
Hemoglobin	X	X	x	X	x	X
Hematocrite		X	X	X	X	X
MCV	X	X	X	X	X	X
Trombocyte count		X	X	X	X	X
Leucocyte count	x	x	x	x	X	x
Machine (automatic) diff	x					x
**EDTA for pharmacy 4mL**						
Tacro or ciclo trough level		x	x	x	x	x
Cellcept trough level		x				x
**EDTA 4mL**						
PTH		x	X	x	x	x
**Clot activator tube 5mL**						
IgA, IgG, IgM	x					x
**Lithium heparin 4.5 mL**						
Sodium	X	X	x	X	x	X
Potassium		X	X	X	X	X

Kreatinin		X	X	X	X	X
ASAT		X	X	X	X	X
ALAT		X	X	X	X	X
AF		X	X	X	X	X
GGT		X	X	X	X	X
LDH		X	X	X	X	X
Phosphate	X	X	X	X	X	X
Calcium		X	X	X	X	X
Albumin		X	X	X	X	X
CRP		x	X	x	X	x
Ferritin	x	X				
Iron	X	x				
Transferrin	x	x				
Troponin-T		X		x		X
NTproBNP		x		X		X
**Lithium heparin 4.5 mL (-> dummy file)**						
Iron			X	x	X	x
Ferritin			X	X	X	X
Transferrin			x	X	x	X

Urine (full day collection)	Screening	Baseline	T=6	T=12	T=18	T=24
Total volume		x	x	x	x	X
Phosphate		x	X	x	X	X
Kreatinin		X	x	X	x	X
Calcium		x	x	X	x	x

Storage at -80 *C (for research only)

	Screening	Baseline	T=6	T=12	T=18	T=24	Analyses	Pipetting
Li-hep 10mL (UMCG only)	X					x	PBMCs (immunology)	Whole tube
Li-hep 10mL (UMCG only)	X					x	PBMCs (immunology)	Whole tube
Li-hep 10mL (UMCG only)	x					x	PBMCs (immunology)	Whole tube
Li-hep 4.0mL		x		x		x	Soluble transferrin receptor (0.5 mL)	4 x 0.5 mL
EDTA 10 mL		x		X		x	iFGF23 (2x0.3 mL)<br><br>cFGF23 (2x0.3 mL)<br><br>Calcidiol (0.75 mL)<br><br>Complement (0.5 mL)	5-7 x 0.5 mL<br>1 x 0.8 mL plasma
EDTA 4 mL			x		x		iFGF23 (2x0.3 mL)	2 x 0.6 mL<br>1 x 0.5 mL plasma

							cFGF23 (2x0.3 mL)<br><br>Complement (0.5 mL)	
EDTA 4 mL	x						Immunology<br><br>Complement (at screening only)	1 x 1 mL<br>1-2 x 0.5 mL plasma
Clot activator 10 mL		x	X (only hepcidin)	x	X (only hepcidin)	x	Calcitriol (not at t=6 and t=18)<br><br>Hepcidine	2 x 0.6 mL<br>2 x 0.5 mL (in PP tube)<br>1-2 x 0.5 mL (extra)
Clot activator 10mL	x					x	Cytokines (IL-2, IL-6, IL-8, IL-10, IL12, IL-15, TNFa)<br><br>Donor-specific antibodies	7 x 0.5 mL (2 PP tubes)
Urine		x	x	x	x	X	MCD1, CD163 (macrophage marker), CD25 (T cell marker), kidney injury marker 1 (KIM-1)	4 x 2 mL supernatant
Faeces		x				x	Microbiota	1 sample

Total blood sampling per visit

	Screening	Baseline	T=6	T=12	T=18	T=24	Total
**Clinical care and reseach purposes**	13.5 mL	16.5 mL	16.5 mL	16.5 mL	16.5 mL	21.5 mL	101 mL
**Research only**	44 mL	24 mL	18.5 mL	28.5 mL	18.5 mL	68.5 mL	202 mL
**Total**	57.5 mL	40.5 mL	35 mL	45 mL	35 mL	90 mL	303 mL

Table 3. *Blood, urine and faeces sampling per study visit. PBMC = Peripheral Blood Mononuclear Cells; CNI = calcineurin inhibitor; PTH = parathyroid hormone.*

	**Before first SARS CoV2-vaccination ***	**Before second SARS CoV2-vaccination***	**28 days after second vaccination***	**6 months after second vaccination ***	**12 months after second vaccination***

**Capillary blood from finger prick (3 drops, 0,15 mL)** SARS-CoV2 specific IgG	x	x		x	x
**Clot activator tube 5mL** SARS-CoV2 specific IgG			X		
**Li-hep 10mL** PBMC Isolation			X		
**Li-hep 10mL** PBMC Isolation			x		

Table 4. *Capillary and venous sampling per study visit of the the COVAC-EFFECT substudy (40-50 participants, UMCG only). PBMC = Peripheral Blood Mononuclear Cells.*
**Vaccinations are not part of the study protocol but performed in the context of the Dutch national vaccination campaign*

Transthoracic echocardiography

A cardiac ultrasound will be performed at baseline and checkout visits to evaluate systolic and diastolic function. Flow and pressure in the atria and ventricles will be assessed, wall thickness and the left ventricular ejection fraction will be measured on the spot and images will be analysed at a later stage for strain.
A Vivid E 90 cardiovascular ultrasound system of the company GE Healthcare will be used. The transthoracic echography will take 30 minutes on average.

Neuropsychological testing

Participants in the UMCG will undergo multiple neuropsychological tests to assess cognitive performance at baseline and after the intervention. The tests are performed by neuropsychology students and will take around 60 minutes in total. Due to logistic reasons, neuropsychological testing will not be performed in participants of the UMCU.

The Cognitive Screening Test (CST) is a Dutch orientation questionnaire to screen for cognitive impairment.[90] The questionnaire is an adaptation of the Short Portable Mental Status Questionnaire and consists of 20 items. It will be taken at baseline only.

The Clock Drawing Test (CDT) is another cognitive screening instrument that will only be taken at baseline.[91] Participants are asked to draw a clock and set the time to ‘a quarter to two’.

The Nederlandse Leestest voor Volwassenen (NLV) is a dutch version of the National Adult Reading Test. It provides an estimation of the intelligence quotient. The participant has to read out a list of 50 irregularly spelled words.[92] This test will only be taken at baseline.

The Trail Making Test (TMT), consists of two parts. Part A is a tool to measure attention and information processing speed. This task involves connecting 25 numbers in an ascending order, as quickly as possible. Part B concerns divided attention and cognitive flexibility. In this condition, numbers as well as letters have to be connected in an alternating ascending order (1-A-2-B- etc.). Both parts of the test are timed to completion.[81]

The Digit Span is a subtest of the Wechsler Adult Intelligence Scale (WAIS-IV) and consists of two tasks: the Digit Span Forward and the Digit Span Backward.[93] The Digit Span Forward measures auditory memory span. In this task, the participant is asked to repeat a series of numbers in the same order as the examiner did. The Digit Span Backward measures working memory. The participant has to repeat the same numbers but in reversed order.

The 15 Woorden Test, the dutch version of the Rey Auditory Verbal Learning Test (RAVLT), assesses verbal memory.[94] In this task five sets of 15 unrelated words are presented to the participant, who is asked to recall as many possible after each set (Immediate Recall). After 20 minutes, participants are asked to recall as many of the 15 words as possible (Delayed Recall). Additionally, a recognition task will be performed: participants are presented with a list of 30 words and are asked which words they recognize from the list they have been presented before.

The Word Fluency is a subtest of the Groninger Intelligentie Test (GIT-2), measuring semantic memory.[95] Participants are asked to name as many words within a two categories (animals and professions) within one minute.

The Controlled Oral Word Association Test (COWAT) is a verbal task measuring executive control.[96] Participants have to name as many words as possible that start with a specific letter within one minute.

The Symbol Digit Modalities Test (SDMT) quantifies psychomotor speed.[97] The test consists of matching symbols and numbers as fast as possible in 90 seconds.

Incidental medical findings

In case of any incidental findings that could have clinical relevance to the patient, the investigators will contact the patients' nephrologist or general practitioner.

Administration of study medication

At the baseline visit and after six, twelve and 18 weeks, study medication will be intravenously administered. An intravenous needle will be placed and the patient will receive either the placebo or the ferric(III)carboxymaltose solution. The volume of both solutions will be 250 mL and administration will take 15 minutes, followed by at least 30 minutes of observation. After the administration, the needle will be removed.

8.4 Withdrawal of individual subjects

Participants can leave the study at any time and for any reason if they wish to do so without any consequences. They will however receive an invitation for a check-out visit if they received at least one dose of study medication. Participants who leave the study early will be included in the analyses according to the intention-to-treat principle.
The investigator can decide to withdraw a subject from the study for urgent medical reasons and serious events that may be related to procedures applied during the trial.

The following events are defined criteria for withdrawal from receiving study medication:

- Allergic reactions including rash
- Remarkable elevations of the transaminases
- Pregnancy

As previously explained, ferritin and TSAT levels at t=6, t=12 and t=18 weeks will be accessible only for a unblinded research nurse or doctor’s assistant. In the event of imminent iron overload, participants will not be withdrawn from the study but will receive placebo instead of ferric(III)carboxymaltose. In case of intermediate or severe hypophosphatemia, defined by a phosphate level of 0.50 mmol/L or lower, the participant will not receive any study medication until the phosphate levels are again above this level. Also in case of an infection or severe hypertension (RR >160/100 mmHg), the patient will receive no study medication.

8.5 Replacement of individual subjects after withdrawal

Our sample size contains a 10% surplus of participants to account for withdrawal. We will not include additional subjects in case of withdrawal of a participant.

8.6 Follow-up of subjects withdrawn from treatment

Participants withdrawn from treatment will be invited for a checkout visit if they received at least one dose of study medication. The check-out visit comprises the same procedures as the last study visit described in this protocol.

8.7 Premature termination of the study

The early termination of the EFFECT-KTx trial will be decided after thorough discussion among members of the research teams of both centers (UMCG: dr. M.H. de Borst, prof. dr. S.J.L. Bakker, dr. P. van der Meer, drs. J.S.J. Vinke; UMCU: dr. A.D. van Zuilen). In case of premature termination of the study, the Medical Ethical Board (METC) will be informed within 15 days.

9 SAFETY REPORTING

9.1 Temporary halt for reasons of subject safety

In accordance to section 10, subsection 4, of the Medical Research Involving Human Subjects ACT (WMO), the sponsor will suspend the study if there is sufficient ground that continuation of the study will jeopardise subject health or safety.[98] The sponsor will notify the accredited METC without undue delay of a temporary halt including the reason for such an

action. The study will be suspended pending a further positive decision by the accredited METC. The investigator will take care that all subjects are kept informed.

9.2 AEs, SAEs and SUSARs

9.2.1 Adverse events (AEs)

Adverse events are defined as any undesirable experience occurring to a subject during the study, whether or not considered related to the investigational product or trial procedure. All adverse events reported spontaneously by the subject or observed by the investigator or his staff will be recorded.

9.2.2 Serious adverse events (SAEs)

A serious adverse event is any untoward medical occurrence or effect that

- results in death;
- is life threatening (at the time of the event);
- requires hospitalisation or prolongation of existing inpatients' hospitalisation;
- results in persistent or significant disability or incapacity;
- is a congenital anomaly or birth defect; or
- any other important medical event that did not result in any of the outcomes listed above due to medical or surgical intervention but could have been based upon appropriate judgement by the investigator.

An elective hospital admission will not be considered as a serious adverse event.

In accordance to section 13p of the WMO, all SAE's will be reported to the METC that approved the protocol through the website toetsingonline.nl, without delay and within 15 days after the sponsor has first knowledge of the SAE.[98,99] In case of death or a life-threatening SAE, the sponsor will inform the METC within seven days of first knowledge of the SAE, followed by a maximal period of eight days to complete the initial preliminary report. There are no predefined exceptions to this.

9.2.3 Suspected unexpected serious adverse reactions (SUSARs)

Adverse reactions are all untoward and unintended responses to an investigational product related to any dose administered.

Unexpected adverse reactions are SUSARs if the following three conditions are met:

1. the event must be serious (see chapter 9.2.2);

2. there must be a certain degree of probability that the event is a harmful and an undesirable reaction to the medicinal product under investigation, regardless of the administered dose;

3. the adverse reaction must be unexpected, that is to say, the nature and severity of the adverse reaction are not in agreement with the product information as recorded in:

- Summary of Product Characteristics (SPC) for an authorised medicinal product;

- Investigator's Brochure for an unauthorised medicinal product.

The sponsor will urgently report the following SUSARs through the web portal ToetsingOnline to the METC:[99]

- SUSARs that have arisen in the clinical trial that was assessed by the METC;
- SUSARs that have arisen in other clinical trials of the same sponsor and with the same medicinal product, and that could have consequences for the safety of the subjects involved in the clinical trial that was assessed by the METC.

The remaining SUSARs are recorded in an overview list (line-listing) that will be submitted once every half year to the METC. This line-listing provides an overview of all SUSARs from the study medicine, accompanied by a brief report highlighting the main points of concern. The expedited reporting of SUSARs through the web portal Eudravigilance or ToetsingOnline is sufficient as notification to the competent authority.[99,100]

The sponsor will urgently report all SUSARs to the competent authorities in other Member States, according to the requirements of the Member States.

The expedited reporting will occur not later than 15 days after the sponsor has first knowledge of the adverse reactions. For fatal or life threatening cases the term will be maximal 7 days for a preliminary report with another 8 days for completion of the report.

In the UMCU, the local investigator has the responsibility to inform the study sponsor about any SAE as soon as possible and to monitor the timely reporting to the METC.

In case of a SAE, the subject's treatment arm will be revealed by the pharmacy. A copy of the randomisation code, readily accessible in emergency situations, will be available in the pharmacy during out of office hours.
If the SAE occurs in a participant of the UMCU, the pharmacy in the UMCG will have to be consulted for breaking of the code.

9.3 Annual safety report

In addition to the expedited reporting of SUSARs, the sponsor will submit, once a year throughout the clinical trial, a safety report to the accredited METC, competent authority, and competent authorities of the concerned Member States. This is in accordance with the WMO, section 13q.[98]

This safety report consists of:

- A short literature review summarizing relevant new studies concerning (intravenous) iron supplementation, published since the writing of the original protocol or since the last version of the annual safety report;
- a list of all suspected (unexpected or expected) serious adverse reactions, along with an aggregated summary table of all reported serious adverse reactions, ordered by organ system, per study;

- a report concerning the safety of the subjects, consisting of a complete safety analysis and an evaluation of the balance between the efficacy and the harmfulness of the medicine under investigation.

9.4 Follow-up of adverse events

All AEs will be followed until they have abated, or until a stable situation has been reached. Depending on the event, follow up may require additional tests or medical procedures as indicated, and/or referral to the general physician or a medical specialist.
SAEs need to be reported till end of study within the Netherlands, as defined in the protocol.

9.5 Data Safety Monitoring Board (DSMB)

Because we consider the trial to be low-risk study, a Data Safety Monitoring Board (DSMB) will be not established.

10 STATISTICAL ANALYSIS

This study will be a double blinded randomized controlled trial. For the randomisation plan of the EFFECT-KTx trial we refer to paragraph 8.2.

In this chapter, a statistical analysis plan (SAP) is described. The plan may be adapted according to new insights and details may be added. However, after the randomisation code is broken, the SAP can no longer be changed.

We will analyse all results according to the intention to treat principle and include all subjects who received at least one study drug administration in the ferric(III)carboxymaltose group and who had at least one efficacy assessment after baseline. Differences are considered statistically significant at P<0.05.

We will determine the normal distribution of the ratio variables according to the following criteria:
- Sample size <30 cases: the variable is not considered as normally distributed.
- Sample size 30-50: the variable is considered as normally distributed on the condition that both the Kolmogorov-Smirnov test and the Shapiro-Wilk test have a probability value of >0.05.
- Sample size of >50: the variable is considered as normally distributed on the condition that both the Normal Q-Q-plot and the Detrended Normal Q-Q-plot suggest this.

In the case of an a non-normal distribution, we will try transforming the data before we decide to use non-parametric tests for analyses.

We will determine the homogeneity of variance of the ratio variables using Levene's test.

Statistical analyses will be performed with IBM SPSS software.

Missing data will primarily not be replaced by estimates. However, in the case of a high percentage of missing data, additional worst-case imputation analyses will be performed for all concerning parameters.

10.1 Primary study parameter

The main study endpoint of the EFFECT-KTx study is exercise tolerance, quantified by the six-minute walk test (6MWT), after treatment with ferric(III)carboxymaltose, compared to placebo. The 6MWT will be performed before the first dose (at t=0) and six weeks after the last dose (at t=24 or at the premature check-out visit).
The aimed sample size of 158 participants is based on a power of 0.9 and an expected dropout ratio of 10%, The calculation is described in detail in paragraph 4.4.

The difference between the 6MWT score at baseline and after the last dosage of study medication will be analyzed with the Paired Samples t-Test. In case the distribution is not considered normal and the variable cannot be transformed to a normally distributed variable the Wilcoxon Matched Pairs Test will be used
The difference between the mean increase or decrease in 6MWT score between the placebo group and the intervention group will be analyzed with the Independent Samples t-Test. In case the distribution is not considered normal and the variable cannot be transformed to a normally distributed variable the Mann Whitney Test will be used. Also, we will perform a mixed model analysis.

Multivariable analysis will be performed to correct for age, gender, glomerular filtration rate, haemoglobin level, serum ferritin, transferrin saturation, glomerular filtration rate and left ventricular ejection fraction. However, the result of the univariate analysis will be our primary result.

10.2 Secondary study parameter(s)

To analyse the effect of iron supplementation on all secondary parameters, with the exception of hepatic parameters, the gut microbiota and the incidence of clinical events, we will use the following techniques:
- To evaluate the difference between outcomes at t=0 and at t=6, t=12, t=18 and t=24 months within a study arm, the Paired Samples t-Test will be used (or in case the distribution is not considered normal and the variable cannot be transformed to a normally distributed variable: the Wilcoxon Matched Pairs Test). Also, we will perform mixed models analyses.
- To evaluate the difference between the placebo and the intervention groups in mean differences between baseline and any other study visit, we will use the Independent Samples t-Test (or in case the distribution is not considered normal and the variable cannot be transformed to a normally distributed variable: the Mann Whitney Test).
- To evaluate the correlation between the mean increase or decrease of the outcome between baseline and any other study visit and the (increase in) haematologic parameters, we will perform linear regression analysis techniques and we will assess the Pearson correlation coefficient (or in case the distribution is not considered normal and the variable cannot be transformed to a normally distributed variable: the Spearman's rank correlation coefficient).
- To evaluate differences in incidence of elevated transaminases, hypophosphatemia, mild side effects, infectious diseases, cardiovascular events, graft rejection and mortality between the placebo and the iron group, we will use survival analysis.

We will perform multiple variable analyses, for example to evaluate:
- The effect of (increase in) iron parameters on the 6MWT and on quality of life, corrected for cardiac function, muscle function, kidney function and haemoglobin levels;
- The effect of (increase in) iron parameters on cardiac function, muscle function and cognitive function after correction for haemoglobin levels;

- The effect of (increase in) iron parameters on serum phosphate, corrected for vitamin D, PTH and FGF23 levels;
- The effect of (increase in) iron parameters on serum FGF23 levels, corrected for serum and urine phosphate, vitamin D and PTH levels;
- The effect of (increase in) iron parameters on the incidence of clinical events, corrected for glomerular filtration rate and haemoglobin levels.

10.3 Other study parameters

We will compare baseline characteristics between the placebo group and the intervention group using descriptive statistics and either the Independent Samples t-Test (or in case the distribution is not considered normal: the Mann Whitney Test) or the Chi Square Test.

10.4 Interim analysis

No predefined interim analysis will be performed.

11 ETHICAL CONSIDERATIONS

11.1 Regulation statement

The study will be conducted according to the principles of the Declaration of Helsinki (tenth version, October 2013) and in accordance with the Medical Research Involving Human Subjects ACT (WMO).[98,101] All investigators have taken notice of these documents.
The health of the participants will be our first consideration and we shall act in their best interest.

11.2 Recruitment and consent

Patients will be recruited at the outpatient clinic of the UMCG (local coordinator: Dr. M.H. de Borst) and the UMCU (local coordinator: Dr. A.D. van Zuilen).
When a patient meets the inclusion criteria, the patients' nephrologist will consider the possible adverse and beneficial effects of participating in the study on the health and wellbeing of his patient. If he or she expects the patient may be fit for participation, he or she will inform the patient about the study, hand him or her an information letter, including an informed consent form, and ask permission to the patient for him or her to receive a phone call from one of the investigators. In case the patient agrees to this, the junior investigator will call the patient after at least five working days and within a month, to ask the patient about any questions he or she may have about the study and to ensure he or she understood all information. If the patient agrees to informed consent, he or she will be invited for a pre-baseline screening visit. During this visit, the researcher will again evaluate whether the patient meets all criteria and the patient will be asked to sign the form. If the consent cannot be expressed in writing, the non-written consent must be formally documented and witnessed. After agreement to informed consent, the patient will be included in the study, randomized to treatment arm and a baseline visit will be planned. Patients in nearby community hospitals may also be proposed by their nephrologist.

According to the Declaration of Helsinki, section 26, and the WMO, section 6, subsection 5 and 6, the information letter will provide the potential participant with adequate information on the aims, methods, funding resources, possible conflicts of interest and the potential benefits and risks of the study.[98,101] It will give a clear description of expected durance and burden of each study visit for the participant. The subject will be informed about the right to refuse to participate in or to prematurely withdraw from the study.

The refusal of a patient to participate in the study or the patient's decision to withdraw from the study should never adversely affect the relation the patient had with his or her physician. It is the responsibility of the physician to emphasise this to the patient and if he suspects that the patient feels obliged to give informed consent, he must delegate the recruitment process to an independent colleague.

11.3 Objection by minors or incapacitated subjects

Since minors and incapacitated subjects will not be included in the study, this paragraph is not applicable to the EFFECT-KTx trial.

11.4 Benefits and risks assessment, group relatedness

As most medical studies involving humans, the EFFECT-KTx involve some risks and burdens to the patient. However, we believe that these disadvantages of participation are in proportion to the value of the research and the potential benefit to the subject.

As described in chapter 8, the participant will have to visit the outpatient clinic five times for study purposes. In most cases, these visits will coincide with regular hospital visits. Both the baseline visit and the check-out visit will take approximately four hours, all other visits will take around two hours. During the visits, participants will have to fill in multiple questionnaires and complete some neuropsychological tests. Together with the cardiac ultrasound, the 6MWT and the muscle strength assessment, this may be an energy-consuming procedure for them.

Before every study visit, participants will be asked to collect a 24-hour urine sample. Also they will collect a faeces sample twice, which has to be stored in the freezer at home. Patients might experience this as a messy inconvenience, especially if they are at work during the urine collection.

At all study visits, the participant will have to be subjected to a venepuncture. For patients in their first year after transplantation, these venepunctures will coincide with the ones performed for clinical purposes. Each study visit, 65 mL of blood will be taken, divided over nine tubes.

Finally, an intravenous needle will be placed and either ferric(III)carboxymaltose or a placebo solution will be administered. As explained in chapter 5 and 6, minor side effects that may occur are headache, dizziness, gastrointestinal symptoms such as nausea and a rash. Potential severe adverse effects are toxic and allergic reactions, which are very uncommon after ferric(III)carboxymaltose infusion. Hypophosphatemia and systemic infections are also adverse events that may occur.

To minimize the potential risk for participants, we will strictly adhere to the exclusion criteria described in chapter 4. Particularly patients with marked hypophosphatemia will not be included and participants who develop hypophosphatemia during the study will not receive any study medication until phosphate levels are adequate. Ferritin and transferrin will be monitored at every study visit and in case of imminent overload, no ferric(III)carboxymaltose will be administered. Relatively low dosages of ferric(III)carboxymaltose will be administered to prevent the incidence of toxic overload reactions.

11.5 Compensation for injury

According to the declaration of Helsinki, section 15 and to the WMO, section 7, appropriate compensation and treatment for subjects who are harmed as a result of participating in the study must be insured.[98,101]

The UMCG, as the sponsor of the study, has an insurance for test subjects which will apply to all subjects of the study. This insurance provides cover for damage to research subjects through injury or death caused by the study.

The insured amounts are:

1. € 650.000,- for death or injury for each subject who participates in the study;
2. € 5.000.000,- for death or injury for all subjects who participates in the study;
3. € 7.500.000,- for the total damage incurred by the organization for all damage disclosed by scientific research for the sponsor as 'verrichter' in the meaning of said Act in each year if insurance coverage.

The insurance applies to the damage that becomes apparent during the study or within 4 years after the end of the study.

The UMCG also has a liability insurance within the same insurance policy. This insurance will only apply to study participants at the UMCG. The UMCU has a liability insurance of its own to cover for the study participants at the UMCU.

11.6 Incentives (if applicable)

For all participants who have to come to the hospital for extra visits, a compensation for travel expenses and parking costs will be available.

11.7 Independent expert

According to the WMO, section 9, an independent expert, who is not involved in the study, is appointed. Participants can contact this independent expert for advise concerning the study.[98]

12 ADMINISTRATIVE ASPECTS, MONITORING AND PUBLICATION

12.1 Handling and storage of data and documents

A list of randomly assigned participant identifications codes, not containing meta-data, will be made so the data can be traced to an individual subject. The list will be guarded by the junior investigator. The sponsor (or the principal investigator), the local investigator in the UMCU and a study monitor will have access to the list. The study data and the code list will be kept for a period for 15 years, according to the Dutch law (Wet op de Geneeskundige Behandelingsovereenkomst, WGBO).[102]
Questionnaires, which contain no patient identification data other than the code, will be scanned and stored for 15 years. Samples will be contained in a Biobank and stored for 15 years as well.

All data will be collected in a raw data database developed by REDCap.

Participants will be asked for consent about the storage of their email address. If they agree, the quality of life questionnaires can be send by email.

12.2 Monitoring and Quality Assurance

An independent study monitor will be assigned by the sponsor. It is the responsibility of the independent monitor to monitor the informed consent of participants, the correctness of the data in the raw data database, the reporting of adverse events and the recruitment prodecure of participants according to the before-mentioned inclusion- and exclusion criteria.

According to the NFU guidelines, and based on our conception that the study has a low risk for participants, the monitor will have to visit both the UMCG and the UMCU once per year.[103] He will monitor the informed consent by verifying one to ten percent of informed consent forms. Also he will verify the inclusion and exclusion criteria of the first three participants of both centres and afterwards one to ten percent of them. Finally, the source data and the reporting of adverse events of one to ten percent of the participants will have to be verified.

12.3 Amendments

A ‘substantial amendment’ is defined as an amendment to the terms of the METC application, or to the protocol or any other supporting documentation, that is likely to affect to a significant degree:

- the safety or physical or mental integrity of the subjects of the trial;
- the scientific value of the trial;
- the conduct or management of the trial; or
- the quality or safety of any intervention used in the trial.

All substantial amendments will be notified to the METC and to the competent authority.

Non-substantial amendments will not be notified to the accredited METC and the competent authority, but will be recorded and filed by the sponsor.

12.4 Annual progress report

The sponsor/investigator will submit a summary of the progress of the trial to the accredited METC once a year. Information will be provided on the date of inclusion of the first subject, numbers of subjects included and numbers of subjects that have completed the trial, serious adverse events/ serious adverse reactions, other problems, and amendments.

12.5 Temporary halt and (prematurely) end of study report

The sponsor will notify the accredited METC and the competent authority of the end of the study within a period of 90 days. The end of the study is defined as the last patient's last visit.

The sponsor will notify the METC immediately of a temporary halt of the study, including the reason of such an action.

In case the study is ended prematurely, the sponsor will notify the accredited METC and the competent authority within 15 days, including the reasons for the premature termination.

Within one year after the end of the study, the investigator/sponsor will submit a final study report with the results of the study, including any publications/abstracts of the study, to the accredited METC and the Competent Authority.

12.6 Public disclosure and publication policy

In agreement with the CCMO publication Statement, the results of the study will be disclosed after statistical analyses have been performed.[104] Regardless of the outcomes, we aim to publish the results in a peer-reviewed scientific journal within reasonable time after finishing the study. Since this will be the first trial in the field of kidney transplantation, we expect to publish the outcomes in a high-impact journal. Some of the secondary outcomes will be published separately. Simultaneously with publication in scientific journals, we will present the data at international congresses. The results will also be included in a PhD thesis.

13 STRUCTURED RISK ANALYSIS

13.1 Potential issues of concern

The investigational product is ferric(III) carboxymaltose, which is a registered medicine for the indication of iron deficiency anaemia. Our study will evaluate the use of ferric(III)carboxymaltose for a broader indication: iron deficiency with or without anaemia, in kidney transplant recipients.

a. Level of knowledge about mechanism of action

Iron is an essential element of the human diet. In the Netherlands, the median daily intake is 8.1-12.1 mg for men and 7.8-10.1 mg for women, of which, under normal circumstances, only around ten percent is absorbed in the intestinal system.[105] Dietary iron can be divided between haem-iron (in red meat) and non-haem iron. Haem-iron is absorbed more efficiently than non-haem iron. Gastric acidity, low body iron stores and vitamin C promote iron absorption, whereas hepcidin and gastric acid inhibitors reduce iron absorption.[6]

Iron can circulate in plasma freely, with a normal level of 13-32 umol/L. However, a large proportion is bound to transferrin or stored as ferritin in hepatocytes, reticuloendothelial cells and skeletal muscle cells. On an average day, 20 mg of iron per day is incorporated in haemoglobin.[6] 0.5 to 1.0 mg of iron is excreted per day, in faeces, urine and perspiration. In women, during menstruation, an additional 0.5-0.7 mg of iron is lost every day.[6]

As described in chapter 1, iron is an important component of haemoglobin and myoglobin and plays a pivotal role in muscle function and oxygen transport through the body. Also, it is involved in various mitochondrial enzymatic reactions.

b. Previous exposure of human beings with the test product(s) and/or products with a similar biological mechanism

Ferric(III) carboxymaltose is the preferred intravenous iron solution for clinical use. Its effectivity has been studied in various populations (chapter 1 and 6.3).[72]
In the heart failure population, ferric(III) carboxymaltose was proved to effectively increase haematologic parameters in case of iron deficiency, and also had a positive effect on exercise tolerance.
The same benefits were observed in a comparative study performed with a population of patients with chronic kidney disease, where ferric(III)carboxymaltose was more effective than iron sucrose solution. Hemodialysis patients with iron deficiency had a significantly larger response to ferric(III)carboxymaltose in comparison with an oral iron product.

c. Can the primary or secondary mechanism be induced in animals and/or in *ex-vivo* human cell material?

ferric(III)carboxymaltose has been tested in animal studies, as is described in chapter 6.2. ferric(III) carboxymaltose does cross the placenta barrier and is also secreted in milk, so the safety in (early) pregnancy and during lactation cannot be guaranteed. In pre-clinical studies,

no allergenic, immunotoxic or carcinogenic effects were observed, and there were no indications of any adverse effect of ferric(III)carboxymaltose on fertility.[72]

d. Selectivity of the mechanism to target tissue in animals and/or human beings

Since iron is important for all human organs, this is not applicable to ferric(III)carboxymaltose.

e. Analysis of potential effect

An extensive description of possible adverse events and safety concerns can be found in chapter 6.4.
Reported side effects of ferric(III)carboxymaltose are nausea (3-7%), local skin reactions (1%), hypophosphatemia (2%), headache (1%), dizziness (2%) and transient hypertension (4-6%). Anaphylactic reactions are extremely rare in ferric(III)carboxymaltose, but may be severe.[72]
In high dosages, ferric(III)carboxymaltose could result in toxic iron overload reactions. However, in dosages of 500mg, no such reactions have been previously reported. Also, to prevent this from occurring, we will control ferritin and transferrin levels and (temporarily) discontinue treatment with ferric(III)carboxymaltose in case of imminent overload.
Hypophosphatemia after administration of ferric(III)carboxymaltose is a potential concern, especially in kidney transplant recipients. Although hypophosphatemia is rarely symptomatic, the long-term effect on the bones is unknown. Therefore, we will closely monitor phosphate levels, exclude patients with severe hypophosphatemia at baseline and administer only low dosages of 10 mL of ferric(III)carboxymaltose (50 mg Fe^{3+}/mL).
Theoretically, the risk of infections after iron supplementation are increased. However, there is little clinical evidence of this.

f. Pharmacokinetic considerations

The half-life elimination of ferric(III) carboxymaltose is seven to twelve hours and the time to peak is 15 to 70 minutes. The excretion is mainly with the urine.
91 to 99% percent of iron from ferric(III)carboxymaltose is incorporated in erythrocytes in case of iron deficiency, and 61 to 84% in anaemic kidney patients. Remaining iron is rapidly eliminated from the blood and transported to bone marrow, the spleen and the liver.[72]
In healthy subjects who received 100 to 1000 mg of ferric(III)carboxymaltose, serum iron levels reached up to 333 ug/mL after 15 to 70 minutes.[72]

g. Study population

The study population will consist of stable kidney transplant recipients suffering from iron deficiency. No pregnant women or children are included.

h. Interaction with other products

Intravenous iron supplementation might reduce the absorption of oral iron products.

i. Predictability of effect

The primary effect of ferric(III)carboxymaltose van be quantified by measuring serum ferritin and transferrin saturation. In case of iron deficiency anaemia, the hemoglobin level is also a valid biomarker for effect.

j. Can effects be managed?

As described in chapter 5, a medical doctor will be available during and after the administration of the study medicine, to treat any adverse reactions. Also, facilities for cardiopulmonary resuscitation and adrenalin solution to treat acute anaphylaxis will be available. After the administration of the study drug, participants will have to wait for departure until at least 30 minutes after treatment.
In case of an accidental overdose or a toxic reaction, serum ferritin and transferrin will be measured. Free serum iron is not a valid parameter to determine iron overdose after intravenous solution. Severe iron accumulation can be treated with deferoxamine.[106]

13.2 Synthesis

In summary, treatment of iron-deficient kidney transplant recipients with ferric(III)carboxymaltose contains some risks. Mild adverse effects may occur and severe reactions, which are extremely rare, cannot be excluded. The long-term effects of ferric(III)carboxymaltose -induced hypophosphatemia on bone density are unknown. However, ferric(III)carboxymaltose has been thoroughly studied and is frequently used in various populations. It has proved to be a safe and highly effective medicine to treat iron deficiency. We realise that participating in the trial may be a time-consuming and in some cases confronting and arduous process for patients.
Based on before-mentioned studies, we expect iron-deficient kidney transplant recipients to benefit from treatment with ferric(III)carboxymaltose as a result of an improvement in exercise tolerance, (cardiac) muscle function, cognitive function and general wellbeing.

14 REFERENCES

1. Registratie Nierfunctievervanging Nederland. www.renine.nl.
2. Zelle DM, Klaassen G, Van Adrichem E, Bakker SJL, Corpeleijn E, Navis G. Physical inactivity: A risk factor and target for intervention in renal care. *Nat Rev Nephrol*. 2017. doi:10.1038/nrneph.2016.187
3. Eisenga MF, Minović I, Berger SP, et al. Iron deficiency, anemia, and mortality in renal transplant recipients. *Transpl Int*. 2016. doi:10.1111/tri.12821
4. Renal Transplantation Database UMCG.
5. Jimeno L, Rodado R, Campos M, Lanuza M. Iron deficiency - An underrecognized problem in nonanemic and erythrocytic kidney transplant recipients: Risks and effects of ACEI and of iron treatment. In: *Transplantation Proceedings*. ; 2005. doi:10.1016/j.transproceed.2004.11.081
6. Camaschella C. Iron-Deficiency Anemia. *N Engl J Med*. 2015. doi:10.1056/NEJMra1401038
7. Eisenga MF, Dullaart RPF, Berger SP, et al. Association of hepcidin-25 with survival after kidney transplantation. *Eur J Clin Invest*. 2016. doi:10.1111/eci.12682
8. Eisenga MF, van Londen M, Leaf DE, et al. C-Terminal Fibroblast Growth Factor 23, Iron Deficiency, and Mortality in Renal Transplant Recipients. *J Am Soc Nephrol*. 2017. doi:10.1681/ASN.2016121350
9. Lam JR, Schneider JL, Quesenberry CP, Corley DA. Proton Pump Inhibitor and Histamine-2 Receptor Antagonist Use and Iron Deficiency. *Gastroenterology*. 2017. doi:10.1053/j.gastro.2016.11.023
10. Tran-Duy A, Connell NJ, Vanmolkot FH, et al. Use of proton pump inhibitors and risk of iron deficiency: a population-based case–control study. *Journal of Internal Medicine*. 2018.
11. Zheng S, Coyne DW, Joist H, et al. Iron deficiency anemia and iron losses after renal transplantation. *Transpl Int*. 2009. doi:10.1111/j.1432-2277.2008.00814.x
12. Murray-Kolb LE, Beard JL. Iron treatment normalizes cognitive functioning in young women. *Am J Clin Nutr*. 2007. doi:10.1093/ajcn/85.3.778
13. Blanton CA, Green MW, Kretsch MJ. Body iron is associated with cognitive executive planning function in college women. *Br J Nutr*. 2013. doi:10.1017/S0007114512002620
14. Bruner AB, Joffe A, Duggan AK, Casella JF, Brandt J. Randomised study of cognitive effects of iron supplementation in non-anaemic iron-deficient adolescent girls. *Lancet (London, England)*. 1996. doi:10.1016/S0140-6736(96)02341-0
15. Jankowska EA, Rozentryt P, Witkowska A, et al. Iron deficiency predicts impaired

exercise capacity in patients with systolic chronic heart failure. *J Card Fail*. 2011. doi:10.1016/j.cardfail.2011.08.003

16. Ebner N, Jankowska EA, Ponikowski P, et al. The impact of iron deficiency and anaemia on exercise capacity and outcomes in patients with chronic heart failure. Results from the Studies Investigating Co-morbidities Aggravating Heart Failure. *Int J Cardiol*. 2016. doi:10.1016/j.ijcard.2015.11.178
17. Bekfani T, Pellicori P, Morris D, et al. Iron deficiency in patients with heart failure with preserved ejection fraction and its association with reduced exercise capacity, muscle strength and quality of life. *Clinical Research in Cardiology*. 2018.
18. Hoes MF, Grote Beverborg N, Kijlstra JD, et al. Iron deficiency impairs contractility of human cardiomyocytes through decreased mitochondrial function. *Eur J Heart Fail*. 2018. doi:10.1002/ejhf.1154
19. Favrat B, Balck K, Breymann C, et al. Evaluation of a single dose of ferric carboxymaltose in fatigued, iron-deficient women - PREFER a randomized, placebo-controlled study. *PLoS One*. 2014. doi:10.1371/journal.pone.0094217
20. Jankowska EA, Tkaczyszyn M, Suchocki T, et al. Effects of intravenous iron therapy in iron-deficient patients with systolic heart failure: a meta-analysis of randomized controlled trials. *Eur J Heart Fail*. 2016. doi:10.1002/ejhf.473
21. Toblli JE, Lombraña A, Duarte P, Di Gennaro F. Intravenous Iron Reduces NT-Pro-Brain Natriuretic Peptide in Anemic Patients With Chronic Heart Failure and Renal Insufficiency. *J Am Coll Cardiol*. 2007. doi:10.1016/j.jacc.2007.07.029
22. Toblli JE, Di Gennaro F, Rivas C. Changes in echocardiographic parameters in iron deficiency patients with heart failure and chronic kidney disease treated with intravenous iron. *Hear Lung Circ*. 2015. doi:10.1016/j.hlc.2014.12.161
23. Okonko DO, Grzeslo A, Witkowski T, et al. Effect of Intravenous Iron Sucrose on Exercise Tolerance in Anemic and Nonanemic Patients With Symptomatic Chronic Heart Failure and Iron Deficiency. FERRIC-HF: A Randomized, Controlled, Observer-Blinded Trial. *J Am Coll Cardiol*. 2008. doi:10.1016/j.jacc.2007.09.036
24. Anker SD, Comin Colet J, Filippatos G, et al. Ferric carboxymaltose in patients with heart failure and iron deficiency. *N Engl J Med*. 2009. doi:10.1056/NEJMoa0908355
25. Comin-Colet J, Lainscak M, Dickstein K, et al. The effect of intravenous ferric carboxymaltose on health-related quality of life in patients with chronic heart failure and iron deficiency: A subanalysis of the FAIR-HF study. *Eur Heart J*. 2013. doi:10.1093/eurheartj/ehr504
26. Ponikowski P, Filippatos G, Colet JC, et al. The impact of intravenous ferric carboxymaltose on renal function: An analysis of the FAIR-HF study. *Eur J Heart Fail*. 2015. doi:10.1002/ejhf.229

27. Ponikowski P, Van Veldhuisen DJ, Comin-Colet J, et al. Beneficial effects of long-term intravenous iron therapy with ferric carboxymaltose in patients with symptomatic heart failure and iron deficiency. *Eur Heart J*. 2015. doi:10.1093/eurheartj/ehu385
28. Van Veldhuisen DJ, Ponikowski P, Van Der Meer P, et al. Effect of Ferric Carboxymaltose on Exercise Capacity in Patients with Chronic Heart Failure and Iron Deficiency. In: *Circulation*. ; 2017. doi:10.1161/CIRCULATIONAHA.117.027497
29. Beck-Da-Silva L, Piardi D, Soder S, et al. IRON-HF study: A randomized trial to assess the effects of iron in heart failure patients with anemia. *Int J Cardiol*. 2013. doi:10.1016/j.ijcard.2013.04.181
30. Eisenga MF, Diepenbroek A, Swinkels DW, Bakker SJL, van der Meer P, Gaillard CAJM. [Parenteral iron therapy in chronic kidney disease or chronic heart failure]. *Ned Tijdschr Geneeskd*. 2015.
31. Macdougall IC, Bock AH, Carrera F, et al. FIND-CKD: a randomized trial of intravenous ferric carboxymaltose versus oral iron in patients with chronic kidney disease and iron deficiency anaemia. *Nephrol Dial Transplant*. 2014. doi:10.1093/ndt/gfu201
32. Moore RA, Gaskell H, Rose P, Allan J. Meta-analysis of efficacy and safety of intravenous ferric carboxymaltose (Ferinject) from clinical trial reports and published trial data. *BMC Blood Disord*. 2011. doi:10.1186/1471-2326-11-4
33. Lyseng-Williamson KA, Keating GM. Ferric carboxymaltose: A review of its use in iron-deficiency anaemia. *Drugs*. 2009. doi:10.2165/00003495-200969060-00007
34. Group KW. KDIGO clinical practice guideline for the care of kidney transplant recipients. *Am J Transplant*. 2009. doi:10.1111/j.1600-6143.2009.02834.x
35. Isakova T, Gutiérrez OM, Wolf M. A blueprint for randomized trials targeting phosphorus metabolism in chronic kidney disease. *Kidney Int*. 2009. doi:10.1038/ki.2009.246
36. Wolf M. Fibroblast growth factor 23 and the future of phosphorus management. *Curr Opin Nephrol Hypertens*. 2009. doi:10.1097/MNH.0b013e328331a8c8
37. Wolf M. Update on fibroblast growth factor 23 in chronic kidney disease. *Kidney Int*. 2012. doi:10.1038/ki.2012.176
38. Scialla JJ, Wolf M. Roles of phosphate and fibroblast growth factor 23 in cardiovascular disease. *Nat Rev Nephrol*. 2014. doi:10.1038/nrneph.2014.49
39. Faul C, Amaral AP, Oskouei B, et al. FGF23 induces left ventricular hypertrophy. *J Clin Invest*. 2011. doi:10.1172/JCI46122
40. Di Marco GS, Reuter S, Kentrup D, et al. Treatment of established left ventricular hypertrophy with fibroblast growth factor receptor blockade in an animal model of CKD. *Nephrol Dial Transplant*. 2014. doi:10.1093/ndt/gfu190

41. Humalda JK, Lambers Heerspink HJ, Kwakernaak AJ, et al. Fibroblast growth factor 23 and the antiproteinuric response to dietary sodium restriction during renin-angiotensin-aldosterone system blockade. *Am J Kidney Dis*. 2015. doi:10.1053/j.ajkd.2014.07.022
42. de Jong MA, Mirkovic K, Mencke R, et al. Fibroblast growth factor 23 modifies the pharmacological effects of angiotensin receptor blockade in experimental renal fibrosis. *Nephrol Dial Transplant*. 2016. doi:10.1093/ndt/gfw105
43. Bhan I, Shah A, Holmes J, et al. Post-transplant hypophosphatemia: Tertiary "Hyper-Phosphatoninism"? *Kidney Int*. 2006. doi:10.1038/sj.ki.5001788
44. Evenepoel P, Naesens M, Claes K, Kuypers D, Vanrenterghem Y. Tertiary "hyperphosphatoninism" accentuates hypophosphatemia and suppresses calcitriol levels in renal transplant recipients. *Am J Transplant*. 2007. doi:10.1111/j.1600-6143.2007.01753.x
45. Wolf M, Weir MR, Kopyt N, et al. A prospective cohort study of mineral metabolism after kidney transplantation. In: *Transplantation*. ; 2016. doi:10.1097/TP.0000000000000823
46. Van Londen M, M. Aarts B, Deetman PE, et al. Post-transplant hypophosphatemia and the risk of death-censored graft failure and mortality after kidney transplantation. *Clin J Am Soc Nephrol*. 2017. doi:10.2215/CJN.10270916
47. Wolf M, Molnar MZ, Amaral AP, et al. Elevated Fibroblast Growth Factor 23 is a Risk Factor for Kidney Transplant Loss and Mortality. *J Am Soc Nephrol*. 2011. doi:10.1681/ASN.2010080894
48. Baia LC, Humalda JK, Vervloet MG, Navis G, Bakker SJL, de Borst MH. Fibroblast growth factor 23 and cardiovascular mortality after kidney transplantation. *Clin J Am Soc Nephrol*. 2013. doi:10.2215/CJN.01880213
49. Wolf M, Koch TA, Bregman DB. Effects of iron deficiency anemia and its treatment on fibroblast growth factor 23 and phosphate homeostasis in women. *J Bone Miner Res*. 2013. doi:10.1002/jbmr.1923
50. Van Breda GF, Bongartz LG, Zhuang W, et al. Cardiac Hepcidin Expression Associates with Injury Independent of Iron. *Am J Nephrol*. 2016. doi:10.1159/000449419
51. Block GA, Fishbane S, Rodriguez M, et al. A 12-Week, Double-Blind, Placebo-Controlled Trial of Ferric Citrate for the Treatment of Iron Deficiency Anemia and Reduction of Serum Phosphate in Patients with CKD Stages 3-5. *Am J Kidney Dis*. 2015. doi:10.1053/j.ajkd.2014.10.014
52. Fukao W, Hasuike Y, Yamakawa T, et al. Oral Versus Intravenous Iron Supplementation for the Treatment of Iron Deficiency Anemia in Patients on

Maintenance Hemodialysis—Effect on Fibroblast Growth Factor-23 Metabolism. *J Ren Nutr*. 2018. doi:10.1053/j.jrn.2017.12.009

53. Roberts MA, Huang L, Lee D, et al. Effects of intravenous iron on fibroblast growth factor 23 (FGF23) in haemodialysis patients: A randomized controlled trial. *BMC Nephrol*. 2016. doi:10.1186/s12882-016-0391-7
54. Schouten BJ, Hunt PJ, Livesey JH, Frampton CM, Soule SG. FGF23 elevation and hypophosphatemia after intravenous iron polymaltose: A prospective study. *J Clin Endocrinol Metab*. 2009. doi:10.1210/jc.2008-2396
55. Mani LY, Nseir G, Venetz JP, Pascual M. Severe hypophosphatemia after intravenous administration of iron carboxymaltose in a stable renal transplant recipient. *Transplantation*. 2010. doi:10.1097/TP.0b013e3181f00a18
56. Sari V, Atiqi R, Hoorn EJ, Heijboer AC, Van Gelder T, Hesselink DA. Ferric carboxymaltose-induced hypophosphataemia after kidney transplantation. *Neth J Med*. 2017.
57. Schaefer B, Effenberger M, Zoller H. Iron metabolism in transplantation. *Transpl Int*. 2014. doi:10.1111/tri.12374
58. Cherukuri A, Rothstein DM, Clark B, et al. Immunologic Human Renal Allograft Injury Associates with an Altered IL-10/TNF- Expression Ratio in Regulatory B Cells. *J Am Soc Nephrol*. 2014. doi:10.1681/ASN.2013080837
59. Jiang Y, Li C, Wu Q, et al. Iron-dependent histone 3 lysine 9 demethylation controls B cell proliferation and humoral immune responses. *Nat Commun*. 2019. doi:10.1038/s41467-019-11002-5
60. Stoffel NU, Uyoga MA, Mutuku FM, et al. Iron Deficiency Anemia at Time of Vaccination Predicts Decreased Vaccine Response and Iron Supplementation at Time of Vaccination Increases Humoral Vaccine Response: A Birth Cohort Study and a Randomized Trial Follow-Up Study in Kenyan Infants. *Front Immunol*. 2020. doi:10.3389/fimmu.2020.01313
61. Krueger KM, Ison MG, Ghossein C. Practical Guide to Vaccination in All Stages of CKD, Including Patients Treated by Dialysis or Kidney Transplantation. *Am J Kidney Dis*. 2020. doi:10.1053/j.ajkd.2019.06.014
62. Vaugier C, Amano MT, Chemouny JM, et al. Serum Iron Protects from Renal Postischemic Injury. *J Am Soc Nephrol*. 2017. doi:10.1681/ASN.2016080926
63. Fricke WF, Maddox C, Song Y, Bromberg JS. Human microbiota characterization in the course of renal transplantation. *Am J Transplant*. 2014. doi:10.1111/ajt.12588
64. Lee JR, Muthukumar T, Dadhania D, et al. Gut Microbial Community Structure and Complications After Kidney Transplantation. *Transplantation*. 2014. doi:10.1097/TP.0000000000000370

65. Ardalan M, Vahed SZ. Gut microbiota and renal transplant outcome. *Biomed Pharmacother*. 2017. doi:10.1016/j.biopha.2017.02.114
66. Kortman GAM, Raffatellu M, Swinkels DW, Tjalsma H. Nutritional iron turned inside out: Intestinal stress from a gut microbial perspective. *FEMS Microbiol Rev*. 2014. doi:10.1111/1574-6976.12086
67. Lee T, Clavel T, Smirnov K, et al. Oral versus intravenous iron replacement therapy distinctly alters the gut microbiota and metabolome in patients with IBD. *Gut*. 2016. doi:10.1136/gutjnl-2015-309940
68. Adams PC, Kertesz AE, Mclaren CE, Barr R, Bamford A, Chakrabarti SC. Population screening for hemochromatosis: A comparison of unbound iron- binding capacity, transferrin saturation, and C282Y genotyping in 5,211 voluntary blood donors. *Hepatology*. 2000. doi:10.1053/he.2000.6984
69. Phatak PD, Sham RL, Raubertas RF, et al. Prevalence of hereditary hemochromatosis in 16031 primary care patients. *Ann Intern Med*. 1998.
70. Riess KJ, Gourishankar S, Oreopoulos A, et al. Impaired arterial compliance and aerobic endurance in kidney transplant recipients. *Transplantation*. 2006. doi:10.1097/01.tp.0000233347.40682.21
71. Sample Size Calculator. https://www.stat.ubc.ca/~rollin/stats/ssize/n2.htm.
72. Public Assessment Report Ferinject. https://db.cbg-meb.nl/Pars/h33865.pdf.
73. Ferric Carboxymaltose. https://www.farmacotherapeutischkompas.nl/bladeren.
74. Braun. https://www.bbraun.ch/content/dam/catalog/bbraun/b.
75. Roger SD, Gaillard CA, Bock AH, et al. Safety of intravenous ferric carboxymaltose versus oral iron in patients with nondialysis-dependent CKD: An analysis of the 1-year FIND-CKD trial. *Nephrol Dial Transplant*. 2017. doi:10.1093/ndt/gfw264
76. Wolf M, Chertow GM, Macdougall IC, Kaper R, Krop J SW. Randomized trial of intravenous iron-induced hypophosphatemia. *JCI Insight*. 2018;6(3). doi:10.1172/jci.insight.124486.
77. Blazevic A, Hunze J, Boots JMM. Severe hypophosphataemia after intravenous iron administration. *Neth J Med*. 2014. doi:10.1136/bcr-2016-219160
78. Yamamoto S, Okada Y, Mori H, Fukumoto S, Tanaka Y. Fibroblast Growth Factor 23-Related Osteomalacia Caused by the Prolonged Administration of Saccharated Ferric Oxide. *Intern Med*. 2012. doi:10.2169/internalmedicine.51.7450
79. Bishay RH, Ganda K, Seibel MJ. Long-term iron polymaltose infusions associated with hypophosphataemic osteomalacia: a report of two cases and review of the literature. *Ther Adv Endocrinol Metab*. 2017. doi:10.1177/2042018816678363
80. van der Weerd NC, Grooteman MPC, Nubé MJ, ter Wee PM, Swinkels DW, Gaillard CAJM. Hepcidin in chronic kidney disease: Not an anaemia management tool, but

promising as a cardiovascular biomarker. *Neth J Med*. 2015.
81. Eisenga, MF., Gomes Neto AW., van Londen M. Rationale and Design of TransplantLines: a Prospective Cohort Study and Biobank of Solid Organ Transplant Recipients. *Unpublished*. 2018.
82. Zuyd Meetinstrumenten in de zorg. http://www.meetinstrumentenzorg.nl/.
83. Trampisch US, Franke J, Jedamzik N, Hinrichs T, Platen P. Optimal jamar dynamometer handle position to assess maximal isometric hand grip strength in epidemiological studies. *J Hand Surg Am*. 2012. doi:10.1016/j.jhsa.2012.08.014
84. Devine Medical. www.devinemedical.com.
85. Kwan MMS, Lin SI, Chen CH, Close JCT, Lord SR. Sensorimotor function, balance abilities and pain influence Timed Up and Go performance in older community-living people. *Aging Clin Exp Res*. 2011. doi:10.1007/BF03324960
86. Podsiadlo D, Richardson S. The timed “Up & Go”: a test of basic functional mobility for frail elderly persons. *J Am Geriatr Soc*. 1991. doi:http://www.ncbi.nlm.nih.gov/entrez/query.fcgi?cmd=Retrieve&db=PubMed&dopt=Citation&list_uids=1991946
87. Evaluation of health-related quality of life (HRQL) in patients with a serious life-threatening illness. https://www.uptodate.com/contents/evaluation-of-health-related-quality-of-life-hrql-in-patients-with-a-serious-life-threatening-illness/contributors.
88. EuroQol. https://euroqol.org/.
89. Hoeymans N, Van Lindert H, Westert GP. The health status of the Dutch population as assessed by the EQ-6D. *Qual Life Res*. 2005. doi:10.1007/s11136-004-1214-z
90. Graaf Ad DB. Cognitieve Screening Test. Handleiding. 1991.
91. Shulman KI, Shedletsky R S IL. The challenge of time: Clock □drawing and cognitive function in the elderly. *Int J Geriatr Psychiatry*. 1986;1 (2):135-140.
92. Schmand B, Lindeboom J van HF. Nederlandse leestest voor volwassenen: handleiding (NLV). 1992.
93. Wechsler D. WAIS-IV-NL: Wechsler Adult Intelligence Scale - Fourth Edition - Nederlandstalige bewerking: Technische handleiding en Afname en scoringshandleiding. 2012.
94. Saan R DB. De 15-Woorden Tests A en B. (Een voorlopige handleiding). 1986.
95. Luteijn F BD. GIT2: Groninger Intelligentie Test 2. 2004.
96. Schmand B, Groenink SC den DM. Letterfluency: psychometrische eigenschappen en Nederlandse normen. *Tijdschr Gerontol Geriatr*. 2008;39 (2):64-74.
97. Smith A. Symbol Digits Modalities Test Los Angeles: Western Psychological Services. 1968.
98. *Wet Medisch-Wetenschappelijk Onderzoek Met Mensen*.; 2017.

http://wetten.overheid.nl/BWBR0009408/2017-03-01.
99. ToetsingOnline. https://www.toetsingonline.nl/.
100. EudraVigilance. https://eudravigilance.ema.europa.eu/Decommissioned/Decommissioned.html.
101. *Declaration of Helsinki*.; 2013. https://www.wma.net/policies-post/wma-declaration-of-helsinki-ethical-principles-for-medical-research-involving-human-subjects/.
102. *Wet Geneeskundige Behandelovereenkomst*.; 2017. http://wetten.overheid.nl/BWBR0005290/2017-10-10#Boek7_Titeldeel7_Afdeling5.
103. *Clinical Trial Agreement NFU (Nederlandse Federatie van Universitair Medische Centra)*.; 2016. http://www.nfu.nl/wetenschap/kwaliteitsborging/klinisch-onderzoek/.
104. *CCMO Statement on Publication Policy*.; 2002. http://www.ccmo.nl/attachments/files/ccmo-statement-publicatiebeleid-3-02-en.pdf.
105. Rossum CTM van. *Dutch National Food Comsumption Survey 2007-2010*.; 2011. https://www.rivm.nl/dsresource?objectid=3f528864-36cb-400b-8411-a47db4f99df4&type=org&disposition=inline.
106. Nationaal Vergiftigingen Informatie Centrum. https://www.vergiftigingen.info/f?p=300:STOFMONOGRAFIE:6093132296988::::P1210_VERB_NO,P1210_FROM_PROD_NO,P1210_FROM_PRODSYN_NO,P1210_ROUTE:745,12571,1,1110.
