# Supplementary material for: Intravenous ferric carboxymaltose and exercise capacity in iron-deficient kidney transplant recipients: a randomised, placebo-controlled trial in the Netherlands
Source: eClinicalMedicine. 2026 Sep 1;99:104169. doi: 10.1016/j.eclinm.2026.104169 (PMC13571158; doi:10.1016/j.eclinm.2026.104169)
Supplement: Supplementary file 4 [file mmc4.docx]

*The following translations in Dutch were submitted by the authors and we reproduce them as supplied. They have not been peer reviewed. Our editorial processes have only been applied to the original abstract in English, which should serve as reference for this manuscript.*

Achtergrond: Het is onbekend of correctie van ijzertekort functionele uitkomsten na niertransplantatie verbetert. In dit onderzoek bestudeerden we intraveneus ferricarboxymaltose ter verbetering van de inspanningscapaciteit bij niertransplantatiepatiënten met ijzertekort.

Methoden: In deze multicenter, dubbelblinde, gerandomiseerde, placebogecontroleerde studie werden volwassen niertransplantatiepatiënten met ijzertekort (ferritine <100 μg/L of 100–299 μg/L met transferrinesaturatie <20%) die ten minste zes maanden na transplantatie waren, tussen 7 oktober 2019 en 16 februari 2024 geïncludeerd in twee Nederlandse universitaire ziekenhuizen. Deelnemers werden in een verhouding van 1:1 toegewezen aan vier intraveneuze doses van 500 mg ferricarboxymaltose of placebo met intervallen van zes weken. De primaire uitkomst was het verschil tussen de groepen in de verandering over 24 weken in de afstand bij de zesminutenlooptest. Secundaire uitkomsten omvatten hart-, nier-, spier- en cognitieve functie en kwaliteit van leven. De werkzaamheid werd volgens de toegewezen behandeling geanalyseerd in de vooraf vastgelegde werkzaamheidsanalysepopulatie van 148 deelnemers, met een per-protocolsensitiviteitsanalyse; de veiligheidsanalyse omvatte 154 deelnemers die ten minste één dosis studiemedicatie of placebo hadden gekregen. De studie werd geregistreerd bij ClinicalTrials.gov (NCT03769441).

Resultaten: Van de 155 gerandomiseerde deelnemers werden er zeven teruggetrokken op grond van een vooraf vastgelegd COVID-19-protocolamendement, waardoor 148 deelnemers in de werkzaamheidsanalysepopulatie overbleven (ferricarboxymaltose, n=75; placebo, n=73). De uitgangswaarde van de afstand bij de zesminutenlooptest was respectievelijk 515±91 m en 510±114 m. Ferricarboxymaltose verbeterde de afstand bij de zesminutenlooptest niet ten opzichte van placebo (gecorrigeerd gemiddeld verschil +9 m [95%-BI −10 tot +28]). Ferricarboxymaltose corrigeerde het ijzertekort en verhoogde de hemoglobineconcentratie wel, maar verbeterde de hartfunctie, spiermassa of -kracht, cognitieve prestaties of kwaliteit van leven niet. Er werden exploratieve verschillen tussen de groepen waargenomen in de geschatte glomerulaire filtratiesnelheid en fibroblastgroeifactor 23. Infecties en andere klinisch relevante bijwerkingen verschilden niet tussen de groepen.

Interpretatie: Ferricarboxymaltose verbeterde de inspanningscapaciteit of de meeste secundaire uitkomsten niet, wat pleit tegen routinematige ijzersuppletie ter verbetering van de functionele capaciteit. Toekomstige studies moeten de waargenomen verschillen in nierfunctie en fibroblastgroeifactor 23-concentraties en klinische langetermijnuitkomsten onderzoeken.

Financiering: Nierstichting, Hartstichting, CSL Vifor.
